# Predicting the most deleterious missense nsSNPs of the protein isoforms of the human HLA-G gene and in silico evaluation of their structural and functional consequences

**DOI:** 10.1186/s12863-020-00890-y

**Published:** 2020-08-31

**Authors:** Elaheh Emadi, Fatemeh Akhoundi, Seyed Mehdi Kalantar, Modjtaba Emadi-Baygi

**Affiliations:** 1 Department of Genetics, Faculty of Medicine, Shahid Sadoughi University of Medical Sciences, Yazd, Iran; 2 Department of Genetics, Faculty of Basic Sciences, Shahrekord University, Shahrekord, Iran; 3 Research Institute of Biotechnology, Shahrekord University, Shahrekord, Iran

**Keywords:** Deleterious SNPs, HLA-G gene, In silico analysis, Missense mutation, Structural and functional impact

## Abstract

**Background:**

The Human Leukocyte Antigen G (HLA-G) protein is an immune tolerogenic molecule with 7 isoforms. The change of expression level and some polymorphisms of the HLA-G gene are involved in various pathologies. Therefore, this study aimed to predict the most deleterious missense non-synonymous single nucleotide polymorphisms (nsSNPs) in HLA-G isoforms via in silico analyses and to examine structural and functional effects of the predicted nsSNPs on HLA-G isoforms.

**Results:**

Out of 301 reported SNPs in dbSNP, 35 missense SNPs in isoform 1, 35 missense SNPs in isoform 5, 8 missense SNPs in all membrane-bound HLA-G isoforms and 8 missense SNPs in all soluble HLA-G isoforms were predicted as deleterious by all eight servers (SIFT, PROVEAN, PolyPhen-2, I-Mutant 3.0, SNPs&GO, PhD-SNP, SNAP2, and MUpro). The Structural and functional effects of the predicted nsSNPs on HLA-G isoforms were determined by MutPred2 and HOPE servers, respectively. Consurf analyses showed that the majority of the predicted nsSNPs occur in conserved sites. I-TASSER and Chimera were used for modeling of the predicted nsSNPs. rs182801644 and rs771111444 were related to creating functional patterns in 5′UTR. 5 SNPs in 3′UTR of the HLA-G gene were predicted to affect the miRNA target sites. Kaplan-Meier analysis showed the HLA-G deregulation can serve as a prognostic marker for some cancers.

**Conclusions:**

The implementation of in silico SNP prioritization methods provides a great framework for the recognition of functional SNPs. The results obtained from the current study would be called laboratory investigations.

## Background

Single-Nucleotide Polymorphisms (SNPs) are the most copious type of human genetic sequence alterations that exist throughout the genome [1–3]. A missense mutation is a type of nonsynonymous (nsSNPs) substitution in which the one amino acid is substituted with another and may produce a mutated protein with structural and functional changes that may lead to disease. One of the main challenges for scientists is to identify SNPs that are pathogenic or related to a particular effect in humans [4]. Nowadays, deleterious nsSNPs in the desired gene can be identified using in -silico approaches. These approaches are reliable, user-friendly, fast, and low cost [5].

The major histocompatibility complex (MHC) is a group of genes encoding essential proteins for the adaptive immune system to identify fragments derived from pathogens [6]. In humans, The MHC complex is also called the human leukocyte antigen (HLA) complex [7]. The HLA genes have been classified into 3 classes I, II, and III. MHC class I genes are divided into two groups: major or classical (HLA-A, HLA-B, and HLA-C) and minor or non-classical (HLA-E, HLA-F, and HLA-G) [8], as they differ from each other by their genetic diversity, expression, structure, and functions [9].

The Human Leukocyte Antigen G (*HLA-G*) is a protein-coding gene on chromosome 6p21.3 and has an important function in modulation of the immune responses and diseases such as chronic viral infections, autoimmune disorders, transplantation and cancers [10, 11].

**Fig. 1:**
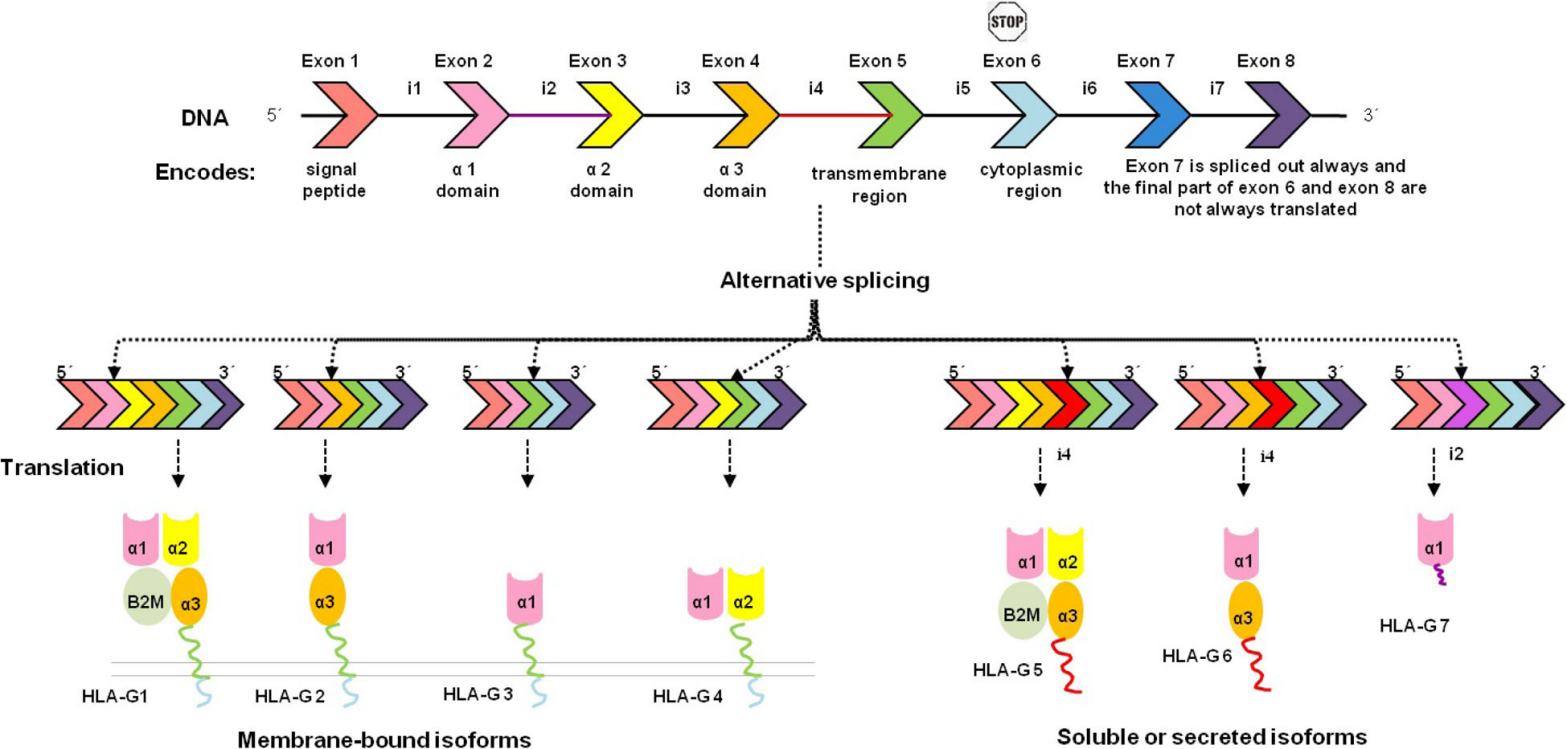
HLA-G heavy chain gene comprises 7 introns (i1-i7) and 8 exons (each with a distinctive color) with an internal stop codon in Exon 6. As shown in figure each exon encodes a discrete part of the heavy chain, except exon 7 and 8. Alternative splicing events of HLA-G primary transcript (exclusion exon 3 or/and exon 4 and keeping of intron 4 or intron 2 from the final gene transcript) generate seven isoforms. Soluble isoforms lack the transmembrane and cytoplasmic regions due to the intron retention, which includes an immature stop codon. HLA-G5 and HLA-G6 have a tail (21 amino acids) that plays a role in their solubility. HLA-G1 is the complete molecule. HLAG1 is homologous to HLA-G5 and both of them associate with B2M. The signal peptide (exon 1) and α1 domain (exon 2) are existing in all isoforms. Figure modified from Bainbridge et al. [14]

*HLA-G* gene displays low polymorphism but several mature mRNAs can be produced as a result of differential splicing of the primary transcript. The mature mRNAs encode 7 different protein isoforms, 4 of them being membrane-bound (HLA-G1 to G4), and 3 soluble or secreted (HLA-G5 to G7) [11]. Also, Roux et al. reported an inventory of novel HLA-G isoforms that have an extended 5′-region and lack the transmembrane and alpha-1 domains [12]. Soluble HLA-G1 (sHLA-G1) protein can be produced through the proteolysis activity of metalloprotease which maintains the functions of the membrane counterpart completely [13]. The general structure of an HLA-G protein consists of a heavy chain of 3 globular domains (α1, α2, and α3) and a light chain (β-2-microglobulin (B2M)) and a peptide (Fig. 1) [9].

HLA-G is involved in the control of the immune responses to maintain a fetomaternal tolerance in pregnancies [15]. Interaction of immune effector cells with HLA-G often introduces the suppression of them. The effects of inhibition are performed via three ITIM-bearing receptors expressed on various immune cells: ILT2/CD85/LIR-1, ILT4/CD85d/LIR-2, and KIR2DL4/CD158d [9, 16]. ILT2 is expressed by myeloid and lymphoid cells, ILT4 by myeloid cells, and KIR2DL4 by NK and T CD8+ [16]. The binding site of receptors to HLA-G is different. Interaction of HLA-G with ILT2 requires the association of the α3 domain with β2M but not for binding to ILT4. The KIR2DL4 binds to the α1 domain [11, 15, 16].

sHLA-G and membrane-bound HLA-G isoforms have alike functions. The membrane-bound HLA-G inhibits peripheral natural killer (NK) cytotoxicity and CD4^+^ cells directly through interaction with ILT2. The decidual NK (dNK) cells up-take and internalize HLA-G from the cell membrane of extravillous trophoblast cells through trogocytosis. HLA-G internalization results in maintaining low cytotoxicity and immunosuppressive status of dNK cells to protect the fetus versus dNKs activity and further release of a set of angiogenic factors to promote vascular remodeling and fetal growth at the beginning of pregnancy. The interaction of HLA-G with ILT2s on CD4^+^ T cells decreases the alloproliferative effect of CD4^+^ T cells. Binding sHLA-G to ILT4^+^ DCs leads to the generation of IL-10 and IL-10-producing DCs can promote the expansion of Tregs (CD4^+^ CD25^high^FOXP3) and Tr1s differentiation. Besides, the rapid reproduction, differentiation, and antibody production of B cells are inhibited due to HLA-G interplay with the LILRB1s on B cells. Moreover, apoptosis of CD8^+^ T cells through activation of the FasR/FasL pathway and endothelial cells are induced by HLA-G5 via interactions with CD8 receptor on CD8^+^ T cells and CD160 on endothelial cells [15–17].

*HLA-G* has a restricted tissue-specific protein expression in normal situations examples being extravillous cytotrophoblasts in the placenta, some immune cells, thymic medulla, and cornea. The neo-expression of HLA-G occurs in different pathological situations [15, 18–20].

The expression of *HLA-G* gene is adjusted mostly by a unique promoter region in comparison with other *HLA* genes and also at the post-transcriptional control level [21].

A single nucleotide polymorphism of a gene in the coding region or the regulatory region can lead to disease as a result of the expression change or structural and/or function alteration [4]. Most experimental and pathological studies of the HLA-G gene have been focused on polymorphisms in the promoter and 3ˊ UTR regions. The rate of polymorphisms in the coding sequence of this gene is low that indicates a powerful evolutionary pressure acting on the coding sequence [10].

Polymorphisms in the coding region may change the conformation of protein which could lead to modification of protein function including modulating immune responses, production of isoforms, peptide binding, and ability polymerization. HLA-G expression may change by altering the binding affinity of targeted sequences to transcriptional or post-transcriptional factors considering variations in the HLA-G promoter and 3ˊ UTR regions [10].

**Fig. 2:**
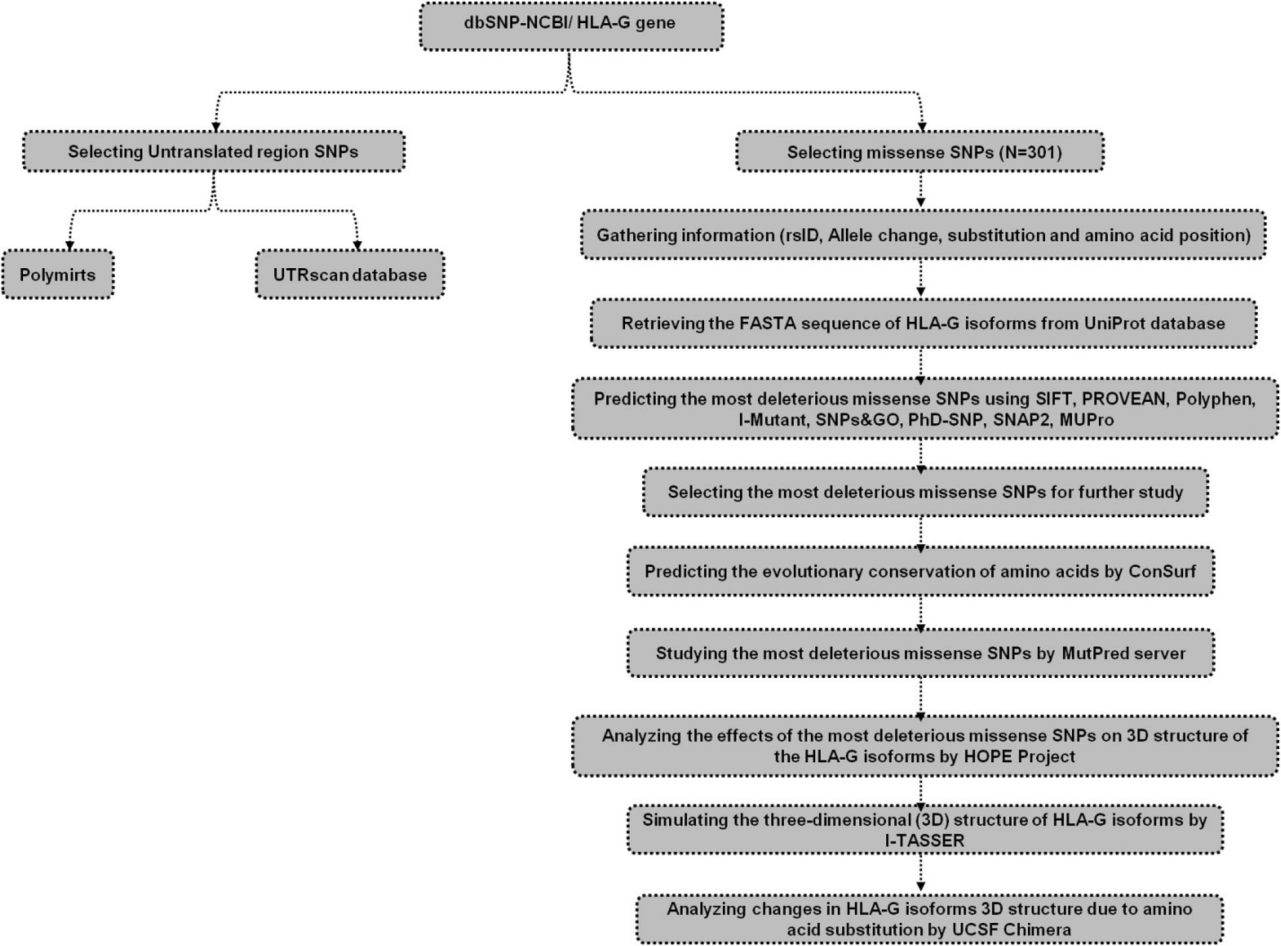
Flowchart of the different steps of the study

Concerning the important function of HLA-G in health and diseases in human, the main objectives of this study are to predict the most deleterious missense SNPs in HLA-G1 and HLA-G5, the common most deleterious missense SNPs in membrane-bound HLA-G isoforms, the common most deleterious missense SNPs in soluble HLA-G isoforms and finally to evaluate the impacts of the SNPs on the structure and function of HLA-G protein. The current study presents useful information about the most deleterious missense SNPs and their effects on the structure and function of HLA-G protein. In this paper, we also investigated the correlation between the survival rates of patients in some cancer types with HLA-G expression. The various steps of our study are shown in a flow chart (Fig. 2).

## Results

Currently, one of the valuable fields of computational genetic research is the identification of SNPs involved in diseases. At present, the advancement of computational biology methods has enabled us to detect the damaging SNPs in the objective genes. Computational methods are used to study the effect of nsSNPs on protein structure and function at the molecular level [22]. In this study, several computational methods were applied to determine the most deleterious common missense SNPs between soluble HLA-G isoforms and the most deleterious common missense SNPs between membrane-bound HLA-G isoforms as well as the most deleterious missense SNPs in HLA-G1 (the longest isoform protein of the HLA-G gene among membrane-bound HLA-G isoforms) and HLA-G5 isoforms (the longest isoform protein of the HLA-G gene among soluble HLA-G isoforms).

### SNP dataset of the *HLA-G* gene from NCBI dbSNP and protein sequences dataset

**Fig. 3:**
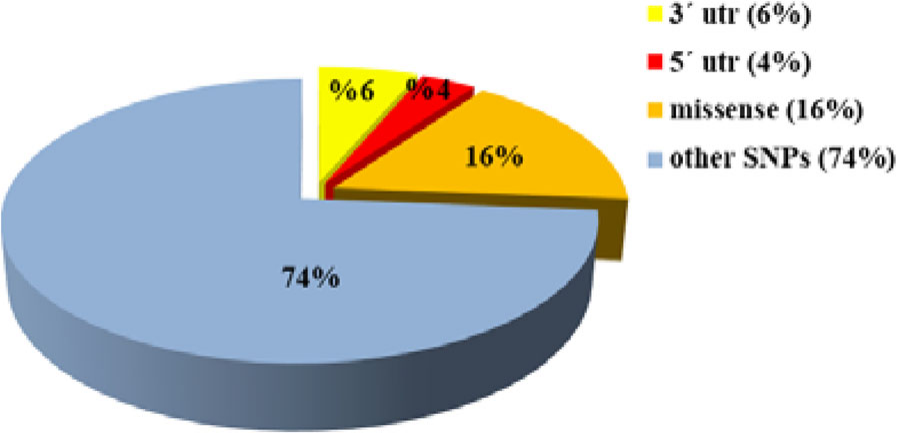
The 3-D pie-chart shows the percentage of missense SNPs, 5′ UTR, 3′ UTR and other types of SNPs in HLA-G gene (according to the dbSNP database on December 2018)

The desired SNPs of the *HLA-G* gene were retrieved from the NCBI dbSNP database because it is the most extensive SNP database [23]. SNPs retrieved from NCBI and their corresponding IMGT/HLA alleles are shown in the supplementary Table 1. Of the total reported SNPs in the human HLA-G gene sequence, 301 SNPs are missense (16.38%), 117 SNPs are in 3ˊUTR (6.36%) and 65 SNPs are in 5ˊUTR (3.53%). A pictorial description of the distribution of SNPs in the HLA-G gene represented in percentage terms is shown in Fig. 3. Most tools for analyzing protein require the amino acid sequence, for this reason, the protein sequences of seven HLA-G isoforms were retrieved from the UniProt database. The seven protein isoforms of HLA-G (HLA-G1–7) consist of 338, 246, 154, 246, 319, 227, and 116 amino acids respectively, and a 24-amino acid signal peptide.

### Identification of the most deleterious missense SNPs in HLA-G isoforms using several different servers

At present, there is an extensive range of computational tools used to predict the consequences of missense SNPs on protein structure and function. The in silico methods accuracy for prioritizing candidate deleterious SNPs can be enhanced by incorporating the results of diverse computational tools based on various parameters. Hence, we performed the concordance analysis with SIFT, PROVEAN, PolyPhen-2, I-Mutant 3.0, SNPs&GO, PhD-SNP, SNAP2, and MUpro techniques to predict the most deleterious nsSNPs from the SNP dataset. All the reported missense SNPs for HLA-G were submitted to eight mentioned in silico nsSNP prediction algorithms. We selected missense SNPs that are deleterious in all 8 algorithmic tools manually. Finally, out of total missense SNPs, 35 missense SNPs were predicted as deleterious in isoform 1 (HLA-G1) (Tables 1 and 2), 35 missense SNPs were predicted as deleterious in isoform 5 (HLA-G5) (Supplementary Tables 2 and 3), 8 missense SNPs were predicted as deleterious in all membrane-bound HLA-G isoforms (HLA-G1–4) (Supplementary Tables 4 and 5) and 8 missense SNPs were predicted as deleterious in all soluble HLA-G isoforms (Supplementary Tables 6 and 7) and all further investigations were held for only these missense SNPs.

**Table 1:** SNPs analyzed in isoform 1by SIFT, PROVEAN, Polyphen 2.0, I-mutant 3.0, SNPs&GO

Isoform 1												
**SNP rsID**	**Codons**	**Substitution**	**SIFT prediction**		**PROVEAN prediction**		**PolyPhen-2 prediction**		**I-Mutant DDG**		**SNPs&GO prediction**	
			Prediction	Score	Prediction	Score	Prediction	Score	SVM3 Prediction Effect	DDG Value	Prediction	**Probability**
rs17851921	CAC ⇒ CCC	H117P	DAMAGING	0	Deleterious	9.043	Probably damaging	1.000	Large Increase	0.33	Disease	0.805
	CAC ⇒ CTC	H117L			Deleterious	9.930	Probably damaging	1.000	Large Increase	0.65	Disease	0.794
rs111233577	CTG ⇒ CGG	L290R	DAMAGING	0	Deleterious	3.802	Probably damaging	1.000	Large Decrease	-1.44	Disease	0.786
rs142596947	CCT ⇒ ACT	P234T	DAMAGING	0	Deleterious	5.194	Probably damaging	0.991	Large Decrease	-1.08	Disease	0.557
rs144577485	CCC ⇒ GCC	P209A	DAMAGING	0	Deleterious	5.495	Possibly damaging	0.774	Large Decrease	-0.99	Disease	0.575
rs145097667	CAT ⇒ TAT	H287Y	DAMAGING	0	Deleterious	3.810	Probably damaging	0.999	Large Increase	0.54	Disease	0.743
rs572025435	AGG ⇒ AGT	R30S	DAMAGING	0	Deleterious	3.653	Probably damaging	0.991	Large Decrease	-1.17	Disease	0.542
rs748013931	ACC ⇒ CCC	T158P	DAMAGING	0	Deleterious	5.173	Probably damaging	1.000	Large Decrease	-0.52	Disease	0.696
rs749006959	TAT ⇒ TGT	Y142C	DAMAGING	0	Deleterious	8.189	Probably damaging	1.000	Large Increase	-1.13	Disease	0.749
rs750238738	ATC ⇒ TTC	I237F	DAMAGING	0	Deleterious	2.554	Probably damaging	0.993	Large Decrease	-1.46	Disease	0.777
rs756652306	GTG ⇒ GCG	V285A	DAMAGING	0	Deleterious	2.527	Probably damaging	0.998	Large Decrease	-1.04	Disease	0.617
rs760500349	CAG ⇒ CTG	Q266L	DAMAGING	0.04	Deleterious	4.451	Probably damaging	1.000	Large Increase	0.19	Disease	0.706
rs763201540	GAC ⇒ AAC	D53N	DAMAGING	0	Deleterious	3.842	Probably damaging	1.000	Large Decrease	-1.07	Disease	0.728
	GAC ⇒ TAC	D53Y			Deleterious	6.941	Probably damaging	1.000	Large Decrease	-0.29	Disease	0.872
rs765275727	TGG ⇒ CGG	W298R	DAMAGING	0	Deleterious	8.828	Probably damaging	1.000	Large Decrease	-0.8	Disease	0.713
rs770027530	TGC ⇒ TAC	C227Y	DAMAGING	0	Deleterious	7.461	Probably damaging	1.000	Large Decrease	-0.12	Disease	0.854
	TGC ⇒ TTC	C227F			Deleterious	7.461	Probably damaging	1.000	Large Decrease	0.06	Disease	0.882
rs770412396	CTG ⇒ CCG	L102P	DAMAGING	0.01	Deleterious	6.187	Probably damaging	1.000	Large Decrease	-1.63	Disease	0.786
rs772834879	TAC ⇒ CAC	Y142H	DAMAGING	0	Deleterious	-4.54	Probably damaging	1.000	Large Decrease	-1.31	Disease	0.635
rs780697086	TGC ⇒ AGC	C188S	DAMAGING	0	Deleterious	-8.51	Probably damaging	0.975	Large Decrease	-0.8	Disease	0.768
rs781774818	CCT ⇒ CAT	P259H	DAMAGING	0	Deleterious	5.764	Probably damaging	1.000	Large Decrease	-1.22	Disease	0.581
rs867319917	TGG ⇒ CGG	W157R	DAMAGING	0	Deleterious	12.658	Probably damaging	1.000	Large Decrease	-0.86	Disease	0.791
rs1200732770	GCC ⇒ GAC	A229D	DAMAGING	0	Deleterious	4.021	Probably damaging	1.000	Large Decrease	-0.87	Disease	0.775
rs1260086927	CAG ⇒ CCG	Q96P	DAMAGING	0.01	Deleterious	4.891	Probably damaging	0.996	Large Decrease	-0.4	Disease	0.524
rs1265409678	CTC ⇒ CGC	L294R	DAMAGING	0.01	Deleterious	3.443	Probably damaging	0.999	Large Decrease	-1.31	Disease	0.664
rs1317292772	GAT ⇒ AAT	D143N	DAMAGING	0	Deleterious	4.485	Probably damaging	0.996	Large Decrease	-0.55	Disease	0.662
	GAT ⇒ CAT	D143H			Deleterious	6.283	Possibly damaging	0.725	Large Decrease	-0.24	Disease	0.643
rs1379742188	CGC ⇒ AGC	R205S	DAMAGING	0.02	Deleterious	4.275	Possibly damaging	0.939	Large Decrease	-1.2	Disease	0.640
rs1390270595	TAC ⇒ TGC	Y51C	DAMAGING	0.03	Deleterious	6.756	Probably damaging	1.000	Large Decrease	-1.29	Disease	0.724
rs1397132797	CTG ⇒ CCG	L196P	DAMAGING	0.01	Deleterious	5.562	Probably damaging	1.000	Large Decrease	-1.66	Disease	0.760
rs1414848134	GAC ⇒ GTC	D54V	DAMAGING	0	Deleterious	6.752	Possibly damaging	0.893	Large Decrease	-1.04	Disease	0.874
rs143056505	CCT ⇒ CTT	P234L	DAMAGING	0	Deleterious	6.497	Possibly damaging	0.454	Large Decrease	-0.33	Disease	0.711
rs147253884	CCC ⇒ CGC	P209R	DAMAGING	0	Deleterious	6.191	Probably damaging	0.993	Large Decrease	-0.61	Disease	0.763
rs1475659109	CCC ⇒ CTC	P39L	DAMAGING	0	Deleterious	7.485	Probably damaging	1.000	Large Increase	-0.48	Disease	0.582
rs555347515	ATG ⇒ AAG	M29K	DAMAGING	0	Deleterious	3.427	Probably damaging	0.996	Large Decrease	-1.48	Disease	0.774
rs556645753	GAC ⇒ GGC	D153G	DAMAGING	0	Deleterious	6.309	Probably damaging	0.999	Large Decrease	-0.73	Disease	0.511
rs565858069	GAC ⇒ CAC	D130H	DAMAGING	0.03	Deleterious	5.927	Probably damaging	1.000	Large Decrease	-0.49	Disease	0.648
rs754527717	GAT ⇒ GGT	D244G	DAMAGING	0.01	Deleterious	3.915	Probably damaging	0.995	Large Decrease	-0.09	Disease	0.561
rs1161818149	CTG ⇒ CAG	L105Q	DAMAGING	0	Deleterious	4.572	Probably damaging	1.000	Large Decrease	-2.04	Disease	0.764
	CTG ⇒ CCG	L105P			Deleterious	5.349	Probably damaging	1.000	Large Decrease	-1.69	Disease	0.799

**Table 2:** SNPs analyzed in isoform 1by PhD-SNP, SNAP2, Mupro

Isoform 1									
SNP rsID	Codons	Substitution	PhD-SNP		SNAP2			MUpro	
			Prediction	Score	Prediction	Score	Expected Accuracy	Prediction	DDG Value
rs17851921	CAC ⇒ CCC	H117P	Disease	5	effect	94	95%	DECREASE	-0.94483558
	CAC ⇒ CTC	H117L	Disease	5	effect	82	91%	INCREASE	0.17248775
rs111233577	CTG ⇒ CGG	L290R	Disease	6	effect	52	75%	DECREASE	-1.4553025
rs142596947	CCT ⇒ ACT	P234T	Disease	8	effect	58	75%	DECREASE	-0.8378542
rs144577485	CCC ⇒ GCC	P209A	Disease	4	effect	30	66%	DECREASE	-1.3457662
rs145097667	CAT ⇒ TAT	H287Y	Disease	6	effect	79	85%	INCREASE	0.00175126
rs572025435	AGG ⇒ AGT	R30S	Disease	4	effect	2	53%	DECREASE	-1.582361
rs748013931	ACC ⇒ CCC	T158P	Disease	3	effect	56	75%	DECREASE	-1.0769639
rs749006959	TAT ⇒ TGT	Y142C	Disease	7	effect	69	80%	DECREASE	-1.7925753
rs750238738	ATC ⇒ TTC	I237F	Disease	5	effect	45	71%	DECREASE	-0.97093071
rs756652306	GTG ⇒ GCG	V285A	Disease	1	effect	36	66%	DECREASE	-0.89978472
rs760500349	CAG ⇒ CTG	Q266L	Disease	6	effect	78	85%	DECREASE	-0.05227910
rs763201540	GAC ⇒ AAC	D53N	Disease	5	effect	52	75%	DECREASE	-0.79149414
	GAC ⇒ TAC	D53Y	Disease	9	effect	84	91%	DECREASE	-0.45294328
rs765275727	TGG ⇒ CGG	W298R	Disease	5	effect	87	91%	DECREASE	-1.1870648
rs770027530	TGC ⇒ TAC	C227Y	Disease	8	effect	68	80%	DECREASE	-0.88892871
	TGC ⇒ TTC	C227F	Disease	9	effect	75	85%	DECREASE	-0.71910267
rs770412396	CTG ⇒ CCG	L102P	Disease	7	effect	79	85%	DECREASE	-2.238046
rs772834879	TAC ⇒ CAC	Y142H	Disease	5	effect	72	85%	DECREASE	-2.1531554
rs780697086	TGC ⇒ AGC	C188S	Disease	4	effect	75	85%	DECREASE	-1.0332928
rs781774818	CCT ⇒ CAT	P259H	Disease	5	effect	4	53%	DECREASE	-1.1893017
rs867319917	TGG ⇒ CGG	W157R	Disease	3	effect	93	95%	DECREASE	-0.91293557
rs1200732770	GCC ⇒ GAC	A229D	Disease	8	effect	75	85%	DECREASE	-0.6414612
rs1260086927	CAG ⇒ CCG	Q96P	Disease	6	effect	43	71%	DECREASE	-0.97929591
rs1265409678	CTC ⇒ CGC	L294R	Disease	6	effect	57	75%	DECREASE	-1.0427076
rs1317292772	GAT ⇒ AAT	D143N	Disease	4	effect	22	63%	DECREASE	-1.2006322
	GAT ⇒ CAT	D143H	Disease	9	effect	25	63%	DECREASE	-1.2840175
rs1379742188	CGC ⇒ AGC	R205S	Disease	5	effect	34	66%	DECREASE	-0.52183883
rs1390270595	TAC ⇒ TGC	Y51C	Disease	2	effect	39	66%	DECREASE	-0.83530596
rs1397132797	CTG ⇒ CCG	L196P	Disease	8	effect	58	75%	DECREASE	-2.0384073
rs1414848134	GAC ⇒ GTC	D54V	Disease	5	effect	75	85%	DECREASE	-0.37383825
rs1430565057	CCT ⇒ CTT	P234L	Disease	7	effect	81	91%	INCREASE	0.3342289
rs1472538844	CCC ⇒ CGC	P209R	Disease	3	effect	46	71%	DECREASE	-1.0509858
rs1475659109	CCC ⇒ CTC	P39L	Disease	5	effect	29	63%	DECREASE	-0.20370663
rs555347515	ATG ⇒ AAG	M29K	Disease	6	effect	25	63%	DECREASE	-1.7776487
rs556645753	GAC ⇒ GGC	D153G	Disease	2	effect	17	59%	DECREASE	-2.384922
rs565858069	GAC ⇒ CAC	D130H	Disease	5	effect	2	53%	DECREASE	-1.0708696
rs754527717	GAT ⇒ GGT	D244G	Disease	2	effect	7	53%	DECREASE	-1.7605711
rs1161818149	CTG ⇒ CAG	L105Q	Disease	3	effect	70	85%	DECREASE	-1.8766335
	CTG ⇒ CCG	L105P	Disease	6	effect	76	85%	DECREASE	-2.1510154

### Conservation analysis of the most deleterious nsSNPs in HLA-G isoforms by ConSurf sever

**Fig. 4:**
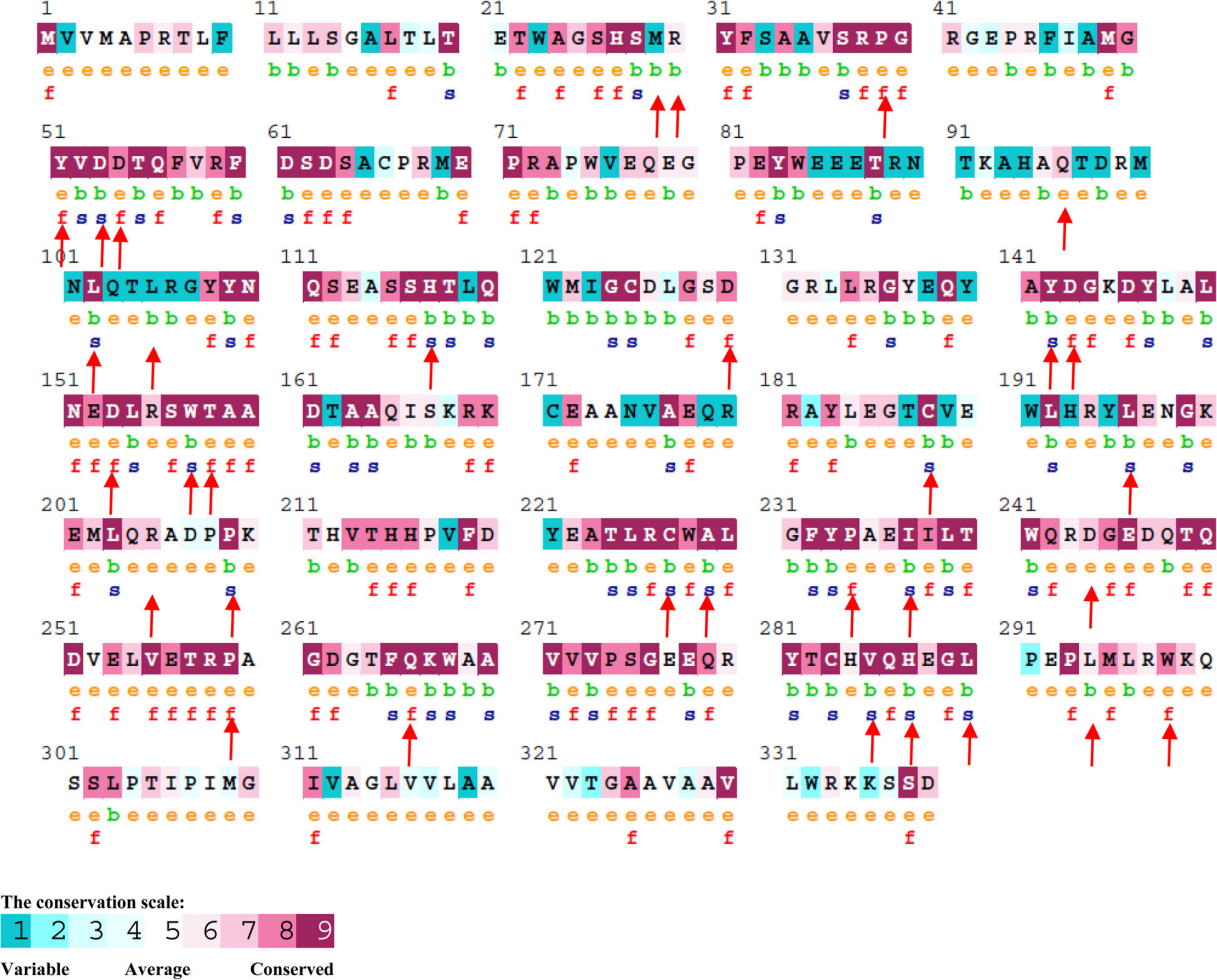
Consurf analysis of HLA-G1. The degree of conservation of amino acids was shown in the colouring scheme. The color intensity increases based on amino acids conservation grades e.g. turquoise indicates variable sites; white indicates average sites; maroon indicates evolutionarily conserved sites. The most deleterious predicted SNPs are marked below the sequence as red arrows. e is the exposed residue. b is the buried residue. f is an estimated functional residue (highly conserved and exposed). s is an estimated structural residue (highly conserved and buried)

Evolutionary information is essential to investigate further the possible impacts of deleterious nsSNPs [24]. The ConSurf web server characterizes the evolutionary conservation profile of amino acid residues in the protein and whether each amino acid is exposed (on protein surface) or buried (inside protein core) in the protein structure. For example, our ConSurf analysis showed that D53 is an exposed and conserved residue in all soluble HLA-G isoforms and is predicted to have a functional impact on soluble HLA-G isoforms whereas D53 is a buried and conserved residue in isoform 1 and is predicted a structural residue. The ConSurf server produces a colorimetric conservation score as a result. The residues with the utmost change are shown in blue and the conserved residues are shown in purple. The most highly conserved residues are significant for biological function and changing these residues has functional and structural impacts on the proteins [25]. The ConSurf results are compiled in Tables 3, supplementary Tables 8–10, Fig. 4, and supplementary Figs. 1–3. The results showed that the majority of the most deleterious nsSNPs (87.5% in isoform 1 and 86.66% in isoform 5) occur in conserved sites.

**Table 3:** Evolutionary conservation pattern of amino acids with solvent accessibility in HLA-G1 by ConSurf server

Isoform 1											
conservation score	exposed or buried	prediction	conservation score	exposed or buried	prediction	conservation score	exposed or buried	prediction	conservation score	exposed or buried	prediction
**M29**			**L105**			**C188**			**D244**		
1 (variable)	buried	-	1 (variable)	buried	-	9 (conserved)	buried	structural	7 (conserved)	exposed	**-**
**R30**			**H117**			**L196**			**P259**		
6 (average)	buried	-	9 (conserved)	buried	structural	9 (conserved)	buried	structural	9 (conserved)	exposed	functional
**P39**			**D130**			**R205**			**Q266**		
9 (conserved)	exposed	functional	8 (conserved)	exposed	functional	7 (conserved)	exposed	-	9 (conserved)	exposed	functional
**Y51**			**Y142**			**P209**			**V285**		
9 (conserved)	exposed	functional	9 (conserved)	buried	structural	9 (conserved)	buried	structural	9 (conserved)	buried	structural
**D53**			**D143**			**C227**			**H287**		
9 (conserved)	buried	structural	9 (conserved)	exposed	functional	9 (conserved)	buried	structural	9 (conserved)	buried	structural
**D54**			**D153**			**A229**			**L290**		
8 (conserved)	exposed	functional	9 (conserved)	exposed	functional	9 (conserved)	buried	structural	9 (conserved)	buried	structural
**Q96**			**W157**			**P234**			**L294**		
7 (conserved)	exposed	-	9 (conserved)	buried	structural	9 (conserved)	exposed	functional	6 (average)	buried	**-**
**L102**			**T158**			**I237**			**W298**		
9 (conserved)	buried	structural	9 (conserved)	exposed	functional	9 (conserved)	buried	structural	8 (conserved)	exposed	functional

Conservation score has a range of 1.0 to 9.0. Score 9 represents the most conserved and 1 represents the very variable amino acid. An amino acid, if is preserved and exposed, is a functional residue and if is preserved and buried, is a structural residue

### Prediction of structural and functional modifications due to the most deleterious SNPs on the HLA-G isoforms by MutPred server

The SNPs were predicted as most deleterious also investigated by the Mutpard server to predict the functional effects of SNPs. The most deleterious SNPs that were submitted to this server along with their predicted functional and structural effect on isoforms and the resultant probability scores were represented in Table 4 and supplementary Tables 11–13. For example, W157R in HLA-G1 was found to be highly deleterious with a g score of 0.936 and was predicted to cause the alteration in transmembrane protein with a p score of 0.000015, showing very confident hypothesis. W157R in HLA-G5 was found to be highly deleterious with a g score of 0.93 and was predicted to induce alteration in ordered interface with a p score of 0.0017, showing a very confident hypothesis. Gain of sulfation at D53 was predicted at D53Y in all membrane-bound HLA-G isoforms (g = 0.746 and p= 0.0044 in HLA-G1, g = 0.628 and p = 0.0088 in HLA-G2, g = 0.785 and p = 0.0051 in HLA-G3 and g = 0.75 and p = 0.0046 in HLA-G4). Loss of proteolytic cleavage at R30 was predicted at M29K in all soluble HLA-G isoforms (g = 0.688 and p = 0.0037 in HLA-G5, g = 0.754 and p = 0.0035 in HLA-G6 and g = 0.772 and p = 0.003 in HLA-G7).

**Table 4:** Prediction of functional effects of the most deleterious SNPs on the HLA-G1 by MutPred

Isoform 1 (ID: 79bb0a85-d537-4be0-bff8-d0fdb06b5211)					
Substitution	Mutpred score (>0.50 is considered pathogenic)	Molecular mechanism with ***p***-value <= 0.05	Probability	***P***-value	prediction
**H117P**	0.823	Altered Metal binding	0.27	0.02	confident hypotheses
		**Altered Transmembrane protein**	0.26	1.0e-03	**very confident hypotheses**
		Altered Ordered interface	0.25	0.03	confident hypotheses
**H117L**	0.665	Altered Transmembrane protein	0.28	4.8e-04	actionable hypotheses
		Loss of Strand	0.28	9.6e-03	actionable hypotheses
		Altered Metal binding	0.27	0.02	actionable hypotheses
		Altered Ordered interface	0.25	0.02	actionable hypotheses
**L290R**	0.644	Altered Transmembrane protein	0.13	0.02	actionable hypotheses
**P234T**	0.767	**Altered Ordered interface**	0.32	1.8e-03	**very confident hypotheses**
		**Altered Transmembrane protein**	0.25	1.3e-03	**very confident hypotheses**
**R30S**	0.792	**Altered Ordered interface**	0.29	5.0e-03	**very confident hypotheses**
		**Loss of Proteolytic cleavage at R30**	0.21	1.2e-03	**very confident hypotheses**
		Altered Stability	0.20	0.01	confident hypotheses
		**Altered Metal binding**	0.16	3.5e-03	**very confident hypotheses**
**T158P**	0.717	Altered Ordered interface	0.32	3.6e-03	actionable hypotheses
		Gain of Relative solvent accessibility	0.29	0.01	actionable hypotheses
		Altered Disordered interface	0.28	0.03	actionable hypotheses
		Loss of Allosteric site at W157	0.28	7.4e-03	actionable hypotheses
		Altered Metal binding	0.27	4.4e-03	actionable hypotheses
		Altered Transmembrane protein	0.27	6.6e-04	actionable hypotheses
		Loss of Helix	0.27	0.05	actionable hypotheses
		Altered Coiled coil	0.10	0.04	actionable hypotheses
**Y142C**	0.681	Altered Metal binding	0.74	1.1e-03	actionable hypotheses
		Altered Disordered interface	0.61	1.7e-04	actionable hypotheses
		Altered Ordered interface	0.50	1.7e-04	actionable hypotheses
		Altered Transmembrane protein	0.29	2.1e-04	actionable hypotheses
		Gain of Relative solvent accessibility	0.27	0.02	actionable hypotheses
		Loss of Acetylation at K145	0.20	0.04	actionable hypotheses
		Loss of Methylation at K145	0.17	0.01	actionable hypotheses
		Loss of Ubiquitylation at K145	0.16	0.04	actionable hypotheses
		Gain of Disulfide linkage at Y142	0.09	0.05	actionable hypotheses
		Loss of Sulfation at Y147	0.09	3.5e-03	actionable hypotheses
**I237F**	0.658	Altered Disordered interface	0.33	0.01	actionable hypotheses
		Altered Ordered interface	0.28	0.04	actionable hypotheses
		Altered Transmembrane protein	0.26	1.1e-03	actionable hypotheses
		Loss of Strand	0.26	0.04	actionable hypotheses
		Loss of Pyrrolidone carboxylic acid at Q242	0.06	0.02	actionable hypotheses
**Q266L**	0.444	Altered Ordered interface	0.28	0.03	-
		Gain of Relative solvent accessibility	0.26	0.03	
		Loss of Methylation at K267	0.23	3.8e-03	
		Gain of Acetylation at K267	0.22	0.02	
		Loss of Pyrrolidone carboxylic acid at Q266	0.21	1.8e-03	
		Altered Metal binding	0.20	0.03	
		Gain of Catalytic site at D262	0.16	0.02	
		Altered Transmembrane protein	0.15	0.01	
		Gain of Proteolytic cleavage at D262	0.14	0.02	
**D53Y**	0.746	Altered Ordered interface	0.39	1.1e-03	actionable hypotheses
		Altered Disordered interface =)	0.38	6.6e-03	actionable hypotheses
		Altered Metal binding	0.37	1.7e-03	actionable hypotheses actionable
		Altered Transmembrane protein	0.19	6.6e-03	hypotheses
		Loss of Proteolytic cleavage at D53	0.12	0.03	actionable hypotheses
		Gain of Pyrrolidone carboxylic acid at Q56	0.09	0.01	actionable hypotheses
		Gain of Sulfation at D53	0.07	4.4e-03	actionable hypotheses
**W298R**	0.822	**Altered Ordered interface**	0.36	2.1e-03	**very confident hypotheses**
		Altered Transmembrane protein	0.12	0.03	confident hypotheses
**C227Y**	0.87	**Altered Disordered interface**	0.42	3.2e-03	**very confident hypotheses**
		**Altered Ordered interface**	0.39	4.0e-04	**very confident hypotheses**
		**Altered Metal binding**	0.34	9.6e-03	**very confident hypotheses**
		**Altered Transmembrane protein**	0.31	1.2e-04	**very confident hypotheses**
		Loss of Helix	0.28	0.03	confident hypotheses
**C227F**	0.888	**Altered Metal binding**	0.37	7.8e-03	**very confident hypotheses**
		**Altered Disordered interface**	0.34	0.01	**very confident hypotheses**
		**Altered Ordered interface**	0.28	4.3e-03	**very confident hypotheses**
		Loss of Helix	0.27	0.04	confident hypotheses
		**Altered Transmembrane protein**	0.26	9.6e-04	**very confident hypotheses**
**L102P**	0.735	Altered Disordered interface	0.36	7.6e-03	actionable hypotheses
		Gain of Intrinsic disorder	0.34	0.02	actionable hypotheses
		Altered Transmembrane protein	0.31	1.1e-04	actionable hypotheses
		Loss of Helix	0.31	4.8e-03	actionable hypotheses
		Altered DNA binding	0.25	8.9e-03	actionable hypotheses
		Altered Stability	0.21	0.01	actionable hypotheses
		Loss of Proteolytic cleavage at R99	0.13	0.02	actionable hypotheses
**Y142H**	0.667	Altered Metal binding	0.78	8.2e-04	actionable hypotheses
		Altered Ordered interface	0.44	5.4e-04	actionable hypotheses
		Altered Disordered interface	0.35	8.9e-03	actionable hypotheses
		Altered Transmembrane protein	0.31	1.1e-04	actionable hypotheses
		Gain of Relative solvent accessibility	0.25	0.03	actionable hypotheses
		Gain of Acetylation at K145	0.20	0.04	actionable hypotheses
		Loss of Methylation at K145	0.17	0.01	actionable hypotheses
		Altered Stability	0.17	0.02	actionable hypotheses
		Loss of Ubiquitylation at K145 (	0.15	0.05	actionable hypotheses
		Altered Coiled coil	0.10	0.04	actionable hypotheses
		Loss of Sulfation at Y147	0.09	3.5e-03	actionable hypotheses
**C188S**	0.795	Altered Disordered interface	0.29	0.03	confident hypotheses
		Altered Ordered interface	0.24	0.03	confident hypotheses
		Altered DNA binding	0.17	0.03	confident hypotheses
		Altered Transmembrane protein	0.09	0.05	confident hypotheses
**P259H**	0.607	Altered Metal binding	0.47	5.9e-03	actionable hypotheses
		Altered Ordered interface	0.28	0.04	actionable hypotheses
		Loss of Loop	0.27	0.02	actionable hypotheses
		Altered Transmembrane protein	0.20	5.0e-03	actionable hypotheses
		Gain of Catalytic site at D262	0.13	0.03	actionable hypotheses
		Loss of Proteolytic cleavage at D262	0.13	0.03	actionable hypotheses
**W157R**	0.936	**Altered Ordered interface**	0.37	1.8e-03	**very confident hypotheses**
		**Altered Transmembrane protein**	0.36	1.5e-05	**very confident hypotheses**
		**Gain of Relative solvent accessibility**	0.33	3.3e-03	**very confident hypotheses**
		Altered Disordered interface	0.30	0.02	confident hypotheses
		**Loss of Allosteric site at W157**	0.30	3.7e-03	**very confident hypotheses**
		**Altered Metal binding**	0.28	3.8e-03	**very confident hypotheses**
		Altered Coiled coil	0.27	0.01	confident hypotheses
**A229D**	0.843	**Altered Transmembrane protein**	0.34	2.4e-05	**very confident hypotheses**
		Altered Metal binding	0.29	0.02	confident hypotheses
		Gain of Relative solvent accessibility	0.28	0.02	confident hypotheses
		**Altered Ordered interface**	0.27	9.8e-03	**very confident hypotheses**
**Q96P**	0.683	Loss of Helix	0.30	9.0e-03	actionable hypotheses
		Altered Transmembrane protein	0.29	3.0e-04	actionable hypotheses
		Altered DNA binding	0.25	6.7e-03	actionable hypotheses
		Gain of Ubiquitylation at K92	0.15	0.04	actionable hypotheses
		Gain of Proteolytic cleavage at R99	0.13	0.02	actionable hypotheses
		Loss of Pyrrolidone carboxylic acid at Q96	0.10	0.01	actionable hypotheses
**L294R**	0.527	Altered Ordered interface	0.27	8.6e-03	actionable hypotheses
**D143N**	0.649	Altered Metal binding	0.38	1.5e-03	actionable hypotheses
		Altered Transmembrane protein	0.28	3.8e-04	actionable hypotheses
		Altered Ordered interface	0.28	0.04	actionable hypotheses
		Altered Disordered interface	0.28	0.03	actionable hypotheses
		Gain of Relative solvent accessibility	0.26	0.03	actionable hypotheses
		Loss of Acetylation at K145	0.19	0.05	actionable hypotheses
		Loss of Methylation at K145	0.16	0.01	actionable hypotheses
		Loss of Ubiquitylation at K145	0.15	0.04	actionable hypotheses
		Loss of Sulfation at Y147	0.09	3.4e-03	actionable hypotheses
**D143H**	0.738	Altered Metal binding	0.43	7.0e-03	actionable hypotheses
		Altered Transmembrane protein	0.32	7.8e-05	actionable hypotheses
		Altered Disordered interface	0.29	0.03	actionable hypotheses
		Altered Ordered interface	0.27	7.0e-03	actionable hypotheses
		Loss of Relative solvent accessibility	0.26	0.03	actionable hypotheses
		Loss of Acetylation at K145	0.19	0.05	actionable hypotheses
		Loss of Methylation at K145	0.17	0.01	actionable hypotheses
		Loss of Ubiquitylation at K145	0.16	0.03	actionable hypotheses
		Loss of Sulfation at Y147	0.09	3.3e-03	actionable hypotheses
**R205S**	0.617	Gain of Intrinsic disorder	0.34	0.02	actionable hypotheses
		Altered Ordered interface	0.29	0.03	actionable hypotheses
		Loss of Helix	0.27	0.05	actionable hypotheses
**Y51C**	0.715	Altered Disordered interface	0.60	1.9e-04	actionable hypotheses
		Altered Metal binding	0.53	4.1e-03	actionable hypotheses
		Altered Ordered interface	0.30	4.6e-03	actionable hypotheses
		Loss of Strand	0.27	0.02	actionable hypotheses
		Altered Transmembrane protein	0.20	5.0e-03	actionable hypotheses
		Loss of Proteolytic cleavage at D53	0.12	0.03	actionable hypotheses
		Altered Stability	0.12	0.03	actionable hypotheses
		Gain of Pyrrolidone carboxylic acid at Q56	0.08	0.02	actionable hypotheses
		Loss of Sulfation at Y51	0.03	0.02	actionable hypotheses
**L196P**	0.884	Altered Disordered interface	0.34	0.01	confident hypotheses
		**Altered Ordered interface**	0.27	9.0e-03	**very confident hypotheses**
		Altered DNA binding	0.16	0.04	confident hypotheses
		Altered Stability	0.14	0.02	confident hypotheses
**D54V**	0.718	Altered Metal binding	0.55	3.1e-04	actionable hypotheses
		Altered Disordered interface	0.40	4.2e-03	actionable hypotheses
		Altered Ordered interface	0.25	0.02	actionable hypotheses
		Altered Transmembrane protein	0.14	0.02	actionable hypotheses
		Loss of Proteolytic cleavage at D53	0.12	0.03	actionable hypotheses
		Gain of Pyrrolidone carboxylic acid at Q56	0.09	0.01	actionable hypotheses
		Loss of Sulfation at Y51	0.03	0.02	actionable hypotheses
**P234L**	0.809	**Altered Ordered interface**	0.33	1.5e-03	**very confident hypotheses**
		Altered Disordered interface	0.30	0.03	confident hypotheses
		**Altered Transmembrane protein**	0.29	2.0e-04	**very confident hypotheses**
		Loss of Strand	0.28	0.01	confident hypotheses
**P39L**	0.659	Altered Disordered interface	0.27	0.04	actionable hypotheses
		Loss of B-factor	0.26	0.04	actionable hypotheses
		Altered Transmembrane protein	0.24	1.6e-03	actionable hypotheses
		Altered DNA binding	0.23	9.6e-03	actionable hypotheses
		Gain of Proteolytic cleavage at R38	0.14	0.02	actionable hypotheses
**M29K**	0.720	Altered Ordered interface	0.26	0.02	actionable hypotheses
		Loss of Proteolytic cleavage at R30	0.20	2.6e-03	actionable hypotheses
		Altered Stability	0.13	0.03	actionable hypotheses
		Altered Transmembrane protein	0.12	0.03	actionable hypotheses
		Altered Metal binding	0.11	7.9e-03	actionable hypotheses
**D153G**	0.853	**Altered Metal binding**	0.45	1.6e-04	**very confident hypotheses**
		**Altered Disordered interface**	0.35	9.1e-03	**very confident hypotheses**
		**Loss of Relative solvent accessibility**	0.30	8.2e-03	**very confident hypotheses**
		**Gain of Strand**	0.30	3.1e-03	**very confident hypotheses**
		**Altered Transmembrane protein**	0.28	4.6e-04	**very confident hypotheses**
		Altered Ordered interface	0.26	0.01	confident hypotheses
		**Gain of Allosteric site at W157**	0.26	7.0e-03	**very confident hypotheses**
		Altered Stability	0.19	0.01	confident hypotheses
		Altered Coiled coil	0.10	0.04	confident hypotheses
**D130H**	0.724	Altered Transmembrane protein	0.30	1.9e-04	actionable hypotheses
		Altered Disordered interface	0.28	0.03	actionable hypotheses
		Altered Metal binding	0.25	0.03	actionable hypotheses
		Loss of Relative solvent accessibility	0.25	0.04	actionable hypotheses
		Altered Ordered interface	0.24	0.04	actionable hypotheses
		Gain of Disulfide linkage at C125	0.10	0.05	actionable hypotheses
		Gain of Catalytic site at D126	0.08	0.05	actionable hypotheses
**D244G**	0.705	Altered Ordered interface	0.30	0.02	actionable hypotheses
		Altered Disordered interface	0.28	0.04	actionable hypotheses
		Gain of Strand	0.27	0.02	actionable hypotheses
		Altered Transmembrane protein	0.26	8.2e-04	actionable hypotheses
		Altered Metal binding	0.18	0.03	actionable hypotheses
		Gain of Pyrrolidone carboxylic acid at Q248	0.15	4.6e-03	actionable hypotheses
		Loss of Proteolytic cleavage at R243	0.12	0.03	actionable hypotheses
		Loss of Catalytic site at R243	0.09	0.04	actionable hypotheses
**L105Q**	0.507	Altered Disordered interface	0.32	0.01	actionable hypotheses
		Gain of Intrinsic disorder	0.30	0.05	actionable hypotheses
		Altered Transmembrane protein	0.29	2.1e-04	actionable hypotheses
		Altered Ordered interface	0.26	0.01	actionable hypotheses
		Altered DNA binding	0.16	0.04	actionable hypotheses
		Altered Stability	0.15	0.02	actionable hypotheses
		Loss of N-linked glycosylation at N110	0.05	0.02	actionable hypotheses
**L105P**	0.702	Altered Disordered interface	0.33	0.01	actionable hypotheses
		Loss of Helix	0.29	0.01	actionable hypotheses
		Altered Transmembrane protein	0.28	3.7e-04	actionable hypotheses
		Altered Ordered interface	0.26	0.01	actionable hypotheses
		Altered Stability	0.20	0.01	actionable hypotheses
		Altered DNA binding	0.16	0.04	actionable hypotheses
		Gain of N-linked glycosylation at N110	0.05	0.02	actionable hypotheses

The predictions which are very confident hypotheses shown in bold font

### The structural analysis of the most deleterious selected SNPs on HLA-G isoforms by project Hope server

Project HOPE predicted the effects of amino acid substitutions on native structures of HLA-G isoforms, the hydrophobicity, charge, and size change between wild-type and mutant residue and model of the 3D structure. The HOPE reports indicated that there was no exact known structural information for HLA-G1, 3, and 5 isoforms, and HOPE built the models of them based on homologous structures while the 3D-structures of HLA-G2, 4, 6 and 7 isoforms were known. All results of the effects of the most deleterious predicted SNPs on structures of the HLA-G isoforms and the difference in physicochemical properties of amino acids of wild type and mutated residue are reported in detail in Additional file 2 and supplementary Tables 14–16. For instance, rs555347515 mutation caused amino acid substitution from methionine into a lysine at the 29th position (M29K). The inspection of this mutation on HLA-G1 showed the mutated residue is bigger than the wild-type residue and probably will not fit in the core of the protein and the mutant residue has a positive charge, while the wild-type residue is neutral, so the positive charge can lead to protein folding problems. Furthermore, the mutation will lead to the loss of hydrophobic interplays in the center of the protein. Additionally, the structural analysis of M29K on HLA-G1 showed this variation is located inside a cluster of residues annotated in UniProt as the Alpha-1 domain and can disturb the domain structure and function (Additional file 2). Moreover, A/G mutation (rs556645753) resulted in a change of the aspartic acid to glycine at the 153rd position (D153G). The inspection of this mutation on HLA-G5 showed the mutated residue is smaller than the wild-type residue and this might induce loss of interplays and a further hydrophobic residue that can lead to loss of hydrogen bonds and disturb correct confirmation. The negative charge of the wild-type residue will be lost upon this mutation and this can lead to loss of interactions with other molecules or residues. Moreover, the structural analysis of D153G on HLA-G5 showed this variation is located inside a cluster of residues annotated in UniProt as the Alpha-2 domain and can distract this domain and disturb its function. Glycines are very flexible and can abolish the needed rigidity of HLA-G5 in this area (supplementary Table 14).

### Modeling of protein

I-TASSER tool created the 5 high-quality 3D structures for each HLA-G isoform from its amino acid sequence. We submitted the protein sequence of each isoform without signal peptide as an input to I-TASSER because there were no most deleterious SNPs in the peptide signal sequence and removing signal peptide from the protein sequence can improve the speed of I-TASSER simulation without loss of modeling accuracy. I-TASSER used the top 10 templates which are structurally closest to query protein sequence to model the protein (supplementary Table 17). Among the 5 predicted models for each HLA-G isoform, the first model was selected because it had the highest confidence score (C-score) and it was used for further investigation using Chimera (Additional file 3). A greater level of C-score indicates a model with great confidence and conversely.

### Chimera software

Chimera viewer was utilized to visualize the structures of the HLA-G isoforms using the first model as predicted by I-TASSER (Additional file 4). Furthermore, the structural characteristics of amino acids in wild and mutant protein chains were visualized by Chimera (Additional file 5 and supplementary Tables 18–20). A physicochemical rationale may be presented for the impact on protein activity by visualizing the location of the mutant amino acids [26].

### Functional SNPs in UTR predicted by UTRscan tool

The total of the UTR SNPs was investigated by applying UTRscan. Then analyzing the functional elements for every UTR SNP, the result showed that rs182801644 was related to the creation of functional pattern of uORF, and rs771111444 was related to the creation of a functional patterns of uORF and IRES in 5′UTR (Table 5). The internal ribosome entry site (IRES) is an alternative translation initiation mechanism in a cap-independent process in comparison with the ordinary 5′-cap dependent ribosome scanning mechanism [27]. Upstream open reading frames (uORF) is in the 5’UTR of mRNA that can regulate eukaryotic gene expression [28].

**Table 5:** Table of HLA-G UTR SNPs with functional importance that were predicted by UTRscan tool

SNP ID	Nucleotide change	UTR position	Functional element change
rs182801644	C/T	5′ UTR	no pattern → uORF
rs771111444	C/G	5′ UTR	no pattern → IRES no pattern → uORF

### The functional SNPs located in 3′UTRs region predicted by PolymiTRS

3′ untranslated regions (UTR) as the putative target site for miRNAs is a significant gene expression regulator. The SNP in the 3′ UTR region may disrupt and/or create miRNA target sites. PolymiRTS database predicted functional SNPs in 3′ UTR of the HLA-G gene. Among all the SNPs in the 3′UTR region of the HLAG gene, 5 functional SNPs were predicted to affect the miRNA target sites. The details of the effect of these SNPs on the miRNA sites are listed in Table 6. Two SNPs, rs17179101 and rs1063320 disrupt 9 miRNA conserved sites (ancestral allele with support ≥2), while all of them produce 15 novel miRNA target sites.

**Table 6:** Prediction results of PolymiRTS database

dbSNP ID	miR ID	Conservation	miRSite	FunctionClass
rs1707	hsa-miR-5702	2	CTGACT**C**ctcttt	C
	hsa-miR-583	3	ctgact**C**CTCTTT	C
rs17179101	hsa-miR-4417	2	AGCCCA**C**ccctgt	D
	hsa-miR-4651	5	agcCCA**C**CCCtgt	D
	hsa-miR-541-3p	2	aGCCCA**C**Ccctgt	D
	hsa-miR-608	2	agcCCA**C**CCCtgt	D
	hsa-miR-654-5p	2	aGCCCA**C**Ccctgt	D
	hsa-miR-6756-5p	2	agCCCA**C**CCctgt	D
	hsa-miR-6766-5p	2	agCCCA**C**CCctgt	D
	hsa-miR-6782-5p	3	agccCA**C**CCCTgt	D
	hsa-miR-1587	2	AGCCCA**A**ccctgt	C
	hsa-miR-296-3p	6	agccCA**A**CCCTgt	C
	hsa-miR-3147	2	aGCCCA**A**Ccctgt	C
	hsa-miR-3620-5p	2	AGCCCA**A**ccctgt	C
	hsa-miR-4674	2	AGCCCA**A**ccctgt	C
	hsa-miR-6823-5p	3	agcccA**A**CCCTGt	C
	hsa-miR-92a-1-5p	2	agCCCA**A**CCctgt	C
rs180827037	hsa-miR-875-3p	5	tttcct**T**TTCCAG	C
rs138249160	hsa-miR-25-5p	2	tCTCCG**C**Ctctgt	C
	hsa-miR-6087	3	tctCCG**C**CTCtgt	C
rs1063320	hsa-miR-3619-3p	3	tgTGGT**C**CActga	C
	hsa-miR-4776-5p	3	tgTGGT**C**CActga	C
	hsa-miR-4800-5p	3	tgtGGT**C**CACtga	C
	hsa-miR-767-5p	3	tgTGGT**G**CActga	D

In miRsite, sequences of the miRNA sites were shown. The capital letters show bases complementary to the seed region and SNPs were shown in bold font.

### Protein-protein interactions analysis

**Fig. 5:**
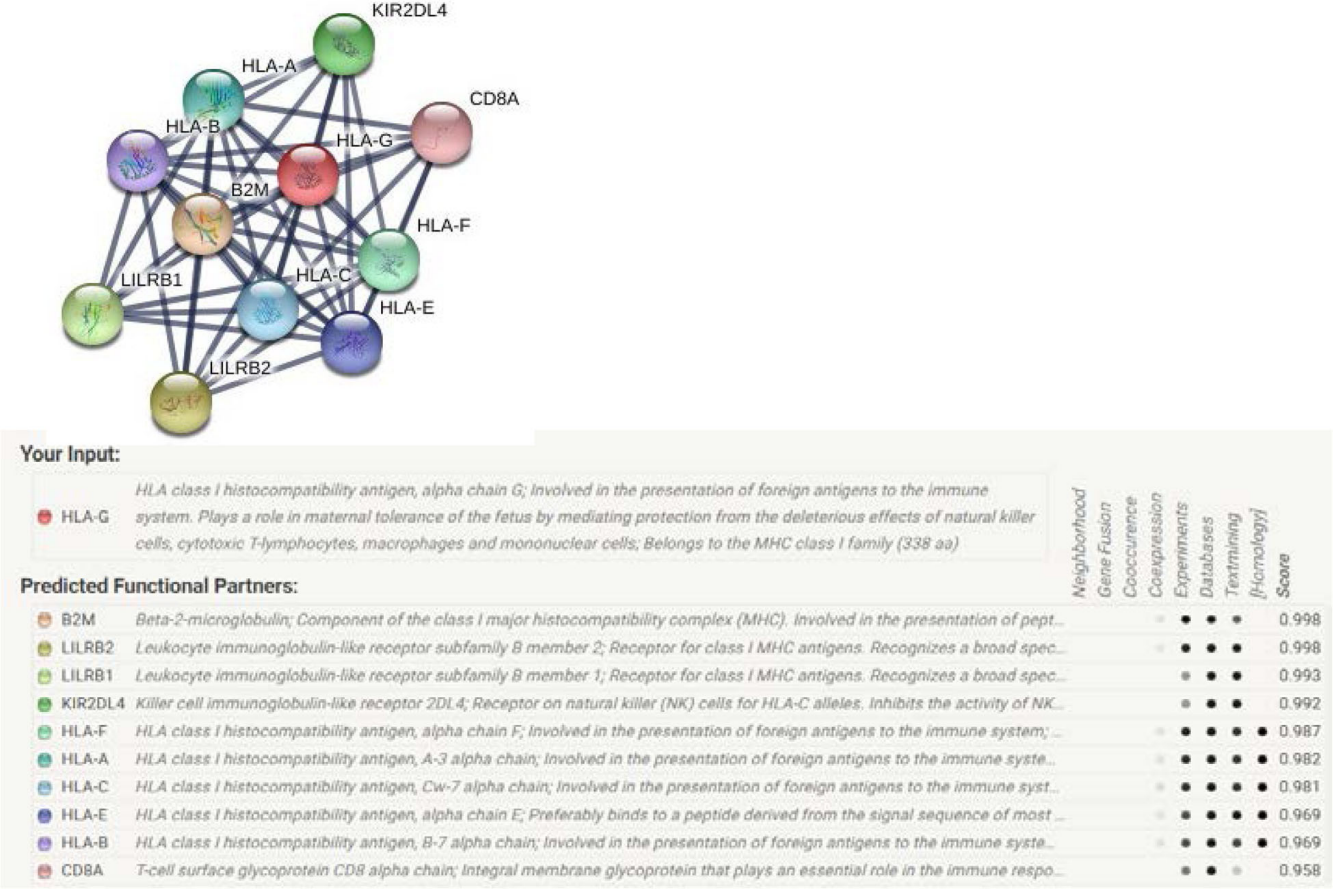
Protein–protein interaction network of HLAG with 10 partners

The mutation may change the structure of a protein and thus the function of protein may change. Therefore, mutated protein may interact with other proteins and lead to phenotypic effects. To investigate the interaction of HLA-G with various proteins, the STRING server was used. The interaction analysis revealed that HLAG is related to Beta-2-microglobulin (B2M), Leukocyte immunoglobulin-like receptor subfamily B member 2 (LILRB2), Leukocyte immunoglobulin-like receptor subfamily B member 1 (LILRB1), Killer cell immunoglobulin-like receptor 2DL4 (KIR2DL4), HLA class I histocompatibility antigen, alpha chain F (HLA-F), HLA class I histocompatibility antigen, A-3 alpha chain (HLA-A), HLA class I histocompatibility antigen, Cw-7 alpha chain (HLA-C), HLA class I histocompatibility antigen, alpha chain E(HLA-E), HLA class I histocompatibility antigen, B-7 alpha chain (HLA-B), T-cell surface glycoprotein CD8 alpha chain (CD8A) (Fig. 5).

### The effect of high and low expression levels of HLA-G on overall survival (OS) in patients with various cancers

Kaplan-Meier plotter was exerted to analyze the prognostic value of the HLA-G gene expression for breast, ovarian, lung, and gastric cancers by combining gene expression and cancer patient survival. The subjects were divided into 2 categories (high or low expression levels) according to the median expression of HLA-G. Subsequently, the correlation of expression levels and cancer patient’s overall survival rate was evaluated using the Kaplan-Meier plotter. Hazard ratio (HR) with 95% confidence intervals (CI) and logrank *p*-value were calculated.

**Fig. 6:**
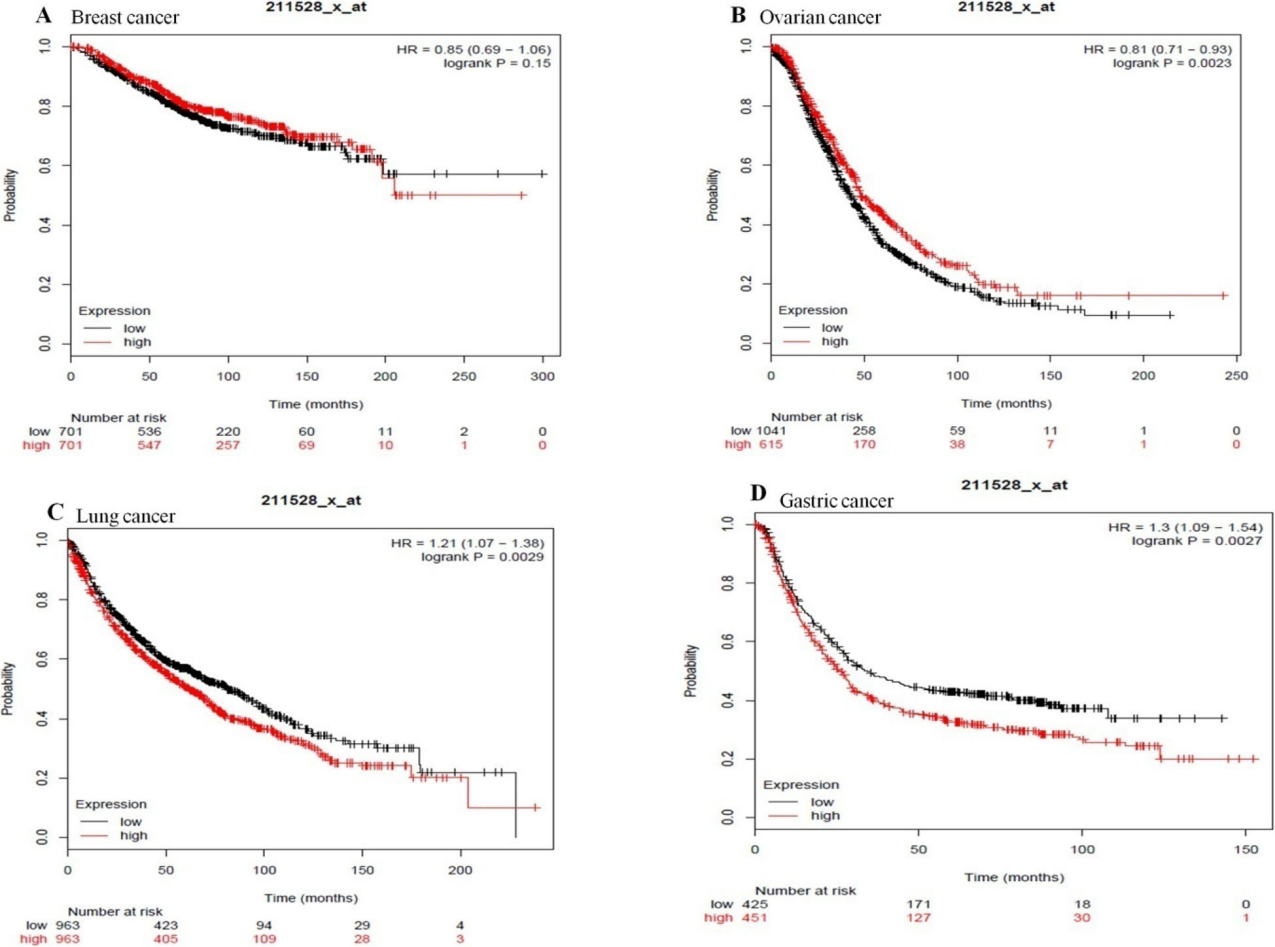
The correlation of deregulation of HLA-G gene and overall survival rate of the cancer patients was evaluated using Kaplan-Meier plotter

HLA-G gene in breast cancer had a hazard ratio (HR) = 0.85 (95% CI, 0.69–1.06) and logrank *p*-value = 0.15; therefore the result was not statistically significant (HLA-G deregulation had not the prognostic value). HLA-G gene in ovarian cancer had an HR = 0.81 (95% CI, 0.71–0.93) and logrank p-value = 0.0023; therefore the result was statistically significant (the relation between the high expression of HLA-G gene and more survival rate). HLA-G gene in lung cancer had a HR = 1.21 (95% CI, 1.07–1.38) and logrank p-value = 0.0029 and in gastric cancer HR = 1.3 (95% CI, 1.09–1.54) and logrank p-value = 0.0027; therefore the results were statistically significant (the relation between the low expression of HLA-G gene and more survival rate) (Fig. 6). The results showed that HLA-G deregulation has distinct implications in different types of cancers. This study shows, the HLA-G deregulation can serve as a prognostic marker for patients with ovarian, lung, and gastric cancer but not for breast cancer.

## Discussion

A large number of SNPs have been distributed throughout the human genome. Increasing evidence has suggested that SNPs are important and valuable in the search for the etiologies of human diseases/traits, the drug design, and human drug response [29, 30]. But the large number of SNPs causes a challenge for scientists because studying all SNPs with molecular approaches to choose target SNPs is an expensive, time-consuming and laborious task [29, 31, 32]. A better sense of genetic variations in susceptibility to disease and their phenotypic effects and reducing the number of them that should be screened in molecular studies may be provided by applying in silico methods [26, 33]. Among SNPs, missense SNPs are correlated with single amino acid substitution in the coded protein as a result of single nucleotide change in a codon that may have an intense impact on the structure and functionality of the relevant protein [4]. There is considerable data about SNPs in the dbSNP/NCBI database [34]. There were 301 missense mutations in the coding region of human HLA-G gene and in this study we focused on them in order to identify the most deleterious missense mutations that could modify the structure and function of the HLA-G isoforms. Identification of functional missense mutations and their role(s) may allow an individualized method for therapeutic goals [10]. HLA-G acts as an immune tolerogenic molecule, playing a role in various pathologies [10]. HLA-G primary mRNA is spliced into seven alternative mRNAs that encode 7 different isoforms of HLA-G protein: four membrane-bound (HLA-G1 to G4) and three soluble (HLA-G5 to G7) protein isoforms [35]. Full-length HLA-G protein exhibits a heavy chain consisting of α1 (residues 25 to 114), α2 (residues 115 to 206) and α3 (residues 207 to 298) domains and a light chain (B2M) [15, 36].

The HLA-Gl isoform consists of α1, α2 and α3 domains, transmembrane and cytoplasmic regions. The HLA-G2 isoform lacks the α2 domain. The HLA-G3 isoform does not comprise both the α2 and α3 domains. The HLA-G4 isoform lacks the α3 domain. The HLA-G5 isoform comprises the α1, α2 and α3 domains and lacks transmembrane and cytoplasmic domains as a result of intron 4 retention and encoding a C-terminal peptide sequence of twenty-one amino acid residues. HLA-G6 comprises α1 and α3 domains plus a C-terminal peptide sequence of twenty-one amino acid residues encoded by intron 4 retention and lacks transmembrane and cytoplasmic domains. The HLA-G7 isoform has only the α1 domain and lacks transmembrane and cytoplasmic domains as a result of intron 2 retention and encoding a C-terminal peptide sequence of two amino acid residues. All of these isoforms comprise α1 domain [36].

HLA-G expression has been widely studied in various disorders; nevertheless, the HLA-G gene polymorphism has not been evaluated to the same extent [10]. On the other hand, nearly half of the known gene-related damages for human hereditary diseases are amino acid substitutions. Consequently, screening of polymorphisms using in silico analyses to identify missense SNPs that affect the function of the protein and that are associated with the disease is an important task [29]. Therefore, in the present study, an attempt was made to predict the functional missense SNPs in human HLA-G isoforms. 301 missense SNPs of the human HLA-G gene were retrieved from dbSNP and were submitted to in silico tools to predict the functionally important missense SNPs in HLA-G1 and HLA-G5 and the common most deleterious missense SNPs in membrane-bound isoforms and in the soluble isoforms.

Existing in silico methods have diverse strengths and weaknesses in predicting the effect of nsSNP because every algorithm uses different parameters for prediction [37, 38]. Therefore, algorithms individually could not be considered as an accurate method for the prediction of functional SNPs [39]. In consequence, screening and prioritizing the candidate functional nsSNPs requires the implementation of different algorithms with different parameters and aspects (e.g. based on evolutionary information and protein structure and/or functional parameters) to combine the advantages of different methods, to enhance the accuracy and reliability of the predictions and to minimize the errors [5, 39–41]. As a general rule, in each study, at least four or five of these tools should be run to obtain a consensus on the effect of single nucleotide polymorphism on the structure and function of the desired protein [37]. In the current investigation, 8 different prediction algorithms were used as follows: SIFT, PROVEAN, PolyPhen-2, I-Mutant 3.0, SNPs&GO, PhD-SNP, SNAP2 and MUpro for the prediction of deleterious missense SNPs present in HLA-G isoforms. SIFT, PROVEAN, PhD SNP and SNP&GO tools predict damaging SNPs based only on the sequence of a protein. PolyPhen-2 and SNAP2 tools predict the functional effects of mutations based on the combination of protein 3D structure and multiple homolog sequence alignment [37]. I-Mutant 3.0 and MUPro tools investigate the effect of candidate SNPs on protein stability [41]. In our analyses, 35 missense substitutions of all the SNPs in HLA-G 1 isoform were predicted to be most deleterious SNPs by all the programs used. These 35 missense substitutions were classified according to the domain where they were located. Nine (25.71%) substitutions (rs555347515, rs572025435, rs1475659109, rs1390270595, rs763201540, rs1414848134, rs1260086927, rs770412396, rs1161818149) are located in the α1 domain, 11 (31.42%) substitutions (rs17851921, rs565858069, rs749006959, rs772834879, rs1317292772, rs556645753, rs867319917, rs748013931, rs780697086, rs1397132797, rs1379742188) are detected in the α2 domain and 15 (42.85%) substitutions (rs144577485, rs1472538844, rs770027530, rs1200732770, rs1430565057, rs142596947, rs750238738, rs754527717, rs781774818, rs760500349, rs756652306, rs145097667, rs111233577, rs1265409678, rs765275727) are located in the α3 domain. Thirty-five missense SNPs were found to be the most deleterious on the stability and function of HLA-G 5 isoform. Twelve (34.28%) substitutions (rs555347515, rs572025435, rs540632198, rs1475659109, rs1390270595, rs763201540, rs1414848134, rs138289952, rs1260086927, rs770412396, rs1161818149, rs776393668) are located in the α1 domain, 12 (34.28%) substitutions (rs17851921, rs565858069, rs749006959, rs772834879, rs1317292772, rs556645753, rs867319917, rs748013931, rs780697086, rs1397132797, rs1438362414, rs1379742188) are sited in the α2 domain and 11 (31.42%) substitutions (rs144577485, rs1472538844, rs770027530, rs1200732770, rs1430565057, rs142596947, rs750238738, rs781774818, rs760500349, rs145097667, rs765275727) are located in the α3 domain. Eight missense mutations in the α1 domain with positions M29K, R30S, Y51C, D53N/Y, D54V, Q96P, L102P and L105Q/P among all membrane-bound HLA-G isoforms and with positions M29K, F32C, Y51C, D53N/Y, D54V, Q96P, L102P, L105P between all soluble HLA-G isoforms were predicted as common deleterious missense mutations.

Evidence indicates that all three domains of the heavy chain of HLA-G molecule are involved in inhibiting immune response through interactions with other molecules, for instance, the α1 domain is an important KIR2DL4 recognition site and the LILRB1, LILRB2 and CD8 molecules interact with the α 3 domain. Nucleotide variations in these domains may affect the function of the HLA-G molecule. For example, the mutations around domain α1 and α2 affect peptide loading, peptide diversity, and T-cell recognition [10, 15, 42].

In this study, the selected variations were further investigated by other servers. For the rational prioritization of the selected most deleterious SNPs for further studies, an analysis of the evolutionary conservation of selected missense mutations was performed by ConSurf. The amino acids at the conserved regions of protein across species are biologically and functionally very important and SNPs that alter these amino acids may lead to structural and functional changes in the protein [29, 31]. We have shown that the selected deleterious SNPs in HLA-G1, HLA-G5, the membrane isoforms and the soluble isoforms were mostly in conserved positions and were functional and structural residues, which indicate these SNPs can be deleterious. The MutPred2 web-server predicted the possible molecular mechanisms that result from selected deleterious missense SNPs. The majority of the selected deleterious SNPs were predicted as ‘pathogenic’ (a g score greater than 0.5) and they are depicted as actionable, confident, and very confident hypotheses based on the g score and p score. The most predicted effects of very confident hypotheses in HLA-G1 and HLA-G5 were altered transmembrane protein and altered ordered interface. There was not any common predicted effect as very confident hypothesis among all of the membrane-bound HLA-G isoforms. The common predicted effect as very confident hypothesis among all of the soluble HLA-G isoforms was altered ordered interface resulting from F32C substitution. HOPE investigated the structural effects of the selected deleterious missense SNPs in HLA-G isoforms. The results revealed that nsSNPs are located in each of the three domains (α1, α2 and α3) of HLA-G. Since the function of any protein depends straightly on its tertiary structure, the modification in the structure of the domain can disrupt its function. The native protein 3D structures are very necessary for better understanding of the functional and structural effect of mutations. In the present study, because the 3D structure of all HLA-G isoforms is not available yet in the PDB database [43]; 3D structural models of native HLA-G isoforms were constructed by I-Tasser server and were visualized using Chimera software. Further, Chimera software was used to visualize the structural consequences of amino acid changes.

The HLA-G promoter region is special in the class of the HLA genes. The 5′ UTR and 3′ UTR regions of HLA-G gene display many polymorphic sites that may affect HLA-G expression and therefore tissue distribution in healthy and pathological conditions [10]. UTRscan analyzed the 5′ and 3′ UTR SNPs of the HLA-G gene. Two SNPs in the 5′ UTR were determined to create the functional patterns. The rs182801644 was related to creation of the functional pattern of uORF and rs771111444 was related to creation of the functional patterns of uORF and IRES in 5′UTR. The creation of uORF due to SNPs can deregulate the downstream original ORF expression and therefore be the cause of pathological conditions [44]. Furthermore, the presence of new IRES due to SNPs affects the regulation of mRNA translation [45]. To better understand the consequences of these UTR SNPs, investigation at the functional levels is needed.

PolymiRTS predicted that 5 functional SNPs are present in the HLA-G mRNA 3′ UTR, two of which them disrupt 9 target sites of the miRNA and all five SNPs create 15 new miRNA target sites. MicroRNAs play an important role in translation regulation. Thus disrupting or creating the microRNA target sites influences the regulation of gene and may lead to pathological conditions [10].

STRING analysis is a global way to understand protein-protein interactions. Any change in protein structure and function can affect its ability to interact with other molecules. STRING map showed the interaction of HLA-G with 10 different proteins. Some experimental studies confirm the interaction of HLA-G with these predicted proteins [9, 10, 14, 15, 17, 18, 46–53].

Lastly, the outcomes obtained from Kaplan Meier bioinformatics analyses indicated that the HLA-G gene deregulation affected the overall survival rate of patients with ovarian, lung and gastric cancer and had the prognostic significance. However, there are some controversies in relation to published original studies as presented in Table 7.

**Table 7:** The correlation of deregulation of HLA-G gene and overall survival rate of the cancer patients as reported by previous studies in comparison with our results

Type of cancer	Results found in our study	Results found in some previous studies	Controversy	Reference
Breast Cancer	HLA-G gene in breast cancer had a hazard ratio (HR) = 0.85 (95%CI, 0.69 − 1.06) and log-rank *p*-value= 0.15; therefore the result was not statistically significant.	In the whole cohort of patients, HLA-G showed no statistically significant difference in outcome between expression versus no expression for overall survival (*P* = 0.74).	No	[54]
		Breast cancer patients with positive HLA-G expression had a lower survival rate in comparison with negative HLA-G expression patients (*P* = 0.028).	Yes	[55]
		HLA-G upregulated expression was confirmed in breast cancer. HLA-G was associated with both improved relapse-free survival and overall survival.	Yes	[56]
		The expression of HLA-G was significantly higher in invasive ductal breast cancer patients with shorter survival time (*P* = 0.03).	Yes	[57]
		Breast cancer patients with HLA-G-positive tumor cells had shorter disease-free survival, though not significantly (*P* = 0.14).	No	[58]
Ovarian Cancer	The relation between the high expression of HLA-G gene and more survival rate was statistically significant (less number of patients at risk) (HR = 0.81 (95%CI, 0.71 − 0.93) and log-rank *p*-value= 0.0023)	Ovarian cancer patients with HLA-G expression >17% showed poor survival than those with HLA-G expression <17% group with a *P* value of 0.04.	Yes	[59]
		The HLA-G5/-G6 was expressed in 79.7% (94/118) of ovarian cancer lesions. lesion HLA-G5/-G6 expression was unrelated to clinicoparameters including histological type, patient age, FIGO stages and patient survival.	Yes	[60]
		Survival was prolonged when ovarian tumors expressed HLA-G (*P* = 0.008) and HLA-G was an independent predictor for better survival (*P* = 0.011). Furthermore, longer progression-free survival (*P* = 0.036) and response to chemotherapy (*P* = 0.014) was correlated with expression of HLA-G.	No	[61]
		The Kaplan-Meier analysis demonstrated no significant association between survival and HLA-G expression status in ovarian carcinoma patients.	Yes	[62]
Lung cancer	HLA-G gene in lung cancer had a HR = 1.21 (95%CI, 1.07 − 1.38) and log-rank *p*-value= 0.0029; therefore the result was statistically significant (the relation between the low expression of HLA-G gene and more survival rate)	The Higher sHLA-G level above the median (≥50 U/ml) in patients is associated with statistically significant shorter survival time in comparison to the lower sHLA-G expression (*P* < 0.0001).	No	[63]
		sHLA-G expression was observed in 34.0% (45/131) of the NSCLC lesions, which was unrelated to patient survival.	Yes	[64]
		Plasma sHLA-G above the median level (≥median, 32.0 U/ml) in NSCLC patients is strongly associated with shorter survival time (*P* = 0.044).	No	[65]
		Patients with sHLA-G <40 ng/ml (*p* = 0.073) showed prolonged overall survival.	No	[66]
		Patients with HLA-G positive tumors had a significantly shorter survival time than those with tumors that were HLA-G negative (*P* = 0.001).	No	[67]
		Survival analyses were shown that the HLA class I loss was correlated to recurrence-free survival time.	No	[68]
		Gastric carcinoma patients with HLA-G positive tumors had a significantly shorter survival time than those patients with tumors that were HLA-G negative (*P* = .001).	No	[69]
Gastric cancer	HLA-G gene in gastric cancer had a HR = 1.3 (95%CI, 1.09 − 1.54) and log-rank *p*-value= 0.0027; therefore the result was statistically significant (the relation between the low expression of HLA-G gene and more survival rate.	Kaplan-Meier analyses indicated that patients with HLA-G-positive gastric cancer had a poorer prognosis than those with HLA-G negative gastric cancer (*P* = 0.008).	No	[70]
		The overall median survival was worse in gastric adenocarcinoma patients with HLA-G-positive tumors compared to those with HLA-G-negative tumors (*p* < 0.0001).	No	[71]
		Kaplan–Meier analysis showed that gastric cancer patients with HLA-G expression had a significantly poorer overall survival than those without HLA-G expression at 5 years after the operation.	No	[72]
		The 5-year survival rate of gastric cancer patients in the HLA-G-positive group was significantly higher than the HLA-G-negative group.	Yes	[73]

Altogether, the findings of the analyses displayed probable alterations that may disrupt the structure and function of HLA-G protein. The deleterious missense mutations determined in this inspection may have functional effects in HLA-G deregulation and may lead to pathological conditions like cancer.

## Conclusion

The implementation of in silico SNP prioritization methods suggests a remarkable framework for the recognition of functional SNPs by reducing the number of alterations that should be screened in molecular studies. Further validation of the results obtained from the current study is recommended using clinical and/or laboratory investigations.

## Methods

### Extracting SNPs and protein sequences of HLA-G isoforms from the databases

In December 2018, NCBI dbSNP database [74] (https://www.ncbi.nlm.nih.gov/snp/) was used to collect information of missense nsSNPs and SNPs in the UTRs of human HLA-G gene. The amino acid sequences of seven human HLA-G isoforms (UniProt ID: P17693–1, P17693–2, P17693–3, P17693–4, P17693–5, P17693–6 and P17693–7) were obtained from the UniProt database [75] (https://www.uniprot.org/uniprot/P17693) in FASTA format for the next stages in this study.

### Predicting the most deleterious missense nsSNPs

We used eight online bioinformatics tools (SIFT, PROVEAN, PolyPhen-2, I-Mutant 3.0, SNPs&GO, PhD-SNP, SNAP2 and MUpro) to increase the precision of prediction of the most deleterious missense nsSNPs. Missense nsSNPs found to be most deleterious using these eight tools were further analyzed by several other programs in the next stages.

Sorting intolerant from tolerant (SIFT) [76] (available at https://sift.bii.a-star.edu.sg/) tool expresses whether a missense mutation at special position effects on the structure and function of protein molecule based on sequence homology and the physiochemical characteristics of substituted amino acid. SIFT computes the normalized probability score (SIFT score) for each substitution. The SIFT score has a range of 0.0 to 1.0. The amino acid substitution with a score greater than or equal to 0.05 (≥0.05) is predicted as tolerated (polymorphism) whereas a score less than 0.05 (< 0.05) is predicted to be damaging (related to disease).

Protein Variation Effect Analyzer (PROVEAN) (available at provean.jcvi.org/) is another sequence homology-based predictor. It is used to assess the possible functional influence of nonsynonymous (single or multiple nonsynonymous) and in-frame indel (insertions and deletions) variations on a protein. It predicts the variation as deleterious or natural, if the functional impact score is less than or equal to − 2.5 (≤ − 2.5) it is estimated deleterious; score above − 2.5 (> − 2.5) is estimated neutral [77].

Polymorphism Phenotyping version2 (PolyPhen-2) (available at genetics.bwh.harvard.edu/pph2/) is a combination of protein 3D structure and multiple homolog sequence alignment-based method. It predicts the potential consequences of single amino acid substitution on both protein function and structure. The prediction is provided as benign, possibly damaging and probably damaging according to the position-specific independent count (PSIC) scores difference between 2 variants (wild amino acid (aa1) and mutant amino acid (aa2)). PSIC score has a range of 0.0 to 1.0. The amino acid substitution with a score of 0.0 to 0.49 is predicted as benign, with a score of 0.5 to 0.89 is predicted as damaging and with a score of 0.9 to 1 is predicted as probably damaging [78, 79].

I-Mutant 3.0 (available at gpcr2.biocomp.unibo.it/cgi/predictors/I-Mutant3.0/I-Mutant3.0.cgi) is a web server including Support Vector Machine (SVM) based predictors suite. It predicts the effect of a particular amino acid substitution on the stability of protein under default parameters (at room temperature and neutral pH) starting from the protein sequence, mutational position and the corresponding novel residue. The protein stability change can disturb both protein function and structure [80]. I-Mutant 3.0 predicts the protein stability change in the unit of change in Gibbs free energy (ΔΔG or DDG). The DDG value (kcal/mol) is computed from the unfolding Gibbs free energy value of the mutant protein minus the unfolding Gibbs free energy value of native protein. The prediction is classified into three categories: neutral stability of the mutated protein (− 0.5 ≤ DDG ≤ 0.5 kcal/mol), a large decrease of stability of the mutated protein (≤ − 0.5 kcal/mol) and large increase of stability of the mutated protein (> 0.5 kcal/mol) [81].

Single Nucleotide Polymorphism Database (SNPs) & Gene Ontology (GO) (SNPs&GO) (available at snps.biofold.org/snps-and-go/snps-and-go.html) is a GO-integrated and single SVM-based predictor. It predicts whether an amino acid substitution is disease-associated or not using functional GO terms, 3D protein structure and protein sequence evolutionary information. The amino acid substitution is associated with the disease if the probability score is greater than 0.5 (> 0.5) [82].

Predictor of human Deleterious Single Nucleotide Polymorphisms (PhD-SNP) (available at snps.biofold.org/phd-snp/phd-snp.html) is a support vector machine (SVM) based server. This server determines whether a certain amino acid substitution is related to disease or neutral by protein sequence information, protein structure, conservation and solvent accessibility. The output is a probability index with a score of 0.0 to 1.0, when the score is higher than 0.5, the substituted amino acid is pathogenic [77, 81].

Screening for Non-Acceptable Polymorphisms (SNAP2) (available at https://rostlab.org/services/snap/) is a neural network-based tool that classifies amino acid substitutions into effective and neutral on protein function by taking a diversity of sequences and different characteristics into consideration. SNAP2 provides a list of all possible substitutions within the protein sequence with a score, functional effect (neutral or effect) and expected accuracy for any replacement. The expected accuracy shows the level of confidence for each prediction. The results are also displayed in heat map representation [83–85].

MUpro (available at mupro.proteomics.ics.uci.edu/) uses the Support Vector Machine (SVM) to assess the variation in the stability of the protein consequent to amino acid substitutions. The output is a confidence score among − 1 and 1. A confidence score < 0 indicates the substituted amino acid decreases the stability and a score > 0 indicates the substituted amino acid increases the stability [86].

### Selecting the most deleterious missense nsSNPs for further study

Missense nsSNPs that were predicted deleterious by all eight servers were selected for further study. The precision of prediction increases to a greater extent by incorporating the scores of all eight servers.

### Predicting the evolutionary conservation of the most deleterious missense nsSNPs by ConSurf

ConSurf web-server (available at consurf.tau.ac.il/) estimates the evolutionary conservation of each residue in a protein utilizing a Bayesian algorithm which often provides the possibility of identifying key structural and functional residues. The extent of conservation of residue at a specific position in a protein was computed by phylogenetic information of close homologous sequences. The measure of residue conservation is shown by the conservation score along with the color scheme as follows: 1–4 variable, 5–6 average, and 7–9 conserved. The ConSurf web - server also determines the buried (b) or exposed (e) residues of protein according to the HHPred 3D model. A residue is predicted functional residue if it is very conserved and exposed and a structural residue is predicted if it is very conserved and buried [87, 88].

### Studying the most deleterious missense nsSNPs by MutPred2 server

MutPred is a bioinformatics web server (available at mutpred.mutdb.org/). It predicts whether a particular missense mutation in a human protein is disease-associated or not, along with its structural and functional effects (effective molecular characteristics). The result of MutPred consists of two important scores (general (g) score and top 5 molecular properties score (p)), affected PROSITE and ELM motifs and changes of different structural and functional properties. The g score (MutPred score) expresses the probability that the missense mutation is disease-related. The g score is between 0.0 and 1.0. The g score > 0.5 means the substituted amino acid is probably pathogenic and if g score is > 0.75, the mutation is more assurance pathogenic. The top 5 molecular properties score (p) is a *P*-value that indicates whether predicted changes of functional and structural characteristics of the protein due to the particular missense mutation are statistically significant. The predicted change is confident if *p*-value is less than 0.05 (< 0.05) and is very confident if p-value is less than 0.01 (< 0.01). The given coalescences of high levels of g scores and low levels of p scores are called hypotheses. Any prediction according to the scores is put in one of these 3 groups: very confident hypotheses (g > 0.75 and *p* < 0.01), confident hypotheses (g > 0.75 and *p* < 0.05) and actionable hypotheses (g > 0.5 and p < 0.05 [89, 90].

### Analyzing the effects of the most deleterious missense SNPs on the 3D structure of the HLA-G isoforms by HOPE project

Project Have yOur Protein Explained (HOPE) is a web server (available at www.cmbi.ru.nl/hope/) that was used for the investigation of the impacts of a missense mutation on the native protein structure. HOPE will roll up and incorporate available information from UniProtKB, protein’s 3D structure and DAS-servers. As regards the exact 3D-structures of some HLA-G protein isoforms are unknown; HOPE built the model of them based on homologous structures. HOPE processes the gathered data and produces a report, including schematic structures of the wild-type and the mutant amino acids, differences in the properties of wild-type and mutant amino acids and the impacts of a substituted amino acid on the protein structure along with figures and animations [91].

### Simulating the three-dimensional (3D) structure of HLA-G isoforms by I-TASSER

To investigate the impact of missense mutations on the structure protein, simulating the protein structure is essential. Iterative Threading ASSEmbly Refinement (I-TASSER) (available at https://zhanglab.ccmb.med.umich.edu/I-TASSER/) is a united program to create the complete protein model and predict protein function based upon the sequence-to-structure-to-function paradigm. Therefore, we used I-TASSER to achieve the high-quality three-dimensional (3D) models of HLA-G protein isoforms by submitting their amino acid sequences in FASTA format. The models are created by excising continuous fragments from threading alignments and iterative structural assembly simulations and their functions are derived by matching the 3D models with other known proteins structurally. I-TASSER produces a report, including predicted secondary and tertiary structures, functional annotations and Gene Ontology terms. The accuracy of predicted models is reflected in the form of the confidence score (C-score). The C-score range is between − 5 and 2. The more values of C-score display higher confidence for the predicted model. Five three-dimensional (3D) models were created for each HLA-G protein isoform and the best model was selected according to C-score values [92, 93].

### Analyzing changes in HLA-G isoforms 3D structure due to amino acid substitution by UCSF chimera

UCSF Chimera is a program for molecular visualization, molecular structures study and related data (available at https://www.cgl.ucsf.edu/chimera/). The structures of the HLA-G isoforms predicted with I-TASSER in PDB formatted structure files were visualized by Chimera. Chimera was also used to achieve the 3D mutated models of the wild models of HLA-G isoforms with the most deleterious missense SNPs predicted in this project. The outputs are graphical models [94].

### Founding functional SNPs in UTR by the UTRscan (available at http://itbtools.ba.itb.cnr.it/utrscan)

This tool is for scrutinizing UTR functional elements throughout user-submitted sequence data for any of the patterns collected in the UTRsite and UTR databases. UTRsite is a pile of functional sequence patterns found in 5ˊ and 3ˊ UTR sequences. If two or three sequences of each particular UTR SNP are concluded to have various functional patterns, specific UTR SNP is determined to have functional significance [95].

### PolymiRTS database 3.0 (polymorphism in microRNAs and their target sites) (available at compbio.uthsc.edu/miRSNP/)

PolymiRTS is a database to analyze the 3’UTR regions of mRNAs in *Homo sapiens* and mouse for SNPs and INDELs variations in microRNA target sites. The polymorphisms of microRNA target sites may alter miRNA-mRNA interactions and accordingly gene expressions. The variations are divided into four categories according to their effect: “D” (the derived allele disrupts a conserved miRNA site), “N” (the derived allele disrupts a nonconserved miRNA site), “C” (the derived allele creates a new miRNA site) and “O” (the ancestral allele cannot be determined). “D” and “C” groups are most likely to have functional effects because they may lead to loss of normal repression and abnormal gene repression control, respectively. We submitted the HLA-G gene symbol to the program and the analysis was performed automatically on the transcript variant 2 (transcript ID: NM_002127) and functional SNPs were determined [96].

### Predicting protein-protein interactions by search tool for the retrieval of interacting proteins (STRING) (available at http://string-db.org/)

STRING is a database of protein-protein interactions. The database contains data from empirical evidences, computational prediction tools and collections of universal text. This provided availability to both experimental and theoretical interaction data of HLA-G [97, 98].

### Kaplan-Meier plotter analysis (KM plotter) (available at https://kmplot.com/analysis/)

The Kaplan Meier plotter is a tool to evaluate the impact of 54,000 genes on survival in 21 types of cancer using the microarray gene expression data. A meta-analysis based detection and validation of biomarkers for cancer patients is the primary aim of Kaplan-Meier. The ʽ211528_x_at̕ probe was used for HLA-G gene. Here, the overall survival (OS) is the period of time from the start of a change in specific gene expression (decrease or increase expression) for a cancer, that patients diagnosed with it are still alive. The expression in patients for each cancer was graded and allocated high and low expression groups according to the median level. The overall survival analysis was performed on 1402 cases of breast cancer, 1656 cases of ovarian cancer, 1926 cases of lung cancer and 876 cases of gastric cancer. These two groups of patients for cancer listed above were compared and the survival was evaluated. The *p*-values less than 0.05 were regarded as statistically significant [99–102].

## Supplementary information

**Additional file 1:**

Additional file 2: **Table 5.** Analysis of structural effects of deleterious SNPs on HLA-G1 by Project HOPE

Additional file 3: **Table 6.** Five models predicted for each human HLA-G isoform by I-TASSER

Additional file 4: **Table 7.** Structural representations of native isoforms of HLA-G predicted with I-TASSE and visualized with UCSF Chimera

Additional file 5: **Table 8.** Graphical representations of amino acid changes due to the most deleterious SNPs in isoform 1

## Data Availability

*SNPs’ information* used in present study were retrieved from NCBI dbSNP database (https://www.ncbi.nlm.nih.gov/snp/) [74]. The rsID of SNPs and their information (allele change, residue change, global minor allele frequency (MAF), and position of substitution) retrieved from NCBI dbSNP database and their corresponding IMGT/HLA alleles from https://raw.githubusercontent.com/ANHIG/IMGTHLA/Latest/ alignments/G_prot.txt) were presented in supplementary data (Table 1). The MAF of SNPs was also obtained from the dbSNP GeneView page (https://www.ncbi.nlm.nih.gov/projects/SNP/snp_ref.cgi?showRare= on&chooseRs = coding&go = Go&locusId = 3135) and is shown in supplementary Table 1. The amino acid sequences of HLA-G isoforms were achieved from the UniProt database (https://www.uniprot.org/uniprot/P17693) [75]. The tools used for prediction of the most deleterious missense nsSNPs were SIFT (https://sift.bii.a-star.edu.sg/) [76], PROVEAN (provean.jcvi.org/) [77], PolyPhen-2 (genetics.bwh.harvard.edu/pph2/) [78, 79], I-Mutant 3.0 (gpcr2.biocomp.unibo.it/cgi/predictors/I-Mutant3.0/I-Mutant3.0.cgi) [80], SNPs&GO (snps.biofold.org/snps-and-go/snps-and-go.html) [82], PhD-SNP (snps.biofold.org/phd-snp/phd-snp.html) [77, 81], SNAP2 (https://rostlab.org/services/snap/) [83–85] and MUpro (mupro.proteomics.ics.uci.edu/) [86]. ConSurf web-server (consurf.tau.ac.il/) [87, 88] estimates the evolutionary conservation of the most deleterious missense nsSNPs. The structural and functional effects of predicted SNPs were investigated with the MutPred web server (mutpred.mutdb.org/) [89, 90] and HOPE web server (www.cmbi.ru.nl/hope/) [91]. The 3D models of HLA-G protein isoforms were achieved using I-TASSER (https://zhanglab.ccmb.med.umich.edu/I-TASSER/). Chimera was used for analyzing changes in 3D structures due to amino acid substitution (https://www.cgl.ucsf.edu/chimera/) [94]. Founding functional SNPs in UTR was performed using the UTRscan (http://itbtools.ba.itb.cnr.it/utrscan) [95]. The used database for analysis of SNPs in microRNA target sites was PolymiRTS (compbio.uthsc.edu/miRSNP/) [96]. Interaction of the HLA-G protein with other proteins was investigated with the STRING database (http://string-db.org/) [97, 98]. The effect of dysregulation expression of HLA-G on survival in four types of cancer was assessed using the Kaplan Meier plotter (https://kmplot.com/analysis/) [99–102].

## Abbreviations

SNPs: Single-Nucleotide Polymorphisms
MHC: Major histocompatibility complex
HLA: Human leukocyte antigen
dNK: Decidual NK
UTR: Untranslated regions
