# Supplementary material for: Predicting the most deleterious missense nsSNPs of the protein isoforms of the human HLA-G gene and in silico evaluation of their structural and functional consequences
Source: BMC Genet. 2020 Aug 31;21:94. doi: 10.1186/s12863-020-00890-y (PMC7457528; doi:10.1186/s12863-020-00890-y)
Supplement: Supplementary file 1 — Additional file 1: [file 12863_2020_890_MOESM1_ESM.docx]

**Supplemental Data**

**Supplementary Table 1.** SNPs retrieved from NCBI and their corresponding IMGT/HLA alleles.

| **SNP rsID** | **Codons** | **Substitution** | **MAF SNP in NCBI** | **Position of substitution in isoforms** | **The most plausible IMGT/HLA alleles** | **MAF SNP: GeneView** |
| --- | --- | --- | --- | --- | --- | --- |
| rs12722477 (rs116210099 ,rs113358968 ,rs59771878 and rs17225116 have merged into rs12722477) | CTC ⇒ ATC | L [Leu] ⇒ I [Ile] | A=0.13804 (34138/247312, GnomAD_exome)  A=0.15029 (18872/125568, TOPMED)  A=0.13875 (16208/116818, ExAC)  A=0.1380 (4325/31340, GnomAD)  A=0.134 (1133/8438, GO-ESP)  A=0.203 (1015/5008, 1000G)  A=0.080 (310/3854, ALSPAC)  A=0.070 (261/3708, TWINSUK)  A=0.25 (153/611, Vietnamese)  A=0.10 (59/600, NorthernSweden) | 134 in NP_002118.1  139 in XP_016866305.1 | G*01:04:01:01 G*01:04:01:02 G*01:04:01:03 G*01:04:02 G*01:04:03 G*01:04:04 G*01:04:05 G*01:04:06 G*01:07 G*01:11 G*01:15 G*01:19 G*01:21N (Substitution: L134I) | 0.2027 |
| rs12722482 (rs142664118 has merged into rs12722482) | ACG ⇒ AAG ACG ⇒ ATG | T [Thr] ⇒ K [Lys] T [Thr] ⇒ M [Met] | T=0.04276 (5369/125568, TOPMED)  T=0.0345 (1079/31254, GnomAD)  T=0.0468 (609/13006, GO-ESP)  T=0.051 (254/5008, 1000G)  T=0.056 (216/3854, ALSPAC)  T=0.052 (191/3708, TWINSUK)  T=0.03 (17/600, NorthernSweden) | 282 in NP_002118.1 287 in XP_016866305.1 190 in XP_016866306.1 190 in XP_016866307.1 | G*01:06 G*01:14 G*01:16 ( Substitution: T282M) | 0.0507 |
| rs41545515 (rs61761353 has merged into rs41545515) | CAG ⇒ CGG | Q [Gln] ⇒ R [Arg] | None | 78 in NP_002118.1 83 in XP_016866305.1 78 in XP_016866306.1 78 in XP_016866307.1 | G*01:02 (Substitution: Q78R) |  |
| rs41551813 (rs150738496 and rs61761352 have merged into rs41551813) | ACG ⇒ TCG | T [Thr] ⇒ S [Ser] | T=0.04566 (11282/247074, GnomAD_exome)  T=0.04579 (5231/114242, ExAC)  T=0.0509 (1596/31362, GnomAD)  T=0.058 (485/8434, GO-ESP)  T=0.054 (270/5008, 1000G)  T=0.030 (117/3854, ALSPAC)  T=0.032 (120/3708, TWINSUK)  T=0.02 (14/600, NorthernSweden) | 55 in NP_002118.1 60 in XP_016866305.1 55 in XP_016866306.1 55 in XP_016866307.1 | G*01:03:01:01 G*01:03:01:02 (Substitution: T55S) | 0.0539 |
| rs41562616 (rs114676355, rs61761355 and rs52817428 have merged into rs41562616) | CAC ⇒ CAA CAC ⇒ CAT | H [His] ⇒ Q [Gln] H [His] ⇒ H [His] | T=0.04557 (11424/250700, GnomAD_exome)  T=0.05954 (7476/125568, TOPMED)  T=0.04475 (5426/121260, ExAC)  T=0.0508 (1592/31346, GnomAD)  T=0.0544 (707/13006, GO-ESP)  T=0.054 (270/5008, 1000G)  T=0.030 (116/3854, ALSPAC)  T=0.032 (119/3708, TWINSUK)  T=0.02 (14/600, NorthernSweden) | 212 in NP_002118.1 217 in XP_016866305.1 120 in XP_016866306.1 120 in XP_016866307.1 |  | 0.0539 |
| rs45530733 | CGG ⇒ TGG | R [Arg] ⇒ W [Trp] | T=0.00029 (74/251060, GnomAD_exome)  T=0.00021 (26/125568, TOPMED)  T=0.00025 (30/120862, ExAC)  T=0.0002 (6/31362, GnomAD)  T=0.001 (2/3854, ALSPAC)  T=0.001 (5/3708, TWINSUK) | 243 in NP_002118.1 248 in XP_016866305.1 151 in XP_016866306.1 151 in XP_016866307.1 | G*01:08:01 G*01:08:02 G*01:22 (Substitution: R243W) |  |
| rs55916353 (rs149745236 has merged into rs55916353) | TAC ⇒ CAC | Y [Tyr] ⇒ H [His] | C=0.00017 (43/249108, GnomAD_exome)  C=0.00015 (19/125568, TOPMED)  C=0.00013 (15/117842, ExAC)  C=0.0002 (14/78698, PAGE_STUDY)  C=0.0001 (2/31350, GnomAD)  C=0.000 (2/8440, GO-ESP) | 183 in NP_002118.1 188 in XP_016866305.1 | G*01:9 (Substitution: Y183H) |  |
| rs72558173 (rs144518615 has merged into rs72558173) | ACG ⇒ ATG | T [Thr] ⇒ M [Met] | T=0.00025 (63/247116, GnomAD_exome)  T=0.00134 (168/125568, TOPMED)  T=0.00032 (36/114280, ExAC)  T=0.0012 (38/31394, GnomAD)  T=0.002 (14/8434, GO-ESP)  T=0.001 (5/5008, 1000G) | 55 in NP_002118.1 60 in XP_016866305.1 55 in XP_016866306.1 55 in XP_016866307.1 | G*01:10 G*01:11 (Substitution: T55M) | 0.0010 |
| rs17851919 | GGC ⇒ AGC | G [Gly] ⇒ S [Ser] | None | 40 in NP_002118.1 45 in XP_016866305.1 40 in XP_016866306.1 40 in XP_016866307.1 |  |  |
| **rs17851921**§ | CAC ⇒ CCC CAC ⇒ CTC | H [His] ⇒ P [Pro]§ H [His] ⇒ L [Leu] | None | **117**§ in NP_002118.1 122 in XP_016866305.1 |  |  |
| rs17875398 (rs139312336 has merged into rs17875398) | TCC ⇒ TTC | S [Ser] ⇒ F [Phe] | T=0.00005 (11/242770, GnomAD_exome)  T=0.00025 (32/125568, TOPMED)  T=0.00005 (5/109318, ExAC)  T=0.0002 (5/31364, GnomAD)  T=0.000 (3/8408, GO-ESP)  T=0.000 (1/5008, 1000G) | 37 in NP_002118.1 42 in XP_016866305.1 37 in XP_016866306.1 37 in XP_016866307.1 | G*01:07 (Substitution: S37F) | 0.0002 |
| rs75783850 (rs775823157 has merged into rs75783850) | CAG ⇒ CAA CAG ⇒ CAT | Q [Gln] ⇒ Q [Gln] Q [Gln] ⇒ H [His] | A=0.00001 (1/117352, ExAC) | 165 in NP_002118.1 170 in XP_016866305.1 |  |  |
| rs76011433 (rs149932370 has merged into rs76011433) | GGC ⇒ GAC | G [Gly] ⇒ D [Asp] | A=0.00016 (40/246990, GnomAD_exome)  A=0.00055 (69/125568, TOPMED)  A=0.00020 (23/116544, ExAC)  A=0.0009 (74/78690, PAGE_STUDY)  A=0.0006 (19/31364, GnomAD)  A=0.000 (4/8436, GO-ESP)  A=0.001 (6/5008, 1000G) | 124 in NP_002118.1 129 in XP_016866305.1 | G*01:14 (Substitution: G124D) | 0.0012 |
| rs76333131 (rs150872094 has merged into rs76333131) | TAC ⇒ CAC | Y [Tyr] ⇒ H [His] | C=0.00013 (31/246546, GnomAD_exome)  C=0.00011 (12/113208, ExAC)  C=0.0001 (2/31362, GnomAD)  C=0.000 (2/8432, GO-ESP) | 51 in NP_002118.1 56 in XP_016866305.1 51 in XP_016866306.1 51 in XP_016866307.1 | G*01:12 (Substitution: Y51H) |  |
| rs77824819 (rs142448115 has merged into rs77824819) | GGG ⇒ GAG GGG ⇒ GTG | G [Gly] ⇒ E [Glu] G [Gly] ⇒ V [Val] | T=0.00054 (68/125568, TOPMED)  T=0.0006 (20/31376, GnomAD)  T=0.000 (3/8436, GO-ESP) | 128 in NP_002118.1 133 in XP_016866305.1 | G*01:15 (Substitution: G128V) |  |
| rs78295860 (rs548955749 has merged into rs78295860) | TCC ⇒ TGC TCC ⇒ TTC | S [Ser] ⇒ C [Cys] S [Ser] ⇒ F [Phe] | G=0.00003 (4/116590, ExAC)  G=0.000 (1/5008, 1000G) | 129 in NP_002118.1 134 in XP_016866305.1 | G*01:16 (Substitution: S129C) | 0.0002 |
| rs79178226 | CAG ⇒ GAG | Q [Gln] ⇒ E [Glu] | G=0.00001 (1/125568, TOPMED) | 78 in NP_002118.1 83 in XP_016866305.1 78 in XP_016866306.1 78 in XP_016866307.1 |  |  |
| rs79984266 (rs142406959 has merged into rs79984266) | CGG ⇒ CAG | R [Arg] ⇒ Q [Gln] | A=0.00017 (43/251152, GnomAD_exome)  A=0.00055 (69/125568, TOPMED)  A=0.00022 (26/120930, ExAC  A=0.0006 (19/31364, GnomAD)  A=0.0005 (7/13006, GO-ESP)  A=0.001 (6/5008, 1000G) | 243 in NP_002118.1 248 in XP_016866305.1 151 in XP_016866306.1 151 in XP_016866307.1 | G*01:14 (Substitution: R243Q) | 0.0012 |
| rs111233577§ | CTG ⇒ CGG | L [Leu] ⇒ R [Arg] | G=0.00001 (1/125568, TOPMED) | 290§ in NP_002118.1 295 in XP_016866305.1 198 in XP_016866306.1 198 in XP_016866307.1 |  |  |
| rs111241895 | GCC ⇒ GAC GCC ⇒ GGC | A [Ala] ⇒ D [Asp] A [Ala] ⇒ G [Gly] | None | 114 in NP_002118.1 119 in XP_016866305.1 114 in XP_016866306.1 114 in XP_016866307.1 |  |  |
| rs112800579 (rs139835805 has merged into rs112800579) | AGG ⇒ AAG | R [Arg] ⇒ K [Lys] | A=0.00000 (1/249024, GnomAD_exome) | 180 in NP_002118.1 185 in XP_016866305.1 |  |  |
| rs113311833 | GAA ⇒ GAC | E [Glu] ⇒ D [Asp] | C=0.00001 (2/248888, GnomAD_exome) | 85 in NP_002118.1 90 in XP_016866305.1 85 in XP_016866306.1 85 in XP_016866307.1 |  |  |
| rs113386443 | AGG ⇒ AGC | R [Arg] ⇒ S [Ser] | None | 180 in NP_002118.1 185 in XP_016866305.1 |  |  |
| rs113468225 | ACG ⇒ ATG | T [Thr] ⇒ M [Met] | None | 187 in NP_002118.1 192 in XP_016866305.1 |  |  |
| **rs138289952** | GAC ⇒ AAC GAC ⇒ TAC | D [Asp] ⇒ N [Asn] **D [Asp] ⇒ Y [Tyr]** | T=0.00003 (4/125568, TOPMED)  A=0.000 (1/8434, GO-ESP) | **54** in NP_002118.1 59 in XP_016866305.1 54 in XP_016866306.1 54 in XP_016866307.1 |  |  |
| rs141130133 | GTC ⇒ ATC | V [Val] ⇒ I [Ile] | A=0.00002 (4/251286, GnomAD_exome)  A=0.00002 (3/121340, ExAC)  A=0.0001 (1/13006, GO-ESP) | 317 in NP_002118.1 322 in XP_016866305.1 225 in XP_016866306.1 225 in XP_016866307.1 |  |  |
| rs141919345 | CGC ⇒ AGC | R [Arg] ⇒ S [Ser] | A=0.00001 (3/247348, GnomAD_exome)  A=0.00002 (2/116806, ExAC)  A=0.000 (1/8438, GO-ESP) | 135 in NP_002118.1 140 in XP_016866305.1 |  |  |
| **rs142596947§** | CCT ⇒ ACT | P [Pro] ⇒ T [Thr] | A=0.00004 (5/125568, TOPMED)  A=0.0001 (1/13002, GO-ESP) | **234§** in NP_002118.1 239 in XP_016866305.1 142 in XP_016866306.1 142 in XP_016866307.1 |  |  |
| rs143732275 | ATG ⇒ ACG | M [Met] ⇒ T [Thr] | C=0.00016 (40/248482, GnomAD_exome)  C=0.00013 (16/125568, TOPMED)  C=0.00021 (25/117334, ExAC)  C=0.0002 (6/31322, GnomAD)  C=0.000 (1/8440, GO-ESP)  C=0.000 (1/3854, ALSPAC)  C=0.000 (0/3708, TWINSUK) | 1 in NP_002118.1  6 in XP_016866305.1  1 in XP_016866306.1  1 in XP_016866307.1 |  |  |
| **rs144577485§** | CCC ⇒ GCC | P [Pro] ⇒ A [Ala] | G=0.00002 (5/250582, GnomAD_exome)  G=0.00004 (5/125568, TOPMED)  G=0.0001 (1/13006, GO-ESP) | **209§** in NP_002118.1  214 in XP_016866305.1  117 in XP_016866306.1  117 in XP_016866307.1 | G*01:18 (Substitution: P209A) |  |
| **rs145097667§** | CAT ⇒ TAT | H [His] ⇒ Y [Tyr] | T=0.00004 (5/125568, TOPMED)  T=0.0002 (2/13006, GO-ESP) | **287§** in NP_002118.1  292 in XP_016866305.1  195 in XP_016866306.1  195 in XP_016866307.1 |  |  |
| rs146376471 | GGG ⇒ GAG | G [Gly] ⇒ E [Glu] | A=0.00000 (1/248040, GnomAD_exome)  A=0.00001 (1/116320, ExAC)  A=0.000 (1/8440, GO-ESP) | 15 in NP_002118.1  20 in XP_016866305.1  15 in XP_016866306.1  15 in XP_016866307.1 |  |  |
| rs147620137 | GCG ⇒ GTG | A [Ala] ⇒ V [Val] | T=0.00010 (26/251152, GnomAD_exome)  T=0.00047 (59/125568, TOPMED)  T=0.00013 (16/121110, ExAC)  T=0.0003 (8/31344, GnomAD)  T=0.0003 (4/13000, GO-ESP) | 235 in NP_002118.1  240 in XP_016866305.1  143 in XP_016866306.1  143 in XP_016866307.1 |  |  |
| rs148951074 | AGG ⇒ AGT | R [Arg] ⇒ S [Ser] | T=0.00002 (4/248502, GnomAD_exome)  T=0.00004 (5/125568, TOPMED)  T=0.00003 (3/117338, ExAC)  T=0.0001 (4/31330, GnomAD)  T=0.000 (1/8438, GO-ESP)  T=0.000 (1/5008, 1000G) | 5 in XP_016866305.1 |  |  |
| rs150671463 | GAA ⇒ GAC GAA ⇒ GAG | E [Glu] ⇒ D [Asp] E [Glu] ⇒ E [Glu] | G=0.00005 (12/247480, GnomAD_exome)  G=0.00002 (3/125568, TOPMED)  G=0.00004 (5/116904, ExAC)  G=0.0001 (2/31368, GnomAD)  G=0.000 (1/8438, GO-ESP) | 138 in NP_002118.1 143 in XP_016866305.1 |  |  |
| rs200094914 | GGG ⇒ GAG | G [Gly] ⇒ E [Glu] | None | 80 in NP_002118.1  85 in XP_016866305.1  80 in XP_016866306.1  80 in XP_016866307.1 |  |  |
| rs200213525 | TTT ⇒ TCT | F [Phe] ⇒ S [Ser] | None | 219 in NP_002118.1  224 in XP_016866305.1  127 in XP_016866306.1  127 in XP_016866307.1 |  |  |
| rs200575956 | ATT ⇒ TTT | I [Ile] ⇒ F [Phe] | T=0.00002 (5/246962, GnomAD_exome)  T=0.00001 (1/125568, TOPMED)  T=0.00003 (3/116534, ExAC)  T=0.000 (1/8438, GO-ESP)  T=0.000 (1/5008, 1000G) | 123 in NP_002118.1  128 in XP_016866305.1 |  | 0.0002 |
| rs200909460 | CGC ⇒ CAC CGC ⇒ CCC CGC ⇒ CTC | R [Arg] ⇒ H [His] R [Arg] ⇒ P [Pro] R [Arg] ⇒ L [Leu] | C=0.00001 (3/248480, GnomAD_exome)  C=0.00004 (5/125568, TOPMED)  C=0.00002 (2/117390, ExAC) | 169 in NP_002118.1 174 in XP_016866305.1 |  |  |
| rs200931762 | GCA ⇒ ACA | A [Ala] ⇒ T [Thr] | A=0.00004 (10/250842, GnomAD_exome)  A=0.00001 (1/125568, TOPMED)  A=0.00002 (3/120194, ExAC)  A=0.0000 (1/31356, GnomAD)  A=0.000 (2/5008, 1000G)  A=0.00 (1/214, Vietnamese) | 269 in NP_002118.1  274 in XP_016866305.1  177 in XP_016866306.1  177 in XP_016866307.1 |  | 0.0004 |
| rs201957061 | GCC ⇒ ACC GCC ⇒ CCC | A [Ala] ⇒ T [Thr] A [Ala] ⇒ P [Pro] | C=0.00000 (1/241920, GnomAD_exome) | 34 in NP_002118.1  39 in XP_016866305.1  34 in XP_016866306.1  34 in XP_016866307.1 |  |  |
| rs202126599 | ACC ⇒ AAC | T [Thr] ⇒ N [Asn] | A=0.00001 (2/250900, GnomAD_exome)  A=0.00002 (2/120276, ExAC)  A=0.000 (1/5008, 1000G) | 264 in NP_002118.1  269 in XP_016866305.1  172 in XP_016866306.1  172 in XP_016866307.1 |  | 0.0002 |
| rs370197833 | AAT ⇒ AGT AAT ⇒ ATT | N [Asn] ⇒ S [Ser] N [Asn] ⇒ I [Ile] | G=0.00001 (3/248792, GnomAD_exome)  G=0.00001 (1/125568, TOPMED)  T=0.00001 (1/125568, TOPMED)  G=0.00002 (2/117574, ExAC)  G=0.0000 (1/31380, GnomAD)  G=0.000 (1/8440, GO-ESP) | 175 in NP_002118.1 180 in XP_016866305.1 |  |  |
| rs373168953 | GGC ⇒ AGC | G [Gly] ⇒ S [Ser] | A=0.00002 (5/248304, GnomAD_exome)  A=0.00010 (12/125568, TOPMED)  A=0.00003 (3/117360, ExAC)  A=0.0001 (4/78702, PAGE_STUDY)  A=0.000 (1/5008, 1000G) | 107 in NP_002118.1  112 in XP_016866305.1  107 in XP_016866306.1  107 in XP_016866307.1 |  | 0.0002 |
| rs373197901 | GAA ⇒ CAA | E [Glu] ⇒ Q [Gln] | C=0.00002 (5/248884, GnomAD_exome)  C=0.00008 (10/125568, TOPMED)  C=0.00001 (1/118052, ExAC)  C=0.0001 (3/31372, GnomAD)  C=0.000 (2/8440, GO-ESP) | 85 in NP_002118.1  90 in XP_016866305.1  85 in XP_016866306.1  85 in XP_016866307.1 |  |  |
| rs375611189 | AAG ⇒ GAG | K [Lys] ⇒ E [Glu] | G=0.00016 (39/248542, GnomAD_exome)  G=0.00003 (4/125568, TOPMED)  G=0.00016 (19/117422, ExAC)  G=0.001 (6/5008, 1000G)  G=0.00 (3/612, Vietnamese) | 170 in NP_002118.1 175 in XP_016866305.1 |  | 0.0012 |
| rs536339156 | TCC ⇒ CCC | S [Ser] ⇒ P [Pro] | C=0.00000 (1/248020, GnomAD_exome)  C=0.00001 (1/117150, ExAC)  C=0.000 (1/5008, 1000G) | 156 in NP_002118.1 161 in XP_016866305.1 |  | 0.0002 |
| rs536613482 | GCC ⇒ ACC GCC ⇒ CCC GCC ⇒ TCC | A [Ala] ⇒ T [Thr] A [Ala] ⇒ P [Pro] A [Ala] ⇒ S [Ser] | C=0.0001 (2/31328, GnomAD)  T=0.000 (1/5008, 1000G) | 149 in NP_002118.1 154 in XP_016866305.1 |  | 0.0002 |
| rs537772641 | ATG ⇒ ATA ATG ⇒ ATC | M [Met] ⇒ I [Ile] | A=0.00007 (9/125568, TOPMED)  C=0.00029 (35/120398, ExAC)  C=0.001 (4/5008, 1000G) | 295 in NP_002118.1  300 in XP_016866305.1  203 in XP_016866306.1  203 in XP_016866307.1 |  | 0.0008 |
| **rs540632198** | TTC ⇒ TGC | F [Phe] ⇒ C [Cys] | G=0.00000 (1/241932, GnomAD_exome) G=0.000 (1/5008, 1000G) | **32** in NP_002118.1  37 in XP_016866305.1  32 in XP_016866306.1  32 in XP_016866307.1 |  | 0.0002 |
| rs541772318 | GGC ⇒ GAC | G [Gly] ⇒ D [Asp] | A=0.00000 (1/249240, GnomAD_exome)  A=0.00001 (1/117970, ExAC)  A=0.000 (1/5008, 1000G) | 186 in NP_002118.1 191 in XP_016866305.1 |  | 0.0002 |
| rs545946708 | GGG ⇒ AGG | G [Gly] ⇒ R [Arg] | A=0.00007 (18/247952, GnomAD_exome)  A=0.00007 (8/116342, ExAC)  A=0.001 (4/5008, 1000G) | 15 in NP_002118.1  20 in XP_016866305.1  15 in XP_016866306.1  15 in XP_016866307.1 |  | 0.0008 |
| rs551240433 | CGC ⇒ GGC CGC ⇒ TGC | R [Arg] ⇒ G [Gly] R [Arg] ⇒ C [Cys] | G=0.00012 (15/125568, TOPMED)  G=0.00002 (2/116692, ExAC)  G=0.000 (1/5008, 1000G) | 132 in NP_002118.1 137 in XP_016866305.1 |  | 0.0002 |
| rs553193145 | ATG ⇒ GTG | M [Met] ⇒ V [Val] | G=0.00000 (1/251220, GnomAD_exome)  G=0.00001 (1/121102, ExAC)  G=0.000 (1/5008, 1000G) | 309 in NP_002118.1  314 in XP_016866305.1  217 in XP_016866306.1  217 in XP_016866307.1 |  | 0.0002 |
| **rs555347515§** | ATG ⇒ AAG | M [Met] ⇒ K [Lys] | A=0.00002 (3/125568, TOPMED)  A=0.000 (1/5008, 1000G) | **29§** in NP_002118.1  34 in XP_016866305.1  29 in XP_016866306.1  29 in XP_016866307.1 |  | 0.0002 |
| **rs556645753§** | GAC ⇒ GGC | D [Asp] ⇒ G [Gly] | G=0.00000 (1/247946, GnomAD_exome)  G=0.00001 (1/117138, ExAC)  G=0.000 (1/5008, 1000G) | **153§** in NP_002118.1 158 in XP_016866305.1 |  | 0.0002 |
| rs556746961 | AGC ⇒ ACC | S [Ser] ⇒ T [Thr] | C=0.00004 (9/249408, GnomAD_exome)  C=0.00006 (8/125568, TOPMED)  C=0.00002 (2/119462, ExAC)  C=0.0001 (3/31350, GnomAD) | 336 in NP_002118.1  341 in XP_016866305.1  244 in XP_016866306.1  244 in XP_016866307.1 |  |  |
| rs562210158 | AGC ⇒ AGA | S [Ser] ⇒ R [Arg] | A=0.00000 (1/247458, GnomAD_exome)  A=0.00001 (1/115176, ExAC)  A=0.000 (1/5008, 1000G) | 62 in NP_002118.1  67 in XP_016866305.1  62 in XP_016866306.1  62 in XP_016866307.1 |  | 0.0002 |
| rs562739922 | GCC ⇒ TCC | A [Ala] ⇒ S [Ser] | T=0.00026 (64/247740, GnomAD_exome)  T=0.00022 (28/125568, TOPMED)  T=0.00022 (25/116188, ExAC)  T=0.0001 (2/31342, GnomAD)  T=0.000 (2/5008, 1000G) | 16 in NP_002118.1  21 in XP_016866305.1  16 in XP_016866306.1  16 in XP_016866307.1 |  | 0.0004 |
| **rs565858069§** | GAC ⇒ AAC GAC ⇒ CAC | D [Asp] ⇒ N [Asn] **D [Asp] ⇒ H [His]** | C=0.00009 (11/125568, TOPMED)  C=0.0000 (1/31366, GnomAD)  C=0.000 (2/5008, 1000G) | **130§** in NP_002118.1 135 in XP_016866305.1 |  | 0.0004 |
| rs571857419 | ATG ⇒ ACG | M [Met] ⇒ T [Thr] | C=0.00003 (8/249246, GnomAD_exome)  C=0.00028 (34/120292, ExAC)  C=0.001 (4/5008, 1000G) | 295 in NP_002118.1  300 in XP_016866305.1  203 in XP_016866306.1  203 in XP_016866307.1 |  | 0.0008 |
| **rs572025435§** | AGG ⇒ AGT | R [Arg] ⇒ S [Ser] | T=0.00001 (1/107928, ExAC) T=0.000 (1/5008, 1000G) | **30§** in NP_002118.1  35 in XP_016866305.1  30 in XP_016866306.1  30 in XP_016866307.1 |  | 0.0002 |
| rs573608167 | CGC ⇒ CAC | R [Arg] ⇒ H [His] | A=0.00001 (2/247988, GnomAD_exome)  A=0.00001 (1/117142, ExAC)  A=0.000 (1/5008, 1000G) | 155 in NP_002118.1 160 in XP_016866305.1 |  | 0.0002 |
| rs745630585 | GTT ⇒ ATT GTT ⇒ CTT | V [Val] ⇒ I [Ile] V [Val] ⇒ L [Leu] | A=0.00001 (1/125568, TOPMED)  A=0.00001 (1/121190, ExAC) | 312 in NP_002118.1  317 in XP_016866305.1  220 in XP_016866306.1  220 in XP_016866307.1 |  |  |
| rs746249960 | GAC ⇒ GGC | D [Asp] ⇒ G [Gly] | G=0.00000 (1/248938, GnomAD_exome) G=0.00001 (1/117928, ExAC) | 98 in NP_002118.1  103 in XP_016866305.1  98 in XP_016866306.1  98 in XP_016866307.1 |  |  |
| rs746923096 | GAG ⇒ GGG | E [Glu] ⇒ G [Gly] | G=0.00000 (1/249210, GnomAD_exome) G=0.00001 (1/117936, ExAC) | 185 in NP_002118.1 190 in XP_016866305.1 |  |  |
| rs747314068 | GCT ⇒ ACT | A [Ala] ⇒ T [Thr] | A=0.00002 (5/251052, GnomAD_exome)  A=0.00002 (3/121314, ExAC)  A=0.000 (0/3854, ALSPAC)  A=0.000 (1/3708, TWINSUK) | 328 in NP_002118.1  333 in XP_016866305.1  236 in XP_016866306.1  236 in XP_016866307.1 |  |  |
| rs747437773 | ACC ⇒ GCC | T [Thr] ⇒ A [Ala] | G=0.00000 (1/251128, GnomAD_exome)  G=0.00001 (1/120576, ExAC) | 257 in NP_002118.1  262 in XP_016866305.1  165 in XP_016866306.1  165 in XP_016866307.1 |  |  |
| **rs748013931§** | ACC ⇒ CCC | T [Thr] ⇒ P [Pro] | C=0.00000 (1/248046, GnomAD_exome) C=0.00001 (1/117156, ExAC) | **158§** in NP_002118.1 163 in XP_016866305.1 |  |  |
| rs748519460 | ACC ⇒ TCC | T [Thr] ⇒ S [Ser] | T=0.00000 (1/250810, GnomAD_exome) T=0.00001 (1/121298, ExAC) | 214 in NP_002118.1  219 in XP_016866305.1  122 in XP_016866306.1  122 in XP_016866307.1 |  |  |
| rs748765033 | TTC ⇒ CTC | F [Phe] ⇒ L [Leu] | C=0.00000 (1/241930, GnomAD_exome)  C=0.00001 (1/108256, ExAC) | 32 in NP_002118.1  37 in XP_016866305.1  32 in XP_016866306.1  32 in XP_016866307.1 |  |  |
| **rs749006959§** | TAT ⇒ TGT | Y [Tyr] ⇒ C [Cys] | G=0.00000 (1/247408, GnomAD_exome) G=0.00001 (1/116874, ExAC) | 137 in NP_002118.1 **142§** in XP_016866305.1 |  |  |
| rs749449002 | CAG ⇒ CGG | Q [Gln] ⇒ R [Arg] | G=0.00003 (7/250830, GnomAD_exome) G=0.00002 (3/120472, ExAC) | 286 in NP_002118.1  291 in XP_016866305.1  194 in XP_016866306.1  194 in XP_016866307.1 |  |  |
| rs749634426 | CAC ⇒ TAC | H [His] ⇒ Y [Tyr] | T=0.00000 (1/249438, GnomAD_exome)  T=0.00001 (1/118198, ExAC) | 193 in NP_002118.1 198 in XP_016866305.1 |  |  |
| **rs750238738§** | ATC ⇒ TTC | I [Ile] ⇒ F [Phe] | T=0.00009 (23/251170, GnomAD_exome)  T=0.00001 (1/125568, TOPMED)  T=0.00011 (13/121088, ExAC) | **237§** in NP_002118.1  242 in XP_016866305.1  145 in XP_016866306.1  145 in XP_016866307.1 |  |  |
| rs751127247 | CAC ⇒ TAC | H [His] ⇒ Y [Tyr] | T=0.00000 (1/248920, GnomAD_exome) T=0.00001 (1/117976, ExAC) | 94 in NP_002118.1  99 in XP_016866305.1  94 in XP_016866306.1  94 in XP_016866307.1 |  |  |
| rs751954365 | CTC ⇒ TTC | L [Leu] ⇒ F [Phe] | T=0.00006 (15/248274, GnomAD_exome)  T=0.00009 (10/116616, ExAC)  T=0.0000 (1/31358, GnomAD) | 13 in NP_002118.1  18 in XP_016866305.1  13 in XP_016866306.1  13 in XP_016866307.1 |  |  |
| rs752196294 | AAG ⇒ ATG | K [Lys] ⇒ M [Met] | T=0.00000 (1/248996, GnomAD_exome)  T=0.00001 (1/118036, ExAC) | 92 in NP_002118.1  97 in XP_016866305.1  92 in XP_016866306.1  92 in XP_016866307.1 |  |  |
| rs752509370 | GCC ⇒ GTC | A [Ala] ⇒ V [Val] | T=0.00003 (7/249074, GnomAD_exome) T=0.00003 (4/117808, ExAC) | 182 in NP_002118.1 187 in XP_016866305.1 |  |  |
| rs753323622 | AAG ⇒ AAT | K [Lys] ⇒ N [Asn] | None | 267 in NP_002118.1  272 in XP_016866305.1  175 in XP_016866306.1  175 in XP_016866307.1 |  |  |
| rs753402473 | CCC ⇒ ACC CCC ⇒ GCC CCC ⇒ TCC | P [Pro] ⇒ T [Thr] P [Pro] ⇒ A [Ala] P [Pro] ⇒ S [Ser] | G=0.00000 (1/249762, GnomAD_exome)  A=0.00001 (1/125568, TOPMED)  T=0.00001 (1/125568, TOPMED)  A=0.00001 (1/121008, ExAC) | 208 in NP_002118.1  213 in XP_016866305.1  116 in XP_016866306.1  116 in XP_016866307.1 |  |  |
| rs753634137 | CGG ⇒ AGG CGG ⇒ GGG CGG ⇒ TGG | R [Arg] ⇒ R [Arg] R [Arg] ⇒ G [Gly] R [Arg] ⇒ W [Trp] | None | 89 in NP_002118.1  94 in XP_016866305.1  89 in XP_016866306.1  89 in XP_016866307.1 |  |  |
| rs754527717§ | GAT ⇒ GGT | D [Asp] ⇒ G [Gly] | G=0.00002 (4/251200, GnomAD_exome)  G=0.00006 (8/125568, TOPMED)  G=0.00002 (3/120944, ExAC) | 244§ in NP_002118.1  249 in XP_016866305.1  152 in XP_016866306.1  152 in XP_016866307.1 |  |  |
| rs755422730 | AGT ⇒ AGG | S [Ser] ⇒ R [Arg] | G=0.00000 (1/245780, GnomAD_exome)  G=0.00001 (1/115736, ExAC) | 115 in NP_002118.1 120 in XP_016866305.1 |  |  |
| rs756079304 | GAC ⇒ GAA GAC ⇒ GAG GAC ⇒ GAT | D [Asp] ⇒ E [Glu] D [Asp] ⇒ E [Glu] D [Asp] ⇒ D [Asp] | None | 207 in NP_002118.1  212 in XP_016866305.1  115 in XP_016866306.1  115 in XP_016866307.1 |  |  |
| rs756652306§ | GTG ⇒ GCG | V [Val] ⇒ A [Ala] | C=0.00007 (17/250918, GnomAD_exome)  C=0.00011 (14/125568, TOPMED)  C=0.00010 (12/120468, ExAC)  C=0.0000 (1/31304, GnomAD) | 285§ in NP_002118.1  290 in XP_016866305.1  193 in XP_016866306.1  193 in XP_016866307.1 |  |  |
| rs756969252 | CAG ⇒ CAT | Q [Gln] ⇒ H [His] | T=0.00000 (1/248982, GnomAD_exome)  T=0.00001 (1/117998, ExAC) | 96 in NP_002118.1  101 in XP_016866305.1  96 in XP_016866306.1  96 in XP_016866307.1 |  |  |
| rs757457041 | GGG ⇒ CGG | G [Gly] ⇒ R [Arg] | C=0.00001 (1/116832, ExAC) | 136 in NP_002118.1 141 in XP_016866305.1 |  |  |
| rs757883678 | GCC ⇒ CCC | A [Ala] ⇒ P [Pro] | C=0.00001 (1/125568, TOPMED)  C=0.00001 (1/118000, ExAC) | 93 in NP_002118.1  98 in XP_016866305.1  93 in XP_016866306.1  93 in XP_016866307.1 |  |  |
| rs758072301 | GAG ⇒ GGG | E [Glu] ⇒ G [Gly] | G=0.00000 (1/251216, GnomAD_exome)  G=0.00001 (1/120726, ExAC) | 253 in NP_002118.1  258 in XP_016866305.1  161 in XP_016866306.1  161 in XP_016866307.1 |  |  |
| rs758520607 | CTC ⇒ TTC | L [Leu] ⇒ F [Phe] | T=0.00001 (1/116432, ExAC) | 119 in NP_002118.1 124 in XP_016866305.1 |  |  |
| rs758548929 | ATC ⇒ AAC | I [Ile] ⇒ N [Asn] | A=0.00000 (1/251206, GnomAD_exome)  A=0.00001 (1/121070, ExAC)  A=0.000 (0/3854, ALSPAC)  A=0.000 (1/3708, TWINSUK) | 308 in NP_002118.1  313 in XP_016866305.1  216 in XP_016866306.1  216 in XP_016866307.1 |  |  |
| rs758613787 | ATC ⇒ GTC | I [Ile] ⇒ V [Val] | G=0.00000 (1/251272, GnomAD_exome)  G=0.00001 (1/121164, ExAC) | 311 in NP_002118.1  316 in XP_016866305.1  219 in XP_016866306.1  219 in XP_016866307.1 |  |  |
| rs759164167 | GCT ⇒ CCT | A [Ala] ⇒ P [Pro] | C=0.00001 (1/117298, ExAC) | 164 in NP_002118.1 169 in XP_016866305.1 |  |  |
| rs759390427 | GAG ⇒ GAA GAG ⇒ GAC | E [Glu] ⇒ E [Glu] E [Glu] ⇒ D [Asp] | A=0.00001 (1/125568, TOPMED) | 113 in NP_002118.1  118 in XP_016866305.1  113 in XP_016866306.1  113 in XP_016866307.1 |  |  |
| rs759548979 | GCT ⇒ GTT | A [Ala] ⇒ V [Val] | T=0.00001 (3/250846, GnomAD_exome)  T=0.00001 (1/120182, ExAC) | 270 in NP_002118.1  275 in XP_016866305.1  178 in XP_016866306.1  178 in XP_016866307.1 |  |  |
| rs759670895 | CCC ⇒ CAC | P [Pro] ⇒ H [His] | A=0.00000 (1/244790, GnomAD_exome)  A=0.00001 (1/111306, ExAC) | 44 in NP_002118.1  49 in XP_016866305.1  44 in XP_016866306.1  44 in XP_016866307.1 |  |  |
| **rs760500349§** | CAG ⇒ CGG CAG ⇒ CTG | Q [Gln] ⇒ R [Arg] Q [Gln] ⇒ L [Leu] | T=0.00001 (2/250788, GnomAD_exome)  G=0.00002 (2/125568, TOPMED)  T=0.00002 (2/120196, ExAC) | **266§** in NP_002118.1  271 in XP_016866305.1  174 in XP_016866306.1  174 in XP_016866307.1 |  |  |
| rs760705156 | ATG ⇒ ACG | M [Met] ⇒ T [Thr] | C=0.00001 (3/249726, GnomAD_exome)  C=0.00002 (2/118376, ExAC) | 202 in NP_002118.1 207 in XP_016866305.1 | G*01:20 (Substitution: M202T) |  |
| rs761148652 | AAG ⇒ AAT | K [Lys] ⇒ N [Asn] | T=0.00001 (2/250098, GnomAD_exome)  T=0.00002 (2/120344, ExAC) | 299 in NP_002118.1  304 in XP_016866305.1  207 in XP_016866306.1  207 in XP_016866307.1 |  |  |
| rs761874800 | GAG ⇒ AAG GAG ⇒ TAG | E [Glu] ⇒ K [Lys] E [Glu] ⇒ Ter[*] [AMB] | None | 113 in NP_002118.1  118 in XP_016866305.1  113 in XP_016866306.1  113 in XP_016866307.1 |  |  |
| rs762321434 | TAC ⇒ CAC | Y [Tyr] ⇒ H [His] | C=0.00001 (3/249442, GnomAD_exome)  C=0.00001 (1/125568, TOPMED)  C=0.00002 (2/118288, ExAC)  C=0.0000 (1/31360, GnomAD) | 195 in NP_002118.1 200 in XP_016866305.1 | G*01:17 (Substitution: Y195H) |  |
| rs763022114 | GAT ⇒ AAT | D [Asp] ⇒ N [Asn] | A=0.00000 (1/250980, GnomAD_exome)  A=0.00001 (1/120366, ExAC) | 262 in NP_002118.1  267 in XP_016866305.1  170 in XP_016866306.1  170 in XP_016866307.1 |  |  |
| **rs763201540§** | GAC ⇒ AAC GAC ⇒ TAC | D [Asp] ⇒ N [Asn] D [Asp] ⇒ Y [Tyr] | T=0.00002 (2/113622, ExAC) | **53§** in NP_002118.1  58 in XP_016866305.1  53 in XP_016866306.1  53 in XP_016866307.1 |  |  |
| rs763875615 | GAG ⇒ AAG | E [Glu] ⇒ K [Lys] | A=0.00002 (4/251010, GnomAD_exome)  A=0.00001 (1/125568, TOPMED)  A=0.00001 (1/120464, ExAC)  A=0.0000 (1/31326, GnomAD) | 278 in NP_002118.1  283 in XP_016866305.1  186 in XP_016866306.1  186 in XP_016866307.1 |  |  |
| rs764558097 | CGC ⇒ AGC | R [Arg] ⇒ S [Ser] | A=0.00000 (1/248470, GnomAD_exome)  A=0.00001 (1/117388, ExAC) | 169 in NP_002118.1 174 in XP_016866305.1 |  |  |
| rs765213288 | CGG ⇒ CCG | R [Arg] ⇒ P [Pro] | C=0.00002 (4/248960, GnomAD_exome)  C=0.00001 (1/117984, ExAC) | 89 in NP_002118.1  94 in XP_016866305.1  89 in XP_016866306.1  89 in XP_016866307.1 |  |  |
| **rs765275727§** | TGG ⇒ CGG | W [Trp] ⇒ R [Arg] | C=0.00001 (2/249990, GnomAD_exome)  C=0.00001 (1/120830, ExAC) | **298§** in NP_002118.1  303 in XP_016866305.1  206 in XP_016866306.1  206 in XP_016866307.1 |  |  |
| rs765362190 | CGC ⇒ TGC | R [Arg] ⇒ C [Cys] | T=0.00003 (8/244972, GnomAD_exome)  T=0.00006 (7/125568, TOPMED)  T=0.00004 (5/111488, ExAC)  T=0.0000 (1/31370, GnomAD) | 45 in NP_002118.1  50 in XP_016866305.1  45 in XP_016866306.1  45 in XP_016866307.1 |  |  |
| rs765798458 | GAT ⇒ AAT | D [Asp] ⇒ N [Asn] | A=0.00001 (2/248188, GnomAD_exome)  A=0.00001 (1/125568, TOPMED)  A=0.00001 (1/118930, ExAC) | 338 in NP_002118.1  343 in XP_016866305.1  246 in XP_016866306.1  246 in XP_016866307.1 |  |  |
| rs766121990 | GAG ⇒ AAG | E [Glu] ⇒ K [Lys] | A=0.00000 (1/248892, GnomAD_exome)  A=0.00001 (1/118046, ExAC) | 86 in NP_002118.1  91 in XP_016866305.1  86 in XP_016866306.1  86 in XP_016866307.1 |  |  |
| rs766671384 | CAG ⇒ CCG CAG ⇒ CTG | Q [Gln] ⇒ P [Pro] Q [Gln] ⇒ L [Leu] | C=0.00001 (3/250270, GnomAD_exome)  T=0.00001 (1/125568, TOPMED)  C=0.00001 (1/120400, ExAC)  C=0.0000 (1/31340, GnomAD) | 300 in NP_002118.1  305 in XP_016866305.1  208 in XP_016866306.1  208 in XP_016866307.1 |  |  |
| rs767147746 | GAG ⇒ CAG GAG ⇒ TAG | E [Glu] ⇒ Q [Gln] E [Glu] ⇒ Ter[*] [AMB] | T=0.00003 (4/125568, TOPMED)  C=0.000 (0/3854, ALSPAC)  C=0.000 (1/3708, TWINSUK) | 152 in NP_002118.1 157 in XP_016866305.1 |  |  |
| rs767564873 | TGT ⇒ CGT | C [Cys] ⇒ R [Arg] | C=0.00001 (2/248554, GnomAD_exome)  C=0.00001 (1/117444, ExAC) | 171 in NP_002118.1 176 in XP_016866305.1 |  |  |
| rs767964419 | AAC ⇒ AGC | N [Asn] ⇒ S [Ser] | G=0.00000 (1/249696, GnomAD_exome)  G=0.00001 (1/118378, ExAC) | 198 in NP_002118.1 203 in XP_016866305.1 |  |  |
| rs768069291 | TCG ⇒ TGG | S [Ser] ⇒ W [Trp] | G=0.00000 (1/247980, GnomAD_exome)  G=0.00001 (1/116394, ExAC) | 14 in NP_002118.1  19 in XP_016866305.1  14 in XP_016866306.1  14 in XP_016866307.1 |  |  |
| rs768731691 | GCG ⇒ GAG GCG ⇒ GTG | A [Ala] ⇒ E [Glu] A [Ala] ⇒ V [Val] | A=0.00002 (3/121328, ExAC)  T=0.0000 (1/31352, GnomAD) | 326 in NP_002118.1  331 in XP_016866305.1  234 in XP_016866306.1  234 in XP_016866307.1 |  |  |
| rs768963082 | CAC ⇒ CCC CAC ⇒ CGC | H [His] ⇒ P [Pro] H [His] ⇒ R [Arg] | G=0.00002 (5/249168, GnomAD_exome)  C=0.00001 (1/125568, TOPMED)  G=0.00003 (4/117984, ExAC)  G=0.0000 (1/31376, GnomAD | 193 in NP_002118.1 198 in XP_016866305.1 | G*01:17 (Substitution: H193R) |  |
| rs769348329 | CAG ⇒ AAG | Q [Gln] ⇒ K [Lys] | A=0.00001 (2/248370, GnomAD_exome)  A=0.00002 (2/117330, ExAC) | 165 in NP_002118.1 170 in XP_016866305.1 |  |  |
| rs769433040 | GAG ⇒ GCG | E [Glu] ⇒ A [Ala] | C=0.00008 (19/249400, GnomAD_exome)  C=0.00008 (9/118130, ExAC)  C=0.0000 (1/31372, GnomAD) | 190 in NP_002118.1 195 in XP_016866305.1 |  |  |
| **rs770027530§** | TGC ⇒ TAC TGC ⇒ TTC | C [Cys] ⇒ Y [Tyr] C [Cys] ⇒ F [Phe] | A=0.00002 (3/121294, ExAC) | **227§** in NP_002118.1  232 in XP_016866305.1  135 in XP_016866306.1  135 in XP_016866307.1 |  |  |
| **rs770412396§** | CTG ⇒ CCG | L [Leu] ⇒ P [Pro] | C=0.00001 (2/248650, GnomAD_exome)  C=0.00002 (2/117638, ExAC) | **102§** in NP_002118.1  107 in XP_016866305.1  102 in XP_016866306.1  102 in XP_016866307.1 | G*01:22 (Substitution: L105P) |  |
| rs770423861 | GCG ⇒ TCG | A [Ala] ⇒ S [Ser] | T=0.00000 (1/248250, GnomAD_exome)  T=0.00001 (1/117288, ExAC) | 163 in NP_002118.1 168 in XP_016866305.1 |  |  |
| rs770992838 | GTC ⇒ GCC | V [Val] ⇒ A [Ala] | C=0.00000 (1/248520, GnomAD_exome)  C=0.00001 (1/117394, ExAC) | 3 in NP_002118.1  8 in XP_016866305.1  3 in XP_016866306.1  3 in XP_016866307.1 |  |  |
| rs771561178 | ACC ⇒ AGC | T [Thr] ⇒ S [Ser] | G=0.00000 (1/251094, GnomAD_exome)  G=0.00001 (1/120538, ExAC) | 257 in NP_002118.1  262 in XP_016866305.1  165 in XP_016866306.1  165 in XP_016866307.1 |  |  |
| rs771577119 | GTC ⇒ ATC GTC ⇒ CTC | V [Val] ⇒ I [Ile] V [Val] ⇒ L [Leu] | C=0.00000 (1/250970, GnomAD_exome)  C=0.00001 (1/121344, ExAC) | 218 in NP_002118.1  223 in XP_016866305.1  126 in XP_016866306.1  126 in XP_016866307.1 |  |  |
| rs771950713 | CCA ⇒ ACA | P [Pro] ⇒ T [Thr] | A=0.00000 (1/248376, GnomAD_exome)  A=0.00001 (1/117284, ExAC) | 4 in XP_016866305.1 |  |  |
| **rs772834879§** | TAC ⇒ CAC | Y [Tyr] ⇒ H [His] | C=0.00001 (3/247594, GnomAD_exome)  C=0.00001 (1/116958, ExAC) | **142§** in NP_002118.1 147 in XP_016866305.1 |  |  |
| rs772870109 | TGG ⇒ AGG | W [Trp] ⇒ R [Arg] | A=0.00002 (4/250434, GnomAD_exome)  A=0.00001 (1/121292, ExAC) | 332 in NP_002118.1  337 in XP_016866305.1  240 in XP_016866306.1  240 in XP_016866307.1 |  |  |
| rs773398196 | GTG ⇒ ATG | V [Val] ⇒ M [Met] | A=0.00001 (1/125568, TOPMED)  A=0.00022 (26/120066, ExAC) | 272 in NP_002118.1  277 in XP_016866305.1  180 in XP_016866306.1  180 in XP_016866307.1 |  |  |
| rs774388746 | TCC ⇒ CCC | S [Ser] ⇒ P [Pro] | C=0.00001 (1/116580, ExAC) | 129 in NP_002118.1 134 in XP_016866305.1 |  |  |
| rs774859148 | AAG ⇒ GAG | K [Lys] ⇒ E [Glu] | G=0.00011 (13/120692, ExAC) | 299 in NP_002118.1  304 in XP_016866305.1  207 in XP_016866306.1  207 in XP_016866307.1 | G*01:22 (Substitution: K299E) |  |
| rs775093980 | GGG ⇒ AGG | G [Gly] ⇒ R [Arg] | A=0.00000 (1/247124, GnomAD_exome)  A=0.00001 (1/116562, ExAC) | 128 in NP_002118.1 133 in XP_016866305.1 |  |  |
| rs775251045 | ACC ⇒ GCC | T [Thr] ⇒ A [Ala] | G=0.00000 (1/248288, GnomAD_exome)  G=0.00001 (1/117758, ExAC) | 8 in NP_002118.1  13 in XP_016866305.1  8 in XP_016866306.1  8 in XP_016866307.1 |  |  |
| rs775624015 | TAC ⇒ TGC | Y [Tyr] ⇒ C [Cys] | G=0.00000 (1/251166, GnomAD_exome)  G=0.00001 (1/121184, ExAC) | 233 in NP_002118.1  238 in XP_016866305.1  141 in XP_016866306.1  141 in XP_016866307.1 |  |  |
| rs775653233 | CCG ⇒ ACG | P [Pro] ⇒ T [Thr] | A=0.00000 (1/247956, GnomAD_exome)  A=0.00001 (1/116102, ExAC) | 74 in NP_002118.1  79 in XP_016866305.1  74 in XP_016866306.1  74 in XP_016866307.1 |  |  |
| rs776034608 | GCG ⇒ GAG | A [Ala] ⇒ E [Glu] | A=0.00000 (1/248234, GnomAD_exome)  A=0.00001 (1/117280, ExAC) | 163 in NP_002118.1 168 in XP_016866305.1 |  |  |
| rs776226094 | ATG ⇒ ATA ATG ⇒ ATC | M [Met] ⇒ I [Ile] M [Met] ⇒ I [Ile] | A=0.00001 (2/248588, GnomAD_exome)  A=0.00001 (1/125568, TOPMED) | 4 in NP_002118.1  9 in XP_016866305.1  4 in XP_016866306.1  4 in XP_016866307.1 |  |  |
| rs776391546 | GAT ⇒ GAA GAT ⇒ GAC | D [Asp] ⇒ E [Glu] D [Asp] ⇒ D [Asp] | None | 143in NP_002118.1 148 in XP_016866305.1 |  |  |
| **rs776393668** | GAG ⇒ GTG | E [Glu] ⇒ V [Val] | T=0.00000 (1/247040, GnomAD_exome)  T=0.00001 (1/116796, ExAC) | **113** in NP_002118.1  118 in XP_016866305.1  113 in XP_016866306.1  113 in XP_016866307.1 |  |  |
| rs776839936 | GCC ⇒ GTC | A [Ala] ⇒ V [Val] | T=0.00001 (3/250994, GnomAD_exome)  T=0.00002 (2/121316, ExAC) | 223 in NP_002118.1  228 in XP_016866305.1  131 in XP_016866306.1  131 in XP_016866307.1 |  |  |
| rs777320486 | GAG ⇒ AAG | E [Glu] ⇒ K [Lys] | A=0.00001 (2/244194, GnomAD_exome)  A=0.00001 (1/110856, ExAC) | 43 in NP_002118.1  48 in XP_016866305.1  43 in XP_016866306.1  43 in XP_016866307.1 |  |  |
| rs777353157 | TTC ⇒ TTA TTC ⇒ TTT | F [Phe] ⇒ L [Leu] F [Phe] ⇒ F [Phe] | A=0.00006 (7/125568, TOPMED) | 60 in NP_002118.1  65 in XP_016866305.1  60 in XP_016866306.1  60 in XP_016866307.1 |  |  |
| rs778230490 | CCT ⇒ GCT | P [Pro] ⇒ A [Ala] | G=0.00000 (1/250878, GnomAD_exome)  G=0.00001 (1/121336, ExAC) | 217 in NP_002118.1  222 in XP_016866305.1  125 in XP_016866306.1  125 in XP_016866307.1 |  |  |
| rs778467118 | GTG ⇒ ATG | V [Val] ⇒ M [Met] | A=0.00003 (7/250730, GnomAD_exome)  A=0.00003 (4/121274, ExAC)  A=0.0000 (1/31362, GnomAD) | 213 in NP_002118.1  218 in XP_016866305.1  121 in XP_016866306.1  121 in XP_016866307.1 |  |  |
| rs778602107 | TAT ⇒ CAT | Y [Tyr] ⇒ H [His] | C=0.00001 (3/247530, GnomAD_exome)  C=0.00001 (1/116922, ExAC) | 140 in NP_002118.1 145 in XP_016866305.1 |  |  |
| rs778906176 | GGT ⇒ AGT | G [Gly] ⇒ S [Ser] | A=0.00001 (2/251256, GnomAD_exome)  A=0.00002 (2/121140, ExAC) | 310 in NP_002118.1  315 in XP_016866305.1  218 in XP_016866306.1  218 in XP_016866307.1 |  |  |
| rs779746040 | CTG ⇒ GTG | L [Leu] ⇒ V [Val] | G=0.00000 (1/251284, GnomAD_exome)  G=0.00001 (1/121334, ExAC) | 315 in NP_002118.1  320 in XP_016866305.1  223 in XP_016866306.1  223 in XP_016866307.1 |  |  |
| **rs780697086§** | TGC ⇒ AGC | C [Cys] ⇒ S [Ser] | A=0.00001 (3/249312, GnomAD_exome)  A=0.00001 (1/118034, ExAC) | **188§** in NP_002118.1 193 in XP_016866305.1 |  |  |
| rs780775783 | CGC ⇒ TGC | R [Arg] ⇒ C [Cys] | T=0.00004 (10/248334, GnomAD_exome)  T=0.00008 (9/117406, ExAC) | 106 in NP_002118.1  111 in XP_016866305.1  106 in XP_016866306.1  106 in XP_016866307.1 |  |  |
| rs781636203 | ACT ⇒ AAT | T [Thr] ⇒ N [Asn] | A=0.00000 (1/248996, GnomAD_exome)  A=0.00001 (1/117966, ExAC) | 97 in NP_002118.1  102 in XP_016866305.1  97 in XP_016866306.1  97 in XP_016866307.1 |  |  |
| **rs781774818§** | CCT ⇒ CAT | P [Pro] ⇒ H [His] | A=0.00002 (6/251050, GnomAD_exome)  A=0.00002 (3/120488, ExAC) | **259§** in NP_002118.1  264 in XP_016866305.1  167 in XP_016866306.1  167 in XP_016866307.1 |  |  |
| rs796748483 | ACT ⇒ ATT | T [Thr] ⇒ I [Ile] | T=0.00000 (1/251238, GnomAD_exome) | 323in NP_002118.1  328 in XP_016866305.1  231 in XP_016866306.1  231 in XP_016866307.1 |  |  |
| rs865916409 | GAT ⇒ AAT | D [Asp] ⇒ N [Asn] | None | 244in NP_002118.1  249 in XP_016866305.1  152 in XP_016866306.1  152 in XP_016866307.1 |  |  |
| **rs867319917§** | TGG ⇒ CGG | W [Trp] ⇒ R [Arg] | None | **157§** in NP_002118.1 162 in XP_016866305.1 |  |  |
| rs878913602 | GAG ⇒ AAG | E [Glu] ⇒ K [Lys] | None | 222 in NP_002118.1  227 in XP_016866305.1  130 in XP_016866306.1  130 in XP_016866307.1 |  |  |
| rs879086343 | ATA ⇒ ACA | I [Ile] ⇒ T [Thr] | None | 238 in NP_002118.1  243 in XP_016866305.1  146 in XP_016866306.1  146 in XP_016866307.1 |  |  |
| rs901286567 | GCG ⇒ GAG GCG ⇒ GTG | A [Ala] ⇒ E [Glu] A [Ala] ⇒ V [Val] | A=0.00001 (1/125568, TOPMED) | 160 in NP_002118.1 165 in XP_016866305.1 |  |  |
| rs944203144 | CTC ⇒ GTC | L [Leu] ⇒ V [Val] | None | 9 in NP_002118.1  14 in XP_016866305.1  9 in XP_016866306.1  9 in XP_016866307.1 |  |  |
| rs952707994 | GTG ⇒ ATG | V [Val] ⇒ M [Met] | A=0.00001 (1/125568, TOPMED) | 189 in NP_002118.1 194 in XP_016866305.1 |  |  |
| rs969558063 | CTG ⇒ GTG | L [Leu] ⇒ V [Val] | G=0.00002 (3/125568, TOPMED) | 127 in NP_002118.1 132 in XP_016866305.1 |  |  |
| rs976391413 | GAC ⇒ AAC | D [Asp] ⇒ N [Asn] | None | 220 in NP_002118.1  225 in XP_016866305.1  128 in XP_016866306.1  128 in XP_016866307.1 |  |  |
| rs994268951 | AAG ⇒ AAC | K [Lys] ⇒ N [Asn] | C=0.00002 (2/125568, TOPMED)  C=0.0000 (1/31406, GnomAD) | 170 in NP_002118.1 175 in XP_016866305.1 |  |  |
| rs1026642003 | ATG ⇒ ACG | M [Met] ⇒ T [Thr] | C=0.00000 (1/251238, GnomAD_exome)  C=0.00001 (1/125568, TOPMED) | 309 in NP_002118.1  314 in XP_016866305.1  217 in XP_016866306.1  217 in XP_016866307.1 |  |  |
| rs1043833437 | CCG ⇒ CTG | P [Pro] ⇒ L [Leu] | T=0.00005 (6/125568, TOPMED) | 291 in NP_002118.1  296 in XP_016866305.1  199 in XP_016866306.1  199 in XP_016866307.1 |  |  |
| s1052565663 | GGA ⇒ GAA | G [Gly] ⇒ E [Glu] | A=0.00000 (1/247256, GnomAD_exome) | 131 in NP_002118.1 136 in XP_016866305.1 |  |  |
| rs1156427519 | CTC ⇒ CGC | L [Leu] ⇒ R [Arg] | G=0.00001 (2/248410, GnomAD_exome)  G=0.00002 (2/125568, TOPMED) | 9 in NP_002118.1  14 in XP_016866305.1  9 in XP_016866306.1  9 in XP_016866307.1 |  |  |
| **rs1161818149§** | CTG ⇒ CAG CTG ⇒ CCG | L [Leu] ⇒ Q [Gln] L [Leu] ⇒ P [Pro] | None | **105§** in NP_002118.1  110 in XP_016866305.1  105 in XP_016866306.1  105 in XP_016866307.1 |  |  |
| rs1170941476 | CAG ⇒ CAC | Q [Gln] ⇒ H [His] | None | 242 in NP_002118.1  247 in XP_016866305.1  150 in XP_016866306.1  150 in XP_016866307.1 |  |  |
| rs1171540559 | CTG ⇒ GTG | L [Leu] ⇒ V [Val] | None | 17 in NP_002118.1  22 in XP_016866305.1  17 in XP_016866306.1  17 in XP_016866307.1 |  |  |
| rs1176466537 | GTG ⇒ GCG | V [Val] ⇒ A [Ala] | C=0.00000 (1/247902, GnomAD_exome) | 76 in NP_002118.1  81 in XP_016866305.1  76 in XP_016866306.1  76 in XP_016866307.1 |  |  |
| rs1177625476 | GAG ⇒ GAT | E [Glu] ⇒ D [Asp] | T=0.00001 (1/125568, TOPMED) | 256 in NP_002118.1  261 in XP_016866305.1  164 in XP_016866306.1  164 in XP_016866307.1 |  |  |
| rs1182630020 | AAC ⇒ AAA AAC ⇒ AAG | N [Asn] ⇒ K [Lys] N [Asn] ⇒ K [Lys] | G=0.00001 (1/125568, TOPMED) | 151 in NP_002118.1 156 in XP_016866305.1 |  |  |
| rs1192743893 | ATG ⇒ ACG | M [Met] ⇒ T [Thr] | None | 4 in NP_002118.1  9 in XP_016866305.1  4 in XP_016866306.1  4 in XP_016866307.1 |  |  |
| rs1197350317 | GTG ⇒ GCG | V [Val] ⇒ A [Ala] | C=0.00001 (1/125568, TOPMED) | 271 in NP_002118.1  276 in XP_016866305.1  179 in XP_016866306.1  179 in XP_016866307.1 |  |  |
| rs1199196375 | GGC ⇒ GAC | G [Gly] ⇒ D [Asp] | A=0.00001 (1/125568, TOPMED) | 40 in NP_002118.1  45 in XP_016866305.1  40 in XP_016866306.1  40 in XP_016866307.1 |  |  |
| rs1200509080 | GAG ⇒ GGG | E [Glu] ⇒ G [Gly] | G=0.00000 (1/251022, GnomAD_exome) | 287 in NP_002118.1  283 in XP_016866305.1  186 in XP_016866306.1  186 in XP_016866307.1 |  |  |
| **rs1200732770§** | GCC ⇒ GAC | A [Ala] ⇒ D [Asp] | None | **229§** in NP_002118.1  234 in XP_016866305.1  137 in XP_016866306.1  137 in XP_016866307.1 |  |  |
| rs1202168553 | GCG ⇒ GAG GCG ⇒ GGG | A [Ala] ⇒ E [Glu] A [Ala] ⇒ G [Gly] | G=0.00001 (1/125568, TOPMED) | 24 in NP_002118.1  29 in XP_016866305.1  24 in XP_016866306.1  29 in XP_016866307.1 |  |  |
| rs1202227237 | CCC ⇒ CAC CCC ⇒ CTC | P [Pro] ⇒ H [His] P [Pro] ⇒ L [Leu] | None | 6 in NP_002118.1  11 in XP_016866305.1  6 in XP_016866306.1  6 in XP_016866307.1 |  |  |
| rs1203737354 | GAC ⇒ CAC | D [Asp] ⇒ H [His] | C=0.00001 (2/248950, GnomAD_exome) | 98 in NP_002118.1  103 in XP_016866305.1  98 in XP_016866306.1  98 in XP_016866307.1 |  |  |
| rs1204402255 | AAC ⇒ GAC | N [Asn] ⇒ D [Asp] | None | 101 in NP_002118.1  106 in XP_016866305.1  101 in XP_016866306.1  101 in XP_016866307.1 |  |  |
| rs1208889824 | CCA ⇒ CGA | P [Pro] ⇒ R [Arg] | G=0.0001 (3/31354, GnomAD) | 4 in XP_016866305.1 |  |  |
| rs1210439429 | GGG ⇒ CGG | G [Gly] ⇒ R [Arg] | C=0.00001 (1/125568, TOPMED) | 42 in NP_002118.1  47 in XP_016866305.1  42 in XP_016866306.1  42 in XP_016866307.1 |  |  |
| rs1211467330 | ACC ⇒ CCC | T [Thr] ⇒ P [Pro] | C=0.00000 (1/250966, GnomAD_exome) | 305 in NP_002118.1  310 in XP_016866305.1  213 in XP_016866306.1  213 in XP_016866307.1 |  |  |
| rs1212068397 | ATG ⇒ GTG ATG ⇒ TTG | M [Met] ⇒ V [Val] M [Met] ⇒ L [Leu] | G=0.00000 (1/249922, GnomAD_exome)  G=0.00001 (1/125568, TOPMED)  T=0.00001 (1/125568, TOPMED) | 295 in NP_002118.1  300 in XP_016866305.1  203 in XP_016866306.1  203 in XP_016866307.1 |  |  |
| rs1212161987 | CGC ⇒ CAC | R [Arg] ⇒ H [His] | A=0.00001 (2/247260, GnomAD_exome)  A=0.00001 (1/125568, TOPMED) | 132 in NP_002118.1 137 in XP_016866305.1 |  |  |
| rs1216496794 | GTG ⇒ ATG | V [Val] ⇒ M [Met] | A=0.00001 (3/251178, GnomAD_exome)  A=0.00001 (1/125568, TOPMED)  A=0.0002 (6/31366, GnomAD) | 255 in NP_002118.1  260 in XP_016866305.1  163 in XP_016866306.1  163 in XP_016866307.1 |  |  |
| rs1217191769 | GCG ⇒ GGG | A [Ala] ⇒ G [Gly] | G=0.00001 (1/125568, TOPMED) | 65 in NP_002118.1  70 in XP_016866305.1  65 in XP_016866306.1  65 in XP_016866307.1 |  |  |
| rs1217517217 | GCG ⇒ CCG | A [Ala] ⇒ P [Pro] | C=0.00000 (1/247572, GnomAD_exome) | 65 in NP_002118.1  70 in XP_016866305.1  65 in XP_016866306.1  65 in XP_016866307.1 |  |  |
| rs1217958499 | CAC ⇒ TAC | H [His] ⇒ Y [Tyr] | None | 117 in NP_002118.1 122 in XP_016866305.1 |  |  |
| rs1218025680 | GAG ⇒ CAG GAG ⇒ TAG | E [Glu] ⇒ Q [Gln] E [Glu] ⇒ Ter[*] [AMB] | T=0.00000 (1/250992, GnomAD_exome) | 277 in NP_002118.1  282 in XP_016866305.1  185 in XP_016866306.1  185 in XP_016866307.1 |  |  |
| rs1219083684 | GAC ⇒ AAC | D [Asp] ⇒ N [Asn] | A=0.00000 (1/251210, GnomAD_exome)  A=0.00001 (1/125568, TOPMED) | 251 in NP_002118.1  256 in XP_016866305.1  159 in XP_016866306.1  159 in XP_016866307.1 |  |  |
| rs1220097996 | GAG ⇒ AAG | E [Glu] ⇒ K [Lys] | A=0.00000 (1/247782, GnomAD_exome) | 70 in NP_002118.1  75 in XP_016866305.1  70 in XP_016866306.1  70 in XP_016866307.1 |  |  |
| rs1220220876 | AGC ⇒ AAC | S [Ser] ⇒ N [Asn] | A=0.00000 (1/247464, GnomAD_exome) | 62 in NP_002118.1  67 in XP_016866305.1  62 in XP_016866306.1  62 in XP_016866307.1 |  |  |
| rs1231277366 | GCT ⇒ CCT | A [Ala] ⇒ P [Pro] | C=0.00001 (2/250856, GnomAD_exome)  C=0.00002 (2/125568, TOPMED) | 270 in NP_002118.1  275 in XP_016866305.1  178 in XP_016866306.1  178 in XP_016866307.1 |  |  |
| rs1232112927 | CAG ⇒ AAG | Q [Gln] ⇒ K [Lys] | A=0.00000 (1/249970, GnomAD_exome) | 300 in NP_002118.1  305 in XP_016866305.1  208 in XP_016866306.1  208 in XP_016866307.1 |  |  |
| rs1240191362 | CAG ⇒ CGG | Q [Gln] ⇒ R [Arg] | None | 103 in NP_002118.1  108 in XP_016866305.1  103 in XP_016866306.1  103 in XP_016866307.1 |  |  |
| rs1242436454 | GTG ⇒ ATG | V [Val] ⇒ M [Met] | A=0.00000 (1/250820, GnomAD_exome) | 330 in NP_002118.1  335 in XP_016866305.1  238 in XP_016866306.1  238 in XP_016866307.1 |  |  |
| rs1259179452 | GCC ⇒ GTC | A [Ala] ⇒ V [Val] | T=0.00000 (1/247288, GnomAD_exome) | 16 in NP_002118.1  21 in XP_016866305.1  16 in XP_016866306.1  16 in XP_016866307.1 |  |  |
| rs1259830650 | ATC ⇒ GTC | I [Ile] ⇒ V [Val] | G=0.00000 (1/245808, GnomAD_exome) | 47 in NP_002118.1  52 in XP_016866305.1  47 in XP_016866306.1  47 in XP_016866307.1 |  |  |
| rs1259972609 | GTG ⇒ GGG | V [Val] ⇒ G [Gly] | None | 2 in NP_002118.1  7 in XP_016866305.1  2 in XP_016866306.1  2 in XP_016866307.1 |  |  |
| **rs1260086927§** | CAG ⇒ CCG | Q [Gln] ⇒ P [Pro] | C=0.00001 (1/125568, TOPMED)  C=0.0000 (1/31358, GnomAD) | **96 §** in NP_002118.1  101 in XP_016866305.1  96 in XP_016866306.1  96 in XP_016866307.1 |  |  |
| rs1263870442 | GAG ⇒ GGG | E [Glu] ⇒ G [Gly] | G=0.00001 (2/249650, GnomAD_exome) | 197 in NP_002118.1 202 in XP_016866305.1 |  |  |
| rs1265409678§ | CTC ⇒ CGC | L [Leu] ⇒ R [Arg] | G=0.00001 (3/249920, GnomAD_exome)  G=0.0000 (1/31258, GnomAD) | 294 § in NP_002118.1  299 in XP_016866305.1  202 in XP_016866306.1  202 in XP_016866307.1 |  |  |
| rs1268237466 | ACC ⇒ AAC | T [Thr] ⇒ N [Asn] | A=0.00001 (1/125568, TOPMED) | 249 in NP_002118.1  254 in XP_016866305.1  157 in XP_016866306.1  157 in XP_016866307.1 |  |  |
| rs1275022648 | AGG ⇒ AAG | R [Arg] ⇒ K [Lys] | None | 68 in NP_002118.1  73 in XP_016866305.1  68 in XP_016866306.1  68 in XP_016866307.1 |  |  |
| rs1279028529 | ATG ⇒ GTG | M [Met] ⇒ V [Val] | None | 100 in NP_002118.1  105 in XP_016866305.1  100 in XP_016866306.1  100 in XP_016866307.1 |  |  |
| rs1282195283 | AGT ⇒ ATT | S [Ser] ⇒ I [Ile] | T=0.00000 (1/245744, GnomAD_exome) | 115 in NP_002118.1 120 in XP_016866305.1 |  |  |
| rs1282397235 | GCC ⇒ TCC | A [Ala] ⇒ S [Ser] | None | 48 in NP_002118.1  53 in XP_016866305.1  48 in XP_016866306.1  48 in XP_016866307.1 |  |  |
| rs1285867439 | TAT ⇒ CAT | Y [Tyr] ⇒ H [His] | None | 221 in NP_002118.1  226 in XP_016866305.1  129 in XP_016866306.1  129 in XP_016866307.1 |  |  |
| rs1302368390 | AGC ⇒ AAC AGC ⇒ ACC | S [Ser] ⇒ N [Asn] S [Ser] ⇒ T [Thr] | None | 33 in NP_002118.1  38 in XP_016866305.1  33 in XP_016866306.1  33 in XP_016866307.1 |  |  |
| rs1303676218 | CGG ⇒ TGG | R [Arg] ⇒ W [Trp] | T=0.00000 (1/242776, GnomAD_exome) | 38 in NP_002118.1  43 in XP_016866305.1  38 in XP_016866306.1  38 in XP_016866307.1 |  |  |
| rs1307121254 | GAG ⇒ GCG | E [Glu] ⇒ A [Ala] | C=0.00000 (1/250426, GnomAD_exome) | 292 in NP_002118.1  297 in XP_016866305.1  200 in XP_016866306.1  200 in XP_016866307.1 |  |  |
| rs1311016872 | CAG ⇒ GAG | Q [Gln] ⇒ E [Glu] | None | 103 in NP_002118.1  108 in XP_016866305.1  103 in XP_016866306.1  103 in XP_016866307.1 |  |  |
| rs1314779801 | GGA ⇒ CGA | G [Gly] ⇒ R [Arg] | C=0.00001 (1/125568, TOPMED) | 131 in NP_002118.1 136 in XP_016866305.1 |  |  |
| **rs1317292772§** | GAT ⇒ AAT GAT ⇒ CAT | D [Asp] ⇒ N [Asn] D [Asp] ⇒ H [His] | C=0.00002 (2/125568, TOPMED) | **143§** in NP_002118.1 148 in XP_016866305.1 |  |  |
| rs1319761119 | GCC ⇒ ACC | A [Ala] ⇒ T [Thr] | A=0.00000 (1/251146, GnomAD_exome) | 229 in NP_002118.1  234 in XP_016866305.1  137 in XP_016866306.1  137 in XP_016866307.1 |  |  |
| rs1323507021 | TTC ⇒ CTC | F [Phe] ⇒ L [Leu] | None | 10 in NP_002118.1  15 in XP_016866305.1  10 in XP_016866306.1  10 in XP_016866307.1 |  |  |
| rs1332402417 | ATG ⇒ GTG | M [Met] ⇒ V [Val] | None | 49 in NP_002118.1  54 in XP_016866305.1  49 in XP_016866306.1  49 in XP_016866307.1 |  |  |
| rs1340921299 | AGC ⇒ TGC | S [Ser] ⇒ C [Cys] | None | 33 in NP_002118.1  38 in XP_016866305.1  33 in XP_016866306.1  33 in XP_016866307.1 |  |  |
| rs1344510974 | GTG ⇒ ATG | V [Val] ⇒ M [Met] | None | 252 in NP_002118.1  257 in XP_016866305.1  160 in XP_016866306.1  160 in XP_016866307.1 |  |  |
| rs1348648082 | GTG ⇒ ATG | V [Val] ⇒ M [Met] | A=0.00000 (1/250884, GnomAD_exome) | 271 in NP_002118.1  276 in XP_016866305.1  179 in XP_016866306.1  179 in XP_016866307.1 |  |  |
| rs1351032573 | AAC ⇒ AGC | N [Asn] ⇒ S [Ser] | None | 101 in NP_002118.1  106 in XP_016866305.1  101 in XP_016866306.1  101 in XP_016866307.1 |  |  |
| rs1353178854 | ACC ⇒ GCC | T [Thr] ⇒ A [Ala] | None | 18 in NP_002118.1  23 in XP_016866305.1  18 in XP_016866306.1  18 in XP_016866307.1 |  |  |
| rs1356383148 | GTG ⇒ ATG | V [Val] ⇒ M [Met] | None | 273 in NP_002118.1  278 in XP_016866305.1  181 in XP_016866306.1  181 in XP_016866307.1 |  |  |
| rs1358080191 | CAC ⇒ CAG CAC ⇒ CAT | H [His] ⇒ Q [Gln] H [His] ⇒ H [His] | None | 94 in NP_002118.1  99 in XP_016866305.1  94 in XP_016866306.1  94 in XP_016866307.1 |  |  |
| rs1358790245 | AGT ⇒ GGT | S [Ser] ⇒ G [Gly] | None | 115 in NP_002118.1  120 in XP_016866305.1  115 in XP_016866306.1  115 in XP_016866307.1 |  |  |
| rs1361301951 | CGG ⇒ CAG | R [Arg] ⇒ Q [Gln] | A=0.00000 (1/243000, GnomAD_exome) | 38 in NP_002118.1  43 in XP_016866305.1  38 in XP_016866306.1  38 in XP_016866307.1 |  |  |
| rs1378591096 | ATT ⇒ ACT | I [Ile] ⇒ T [Thr] | C=0.00000 (1/247008, GnomAD_exome) | 123 in NP_002118.1 128 in XP_016866305.1 |  |  |
| rs1379239345 | CCC ⇒ TCC | P [Pro] ⇒ S [Ser] | T=0.00000 (1/250056, GnomAD_exome) | 293 in NP_002118.1  298 in XP_016866305.1  201 in XP_016866306.1  201 in XP_016866307.1 |  |  |
| rs1379549384 | GTG ⇒ TTG | V [Val] ⇒ L [Leu] | T=0.00001 (1/125568, TOPMED) | 58 in NP_002118.1  63 in XP_016866305.1  58 in XP_016866306.1  58 in XP_016866307.1 | G*01:19 (Substitution: V58L) |  |
| **rs1379742188§** | CGC ⇒ AGC | R [Arg] ⇒ S [Ser] | A=0.00000 (1/249572, GnomAD_exome) | **205§** in NP_002118.1 210 in XP_016866305.1 |  |  |
| rs1381435641 | ACC ⇒ AAC | T [Thr] ⇒ N [Asn] | None | 104 in NP_002118.1  109 in XP_016866305.1  104 in XP_016866306.1  104 in XP_016866307.1 |  |  |
| rs1384515198 | CTC ⇒ ATC | L [Leu] ⇒ I [Ile] | None | 148 in NP_002118.1 153 in XP_016866305.1 |  |  |
| rs1385852462 | GCA ⇒ GAA GCA ⇒ GTA | A [Ala] ⇒ E [Glu] A [Ala] ⇒ V [Val] | None | 319 in NP_002118.1  324 in XP_016866305.1  227 in XP_016866306.1  227 in XP_016866307.1 |  |  |
| **rs1390270595§** | TAC ⇒ TGC | Y [Tyr] ⇒ C [Cys] | None | **51§** in NP_002118.1  56 in XP_016866305.1  51 in XP_016866306.1  51 in XP_016866307.1 |  |  |
| **rs1397132797§** | CTG ⇒ CCG | L [Leu] ⇒ P [Pro] | C=0.00002 (3/125568, TOPMED) | **196§** in NP_002118.1 201 in XP_016866305.1 |  |  |
| rs1399267444 | GAC ⇒ TAC | D [Asp] ⇒ Y [Tyr] | T=0.00000 (1/251200, GnomAD_exome) | 247 in NP_002118.1  252 in XP_016866305.1  155 in XP_016866306.1  155 in XP_016866307.1 |  |  |
| **rs1414848134§** | GAC ⇒ GTC | D [Asp] ⇒ V [Val] | T=0.00000 (1/247070, GnomAD_exome) | **54§** in NP_002118.1  59 in XP_016866305.1  54 in XP_016866306.1  54 in XP_016866307.1 |  |  |
| rs1415054338 | CGG ⇒ CAG | R [Arg] ⇒ Q [Gln] | A=0.0000 (1/31390, GnomAD) | 59 in NP_002118.1  64 in XP_016866305.1  59 in XP_016866306.1  59 in XP_016866307.1 |  |  |
| rs1417469901 | GGG ⇒ AGG | G [Gly] ⇒ R [Arg] | None | 245 in NP_002118.1  250 in XP_016866305.1  153 in XP_016866306.1  153 in XP_016866307.1 |  |  |
| rs1420195749 | CAT ⇒ TAT | H [His] ⇒ Y [Tyr] | T=0.00000 (1/250892, GnomAD_exome) | 284 in NP_002118.1  289 in XP_016866305.1  192 in XP_016866306.1  192 in XP_016866307.1 |  |  |
| rs1421876745 | ACC ⇒ ATC | T [Thr] ⇒ I [Ile] | T=0.00002 (4/251140, GnomAD_exome) | 240 in NP_002118.1  245 in XP_016866305.1  148 in XP_016866306.1  148 in XP_016866307.1 |  |  |
| rs1423727357 | ATT ⇒ ATG | I [Ile] ⇒ M [Met] | None | 123 in NP_002118.1 128 in XP_016866305.1 |  |  |
| rs1424014138 | GCT ⇒ GTT | A [Ala] ⇒ V [Val] | None | 177 in NP_002118.1 182 in XP_016866305.1 |  |  |
| rs1425089505 | GAG ⇒ GTG | E [Glu] ⇒ V [Val] | T=0.00000 (1/247762, GnomAD_exome) | 152 in NP_002118.1 157 in XP_016866305.1 |  |  |
| rs1426237465 | ATG ⇒ GTG | M [Met] ⇒ V [Val] | None | 29 in NP_002118.1  34 in XP_016866305.1  29 in XP_016866306.1  29 in XP_016866307.1 |  |  |
| **rs1430565057§** | CCT ⇒ CTT | P [Pro] ⇒ L [Leu] | T=0.00001 (1/125568, TOPMED) | **234§** in NP_002118.1  239 in XP_016866305.1  142 in XP_016866306.1  142 in XP_016866307.1 |  |  |
| **rs1438362414** | CGC ⇒ CTC | R [Arg] ⇒ L [Leu] | T=0.00001 (1/125568, TOPMED) | **205** in NP_002118.1 210 in XP_016866305.1 |  |  |
| rs1438528736 | ACG ⇒ GCG | T [Thr] ⇒ A [Ala] | G=0.00000 (1/251084, GnomAD_exome) | 282 in NP_002118.1  287 in XP_016866305.1  190 in XP_016866306.1  190 in XP_016866307.1 |  |  |
| rs1440397389 | CAG ⇒ GAG | Q [Gln] ⇒ E [Glu] | None | 96 in NP_002118.1  101 in XP_016866305.1  96 in XP_016866306.1  96 in XP_016866307.1 |  |  |
| rs1442285056 | CAT ⇒ CTT | H [His] ⇒ L [Leu] | None | 284 in NP_002118.1  289 in XP_016866305.1  192 in XP_016866306.1  192 in XP_016866307.1 |  |  |
| rs1444084886 | ACC ⇒ GCC | T [Thr] ⇒ A [Ala] | G=0.00000 (1/248612, GnomAD_exome) | 104 in NP_002118.1  109 in XP_016866305.1  104 in XP_016866306.1  104 in XP_016866307.1 |  |  |
| rs1446958325 | TAT ⇒ TGT | Y [Tyr] ⇒ C [Cys] | G=0.00002 (2/125568, TOPMED) | 83 in NP_002118.1  88 in XP_016866305.1  83 in XP_016866306.1  83 in XP_016866307.1 |  |  |
| rs1448777822 | GTC ⇒ ATC | V [Val] ⇒ I [Ile] | A=0.00001 (2/248548, GnomAD_exome) | 3 in NP_002118.1  8 in XP_016866305.1  3 in XP_016866306.1  3 in XP_016866307.1 |  |  |
| rs1449528925 | AGC ⇒ AAC | S [Ser] ⇒ N [Asn] | A=0.00000 (1/247396, GnomAD_exome) | 112 in NP_002118.1  117 in XP_016866305.1  112 in XP_016866306.1  112 in XP_016866307.1 |  |  |
| rs1450077461 | CGC ⇒ AGC | R [Arg] ⇒ S [Ser] | None | 41 in NP_002118.1  46 in XP_016866305.1  41 in XP_016866306.1  41 in XP_016866307.1 |  |  |
| rs1455936700 | CTC ⇒ TTC | L [Leu] ⇒ F [Phe] | None | 133 in NP_002118.1 138 in XP_016866305.1 |  |  |
| rs1456557492 | CCC ⇒ CTC | P [Pro] ⇒ L [Leu] | T=0.00000 (1/249920, GnomAD_exome) | 208 in NP_002118.1  213 in XP_016866305.1  116 in XP_016866306.1  116 in XP_016866307.1 |  |  |
| rs1458151590 | TCG ⇒ TTG | S [Ser] ⇒ L [Leu] | T=0.00001 (1/125568, TOPMED) | 64 in NP_002118.1  69 in XP_016866305.1  64 in XP_016866306.1  64 in XP_016866307.1 |  |  |
| rs1459341178 | GAA ⇒ AAA GAA ⇒ CAA | E [Glu] ⇒ K [Lys] E [Glu] ⇒ Q [Gln] | A=0.00000 (1/247446, GnomAD_exome)  A=0.0000 (1/31390, GnomAD) | 138 in NP_002118.1 143 in XP_016866305.1 |  |  |
| rs1461052993 | GCC ⇒ TCC | A [Ala] ⇒ S [Ser] | None | 35 in NP_002118.1  40 in XP_016866305.1  35 in XP_016866306.1  35 in XP_016866307.1 |  |  |
| rs1462477198 | GTG ⇒ GGG | V [Val] ⇒ G [Gly] | G=0.00002 (2/125568, TOPMED) | 255 in NP_002118.1  260 in XP_016866305.1  163 in XP_016866306.1  163 in XP_016866307.1 |  |  |
| rs1463712721 | CCT ⇒ ACT | P [Pro] ⇒ T [Thr] | A=0.00001 (1/125568, TOPMED) | 259 in NP_002118.1  264 in XP_016866305.1  167 in XP_016866306.1  167 in XP_016866307.1 |  |  |
| rs1466305795 | GCA ⇒ ACA | A [Ala] ⇒ T [Thr] | A=0.00000 (1/248060, GnomAD_exome) | 159 in NP_002118.1 164 in XP_016866305.1 |  |  |
| rs1466828249 | CCT ⇒ CTT | P [Pro] ⇒ L [Leu] | None | 217 in NP_002118.1  222 in XP_016866305.1  125 in XP_016866306.1  125 in XP_016866307.1 |  |  |
| rs1469197662 | GAG ⇒ GAT | E [Glu] ⇒ D [Asp] | T=0.00002 (2/125568, TOPMED) | 21 in NP_002118.1  26 in XP_016866305.1  21 in XP_016866306.1  21 in XP_016866307.1 |  |  |
| rs1469252648 | GGC ⇒ CGC | G [Gly] ⇒ R [Arg] | C=0.00000 (1/247014, GnomAD_exome) | 124 in NP_002118.1 129 in XP_016866305.1 |  |  |
| **rs1472538844§** | CCC ⇒ CGC | P [Pro] ⇒ R [Arg] | G=0.00000 (1/250580, GnomAD_exome) | **209§** in NP_002118.1  214 in XP_016866305.1  117 in XP_016866306.1  117 in XP_016866307.1 |  |  |
| **rs1475659109§** | CCC ⇒ CTC | P [Pro] ⇒ L [Leu] | T=0.0000 (1/31366, GnomAD) | **39§** in NP_002118.1  44 in XP_016866305.1  39 in XP_016866306.1  39 in XP_016866307.1 |  |  |
| rs1476760586 | GCG ⇒ GAG | A [Ala] ⇒ E [Glu] | A=0.00000 (1/248646, GnomAD_exome) | 173 in NP_002118.1 178 in XP_016866305.1 |  |  |
| rs1480037203 | GTG ⇒ ATG | V [Val] ⇒ M [Met] | A=0.00000 (1/246660, GnomAD_exome) | 52 in NP_002118.1  57 in XP_016866305.1  52 in XP_016866306.1  52 in XP_016866307.1 |  |  |
| rs1484457198 | ATC ⇒ GTC | I [Ile] ⇒ V [Val] | G=0.0000 (1/31348, GnomAD) | 308 in NP_002118.1  313 in XP_016866305.1  216 in XP_016866306.1  216 in XP_016866307.1 |  |  |
| rs1484591795 | GGC ⇒ GAC | G [Gly] ⇒ D [Asp] | A=0.00000 (1/246428, GnomAD_exome) | 50 in NP_002118.1  55 in XP_016866305.1  50 in XP_016866306.1  50 in XP_016866307.1 |  |  |
| rs1489890404 | GAG ⇒ AAG | E [Glu] ⇒ K [Lys] | A=0.00001 (3/246236, GnomAD_exome) | 21 in NP_002118.1  26 in XP_016866305.1  21 in XP_016866306.1  21 in XP_016866307.1 |  |  |
| rs1490053246 | CCG ⇒ CAG | P [Pro] ⇒ Q [Gln] | A=0.00000 (1/247566, GnomAD_exome) | 67 in NP_002118.1  72 in XP_016866305.1  67 in XP_016866306.1  67 in XP_016866307.1 |  |  |

Missense SNPs were predicted as deleterious in isoform 1 (HLA-G1) by all eight servers are shown with **§** sign. Missense SNPs were predicted as deleterious in isoform 5 (HLA-G5) by all eight servers are shown in the Bold font. A thick black underline shows missense SNPs that were predicted as deleterious in all membrane-bound HLA-G isoforms (HLA-G1-4). A black oval shows missense SNPs that were predicted as deleterious in all soluble HLA-G isoforms (HLA-G5-7). Global minor allele frequency (MAF) is actually the second most frequent allele value. For example, rs12722477 reports: MAF Count: A=0.203 (1015/5008, 1000G). This means that for rs12722477, minor allele is ' A ' and has a frequency of 20.3% in the 1000Genome population and that 'A' is observed 1015 times in the sample population of 5008 people.

**Supplementary Table 2.** SNPs analyzed in isoform 5 by SIFT, PROVEAN, Polyphen 2.0, I-mutant 3.0, SNPs&GO

| **Isoform 5** | | | | | | | | | | | | | | | |
| --- | --- | --- | --- | --- | --- | --- | --- | --- | --- | --- | --- | --- | --- | --- | --- |
| **SNP rsID** | **Codons** | **Substitution** | **SIFT prediction** | | **PROVEAN prediction** | | **PolyPhen-2 prediction** | | | | **I-Mutant DDG** | | **SNPs&GO prediction** | | |
|  |  |  | **Prediction** | **Score** | **Prediction of** | **Score** | **Prediction** | **Score** | **Sensitivity** | **Specificity** | **SVM3 Prediction Effect** | **DDG Value Prediction** | **Prediction** | **RI** | **Probability** |
| rs17851921 | CAC ⇒ CCC CAC ⇒ CTC | H117P H117L | DAMAGING | 0 | Deleterious Deleterious | -9.121 -10.015 | Probably damaging  Probably damaging | 1.000 1.000 | 0.00 0.00 | 1.00 1.00 | Large Increase Large Increase | 0.33 0.65 | Disease Disease | 6 6 | \| 0.805 0.793 \|  \| \| --- \| --- \| |
| rs138289952 | GAC ⇒ AAC | D54Y | DAMAGING | 0.04 | Deleterious | -6.874 | Probably damaging | 1.000 | 0.00 | 1.00 | Large Decrease | -0.32 | Disease | 7 | 0.832 |
| rs142596947 | CCT ⇒ ACT | P234T | DAMAGING | 0 | Deleterious | -5.309 | Probably damaging | 1.000 | 0.00 | 1.00 | Large Decrease | -1.08 | Disease | 1 | 0.557 |
| rs144577485 | CCC ⇒ GCC | P209A | DAMAGING | 0 | Deleterious | -5.598 | Probably damaging | 1.000 | 0.00 | 1.00 | Large Decrease | -0.99 | Disease | 1 | 0.574 |
| rs145097667 | CAT ⇒ TAT | H287Y | DAMAGING | 0 | Deleterious | -3.958 | Probably damaging | 0.998 | 0.27 | 0.99 | Large Increase | 0.54 | Disease | 5 | 0.743 |
| rs540632198 | TTC ⇒ TGC | F32C | DAMAGING | 0 | Deleterious | -5.85 | Probably damaging | 1.000 | 0.00 | 1.00 | Large Decrease | -1.46 | Disease | 1 | 0.529 |
| rs572025435 | AGG ⇒ AGT | R30S | DAMAGING | 0 | Deleterious | -3.832 | Probably damaging | 0.972 | 0.77 | 0.96 | Large Decrease | -1.17 | Disease | 1 | 0.541 |
| rs748013931 | ACC ⇒ CCC | T158P | DAMAGING | 0 | Deleterious | -5.223 | Probably damaging | 0.983 | 0.74 | 0.96 | Large Decrease | -0.52 | Disease | 4 | 0.697 |
| rs749006959 | TAT ⇒ TGT | Y142C | DAMAGING | 0 | Deleterious | -8.264 | Probably damaging | 1.000 | 0.00 | 1.00 | Large Increase | -1.13 | Disease | 5 | 0.749 |
| rs750238738 | ATC ⇒ TTC | I237F | DAMAGING | 0 | Deleterious | -2.666 | Probably damaging | 1.000 | 0.00 | 1.00 | Large Decrease | -1.46 | Disease | 6 | 0.779 |
| rs760500349 | CAG ⇒ CTG | Q266L | DAMAGING | 0.04 | Deleterious | -4.628 | Probably damaging | 1.000 | 0.00 | 1.00 | Large Increase | 0.19 | Disease | 4 | 0.705 |
| rs763201540 | GAC ⇒ AAC GAC ⇒ TAC | D53N D53Y | DAMAGING | 0 | Deleterious  Deleterious | -3.905 -7.055 | Probably damaging Probably damaging | 1.000 1.000 | 0.00 0.00 | 1.00 1.00 | Large Decrease Large Decrease | -1.07 -0.29 | Disease Disease | 5 7 | \| 0.726 0.871 \|  \| \| --- \| --- \| |
| rs765275727 | TGG ⇒ CGG | W298R | DAMAGING | 0 | Deleterious | -9.014 | Probably damaging | 1.000 | 0.00 | 1.00 | Large Decrease | -0.85 | Disease | 7 | 0.828 |
| rs770027530 | TGC ⇒ TAC TGC ⇒ TTC | C227Y C227F | DAMAGING | 0 | Deleterious Deleterious | -7.637 -7.635 | Probably damaging Probably damaging | 1.000 1.000 | 0.00 0.00 | 1.00 1.00 | Large Decrease Large Decrease | -0.12 0.06 | Disease Disease | 7 8 | \| 0.854 0.882 \|  \| \| --- \| --- \| |
| rs772834879 | TAC ⇒ CAC | Y142H | DAMAGING | 0 | Deleterious | -4.581 | Probably damaging | 1.000 | 0.00 | 1.00 | Large Decrease | -1.31 | Disease | 3 | 0.635 |
| rs780697086 | TGC ⇒ AGC | C188S | DAMAGING | 0 | Deleterious | -8.689 | Probably damaging | 0.999 | 0.14 | 0.99 | Large Decrease | -0.8 | Disease | 5 | 0.768 |
| rs781774818 | CCT ⇒ CAT | P259H | DAMAGING | 0 | Deleterious | -5.987 | Probably damaging | 1.000 | 0.00 | 1.00 | Large Decrease | -1.22 | Disease | 2 | 0.580 |
| rs867319917 | TGG ⇒ CGG | W157R | DAMAGING | 0 | Deleterious | -12.775 | Probably damaging | 1.000 | 0.00 | 1.00 | Large Decrease | -0.86 | Disease | 6 | 0.791 |
| rs1200732770 | GCC ⇒ GAC | A229D | DAMAGING | 0 | Deleterious | -4.124 | Probably damaging | 1.000 | 0.00 | 1.00 | Large Decrease | -0.87 | Disease | 6 | 0.776 |
| rs1260086927 | CAG ⇒ CCG | Q96P | DAMAGING | 0.01 | Deleterious | -5.005 | Probably damaging | 0.993 | 0.70 | 0.97 | Large Decrease | -0.4 | Disease | 0 | 0.523 |
| rs1317292772 | GAT ⇒ AAT GAT ⇒ CAT | D143N D143H | DAMAGING | 0 | Deleterious Deleterious | -4.484 -6.27 | Probably damaging Probably damaging | 1.000 1.000 | 0.00 0.00 | 1.00 1.00 | Large Decrease Large Decrease | -0.55 -0.24 | Disease Disease | 3 3 | \| 0.659 0.640 \|  \| \| --- \| --- \| |
| rs1379742188 | CGC ⇒ AGC | R205S | DAMAGING | 0.02 | Deleterious | -4.471 | Probably damaging | 0.999 | 0.14 | 0.99 | Large Decrease | -1.2 | Disease | 3 | 0.639 |
| rs1390270595 | TAC ⇒ TGC | Y51C | DAMAGING | 0.03 | Deleterious | -6.87 | Probably damaging | 1.000 | 0.00 | 1.00 | Large Decrease | -1.29 | Disease | 4 | 0.723 |
| rs1397132797 | CTG ⇒ CCG | L196P | DAMAGING | 0.01 | Deleterious | -5.73 | Probably damaging | 1.000 | 0.00 | 1.00 | Large Decrease | -1.66 | Disease | 5 | 0.760 |
| rs1414848134 | GAC ⇒ GTC | D54V | DAMAGING | 0 | Deleterious | -6.866 | Probably damaging | 0.995 | 0.68 | 0.97 | Large Decrease | -1.04 | Disease | 7 | 0.874 |
| rs1430565057 | CCT ⇒ CTT | P234L | DAMAGING | 0 | Deleterious | -6.637 | Probably damaging | 1.000 | 0.00 | 1.00 | Large Decrease | -0.33 | Disease | 4 | 0.711 |
| rs1438362414 | CGC ⇒ CTC | R205L | DAMAGING | 0.01 | Deleterious | -5.302 | Probably damaging | 0.996 | 0.55 | 0.98 | Large Increase | -0.25 | Disease | 3 | 0.674 |
| rs1472538844 | CCC ⇒ CGC | P209R | DAMAGING | 0 | Deleterious | -6.307 | Probably damaging | 1.000 | 0.00 | 1.00 | Large Decrease | -0.61 | Disease | 5 | 0.763 |
| rs555347515 | ATG ⇒ AAG | M29K | DAMAGING | 0 | Deleterious | -3.594 | Probably damaging | 0.998 | 0.27 | 0.99 | Large Decrease | -1.48 | Disease | 5 | 0.774 |
| rs556645753 | GAC ⇒ GGC | D153G | DAMAGING | 0 | Deleterious | -6.367 | Probably damaging | 0.999 | 0.14 | 0.99 | Large Decrease | -0.73 | Disease | 0 | 0.509 |
| rs565858069 | GAC ⇒ CAC | D130H | DAMAGING | 0.03 | Deleterious | -6.015 | Probably damaging | 0.999 | 0.14 | 0.99 | Large Decrease | -0.49 | Disease | 3 | 0.647 |
| rs770412396 | CTG ⇒ CCG | L102P | DAMAGING | 0.01 | Deleterious | -6.241 | Probably damaging | 1.000 | 0.00 | 1.00 | Large Decrease | -1.63 | Disease | 6 | 0.786 |
| rs776393668 | GAG ⇒ GTG | E113V | DAMAGING | 0 | Deleterious | -5.73 | Possibly damaging | 0.854 | 0.83 | 0.93 | Large Increase | 0.22 | Disease | 0 | 0.517 |
| rs1161818149 | CTG ⇒ CAG CTG ⇒ CCG | L105Q L105P | DAMAGING | 0 | Deleterious Deleterious | -4.564 -5.34 | Probably damaging Probably damaging | 1.000 1.000 | 0.00 0.00 | 1.00 1.00 | Large Decrease Large Decrease | -2.04  -1.69 | Disease Disease | 5 6 | 0.764 0.799 |
| rs1475659109 | CCC ⇒ CTC | P44L | DAMAGING | 0 | Deleterious | -7.438 | Probably damaging | 1.000 | 0.00 | 1.00 | Large Increase | -0.51 | Disease | 1 | 0.542 |

**Supplementary Table 3.** SNPs analyzed in isoform 5 by PhD-SNP, SNAP2, MUpro

| **Isoform 5** | | | | | | | | | |
| --- | --- | --- | --- | --- | --- | --- | --- | --- | --- |
| **SNP rsID** | **Codons** | **Substitution** | **PhD-SNP prediction** | | **SNAP2** | | | **MUpro** | |
|  |  |  | **Prediction** | **Score** | **Prediction** | **Score** | **Expected Accuracy** | **Prediction** | **DDG Value** |
| rs17851921 | CAC ⇒ CCC CAC ⇒ CTC | H117P H117L | Disease Disease | 5 6 | effect effect | 85 71 | 91% 85% | DECREASE INCREASE | -0.94483558 0.17248775 |
| rs138289952 | GAC ⇒ AAC | D54Y | Disease | 8 | effect | 58 | 75% | DECREASE | -0.65574885 |
| rs142596947 | CCT ⇒ ACT | P234T | Disease | 8 | effect | 27 | 63% | DECREASE | -0.8378542 |
| rs144577485 | CCC ⇒ GCC | P209A | Disease | 4 | effect | 24 | 63% | DECREASE | -1.3457662 |
| rs145097667 | CAT ⇒ TAT | H287Y | Disease | 6 | effect | 76 | 85% | INCREASE | 0.0017512632 |
| rs540632198 | TTC ⇒ TGC | F32C | Disease | 0 | effect | 23 | 63% | DECREASE | -0.87381309 |
| rs572025435 | AGG ⇒ AGT | R30S | Disease | 4 | effect | 21 | 63% | DECREASE | -1.582361 |
| rs748013931 | ACC ⇒ CCC | T158P | Disease | 3 | effect | 38 | 66% | DECREASE | -1.0769639 |
| rs749006959 | TAT ⇒ TGT | Y142C | Disease | 7 | effect | 64 | 80% | DECREASE | -1.7925753 |
| rs750238738 | ATC ⇒ TTC | I237F | Disease | 5 | effect | 37 | 66% | DECREASE | -0.97093071 |
| rs760500349 | CAG ⇒ CTG | Q266L | Disease | 6 | effect | 63 | 80% | DECREASE | -0.052279107 |
| rs763201540 | GAC ⇒ AAC GAC ⇒ TAC | D53N D53Y | Disease Disease | 5 9 | effect effect | 47 83 | 71% 91% | DECREASE DECREASE | -0.79149414 -0.45294328 |
| rs765275727 | TGG ⇒ CGG | W298R | Disease | 7 | effect | 69 | 80% | DECREASE | -0.98499465 |
| rs770027530 | TGC ⇒ TAC TGC ⇒ TTC | C227Y C227F | Disease Disease | 8 6 | effect effect | 85 84 | 91% 91% | DECREASE DECREASE | -0.88892871 -0.71910267 |
| rs772834879 | TAC ⇒ CAC | Y142H | Disease | 5 | effect | 89 | 91% | DECREASE | -2.1531554 |
| rs780697086 | TGC ⇒ AGC | C188S | Disease | 4 | effect | 55 | 75% | DECREASE | -1.0332928 |
| rs781774818 | CCT ⇒ CAT | P259H | Disease | 5 | effect | 43 | 71% | DECREASE | -1.1893017 |
| rs867319917 | TGG ⇒ CGG | W157R | Disease | 3 | effect | 90 | 95% | DECREASE | -0.91293557 |
| rs1200732770 | GCC ⇒ GAC | A229D | Disease | 8 | effect | 68 | 80% | DECREASE | -0.6414612 |
| rs1260086927 | CAG ⇒ CCG | Q96P | Disease | 6 | effect | 50 | 75% | DECREASE | -0.97929591 |
| rs1317292772 | GAT ⇒ AAT GAT ⇒ CAT | D143N D143H | Disease Disease | 4 6 | effect effect | 51 47 | 75% 71% | DECREASE DECREASE | -1.2006322 -1.2840175 |
| rs1379742188 | CGC ⇒ AGC | R205S | Disease | 5 | effect | 41 | 71% | DECREASE | -0.52183883 |
| rs1390270595 | TAC ⇒ TGC | Y51C | Disease | 2 | effect | 1 | 53% | DECREASE | -0.83530596 |
| rs1397132797 | CTG ⇒ CCG | L196P | Disease | 8 | effect | 54 | 75% | DECREASE | -2.0384073 |
| rs1414848134 | GAC ⇒ GTC | D54V | Disease | 5 | effect | 69 | 80% | DECREASE | -0.37383825 |
| rs1430565057 | CCT ⇒ CTT | P234L | Disease | 7 | effect | 57 | 75% | INCREASE | 0.3342289 |
| rs1438362414 | CGC ⇒ CTC | R205L | Disease | 8 | effect | 40 | 71% | DECREASE | -1.0509858 |
| rs1472538844 | CCC ⇒ CGC | P209R | Disease | 3 | effect | 29 | 63% | DECREASE | -1.0509858 |
| rs555347515 | ATG ⇒ AAG | M29K | Disease | 6 | effect | 80 | 91% | DECREASE | -1.7776487 |
| rs556645753 | GAC ⇒ GGC | D153G | Disease | 2 | effect | 42 | 71% | DECREASE | -2.384922 |
| rs565858069 | GAC ⇒ CAC | D130H | Disease | 5 | effect | 7 | 53% | DECREASE | -1.0708696 |
| rs770412396 | CTG ⇒ CCG | L102P | Disease | 7 | effect | 72 | 85% | DECREASE | -2.238046 |
| rs776393668 | GAG ⇒ GTG | E113V | Disease | 6 | effect | 55 | 75% | DECREASE | -0.25206795 |
| rs1161818149 | CTG ⇒ CAG CTG ⇒ CCG | L105Q L105P | Disease Disease | 3 6 | effect effect | 56 67 | 75% 80% | DECREASE DECREASE | -1.8766335 -2.1510154 |
| rs1475659109 | CCC ⇒ CTC | P44L | Disease | 6 | effect | 25 | 63% | DECREASE | -0.074155665 |

**Supplementary Table 4.** SNPs analyzed in all membrane-bound HLA-G isoforms (HLA-G1-4) by SIFT, PROVEAN, Polyphen 2.0, I-mutant 3.0, SNPs&GO

| **Isoform 1** | | | | | | | | | | | | | | | |
| --- | --- | --- | --- | --- | --- | --- | --- | --- | --- | --- | --- | --- | --- | --- | --- |
| **SNP rsID** | **Codons** | **Substitution** | **SIFT prediction** | | **PROVEAN prediction** | | **PolyPhen-2 prediction** | | | | **I-Mutant DDG** | | **SNPs&GO prediction** | | |
|  |  |  | **Prediction** | **Score** | **Prediction of** | **Score** | **Prediction** | **Score** | **Sensitivity** | **Specificity** | **SVM3 Prediction Effect** | **DDG Value Prediction** | **Prediction** | **RI** | **Probability** |
| rs555347515 | ATG ⇒ AAG | M29K | DAMAGING | 0 | Deleterious | -3.427 | Probably damaging | 0.996 | 0.55 | 0.98 | Large Decrease | -1.48 | Disease | 5 | 0.774 |
| rs572025435 | AGG ⇒ AGT | R30S | DAMAGING | 0 | Deleterious | -3.653 | Probably damaging | 0.991 | 0.71 | 0.97 | Large Decrease | -1.17 | Disease | 1 | 0.542 |
| rs763201540 | GAC ⇒ AAC GAC ⇒ TAC | D53N D53Y | DAMAGING | 0 | Deleterious Deleterious | -3.842 -6.941 | Probably damaging  Probably damaging | 1.000 1.000 | 0.00 0.00 | 1. 00 1. 00 | Large Decrease Large Decrease | -1.07 -0.29 | Disease Disease | 5 7 | \| 0.728 0.872 \|  \| \| --- \| --- \| |
| rs1161818149 | CTG ⇒ CAG CTG ⇒ CCG | L105Q L105P | DAMAGING | 0 | Deleterious Deleterious | -4.572 -5.349 | Probably damaging  Probably damaging | 1.000 1.000 | 0.00 0.00 | 1.00 1.00 | Large Decrease Large Decrease | -2.04 -1.69 | Disease Disease | 5 6 | \| 0.764 0.799 \|  \| \| --- \| --- \| |
| rs1260086927 | CAG ⇒ CCG | Q96P | DAMAGING | 0.01 | Deleterious | -4.891 | Probably damaging | 0.996 | 0.55 | 0.98 | Large Decrease | -0.4 | Disease | 0 | 0.524 |
| rs1390270595 | TAC ⇒ TGC | Y51C | DAMAGING | 0.03 | Deleterious | -6.756 | Probably damaging | 1.000 | 0.00 | 1.00 | Large Decrease | -1.29 | Disease | 4 | 0.724 |
| rs1414848134 | GAC ⇒ GTC | D54V | DAMAGING | 0 | Deleterious | -6.752 | Possibly damaging | 0.893 | 0.82 | 0.94 | Large Decrease | -1.04 | Disease | 7 | 0.874 |
| rs770412396 | CTG ⇒ CCG | L102P | DAMAGING | 0.01 | Deleterious | -6.187 | Probably damaging | 1.000 | 0.00; | 1.00 | Large Decrease | -1.63 | Disease | 6 | 0.786 |
| **Isoform 2** | | | | | | | | | | | | | | | |
| **SNP rsID** | **Codons** | **Substitution** | **SIFT prediction** | | **PROVEAN prediction** | | **PolyPhen-2 prediction** | | | | **I-Mutant DDG** | | **SNPs&GO prediction** | | |
|  |  |  | **Prediction** | **Score** | **Prediction of** | **Score** | **Prediction** | **Score** | **Sensitivity** | **Specificity** | **SVM3 Prediction Effect** | **DDG Value Prediction** | **Prediction** | **RI** | **Prediction** |
| rs555347515 | ATG ⇒ AAG | M29K | DAMAGING | 0 | Deleterious | -3.414 | Probably damaging | 0.988 | 0.73 | 0.96 | Large Decrease | -1.48 | Disease | 5 | 0.757 |
| rs572025435 | AGG ⇒ AGT | R30S | DAMAGING | 0 | Deleterious | -3.422 | Probably damaging | 0.992 | 0.70 | 0.97 | Large Decrease | -1.17 | Disease | 1 | 0.542 |
| rs763201540 | GAC ⇒ AAC GAC ⇒ TAC | D53N D53Y | DAMAGING | 0 | Deleterious Deleterious | -3.594 -6.318 | Probably damaging Probably damaging | 1.000 1.000 | 0.00 0.00 | 1.00 1.00 | Large Decrease Large Decrease | -1.07 -0.29 | Disease Disease | 5 7 | \| 0.746 0.872 \|  \| \| --- \| --- \| |
| rs1161818149 | CTG ⇒ CAG CTG ⇒ CCG | L105Q L105P | DAMAGING | 0 | Deleterious Deleterious | -3.537 -4.269 | Probably damaging Probably damaging | 1.000 1.000 | 0.00 0.00 | 1.00 1.00 | Large Decrease Large Decrease | -2.04 -1.69 | Disease Disease | 5 6 | \| 0.752 0.776 \|  \| \| --- \| --- \| |
| rs1260086927 | CAG ⇒ CCG | Q96P | DAMAGING | 0.01 | Deleterious | -3.99 | Probably damaging | 0.998 | 0.27 | 0.99 | Large Decrease | -0.4 | Disease | 2 | 0.585 |
| rs1390270595 | TAC ⇒ TGC | Y51C | DAMAGING | 0.03 | Deleterious | -6.111 | Probably damaging | 1.000 | 0.00 | 1.00 | Large Decrease | -1.29 | Disease | 4 | 0.719 |
| rs1414848134 | GAC ⇒ GTC | D54V | DAMAGING | 0 | Deleterious | -5.845 | Probably damaging | 1.000 | 0.00 | 1.00 | Large Decrease | -1.04 | Disease | 8 | 0.876 |
| rs770412396 | CTG ⇒ CCG | L102P | DAMAGING | 0.01 | Deleterious | -5.377 | Probably damaging | 1.000 | 0.00 | 1.00 | Large Decrease | -1.63 | Disease | 6 | 0.788 |
| **Isoform 3** | | | | | | | | | | | | | | | |
| **SNP rsID** | **Codons** | **Substitution** | **SIFT prediction** | | **PROVEAN prediction** | | **PolyPhen-2 prediction** | | | | **I-Mutant DDG** | | **SNPs&GO prediction** | | |
|  |  |  | **Prediction** | **Score** | **Prediction of** | **Score** | **Prediction** | **Score** | **Sensitivity** | **Specificity** | **SVM3 Prediction Effect** | **DDG Value Prediction** | **Prediction** | **RI** | **Probability** |
| rs555347515 | ATG ⇒ AAG | M29K | DAMAGING | 0 | Deleterious | -3.919 | Probably damaging | 1.000 | 0.00 | 1.00 | Large Decrease | -1.48 | Disease | 5 | 0.759 |
| rs572025435 | AGG ⇒ AGT | R30S | DAMAGING | 0 | Deleterious | -4.195 | Probably damaging | 0.999 | 0.14 | 0.99 | Large Decrease | -1.17 | Disease | 1 | 0.542 |
| rs763201540 | GAC ⇒ AAC GAC ⇒ TAC | D53N D53Y | DAMAGING | 0 | Deleterious  Deleterious | -4.241 -7.643 | Probably damaging Probably damaging | 0.999 1.000 | 0.14 0.00 | 0.99 1.00 | Large Decrease Large Decrease | -1.07 -0.29 | Disease Disease | 5 7 | \| 0.745 0.872 \|  \| \| --- \| --- \| |
| rs1161818149 | CTG ⇒ CAG CTG ⇒ CCG | L105Q L105P | DAMAGING | 0 | Deleterious  Deleterious | -4.694 -5.437 | Probably damaging Probably damaging | 1.000 1.000 | 0.00 0.00 | 1.00 1.00 | Large Decrease Large Decrease | -2.04 -1.69 | Disease Disease | 5 6 | 0.757 0.785 |
| rs1260086927 | CAG ⇒ CCG | Q96P | DAMAGING | 0.01 | Deleterious | -5.297 | Probably damaging | 0.991 | 0.71 | 0.97 | Large Decrease | -0.4 | Disease | 0 | 0.512 |
| rs1390270595 | TAC ⇒ TGC | Y51C | DAMAGING | 0.03 | Deleterious | -7.49 | Probably damaging | 1.000 | 0.00 | 1.00 | Large Decrease | -1.29 | Disease | 4 | 0.716 |
| rs1414848134 | GAC ⇒ GTC | D54V | DAMAGING | 0 | Deleterious | -7.689 | Probably damaging | 1.000 | 0.00 | 1.00 | Large Decrease | -1.04 | Disease | 8 | 0.875 |
| rs770412396 | CTG ⇒ CCG | L102P | DAMAGING | 0.01 | Deleterious | -6.038 | Probably damaging | 1.000 | 0.00 | 1.00 | Large Decrease | -1.63 | Disease | 5 | 0.756 |
| **Isoform 4** | | | | | | | | | | | | | | | |
| **SNP rsID** | **Codons** | **Substitution** | **SIFT prediction** | | **PROVEAN prediction** | | **PolyPhen-2 prediction** | | | | **I-Mutant DDG** | | **SNPs&GO prediction** | | |
|  |  |  | **Prediction** | **Score** | **Prediction of** | **Score** | **Prediction** | **Score** | **Sensitivity** | **Specificity** | **SVM3 Prediction Effect** | **DDG Value Prediction** | **Prediction** | **RI** | **Probability** |
| rs555347515 | ATG ⇒ AAG | M29K | DAMAGING | 0 | Deleterious | -3.905 | Probably damaging | 1.000 | 0.00 | 1.00 | Large Decrease | -1.48 | Disease | 5 | 0.774 |
| rs572025435 | AGG ⇒ AGT | R30S | DAMAGING | 0 | Deleterious | -4.169 | Probably damaging | 1.000 | 0.00 | 1.00 | Large Decrease | -1.17 | Disease | 1 | 0.543 |
| rs763201540 | GAC ⇒ AAC GAC ⇒ TAC | D53N D53Y | DAMAGING | 0 | Deleterious  Deleterious | -4.148 -7.492 | Probably damaging Probably damaging | 1.000 1.000 | 0.00 0.00 | 1.00 1.00 | Large Decrease Large Decrease | -1.07 -0.29 | Disease Disease | 5 7 | 0.735 0.871 |
| rs1161818149 | CTG ⇒ CAG CTG ⇒ CCG | L105Q L105P | DAMAGING | 0 | Deleterious Deleterious | -4.668 -5.391 | Probably damaging Probably damaging | 1.000 0.989 | 0.00 0.72 | 1.00 0.97 | Large Decrease Large Decrease | -2.04 -1.69 | Disease Disease | 5 6 | 0.761 0.794 |
| rs1260086927 | CAG ⇒ CCG | Q96P | DAMAGING | 0.01 | Deleterious | -5.215 | Probably damaging | 1.000 | 0.00 | 1.00 | Large Decrease | -0.4 | Disease | 0 | 0.522 |
| rs1390270595 | TAC ⇒ TGC | Y51C | DAMAGING | 0.03 | Deleterious | -7.307 | Probably damaging | 1.000 | 0.00 | 1.00 | Large Decrease | -1.29 | Disease | 4 | 0.719 |
| rs1414848134 | GAC ⇒ GTC | D54V | DAMAGING | 0 | Deleterious | -7.315 | Probably damaging | 0.982 | 0.75 | 0.96 | Large Decrease | -1.04 | Disease | 7 | 0.874 |
| rs770412396 | CTG ⇒ CCG | L102P | DAMAGING | 0.01 | Deleterious | -6.454 | Probably damaging | 1.000 | 0.00 | 1.00 | Large Decrease | -1.63 | Disease | 5 | 0.771 |

**Supplementary Table 5.** SNPs analyzed in all membrane-bound HLA-G isoforms (HLA-G1-4) by PhD-SNP, SNAP2, MUpro

| **Isoform 1** | | | | | | | | | | |
| --- | --- | --- | --- | --- | --- | --- | --- | --- | --- | --- |
| **SNP rsID** | **Codons** | **Substitution** | **PhD-SNP** | | **SNAP2** | | | **MUpro** | | |
|  |  |  | **Prediction** | **Score** | **Prediction** | **Score** | **Expected Accuracy** | **Prediction** | | **DDG Value** |
| rs555347515 | ATG ⇒ AAG | M29K | Disease | 6 | effect | 25 | 63% | DECREASE | | -1.7776487 |
| rs572025435 | AGG ⇒ AGT | R30S | Disease | 4 | effect | 2 | 53% | DECREASE | | -1.582361 |
| rs763201540 | GAC ⇒ AAC GAC ⇒ TAC | D53N D53Y | Disease Disease | 5 9 | effect effect | 52 84 | 75% 91% | DECREASE DECREASE | | -0.79149414 -0.45294328 |
| rs1161818149 | CTG ⇒ CAG CTG ⇒ CCG | L105Q L105P | Disease Disease | 3 6 | effect effect | 70 76 | 85% 85% | DECREASE DECREASE | | -1.8766335 -2.1510154 |
| rs1260086927 | CAG ⇒ CCG | Q96P | Disease | 6 | effect | 43 | 71% | DECREASE | | -0.97929591 |
| rs1390270595 | TAC ⇒ TGC | Y51C | Disease | 2 | effect | 39 | 66% | DECREASE | | -0.83530596 |
| rs1414848134 | GAC ⇒ GTC | D54V | Disease | 5 | effect | 75 | 85% | DECREASE | | -0.37383825 |
| rs770412396 | CTG ⇒ CCG | L102P | Disease | 7 | effect | 79 | 85% | DECREASE | | -2.238046 |
| **Isoform 2** | | | | | | | | | | |
| **SNP rsID** | **Codons** | **Substitution** | **PhD-SNP prediction** | | **SNAP2** | | | **MUpro** | | |
|  |  |  | **Prediction** | **PI** | **Prediction** | **Score** | **Expected Accuracy** | **Prediction** | | **DDG Value** |
| rs555347515 | ATG ⇒ AAG | M29K | Disease | 3 | effect | 78 | 85% | DECREASE | | -1.7776487 |
| rs572025435 | AGG ⇒ AGT | R30S | Disease | 7 | effect | 65 | 80% | DECREASE | | -1.582361 |
| rs763201540 | GAC ⇒ AAC GAC ⇒ TAC | D53N D53Y | Disease Disease | 7 8 | effect effect | 51 78 | 75% 85% | DECREASE DECREASE | | -0.79149414 -0.45294328 |
| rs1161818149 | CTG ⇒ CAG CTG ⇒ CCG | L105Q L105P | Disease Disease | 4 7 | effect effect | 3 42 | 53% 71% | DECREASE DECREASE | | -1.8766335 -2.1510154 |
| rs1260086927 | CAG ⇒ CCG | Q96P | Disease | 5 | effect | 41 | 71% | DECREASE | | -0.97929591 |
| rs1390270595 | TAC ⇒ TGC | Y51C | Disease | 2 | effect | 34 | 66% | DECREASE | | -0.83530596 |
| rs1414848134 | GAC ⇒ GTC | D54V | Disease | 7 | effect | 56 | 75% | DECREASE | | -0.37383825 |
| rs770412396 | CTG ⇒ CCG | L102P | Disease | 7 | effect | 52 | 75% | DECREASE | | -2.238046 |
| **Isoform 3** | | | | | | | | | | |
| **SNP rsID** | **Codons** | **Substitution** | **PhD-SNP prediction** | | **SNAP2** | | | **MUpro** | | |
|  |  |  | **Prediction** | **PI** | **Prediction** | **Score** | **Expected Accuracy** | **Prediction** | **DDG Value** | |
| rs555347515 | ATG ⇒ AAG | M29K | Disease | 4 | effect | 71 | 85% | DECREASE | -1.7776487 | |
| rs572025435 | AGG ⇒ AGT | R30S | Disease | 7 | effect | 51 | 75% | DECREASE | -1.582361 | |
| rs763201540 | GAC ⇒ AAC GAC ⇒ TAC | D53N D53Y | Disease Disease | 7 6 | effect effect | 70 84 | 85% 91% | DECREASE DECREASE | -0.79149414 -0.45294328 | |
| rs1161818149 | CTG ⇒ CAG CTG ⇒ CCG | L105Q L105P | Disease Disease | 6 7 | effect effect | 66 81 | 80% 91% | DECREASE DECREASE | -1.8766335 -2.1510154 | |
| rs1260086927 | CAG ⇒ CCG | Q96P | Disease | 6 | effect | 45 | 71% | DECREASE | -0.9792959 | |
| rs1390270595 | TAC ⇒ TGC | Y51C | Disease | 6 | effect | 42 | 71% | DECREASE | -0.83530596 | |
| rs1414848134 | GAC ⇒ GTC | D54V | Disease | 7 | effect | 63 | 80% | DECREASE | -0.37383825 | |
| rs770412396 | CTG ⇒ CCG | L102P | Disease | 6 | effect | 78 | 85% | DECREASE | -2.238046 | |
| **Isoform 4** | | | | | | | | | | |
| **SNP rsID** | **Codons** | **Substitution** | **PhD-SNP prediction** | | **SNAP2** | | | **MUpro** | | |
|  |  |  | **Prediction** | **Score** | **Prediction** | **Score** | **Expected Accuracy** | **Prediction** | **DDG Value** | |
| rs555347515 | ATG ⇒ AAG | M29K | Disease | 5 | effect | 81 | 91% | DECREASE | -1.7776487 | |
| rs572025435 | AGG ⇒ AGT | R30S | Disease | 5 | effect | 45 | 71% | DECREASE | -1.582361 | |
| rs763201540 | GAC ⇒ AAC GAC ⇒ TAC | D53N D53Y | Disease Disease | 5 9 | effect effect | 66 83 | 80% 91% | DECREASE DECREASE | -0.79149414 -0.45294328 | |
| rs1161818149 | CTG ⇒ CAG CTG ⇒ CCG | L105Q L105P | Disease Disease | 3 7 | effect effect | 44 59 | 71% 75% | DECREASE DECREASE | -1.8766335 -2.1510154 | |
| rs1260086927 | CAG ⇒ CCG | Q96P | Disease | 6 | effect | 50 | 75% | DECREASE | -0.97929591 | |
| rs1390270595 | TAC ⇒ TGC | Y51C | Disease | 2 | effect | 12 | 59% | DECREASE | -0.83530596 | |
| rs1414848134 | GAC ⇒ GTC | D54V | Disease | 5 | effect | 63 | 80% | DECREASE | -0.37383825 | |
| rs770412396 | CTG ⇒ CCG | L102P | Disease | 7 | effect | 58 | 75% | DECREASE | -2.238046 | |

**Supplementary Table 6.** SNPs analyzed in all soluble HLA-G isoforms (HLA-G5-7) by SIFT, PROVEAN, Polyphen 2.0, I-mutant 3.0, SNPs&GO

| **Isoform 5** | | | | | | | | | | | | | | | |
| --- | --- | --- | --- | --- | --- | --- | --- | --- | --- | --- | --- | --- | --- | --- | --- |
| **SNP rsID** | **Codons** | **Substitution** | **SIFT prediction** | | **PROVEAN prediction** | | **PolyPhen-2 prediction** | | | | **I-Mutant DDG** | | **SNPs&GO prediction** | | |
|  |  |  | **Prediction** | **Score** | **Prediction of** | **Score** | **Prediction** | **Score** | **Sensitivity** | **Specificity** | **SVM3 Prediction Effect** | **DDG Value Prediction** | **Prediction** | **RI** | **Probability** |
| rs540632198 | TTC ⇒ TGC | F32C | DAMAGING | 0 | Deleterious | -5.85 | Probably damaging | 1.000 | 0.00 | 1.00 | Large Decrease | -1.46 | Disease | 1 | 0.529 |
| rs763201540 | GAC ⇒ AAC GAC ⇒ TAC | D53N D53Y | DAMAGING | 0 | Deleterious  Deleterious | -3.905 -7.055 | Probably damaging Probably damaging | 1.000 1.000 | 0.00 0.00 | 1.00 1.00 | Large Decrease Large Decrease | -1.07 -0.29 | Disease Disease | 5 7 | \| 0.726 0.871 \|  \| \| --- \| --- \| |
| rs770412396 | CTG ⇒ CCG | L102P | DAMAGING | 0.01 | Deleterious | -6.241 | Probably damaging | 1.000 | 0.00 | 1.00 | Large Decrease | -1.63 | Disease | 6 | 0.786 |
| rs1260086927 | CAG ⇒ CCG | Q96P | DAMAGING | 0.01 | Deleterious | -5.005 | Probably damaging | 0.993 | 0.70 | 0.97 | Large Decrease | -0.4 | Disease | 0 | 0.523 |
| rs1390270595 | TAC ⇒ TGC | Y51C | DAMAGING | 0.03 | Deleterious | -6.87 | Probably damaging | 1.000 | 0.00 | 1.00 | Large Decrease | -1.29 | Disease | 4 | 0.723 |
| rs1414848134 | GAC ⇒ GTC | D54V | DAMAGING | 0 | Deleterious | -6.866 | Probably damaging | 0.995 | 0.68 | 0.97 | Large Decrease | -1.04 | Disease | 7 | 0.874 |
| rs555347515 | ATG ⇒ AAG | M29K | DAMAGING | 0 | Deleterious | -3.594 | Probably damaging | 0.998 | 0.27 | 0.99 | Large Decrease | -1.48 | Disease | 5 | 0.774 |
| rs1161818149 | CTG ⇒ CCG | L105P | DAMAGING | 0 | Deleterious | -5.34 | Probably damaging | 1.000 | 0.00 | 1.00 | Large Decrease | -1.69 | Disease | 6 | 0.799 |
| **Isoform 6** | | | | | | | | | | | | | | | |
| **SNP rsID** | **Codons** | **Substitution** | **SIFT prediction** | | **PROVEAN prediction** | | **PolyPhen-2 prediction** | | | | **I-Mutant DDG** | | **SNPs&GO prediction** | | |
|  |  |  | **Prediction** | **Score** | **Prediction of** | **Score** | **Prediction** | **Score** | **Sensitivity** | **Specificity** | **SVM3 Prediction Effect** | **DDG Value Prediction** | **Prediction** | **RI** | **Probability** |
| rs540632198 | TTC ⇒ TGC | F32C | DAMAGING | 0 | Deleterious | -5.162 | Probably damaging | 1.000 | 0.00 | 1.00 | Large Decrease | -1.46 | Disease | 1 | 0.543 |
| rs763201540 | GAC ⇒ AAC GAC ⇒ TAC | D53N D53Y | DAMAGING | 0 | Deleterious Deleterious | -3.759 -6.741 | Probably damaging Probably damaging | 1.000 1.000 | 0.00 0.00 | 1.00 1.00 | Large Decrease Large Decrease | -1.07  -0.29 | Disease Disease | 5 7 | 0.744 0.872 |
| rs770412396 | CTG ⇒ CCG | L102P | DAMAGING | 0.01 | Deleterious | -5.513 | Probably damaging | 1.000 | 0.00 | 1.00 | Large Decrease | -1.63 | Disease | 6 | 0.791 |
| rs1260086927 | CAG ⇒ CCG | Q96P | DAMAGING | 0.01 | Deleterious | -4.191 | Probably damaging | 0.997 | 0.41 | 0.98 | Large Decrease | -0.4 | Disease | 2 | 0.587 |
| rs1390270595 | TAC ⇒ TGC | Y51C | DAMAGING | 0.03 | Deleterious | -7.307 | Probably damaging | 1.000 | 0.00 | 1.00 | Large Decrease | -1.29 | Disease | 4 | 0.719 |
| rs1414848134 | GAC ⇒ GTC | D54V | DAMAGING | 0 | Deleterious | -5.82 | Probably damaging | 0.986 | 0.74 | 0.96 | Large Decrease | -1.04 | Disease | 8 | 0.876 |
| rs555347515 | ATG ⇒ AAG | M29K | DAMAGING | 0 | Deleterious | -3.61 | Possibly damaging | 0.823 | 0.84 | 0.93 | Large Decrease | -1.48 | Disease | 5 | 0.756 |
| rs1161818149 | CTG ⇒ CCG | L105P | DAMAGING | 0 | Deleterious | -4.428 | Probably damaging | 1.000 | 0.00 | 1.00 | Large Decrease | -1.69 | Disease | 6 | 0.775 |
| **Isoform 7** | | | | | | | | | | | | | | | |
| **SNP rsID** | **Codons** | **Substitution** | **SIFT prediction** | | **PROVEAN prediction** | | **PolyPhen-2 prediction** | | | | **I-Mutant DDG** | | **SNPs&GO prediction** | | |
|  |  |  | **Prediction** | **Score** | **Prediction of** | **Score** | **Prediction** | **Score** | **Sensitivity** | **Specificity** | **SVM3 Prediction Effect** | **DDG Value Prediction** | **Prediction** | **RI** | **Probability** |
| rs540632198 | TTC ⇒ TGC | F32C | DAMAGING | 0 | Deleterious | -6.383 | Probably damaging | 1.000 | 0.00 | 1.00 | Large Decrease | -1.46 | Disease | 0 | 0.506 |
| rs763201540 | GAC ⇒ AAC GAC ⇒ TAC | D53N D53Y | DAMAGING | 0 | Deleterious  Deleterious | -4.404 -7.928 | Probably damaging Probably damaging | 1.000 1.000 | 0.00 0.00 | 1.00 1.00 | Large Decrease Large Decrease | -1.07 -0.29 | Disease Disease | 5 7 | \| 0.746 0.872 \|  \| \| --- \| --- \| |
| rs770412396 | CTG ⇒ CCG | L102P | DAMAGING | 0.01 | Deleterious | -6.383 | Probably damaging | 1.000 | 0.00 | 1.00 | Large Decrease | -1.63 | Disease | 5 | 0.756 |
| rs1260086927 | CAG ⇒ CCG | Q96P | DAMAGING | 0.01 | Deleterious | -5.235 | Probably damaging | 0.996 | 0.55 | 0.98 | Large Decrease | -0.4 | Disease | 0 | 0.512 |
| rs1390270595 | TAC ⇒ TGC | Y51C | DAMAGING | 0.03 | Deleterious | -7.733 | Probably damaging | 0.996 | 0.55 | 0.98 | Large Decrease | -1.29 | Disease | 4 | 0.714 |
| rs1414848134 | GAC ⇒ GTC | D54V | DAMAGING | 0 | Deleterious | -7.751 | Probably damaging | 0.986 | 0.74 | 0.96 | Large Decrease | -1.04 | Disease | 8 | 0.875 |
| rs555347515 | ATG ⇒ AAG | M29K | DAMAGING | 0 | Deleterious | -4.076 | Possibly damaging | 0.584 | 0.88 | 0.91 | Large Decrease | -1.48 | Disease | 5 | 0.760 |
| rs1161818149 | CTG ⇒ CCG | L105P | DAMAGING | 0 | Deleterious | -5.565 | Probably damaging | 0.993 | 0.70 | 0.97 | Large Decrease | -1.69 | Disease | 5 | 0.786 |

**Supplementary Table 7.** SNPs analyzed in all soluble HLA-G isoforms (HLA-G5-7) by PhD-SNP, SNAP2, MUpro

| **Isoform 5** | | | | | | | | | |
| --- | --- | --- | --- | --- | --- | --- | --- | --- | --- |
| **SNP rsID** | **Codons** | **Substitution** | **PhD-SNP prediction** | | **SNAP2** | | | **MUpro** | |
|  |  |  | **Prediction** | **Score** | **Prediction** | **Score** | **Expected Accuracy** | **Prediction** | **DDG Value** |
| rs540632198 | TTC ⇒ TGC | F32C | Disease | 0 | effect | 23 | 63% | DECREASE | -0.87381309 |
| rs763201540 | GAC ⇒ AAC GAC ⇒ TAC | D53N D53Y | Disease Disease | 5 9 | effect effect | 47 83 | 71% 91% | DECREASE DECREASE | -0.79149414 -0.45294328 |
| rs770412396 | CTG ⇒ CCG | L102P | Disease | 7 | effect | 72 | 85% | DECREASE | -2.238046 |
| rs1260086927 | CAG ⇒ CCG | Q96P | Disease | 6 | effect | 50 | 75% | DECREASE | -0.97929591 |
| rs1390270595 | TAC ⇒ TGC | Y51C | Disease | 2 | effect | 1 | 53% | DECREASE | -0.83530596 |
| rs1414848134 | GAC ⇒ GTC | D54V | Disease | 5 | effect | 69 | 80% | DECREASE | -0.37383825 |
| rs555347515 | ATG ⇒ AAG | M29K | Disease | 6 | effect | 80 | 91% | DECREASE | -1.7776487 |
| rs1161818149 | CTG ⇒ CCG | L105P | Disease | 6 | effect | 67 | 80% | DECREASE | -2.1510154 |
| **Isoform 6** | | | | | | | | | |
| **SNP rsID** | **Codons** | **Substitution** | **PhD-SNP prediction** | | **SNAP2** | | | **MUpro** | |
|  |  |  | **Prediction** | **RI** | **Prediction** | **Score** | **Expected Accuracy** | **Prediction of**  stability | **DDG Value** |
| rs540632198 | TTC ⇒ TGC | F32C | Disease | 3 | effect | 50 | 75% | DECREASE | -0.87381309 |
| rs763201540 | GAC ⇒ AAC GAC ⇒ TAC | D53N D53Y | Disease Disease | 7 8 | effect effect | 47 77 | 71% 85% | DECREASE DECREASE | -0.79149414 -0.45294328 |
| rs770412396 | CTG ⇒ CCG | L102P | Disease | 7 | effect | 57 | 75% | DECREASE | -2.238046 |
| rs1260086927 | CAG ⇒ CCG | Q96P | Disease | 5 | effect | 55 | 75% | DECREASE | -0.97929591 |
| rs1390270595 | TAC ⇒ TGC | Y51C | Disease | 2 | effect | 18 | 59% | DECREASE | -0.83530596 |
| rs1414848134 | GAC ⇒ GTC | D54V | Disease | 7 | effect | 65 | 80% | DECREASE | -0.37383825 |
| rs555347515 | ATG ⇒ AAG | M29K | Disease | 3 | effect | 83 | 91% | DECREASE | -1.7776487 |
| rs1161818149 | CTG ⇒ CCG | L105P | Disease | 7 | effect | 29 | 63% | DECREASE | -2.1510154 |
| **Isoform 7** | | | | | | | | | |
| **SNP rsID** | **Codons** | **Substitution** | **PhD-SNP prediction** | | **SNAP2** | | | **MUpro** | |
|  |  |  | **Prediction** | **RI** | **Prediction** | **Score** | **Expected Accuracy** | **Prediction of**  stability | **DDG Value** |
| rs540632198 | TTC ⇒ TGC | F32C | Disease | 7 | effect | 54 | 75% | DECREASE | -0.87381309 |
| rs763201540 | GAC ⇒ AAC GAC ⇒ TAC | D53N D53Y | Disease Disease | 7 6 | effect effect | 62 83 | 80% 91% | DECREASE DECREASE | -0.79149414 -0.45294328 |
| rs770412396 | CTG ⇒ CCG | L102P | Disease | 6 | effect | 67 | 80% | DECREASE | -2.238046 |
| rs1260086927 | CAG ⇒ CCG | Q96P | Disease | 6 | effect | 54 | 75% | DECREASE | -0.97929591 |
| rs1390270595 | TAC ⇒ TGC | Y51C | Disease | 6 | effect | 31 | 66% | DECREASE | -0.83530596 |
| rs1414848134 | GAC ⇒ GTC | D54V | Disease | 7 | effect | 51 | 75% | DECREASE | -0.37383825 |
| rs555347515 | ATG ⇒ AAG | M29K | Disease | 4 | effect | 82 | 91% | DECREASE | -1.7776487 |
| rs1161818149 | CTG ⇒ CCG | L105P | Disease | 7 | effect | 74 | 85% | DECREASE | -2.1510154 |

**Supplementary Table 8.** Evolutionary conservation pattern of amino acids with solvent accessibility in HLA-G5 by ConSurf server. Conservation score has a range of 1.0 to 9.0. Score 9 represents the most conserved and 1 represents the very variable amino acid. An amino acid, if is preserved and exposed, is a functional residue and if is preserved and buried, is a structural residue

| **Isoform 5** | | | | | | | | | | | |
| --- | --- | --- | --- | --- | --- | --- | --- | --- | --- | --- | --- |
| **conservation score** | **exposed or buried** | | | | **prediction** | **conservation score** | **exposed or buried** | **prediction** | **conservation score** | **exposed or buried** | **prediction** |
| **M29** | | | | | | **E113** | | | **R205** | | |
| 1 (variable) | buried | | | | - | 7 (conserved) | exposed | - | 8 (conserved) | exposed | functional |
| **R30** | | | | | | **H117** | | | **P209** | | |
| 6 (average) | exposed | | | | - | 9 (conserved) | exposed | functional | 9 (conserved) | exposed | functional |
| **F32** | | | | | | **D130** | | | **C227** | | |
| 8 (conserved) | buried | | | | - | 7 (conserved) | exposed |  | 9 (conserved) | buried | structural |
| **P44** | | | | | | **Y142** | | | **A229** | | |
| 9 (conserved) | exposed | | | | functional | 9 (conserved) | exposed | functional | 8(conserved) | buried | - |
| **Y51** | | | | | | **D143** | | | **P234** | | |
| 9 (conserved) | buried | | | | structural | 9 (conserved) | exposed | functional | 9 (conserved) | exposed | functional |
| **D53** | | | | | | **D153** | | | **I237** | | |
| 9 (conserved) | exposed | | | | functional | 9 (conserved) | exposed | functional | 9 (conserved) | buried | structural |
| **D54** | | | | | | **W157** | | | **P259** | | |
| 9 (conserved) | | exposed | | functional | | 9 (conserved) | buried | structural | 9 (conserved) | exposed | functional |
| **Q96** | | | | | | **T158** | | | **Q266** | | |
| 8 (conserved) | | | exposed | functional | | 8 (conserved) | buried | - | 9 (conserved) | exposed | functional |
| **L102** | | | | | | **C188** | | | **H287** | | |
| 9 (conserved) | | | buried | structural | | 9 (conserved) | buried | structural | 9 (conserved) | exposed | functional |
| **L105** | | | | | | **L196** | | | **W298** | | |
| 1 (variable) | | | buried |  | | 8 (conserved) | buried | - | 6 (average) | buried | - |

**The conservation scale:** 1 2 3 4 5 6 7 8 9

**Variable Average Conserved**

**Supplementary Figure 1.** Consurf analysis of HLA-G5. The degree of conservation of amino acids was shown in the colouring scheme. The color intensity increases based on amino acids conservation grades e.g turquoise indicates variable sites; white indicates average sites; maroon indicates evolutionarily conserved sites. The most deleterious predicted SNPs are marked below the sequence as red arrows. e is the exposed residue. b is the buried residue. f is an estimated functional residue (highly conserved and exposed). s is an estimated structural residue (highly conserved and buried).

**Supplementary Table 9.** Evolutionary conservation pattern of the common most deleterious predicted SNPs with their solvent accessibility in HLA-G1-4 by ConSurf server. Conservation score has a range of 1.0 to 9.0. Score 9 represents the most conserved and 1 represents the very variable amino acid. An amino acid, if is preserved and exposed, is a functional residue and an amino acid, if is preserved and buried, is a structural residue

| **Isoform 1** | | | | | | | | **Isoform 2** | | | | | | | | | | | | | | | | | **Isoform 3** | | | **Isoform 4** | | |
| --- | --- | --- | --- | --- | --- | --- | --- | --- | --- | --- | --- | --- | --- | --- | --- | --- | --- | --- | --- | --- | --- | --- | --- | --- | --- | --- | --- | --- | --- | --- |
| **conservation score** | **exposed or buried** | | | **prediction** | | | | **conservation score** | | | | | | **exposed or buried** | | | | | **prediction** | | | | | | **conservation score** | **exposed or buried** | **prediction** | **conservation score** | **exposed or buried** | **prediction** |
| **M29** | | | | | | | | | | | | | | | | | | | | | | | | | | | | | | |
| 1 (variable) | buried | | | - | | | | 1 (variable) | | | | | | buried | | | | | - | | | | | | 3 (variable) | buried | - | 3 (variable) | buried | - |
| **R30** | | | | | | | | | | | | | | | | | | | | | | | | | | | | | | |
| 6 (average) | buried | | | - | | | | 7 (conserved) | | | | | | exposed | | | | | - | | | | | | 7 (conserved) | exposed | - | 7 (conserved) | exposed | - |
| **Y51** | | | | | | | | | | | | | | | | | | | | | | | | | | | | | | |
| 9 (conserved) | exposed | | | functional | | | | | 8 (conserved) | | | | | buried | | | | | | - | | | | | 9 (conserved) | buried | structural | 9 (conserved) | buried | structural |
| **D53** | | | | | | | | | | | | | | | | | | | | | | | | | | | | | | |
| 9 (conserved) | buried | | | structural | | | | | 9 (conserved) | | | | | exposed | | | | | | | functional | | | | 9 (conserved) | exposed | functional | 9 (conserved) | exposed | functional |
| **D54** | | | | | | | | | | | | | | | | | | | | | | | | | | | | | | |
| 8 (conserved) | exposed | | | functional | | | | | | 7 (conserved) | | | | | exposed | | | | | | - | | | | 9 (conserved) | exposed | functional | 9 (conserved) | exposed | functional |
| **Q96** | | | | | | | | | | | | | | | | | | | | | | | | | | | | | | |
| 7 (conserved) | | exposed | | | - | | | | | | 4 (variable) | | | | | exposed | | | | | | - | | | 8 (conserved) | exposed | functional | 8 (conserved) | exposed | functional |
| **L102** | | | | | | | | | | | | | | | | | | | | | | | | | | | | | | |
| 9 (conserved) | | | buried | | | structural | | | | | | 3 (variable) | | | | | buried | | | | | | - | | 9 (conserved) | buried | structural | 8 (conserved) | buried | structural |
| **L105** | | | | | | | | | | | | | | | | | | | | | | | | | | | | | | |
| 1 (variable) | | | buried | | | | - | | | | | | 3 (variable) | | | | | buried | | | | | | - | 1 (variable) | buried | - | 1 (variable) | buried | - |


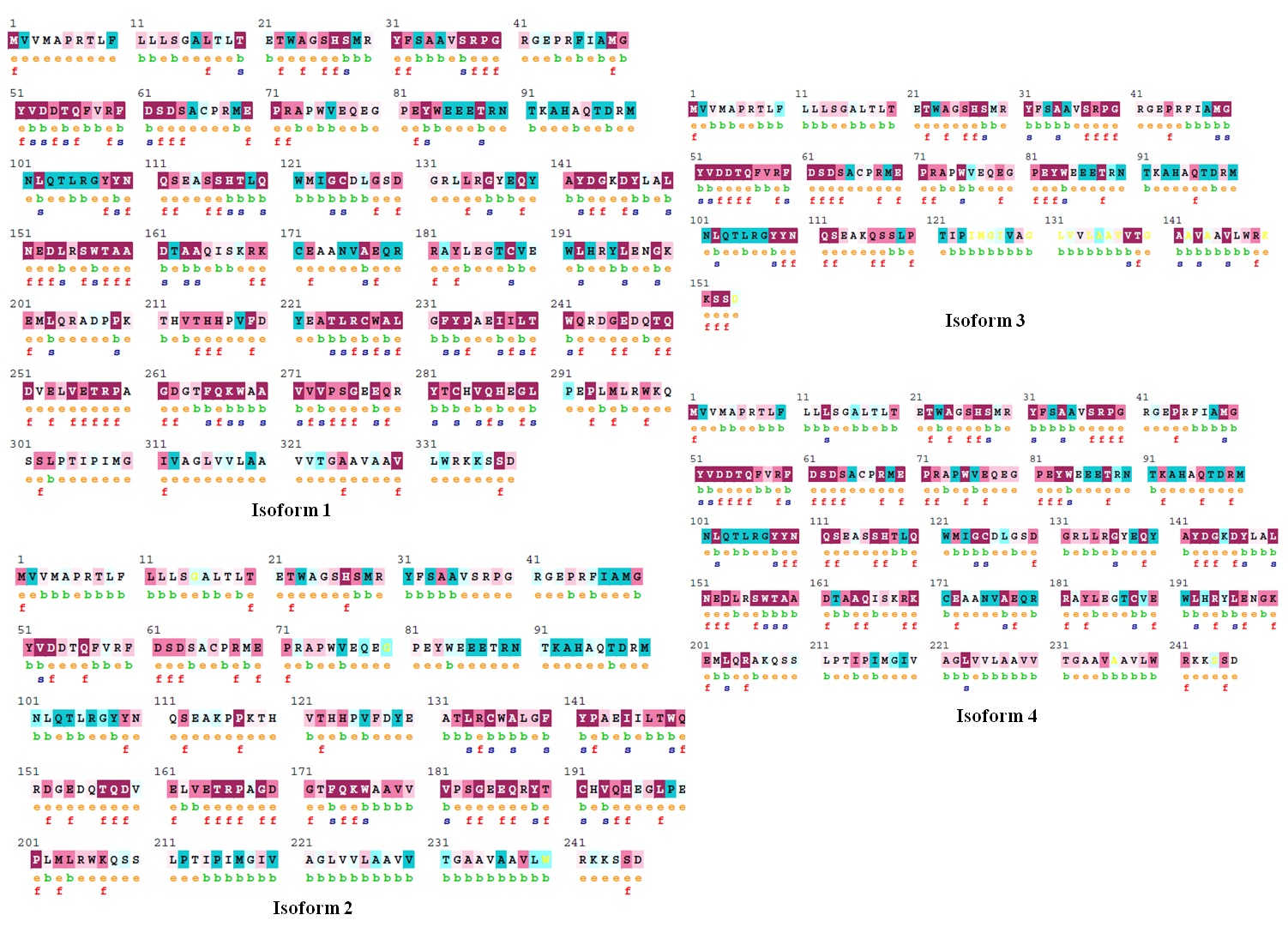


**Supplementary Figure 2.** Consurf analysis of the most deleterious predicted SNPs which is common in HLA-G1-4. The degree of conservation of amino acids was shown in the colouring scheme. The color intensity increases based on amino acids conservation grades e.g turquoise indicates variable sites; white indicates average sites; maroon indicates evolutionarily conserved sites. The most deleterious predicted SNPs are marked below the sequence as red arrows. e is the exposed residue. b is the buried residue. f is an estimated functional residue (highly conserved and exposed). s is an estimated structural residue (highly conserved and buried).

**Supplementary Table 10.** Evolutionary conservation pattern of the common most deleterious predicted SNPs with their solvent accessibility in HLA-G5-7 by ConSurf server. Conservation score has a range of 1.0 to 9.0. Score 9 represents the most conserved and 1 represents the very variable amino acid. An amino acid, if is preserved and exposed, is a functional residue and an amino acid, if is preserved and buried, is a structural residue

| **Isoform 5** | | | | | | | | **Isoform 6** | | | | | | | | | | | | | | | | | **Isoform 7** | | |
| --- | --- | --- | --- | --- | --- | --- | --- | --- | --- | --- | --- | --- | --- | --- | --- | --- | --- | --- | --- | --- | --- | --- | --- | --- | --- | --- | --- |
| **conservation score** | **exposed or buried** | | | **prediction** | | | | **conservation score** | | | | | | **exposed or buried** | | | | | **prediction** | | | | | | **conservation score** | **exposed or buried** | **prediction** |
| **M29** | | | | | | | | | | | | | | | | | | | | | | | | | | | |
| 1 (variable) | buried | | | - | | | | 1 (variable) | | | | | | buried | | | | | - | | | | | | 3 (variable) | buried | - |
| **F32** | | | | | | | | | | | | | | | | | | | | | | | | | | | |
| 8 (conserved) | buried | | | - | | | | 7 (conserved) | | | | | | buried | | | | | - | | | | | | 7 (conserved) | buried | - |
| **Y51** | | | | | | | | | | | | | | | | | | | | | | | | | | | |
| 9 (conserved) | buried | | | structural | | | | | 9 (conserved) | | | | | buried | | | | | | - | | | | | 9 (conserved) | buried | structural |
| **D53** | | | | | | | | | | | | | | | | | | | | | | | | | | | |
| 9 (conserved) | exposed | | | functional | | | | | 9 (conserved) | | | | | exposed | | | | | | | functional | | | | 9 (conserved) | exposed | functional |
| **D54** | | | | | | | | | | | | | | | | | | | | | | | | | | | |
| 9 (conserved) | exposed | | | functional | | | | | | 7 (conserved) | | | | | exposed | | | | | | - | | | | 9 (conserved) | exposed | functional |
| **Q96** | | | | | | | | | | | | | | | | | | | | | | | | | | | |
| 8 (conserved) | | exposed | | | - | | | | | | 2 (variable) | | | | | exposed | | | | | | - | | | 7 (conserved) | exposed | functional |
| **L102** | | | | | | | | | | | | | | | | | | | | | | | | | | | |
| 9 (conserved) | | | buried | | | structural | | | | | | 1(variable) | | | | | buried | | | | | | - | | 9 (conserved) | buried | structural |
| **L105** | | | | | | | | | | | | | | | | | | | | | | | | | | | |
| 1 (variable) | | | buried | | | | - | | | | | | 4 (variable) | | | | | buried | | | | | | - | 2 (variable) | buried | - |


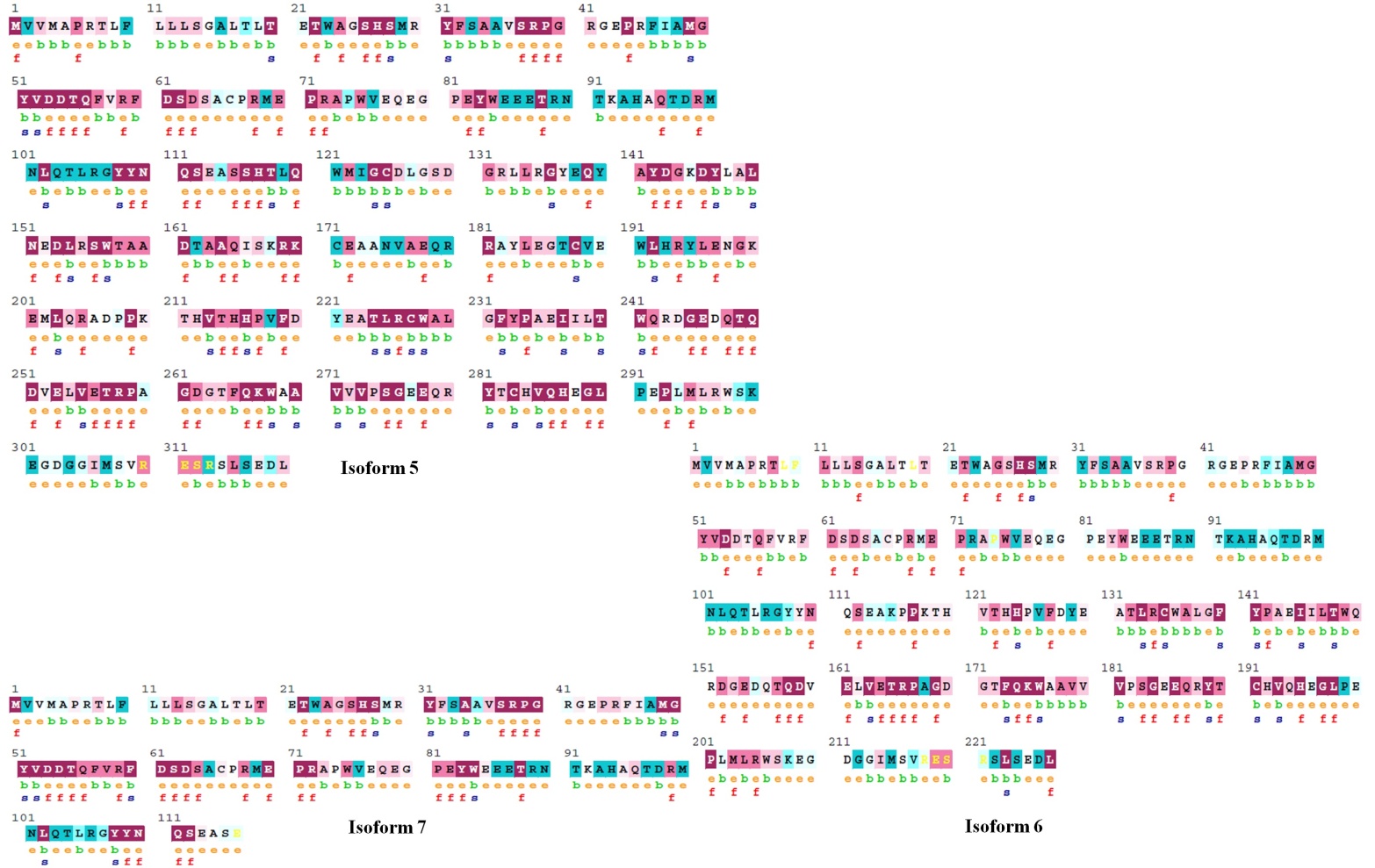


**Supplementary Figure 3.** Consurf analysis of the most deleterious predicted SNPs which is common in HLA-G5-7. The degree of conservation of amino acids was shown in the colouring scheme. The color intensity increases based on amino acids conservation grades e.g turquoise indicates variable sites; white indicates average sites; maroon indicates evolutionarily conserved sites. The most deleterious predicted SNPs are marked below the sequence as red arrows. e is the exposed residue. b is the buried residue. f is an estimated functional residue (highly conserved and exposed). s is an estimated structural residue (highly conserved and buried).

**Supplementary Table 11.** Prediction of functional effects of the most deleterious SNPs on the HLA-G5 by MutPred. The predictions which are very confident hypotheses shown in bold font.

| **Isoform 5 (ID: 473e2e18-9b20-4b9d-b88a-5be8edfb7dde)** | | | | | |
| --- | --- | --- | --- | --- | --- |
| **Substitution** | **Mutpred score**  **(>0.50 is considered pathogenic)** | **Molecular mechanism with p-value <= 0.05** | **Probability** | **P-value** | **prediction** |
| **H117P** | 0.791 | **Altered Metal binding  Altered Transmembrane protein**  Altered Ordered interface | 0.37 0.25 0.24 | 7.7e-03 1.1e-03 0.04 | **very confident hypotheses very confident hypotheses** confident hypotheses |
| **H117L** | 0.622 | Altered Metal binding  Gain of Helix  Loss of Strand  Altered Transmembrane protein Altered Ordered interface | 0.38 0.31 0.29 0.27 0.23 | 7.4e-03 4.4e-03 3.3e-03 5.2e-04 0.05 | actionable hypotheses actionable hypotheses actionable hypotheses actionable hypotheses  actionable hypotheses |
| **D54Y** | 0.684 | Altered Metal binding  Altered Ordered interface  Altered Disordered interface  Gain of Strand  Altered Transmembrane protein  Loss of Proteolytic cleavage at D53  Gain of Pyrrolidone carboxylic acid at Q56  Gain of Sulfation at D54 | 0.47 0.39 0.39 0.26 0.14 0.13 0.09 0.08 | 6.0e-04 1.4e-03 5.7e-03 0.04 0.02 0.03 0.01 3.5e-03 | actionable hypotheses actionable hypotheses actionable hypotheses actionable hypotheses  actionable hypotheses  actionable hypotheses actionable hypotheses actionable hypotheses |
| **P234T** | 0.733 | Altered Ordered interface  Altered Transmembrane protein | 0.32 0.27 | 1.8e-03 6.9e-04 | actionable hypotheses actionable hypotheses |
| **P209A** | 0.134 | - | - | - | - |
| **H287Y** | 0.321 | - | - | - | - |
| **F32C** | 0.763 | **Altered Ordered interface** Loss of Strand  **Loss of Proteolytic cleavage at R30  Altered Metal binding** | 0.28 0.27 0.20 0.12 | 4.4e-03 0.02 2.2e-03 7.3e-03 | **very confident hypotheses** confident hypotheses **very confident hypotheses very confident hypotheses** |
| **R30S** | 0.697 | Altered Ordered interface  Loss of Proteolytic cleavage at R30  Altered Stability  Altered Metal binding | 0.30 0.20 0.19 0.16 | 4.8e-03 2.0e-03 0.01 3.6e-03 | actionable hypotheses actionable hypotheses actionable hypotheses actionable hypotheses |
| **T158P** | 0.638 | Altered Ordered interface  Gain of Relative solvent accessibility  Altered Disordered interface  Loss of Allosteric site at W157  Altered Metal binding  Altered Transmembrane protein  Altered Coiled coil | 0.31 0.29 0.28 0.28 0.27 0.27 0.10 | 3.8e-03 0.01 0.03 7.3e-03 5.2e-03 5.4e-04 0.04 | actionable hypotheses actionable hypotheses actionable hypotheses actionable hypotheses  actionable hypotheses actionable hypotheses actionable hypotheses |
| **Y142C** | 0.625 | Altered Metal binding  Altered Disordered interface  Altered Ordered interface  Altered Transmembrane protein  Gain of Relative solvent accessibility  Loss of Acetylation at K145  Loss of Methylation at K145  Loss of Ubiquitylation at K145  Gain of Disulfide linkage at Y142  Loss of Sulfation at Y147  Loss of Pyrrolidone carboxylic acid at Q139 | 0.74 0.61 0.51 0.29 0.27 0.20 0.18 0.16 0.10 0.09 0.04 | 1.1e-03 1.5e-04 1.7e-04 2.1e-04 0.02 0.04 0.01 0.04 0.05 3.1e-03 0.05 | actionable hypotheses actionable hypotheses actionable hypotheses actionable hypotheses  actionable hypotheses actionable hypotheses actionable hypotheses actionable hypotheses actionable hypotheses actionable hypotheses actionable hypotheses |
| **I237F** | 0.697 | Altered Disordered interface  Altered Transmembrane protein  Altered Ordered interface  Loss of Strand  Loss of Pyrrolidone carboxylic acid at Q242 | 0.33 0.28 0.28 0.27 0.07 | 0.01 4.8e-04 0.05 0.03 0.02 | actionable hypotheses actionable hypotheses actionable hypotheses actionable hypotheses actionable hypotheses |
| **Q266L** | 0.597 | Altered Ordered interface  Loss of Strand  Gain of Relative solvent accessibility  Loss of Methylation at K267  Gain of Acetylation at K267  Loss of Pyrrolidone carboxylic acid at Q266  Altered Metal binding  Altered Transmembrane protein  Gain of Proteolytic cleavage at D262  Gain of Catalytic site at D262 | 0.27 0.26 0.25 0.23 0.22 0.20 0.18 0.15 0.15 0.14 | 0.05 0.05 0.04 3.6e-03 0.03 2.2e-03 0.05 0.01 0.01 0.02 | actionable hypotheses actionable hypotheses actionable hypotheses actionable hypotheses actionable hypotheses actionable hypotheses actionable hypotheses actionable hypotheses  actionable hypotheses actionable hypotheses |
| **D53N** | 0.432 | - | - | - | - |
| **D53Y** | 0.661 | Altered Ordered interface  Altered Disordered interface  Altered Metal binding  Gain of Strand  Altered Transmembrane protein  Loss of Proteolytic cleavage at D53  Gain of Pyrrolidone carboxylic acid at Q56  Gain of Sulfation at D53 | 0.41 0.38 0.37 0.26 0.19 0.13 0.09 0.07 | 7.2e-04 6.6e-03 1.7e-03 0.04 6.0e-03 0.03 0.01 4.6e-03 | actionable hypotheses actionable hypotheses actionable hypotheses actionable hypotheses actionable hypotheses actionable hypotheses actionable hypotheses actionable hypotheses |
| **W298R** | 0.647 | Gain of Intrinsic disorder  Gain of B-factor  Gain of Loop  Gain of Ubiquitylation at K300 | 0.59 0.27 0.27 0.16 | 4.1e-04 0.01 0.04 0.03 | actionable hypotheses actionable hypotheses actionable hypotheses actionable hypotheses |
| **C227Y** | 0.868 | **Altered Disordered interface  Altered Ordered interface  Altered Transmembrane protein**  Altered Metal binding  Loss of Helix | 0.41 0.39 0.31 0.29 0.27 | 3.6e-03 4.0e-04 1.2e-04 0.02 0.04 | **very confident hypotheses very confident hypotheses very confident hypotheses** confident hypotheses confident hypotheses |
| **C227F** | 0.891 | Altered Disordered interface  Altered Metal binding  **Altered Ordered interface  Altered Transmembrane protein**  Loss of Helix | 0.34 0.31 0.28 0.27 0.27 | 0.01 0.01 4.2e-03 7.0e-04 0.05 | confident hypotheses confident hypotheses **very confident hypotheses very confident hypotheses** confident hypotheses |
| **Y142H** | 0.614 | Altered Metal binding  Altered Ordered interface  Altered Disordered interface  Altered Transmembrane protein  Gain of Relative solvent accessibility  Gain of Acetylation at K145  Loss of Methylation at K145  Altered Stability  Loss of Ubiquitylation at K145  Altered Coiled coil  Loss of Sulfation at Y147  Loss of Pyrrolidone carboxylic acid at Q139 | 0.78 0.44 0.37 0.30 0.25 0.20 0.17 0.17 0.15 0.10 0.09 0.04 | 8.3e-04 5.3e-04 7.0e-03 1.4e-04 0.03 0.04 0.01 0.02 0.05 0.04 3.1e-03 0.05 | actionable hypotheses actionable hypotheses actionable hypotheses actionable hypotheses actionable hypotheses actionable hypotheses actionable hypotheses actionable hypotheses actionable hypotheses actionable hypotheses actionable hypotheses actionable hypotheses |
| **C188S** | 0.767 | Altered Disordered interface  Altered Ordered interface  Altered DNA binding  Altered Transmembrane protein | 0.28 0.25 0.15 0.13 | 0.04 0.02 0.04 0.02 | confident hypotheses confident hypotheses confident hypotheses confident hypotheses |
| **P259H** | 0.518 | Altered Metal binding  Loss of Loop  Altered Ordered interface  Altered Transmembrane protein  Loss of Proteolytic cleavage at D262  Gain of Catalytic site at D262 | 0.44 0.27 0.24 0.21 0.13 0.11 | 6.8e-03 0.03 0.03 3.8e-03 0.02 0.04 | actionable hypotheses actionable hypotheses actionable hypotheses actionable hypotheses actionable hypotheses actionable hypotheses |
| **W157R** | 0.93 | **Altered Transmembrane protein  Altered Ordered interface  Gain of Relative solvent accessibility**  Altered Disordered interface  **Loss of Allosteric site at W157**  Altered Coiled coil  **Altered Metal binding** | 0.37 0.37 0.33 0.30 0.30 0.27 0.26 | 1.5e-05 1.7e-03 2.9e-03 0.02 3.8e-03 0.01 6.5e-03 | **very confident hypotheses very confident hypotheses very confident hypotheses** confident hypotheses **very confident hypotheses** confident hypotheses **very confident hypotheses** |
| **A229D** | 0.778 | **Altered Transmembrane protein**  Gain of Relative solvent accessibility  Altered Metal binding  **Altered Ordered interface** | 0.34 0.28 0.27 0.27 | 2.4e-05 0.02 0.03 9.5e-03 | **very confident hypotheses** confident hypotheses confident hypotheses **very confident hypotheses** |
| **Q96P** | 0.619 | Loss of Helix  Altered Transmembrane protein  Altered DNA binding  Gain of Ubiquitylation at K92  Gain of Proteolytic cleavage at R99  Loss of Pyrrolidone carboxylic acid at Q96 | 0.30 0.28 0.25 0.15 0.13 0.10 | 9.3e-03 4.2e-04 6.6e-03 0.04 0.02 0.01 | actionable hypotheses actionable hypotheses actionable hypotheses actionable hypotheses actionable hypotheses actionable hypotheses |
| **D143N** | 0.599 | Altered Metal binding  Altered Transmembrane protein  Altered Disordered interface  Gain of Relative solvent accessibility  Altered Ordered interface  Loss of Acetylation at K145  Loss of Methylation at K145  Loss of Ubiquitylation at K145  Loss of Sulfation at Y147  Loss of Pyrrolidone carboxylic acid at Q139 | 0.38 0.28 0.28 0.27 0.26 0.19 0.16 0.15 0.10 0.04 | 1.4e-03 3.8e-04 0.04 0.02 0.01 0.05 0.01 0.04 2.9e-03 0.05 | actionable hypotheses actionable hypotheses actionable hypotheses actionable hypotheses actionable hypotheses actionable hypotheses  actionable hypotheses actionable hypotheses actionable hypotheses actionable hypotheses |
| **D143H** | 0.702 | Altered Metal binding  Altered Transmembrane protein  Altered Disordered interface  Altered Ordered interface  Loss of Relative solvent accessibility  Loss of Acetylation at K145  Loss of Methylation at K145  Loss of Ubiquitylation at K145  Loss of Sulfation at Y147  Gain of Pyrrolidone carboxylic acid at Q139 | 0.44 0.30 0.30 0.27 0.26 0.19 0.17 0.16 0.10 0.04 | 6.7e-03 1.5e-04 0.02 6.7e-03 0.03 0.05 0.01 0.03  2.8e-03 0.05 | actionable hypotheses actionable hypotheses actionable hypotheses actionable hypotheses actionable hypotheses actionable hypotheses actionable hypotheses actionable hypotheses actionable hypotheses actionable hypotheses |
| **R205S** | 0.558 | Gain of Intrinsic disorder  Altered Ordered interface | 0.34 0.23 | 0.02 0.05 | actionable hypotheses actionable hypotheses |
| **Y51C** | 0.668 | Altered Disordered interface  Altered Metal binding  Altered Ordered interface  Loss of Strand  Altered Transmembrane protein  Loss of Proteolytic cleavage at D53  Altered Stability  Gain of Pyrrolidone carboxylic acid at Q56  Loss of Sulfation at Y51 | 0.61 0.52 0.30 0.28 0.21 0.13 0.12 0.07 0.03 | 1.6e-04 4.3e-03 4.3e-03 0.01 4.4e-03 0.03 0.03 0.02 0.02 | actionable hypotheses actionable hypotheses actionable hypotheses actionable hypotheses actionable hypotheses actionable hypotheses  actionable hypotheses actionable hypotheses actionable hypotheses |
| **L196P** | 0.878 | Altered Disordered interface  **Altered Ordered interface**  Altered Stability | 0.33 0.27 0.15 | 0.01 8.4e-03 0.02 | confident hypotheses **very confident hypotheses** confident hypotheses |
| **D54V** | 0.638 | Altered Metal binding  Altered Disordered interface  Altered Ordered interface  Altered Transmembrane protein  Loss of Proteolytic cleavage at D53  Gain of Pyrrolidone carboxylic acid at Q56  Loss of Sulfation at Y51 | 0.52 0.40 0.25 0.14 0.12 0.08 0.03 | 3.9e-04 4.6e-03 0.02 0.02 0.03 0.01 0.02 | actionable hypotheses actionable hypotheses actionable hypotheses actionable hypotheses actionable hypotheses actionable hypotheses actionable hypotheses |
| **P234L** | 0.785 | **Altered Ordered interface  Altered Transmembrane protein**  Altered Disordered interface  **Loss of Strand** | 0.32 0.31 0.30 0.28 | 1.5e-03 1.1e-04 0.03 8.6e-03 | **very confident hypotheses very confident hypotheses** confident hypotheses **very confident hypotheses** |
| **R205L** | 0.398 | - | - | - | - |
| **P209R** | 0.237 | - | **-** | **-** | **-** |
| **M29K** | 0.688 | Altered Ordered interface  Loss of Proteolytic cleavage at R30  Altered Stability  Altered Metal binding  Altered Transmembrane protein | 0.26 0.19 0.13 0.11 0.11 | 0.02 3.7e-03 0.03 8.3e-03 0.03 | actionable hypotheses actionable hypotheses actionable hypotheses actionable hypotheses actionable hypotheses |
| **D153G** | 0.832 | **Altered Metal binding  Altered Disordered interface  Loss of Relative solvent accessibility  Gain of Strand  Altered Transmembrane protein**  Altered Ordered interface  **Gain of Allosteric site at W157** Altered Stability  Altered Coiled coil | 0.42 0.36 0.30 0.30 0.28 0.26 0.26 0.19 0.10 | 3.1e-04 8.2e-03 9.9e-03 2.7e-03 3.5e-04 0.01 7.3e-03 0.01 0.04 | **very confident hypotheses very confident hypotheses very confident hypotheses very confident hypotheses very confident hypotheses** confident hypotheses **very confident hypotheses** confident hypotheses confident hypotheses |
| **D130H** | 0.642 | Altered Metal binding  Altered Transmembrane protein  Altered Disordered interface  Loss of Relative solvent accessibility  Altered Ordered interface  Gain of Disulfide linkage at C125 | 0.30 0.29 0.29 0.25 0.24 0.12 | 0.02 2.1e-04 0.03 0.03 0.04 0.04 | actionable hypotheses actionable hypotheses actionable hypotheses actionable hypotheses actionable hypotheses actionable hypotheses |
| **L102P** | 0.667 | Altered Disordered interface  Gain of Intrinsic disorder  Altered Transmembrane protein  Loss of Helix  Altered DNA binding  Altered Stability  Loss of Proteolytic cleavage at R99 | 0.37 0.34 0.31 0.30 0.24 0.21 0.14 | 6.7e-03 0.02 1.1e-04 8.3e-03 0.01 0.01 0.02 | actionable hypotheses actionable hypotheses actionable hypotheses actionable hypotheses actionable hypotheses actionable hypotheses actionable hypotheses |
| **E113V** | 0.388 | - |  |  |  |
| **L105Q** | 0.505 | Altered Disordered interface  Gain of Intrinsic disorder  Altered Transmembrane protein  Altered Ordered interface   Altered Stability  Loss of Proteolytic cleavage at R106  Loss of N-linked glycosylation at N110 | 0.31 0.30 0.29 0.26 0.14 0.11 0.05 | 0.02 0.05 2.1e-04 0.01 0.02 0.05 0.02 | actionable hypotheses actionable hypotheses actionable hypotheses actionable hypotheses actionable hypotheses actionable hypotheses actionable hypotheses |
| **L105P** | 0.688 | Altered Disordered interface  Loss of Helix  Altered Transmembrane protein  Altered Ordered interface  Altered Stability  Loss of Proteolytic cleavage at R106  Gain of N-linked glycosylation at N110 | 0.35 0.30 0.27 0.26 0.19 0.11 0.05 | 9.3e-03 9.2e-03 4.9e-04 0.01 0.01 0.04 0.02 | actionable hypotheses actionable hypotheses actionable hypotheses actionable hypotheses actionable hypotheses actionable hypotheses actionable hypotheses |
| **P44L** | 0.534 | Altered Disordered interface  Altered Transmembrane protein  Altered DNA binding  Gain of Proteolytic cleavage at R45 | 0.32 0.23 0.22 0.17 | 0.01 2.1e-03 0.01 7.0e-03 | actionable hypotheses actionable hypotheses actionable hypotheses actionable hypotheses |

**Supplementary Table 12.** Prediction of functional effects of the most deleterious SNPs on the all membrane-bound HLA-G isoforms (HLA-G1-4) by MutPred. The predictions which are very confident hypotheses shown in bold font.

| **M29K** | | | | | | | | | | | | |
| --- | --- | --- | --- | --- | --- | --- | --- | --- | --- | --- | --- | --- |
| **Isoform 1** | | | **Isoform 2** | | | **Isoform 3** | | | | **Isoform 4** | | |
| **Mutpred score** | **Molecular mechanism** | **P-value** | **Mutpred score** | **Molecular mechanism** | **P-value** | **Mutpred score** | | **Molecular mechanism** | **P-value** | **Mutpred score** | **Molecular  mechanism** | **P-value** |
| 0.720 | Altered Ordered interface  Loss of Proteolytic cleavage at R30  Altered Stability  Altered Transmembrane protein Altered Metal binding | 0.02 2.6e-03  0.03 0.03 7.9e-03 | 0.523 | Loss of Loop  Altered Stability  Altered Ordered interface  Altered Transmembrane protein  Altered Metal binding | 0.03 7.5e-03 0.02 0.02  0.01 | 0.793 | | Altered Ordered interface  **Loss of Proteolytic cleavage at R30**  Altered Transmembrane protein  **Altered Stability  Altered Metal binding** | 0.02 3.8e-03  0.01  0.02 3.5e-03 | 0.754 | Altered Ordered interface  Loss of Proteolytic cleavage at R30 Altered Stability  Altered Metal binding  Altered Transmembrane protein | 0.02 3.5e-03  0.03 8.2e-03 0.03 |
| **R30S** | | | | | | | | | | | | |
| **Isoform 1** | | | **Isoform 2** | | | **Isoform 3** | | | | **Isoform 4** | | |
| **Mutpred score** | **Molecular mechanism** | **P-value** | **Mutpred score** | **Molecular mechanism** | **P-value** | **Mutpred score** | | **Molecular mechanism** | **P-value** | **Mutpred score** | **Molecular  mechanism** | **P-value** |
| 0.792 | **Altered Ordered interface Loss of Proteolytic cleavage at R30** Altered Stability **Altered Metal binding** | 5.0e-03 1.2e-03  0.01 3.5e-03 | 0.475 | **-** | - | 0.78 | | **Altered Ordered interface  Altered Stability  Altered Metal binding  Loss of Proteolytic cleavage at R30** Altered Transmembrane protein | 6.6e-03 9.6e-03 1.2e-03 1.9e-03  0.03 | 0.776 | **Altered Ordered interface  Loss of Proteolytic cleavage at R30** Altered Stability  **Altered Metal binding** | 4.9e-03 1.8e-03  0.01 3.5e-03 |
| **Y51C** | | | | | | | | | | | | |
| **Isoform 1** | | | **Isoform 2** | | | **Isoform 3** | | | | **Isoform 4** | | |
| **Mutpred score** | **Molecular mechanism** | **P-value** | **Mutpred score** | **Molecular mechanism** | **P-value** | **Mutpred score** | | **Molecular mechanism** | **P-value** | **Mutpred score** | **Molecular  mechanism** | **P-value** |
| 0.715 | Altered Disordered interface  Altered Metal binding  Altered Ordered interface  Loss of Strand  Altered Transmembrane protein  Loss of Proteolytic cleavage at D53  Altered Stability  Gain of Pyrrolidone carboxylic acid at Q56  Loss of Sulfation at Y51 | 1.9e-04 4.1e-03 4.6e-03 0.02 5.0e-03 0.03  0.03 0.02  0.02 | 0.611 | Loss of Helix  Gain of Relative solvent accessibility  Altered Transmembrane protein | 3.0e-03 0.02 1.5e-03 | 0.766 | | **Altered Disordered interface**  **Altered Metal binding**  Altered Ordered interface  Loss of Strand  **Altered Transmembrane protein**  Loss of Proteolytic cleavage at D53  Altered Stability  Gain of Pyrrolidone carboxylic acid at Q56  Loss of Sulfation at Y51 | 2.7e-04  3.4e-03 0.02 0.02 1.2e-03  0.03  0.03 0.02  0.02 | 0.698 | Altered Disordered interface  Altered Metal binding  Altered Ordered interface  Loss of Strand  Altered Transmembrane protein  Loss of Proteolytic cleavage at D53  Altered Stability  Gain of Pyrrolidone carboxylic acid at Q56  Loss of Sulfation at Y51 | 1.7e-04 3.7e-03 4.5e-03 0.01 5.8e-03  0.03  0.03 0.02  0.02 |
| **D53N** | | | | | | | | | | | | |
| **Isoform 1** | | | **Isoform 2** | | | **Isoform 3** | | | | **Isoform 4** | | |
| **Mutpred score** | **Molecular mechanism** | **P-value** | **Mutpred score** | **Molecular mechanism** | **P-value** | **Mutpred score** | | **Molecular mechanism** | **P-value** | **Mutpred score** | **Molecular  mechanism** | **P-value** |
| 0.486 | - | - | 0.628 | Altered Metal binding Altered Disordered interface  Gain of Strand  Gain of Relative solvent accessibility  Altered Transmembrane protein  Loss of Proteolytic cleavage at D53 Gain of N-linked glycosylation at D53 Loss of Sulfation at Y51 | 7.2e-03 0.01  0.02 0.03  2.8e-03  1.5e-03  8.8e-03  0.02 | 0.593 | | Altered Metal binding  Altered Disordered interface Altered Ordered interface  Altered Transmembrane protein  Loss of Proteolytic cleavage at D53  Loss of Pyrrolidone carboxylic acid at Q56  Gain of N-linked glycosylation at D53  Loss of Sulfation at Y51 | 1.6e-03 0.01 0.03 3.6e-03  0.03  0.02  0.02  0.02 | 0.508 | Altered Metal binding  Altered Disordered interface  Altered Ordered interface  Altered Transmembrane protein  Loss of Proteolytic cleavage at D53 ( Loss of Pyrrolidone carboxylic acid at Q56  Gain of N-linked glycosylation at D53  Loss of Sulfation at Y51 | 9.5e-04 0.01 0.04 9.1e-03  0.03  0.02  0.02  0.02 |
| **D53Y** | | | | | | | | | | | | |
| **Isoform 1** | | | **Isoform 2** | | | **Isoform 3** | | | | **Isoform 4** | | |
| **Mutpred score** | **Molecular mechanism** | **P-value** | **Mutpred score** | **Molecular mechanism** | **P-value** | **Mutpred score** | | **Molecular mechanism** | **P-value** | **Mutpred score** | **Molecular  mechanism** | **P-value** |
| 0.746 | Altered Ordered interface  Altered Disordered interface  Altered Metal binding  Altered Transmembrane protein  Loss of Proteolytic cleavage at D53  Gain of Pyrrolidone carboxylic acid at Q56  Gain of Sulfation at D53 | 1.1e-03 6.6e-03 1.7e-03 6.6e-03 0.03  0.01  4.4e-03 | 0.628 | Altered Metal binding  Altered Disordered interface  Gain of Strand  Gain of Relative solvent accessibility  Altered Transmembrane protein  Loss of Proteolytic cleavage at D53  Gain of N-linked glycosylation at D53  Loss of Sulfation at Y51 | 7.2e-03 0.01  0.02 0.03  2.8e-03  1.5e-03  8.8e-03  0.02 | 0.785 | | **Altered Ordered interface**  **Altered Disordered interface**  **Altered Metal binding  Altered Transmembrane protein** Loss of Proteolytic cleavage at D53  Gain of Pyrrolidone carboxylic acid at Q56  **Gain of Sulfation at D53** | 4.3e-04 9.1e-03  2.0e-03 2.3e-03  0.03  0.01  5.1e-03 | 0.75 | Altered Ordered interface  Altered Metal binding  Altered Disordered interface  Gain of Strand  Altered Transmembrane protein  Loss of Proteolytic cleavage at D53  Gain of Pyrrolidone carboxylic acid at Q56  Gain of Sulfation at D53 | 5.5e-04 1.6e-03 7.3e-03 0.04 7.4e-03  0.03  0.01  4.6e-03 |
| **D54V** | | | | | | | | | | | | |
| **Isoform 1** | | | **Isoform 2** | | | **Isoform 3** | | | | **Isoform 4** | | |
| **Mutpred score** | **Molecular mechanism** | **P-value** | **Mutpred score** | **Molecular mechanism** | **P-value** | **Mutpred score** | | **Molecular mechanism** | **P-value** | **Mutpred score** | **Molecular  mechanism** | **P-value** |
| 0.718 | Altered Metal binding  Altered Disordered interface  Altered Ordered interface  Altered Transmembrane protein  Loss of Proteolytic cleavage at D53  Gain of Pyrrolidone carboxylic acid at Q56  Loss of Sulfation at Y51 | 3.1e-04 4.2e-03 0.02 0.02 0.03  0.01  0.02 | 0.681 | Altered Disordered interface  Altered Metal binding  Altered Transmembrane protein  Loss of Strand  Loss of Proteolytic cleavage at D53  Loss of Sulfation at Y51 | 1.5e-03  6.8e-03 3.8e-04  0.02 1.9e-03  0.02 | 0.77 | | **Altered Metal binding**  **Altered Disordered interface**  Altered Ordered interface  Loss of Loop  **Altered Transmembrane protein**  Loss of Proteolytic cleavage at D53 Gain of Pyrrolidone carboxylic acid at Q56  Loss of Sulfation at Y51 | 6.2e-04 5.5e-03  0.03 0.04 8.5e-03  0.03  0.01  0.02 | 0.697 | Altered Metal binding  Altered Disordered interface  Altered Ordered interface  Altered Transmembrane protein  Loss of Proteolytic cleavage at D53 Gain of Pyrrolidone carboxylic acid at Q56  Loss of Sulfation at Y51 | 2.6e-04 4.5e-03 0.04 0.02  0.03  0.01  0.02 |
| **Q96P** | | | | | | | | | | | | |
| **Isoform 1** | | | **Isoform 2** | | | | **Isoform 3** | | | **Isoform 4** | | |
| **Mutpred score** | **Molecular mechanism** | **P-value** | **Mutpred score** | **Molecular mechanism** | **P-value** | | **Mutpred score** | **Molecular mechanism** | **P-value** | **Mutpred score** | **Molecular  mechanism** | **P-value** |
| 0.683 | Loss of Helix  Altered Transmembrane protein  Altered DNA binding  Gain of Ubiquitylation at K92  Gain of Proteolytic cleavage at R99  Loss of Pyrrolidone carboxylic acid at Q96 | 9.0e-03 3.0e-04 6.7e-03 0.04 0.02 0.01 | 0.88 | **Gain of Intrinsic disorder**  Altered Disordered interface  Loss of Helix  Altered Ordered interface  **Altered Transmembrane protein**  Altered Stability Gain of N-linked glycosylation at N110 | 8.3e-03  0.01  0.02 0.01 3.2e-03  0.01 0.01 | | 0.692 | Loss of Helix  Altered Transmembrane protein  Altered DNA binding  Loss of Pyrrolidone carboxylic acid at Q96  Gain of Proteolytic cleavage at R99 | 8.5e-03 1.3e-03 9.5e-03 5.0e-03 0.02 | 0.708 | Loss of Helix  Altered Transmembrane protein  Altered DNA binding  Gain of Proteolytic cleavage at R99  Loss of Pyrrolidone carboxylic acid at Q96 | 6.8e-03 4.1e-04 8.9e-03 0.02 0.01 |
| **L102P** | | | | | | | | | | | | |
| **Isoform 1** | | | **Isoform 2** | | | | **Isoform 3** | | | **Isoform 4** | | |
| **Mutpred score** | **Molecular mechanism** | **P-value** | **Mutpred score** | **Molecular mechanism** | **P-value** | | **Mutpred score** | **Molecular mechanism** | **P-value** | **Mutpred score** | **Molecular  mechanism** | **P-value** |
| 0.702 | Altered Disordered interface  Loss of Helix  Altered Transmembrane protein  Altered Ordered interface  Altered Stability  Altered DNA binding  Gain of N-linked glycosylation at N110 | 0.01 0.01 3.7e-04 0.01 0.01 0.04 0.02 | 0.609 | Altered Metal binding  Altered Disordered interface  Gain of Strand  Altered Transmembrane protein  Loss of Proteolytic cleavage at D53  Loss of Sulfation at Y51 | 4.9e-03 5.6e-03  0.02 2.4e-03  2.1e-03 0.02 | | 0.828 | Gain of Intrinsic disorder  **Altered Disordered interface  Altered Transmembrane protein  Loss of Helix  Altered DNA binding**  Altered Stability  Loss of Proteolytic cleavage at R99 | 0.01 9.4e-03  5.8e-05  2.4e-03 6.2e-03 0.01 0.01 | 0.775 | **Altered Disordered interface**  Gain of Intrinsic disorder  **Loss of Helix**  **Altered Transmembrane protein**  Altered DNA binding  Altered Stability  Loss of Proteolytic cleavage at R99 | 5.3e-03 0.02 2.3e-03 9.3e-05  0.01 0.01 0.02 |
| **L105Q** | | | | | | | | | | | | |
| **Isoform 1** | | | **Isoform 2** | | | | **Isoform 3** | | | **Isoform 4** | | |
| **Mutpred score** | **Molecular mechanism** | **P-value** | **Mutpred score** | **Molecular mechanism** | **P-value** | | **Mutpred score** | **Molecular mechanism** | **P-value** | **Mutpred score** | **Molecular  mechanism** | **P-value** |
| 0.507 | Altered Disordered interface  Gain of Intrinsic disorder  Altered Transmembrane protein  Altered Ordered interface  Altered DNA binding  Altered Stability  Loss of N-linked glycosylation at N110 | 0.01 0.05 2.1e-04 0.01 0.04 0.02 0.02 | 0.825 | **Altered Metal binding**  **Altered Ordered interface**  Altered Disordered interface  Gain of Strand  **Altered Transmembrane protein**  **Loss of Proteolytic cleavage at D53**  **Gain of Sulfation at D53** | 3.8e-03 3.3e-03  0.02  0.04 1.5e-03  1.5e-03  7.7e-03 | | 0.65 | Altered Disordered interface  Gain of Intrinsic disorder  Altered Transmembrane protein  Loss of Helix  Altered Ordered interface  Altered DNA binding  Loss of Proteolytic cleavage at R106  Altered Stability  Gain of N-linked glycosylation at N110 | 0.02 0.04 1.8e-04  0.04 0.02 0.05 0.02  0.03 0.02 | 0.535 | Altered Disordered interface  Gain of Intrinsic disorder  Altered Transmembrane protein  Altered Ordered interface  Altered Stability  Loss of Proteolytic cleavage at R106  Gain of N-linked glycosylation at N110 | 0.02 0.05 1.9e-04  0.01 0.02 0.05  0.02 |
| **L105P** | | | | | | | | | | | | |
| **Isoform 1** | | | **Isoform 2** | | | | **Isoform 3** | | | **Isoform 4** | | |
| **Mutpred score** | **Molecular mechanism** | **P-value** | **Mutpred score** | **Molecular mechanism** | **P-value** | | **Mutpred score** | **Molecular mechanism** | **P-value** | **Mutpred score** | **Molecular  mechanism** | **P-value** |
| 0.702 | Altered Disordered interface  Loss of Helix  Altered Transmembrane protein  Altered Ordered interface  Altered Stability  Altered DNA binding  Gain of N-linked glycosylation at N110 | 0.01 0.01 3.7e-04 0.01 0.01 0.04 0.02 | 0.731 | Gain of Intrinsic disorder  Altered Disordered interface  Altered Ordered interface  Altered Transmembrane protein  Altered Stability  Loss of N-linked glycosylation at N110 | 0.01 0.02  0.02 4.1e-03  0.02 0.01 | | 0.835 | Altered Disordered interface  **Loss of Helix**  **Altered Transmembrane protein**  Gain of Intrinsic disorder  Altered Ordered interface  Altered Stability  Loss of Proteolytic cleavage at R106  Gain of N-linked glycosylation at N110 | 0.01 3.1e-03 1.9e-04  0.04 0.01 0.02 0.02  0.02 | 0.747 | Altered Disordered interface  Loss of Helix  Altered Transmembrane protein  Altered Ordered interface  Altered Stability  Loss of Proteolytic cleavage at R106  Gain of N-linked glycosylation at N110 | 6.6e-03 3.1e-03 2.8e-04  0.01 0.01 0.04  0.02 |

**Supplementary Table 13.** Prediction of functional effects of the most deleterious SNPs on all soluble HLA-G isoforms (HLA-G5-7) by MutPred. The predictions which are very confident hypotheses shown in bold font.

| **M29K** | | | | | | | | | | | | | | | |
| --- | --- | --- | --- | --- | --- | --- | --- | --- | --- | --- | --- | --- | --- | --- | --- |
| **Isoform 5** | | | | | | **Isoform 6** | | | | | | **Isoform 7** | | | |
| **Mutpred score** | **Molecular mechanism** | **P-value** | | | | **Mutpred score** | | **Molecular mechanism** | | **P-value** | | **Mutpred score** | | **Molecular mechanism** | **P-value** |
| 0.688 | Altered Ordered interface  Loss of Proteolytic cleavage at R30  Altered Stability  Altered Metal binding  Altered Transmembrane protein | 0.02 3.7e-03 0.03 8.3e-03 0.03 | | | | 0.754 | | Altered Ordered interface  Loss of Proteolytic cleavage at R30  Altered Stability  Altered Metal binding  Altered Transmembrane protein | | 0.02 3.5e-03 0.03 8.2e-03 0.03 | | 0.772 | | Altered Ordered interface  **Loss of Proteolytic cleavage at R30**  **Altered Metal binding**  Altered Transmembrane protein  Altered Stability | 0.02 3.0e-03 4.7e-03 0.02 0.03 |
| **F32C** | | | | | | | | | | | | | | | |
| **Isoform 5** | | | | | | **Isoform 6** | | | | | **Isoform 7** | | | | |
| **Mutpred score** | **Molecular mechanism** | **P-value** | | | | **Mutpred score** | **Molecular mechanism** | | **P-value** | | **Mutpred score** | | **Molecular mechanism** | | **P-value** |
| 0.763 | **Altered Ordered interface**  Loss of Strand  **Loss of Proteolytic cleavage at R30**  **Altered Metal binding** | 4.4e-03 0.02 2.2e-03 7.3e-03 | | | | 0.818 | **Altered Ordered interface** Loss of Strand  Altered Metal binding **Altered Transmembrane protein** | | 9.2e-03 0.04 0.05 9.1e-03 | | 0.855 | | **Altered Ordered interface**  Loss of Strand  **Loss of Proteolytic cleavage at R30**  **Altered Metal binding** Altered Transmembrane protein | | 5.6e-03 0.02 1.6e-03 3.7e-03 0.04 |
| **Y51C** | | | | | | | | | | | | | | | |
| **Isoform 5** | | | | | | **Isoform 6** | | | | | **Isoform 7** | | | | |
| **Mutpred score** | **Molecular mechanism** | **P-value** | | | | **Mutpred score** | **Molecular mechanism** | | **P-value** | | **Mutpred score** | | **Molecular mechanism** | | **P-value** |
| 0.668 | Altered Disordered interface  Altered Metal binding  Altered Ordered interface  Loss of Strand  Altered Transmembrane protein  Loss of Proteolytic cleavage at D53  Altered Stability  Gain of Pyrrolidone carboxylic acid at Q56  Loss of Sulfation at Y51 | 1.6e-04 4.3e-03 4.3e-03 0.01 4.4e-03 0.03 0.03 0.02 0.02 | | | | 0.597 | Altered Metal binding  Altered Disordered interface  Altered Transmembrane protein  Loss of Strand  Loss of Proteolytic cleavage at D53  Loss of Sulfation at Y51 | | 3.6e-03 6.1e-04 5.8e-05 0.02 1.9e-03 0.02 | | 0.727 | | Altered Disordered interface  Altered Metal binding  Altered Ordered interface  Loss of Strand  Altered Transmembrane protein  Loss of Proteolytic cleavage at D53  Altered Stability  Gain of Pyrrolidone carboxylic acid at Q56  Loss of Sulfation at Y51 | | 2.7e-04 3.3e-03 0.02 0.01 1.9e-03 0.03 0.03 0.02 0.02 |
| **D53N** | | | | | | | | | | | | | | | |
| **Isoform 5** | | | **Isoform 6** | | | | | | | | **Isoform 7** | | | | |
| **Mutpred score** | **Molecular mechanism** | **P-value** | **Mutpred score** | | | | **Molecular mechanism** | | **P-value** | | **Mutpred score** | | **Molecular mechanism** | | **P-value** |
| 0.432 | - | - | 0.625 | | | | Altered Disordered interface Altered Metal binding  Gain of Strand  Loss of Loop  Altered Transmembrane protein  Loss of Proteolytic  Gain of N-linked glycosylation at D53  Loss of Sulfation at Y51 | | 9.0e-03 1.0e-02 0.03 0.04 1.4e-03 1.4e-03 0.01 0.02 | | 0.536 | | Altered Metal binding  Altered Disordered interface  Altered Ordered interface  Altered Transmembrane protein  Loss of Proteolytic cleavage at D53  Loss of Pyrrolidone carboxylic acid at Q56  Gain of N-linked glycosylation at D53 Loss of Sulfation at Y51 | | 1.5e-03 0.01 0.04 4.7e-03 0.03 0.02 0.02 0.02 |
| **D53Y** | | | | | | | | | | | | | | | |
| **Isoform 5** | | | **Isoform 6** | | | | | | | | **Isoform 7** | | | | |
| **Mutpred score** | **Molecular mechanism** | **P-value** | **Mutpred score** | | | | **Molecular mechanism** | | **P-value** | | **Mutpred score** | | **Molecular mechanism** | | **P-value** |
| 0.661 | Altered Ordered interface  Altered Disordered interface  Altered Metal binding  Gain of Strand  Altered Transmembrane protein  Loss of Proteolytic cleavage at D53  Gain of Pyrrolidone carboxylic acid at Q56  Gain of Sulfation at D53 | 7.2e-04 6.6e-03 1.7e-03 0.04 6.0e-03 0.03 0.01 4.6e-03 | 0.814 | | | | **Altered Metal binding**  **Altered Ordered interface**  Altered Disordered interface  **Altered Transmembrane protein**  Loss of Loop  **Loss of Proteolytic cleavage at D53  Gain of Sulfation at D53** | | 6.1e-03 5.6e-03 0.01 6.0e-04 0.04 1.4e-03 6.7e-03 | | 0.772 | | **Altered Ordered interface  Altered Disordered interface  Altered Metal binding Altered Transmembrane protein**  Loss of Proteolytic cleavage at D53  Gain of Pyrrolidone carboxylic acid at Q56  **Gain of Sulfation at D53** | | 3.8e-04 9.3e-03 1.9e-03 3.2e-03 0.03 0.01 4.5e-03 |
| **D54V** | | | | | | | | | | | | | | | |
| **Isoform 5** | | | **Isoform 6** | | | | | | | | **Isoform 7** | | | | |
| **Mutpred score** | **Molecular mechanism** | **P-value** | **Mutpred score** | | | | **Molecular mechanism** | | **P-value** | | **Mutpred score** | | **Molecular mechanism** | | **P-value** |
| 0.638 | Altered Metal binding  Altered Disordered interface  Altered Ordered interface  Altered Transmembrane protein  Loss of Proteolytic cleavage at D53  Gain of Pyrrolidone carboxylic acid at Q56  Loss of Sulfation at Y51 | 3.9e-04 4.6e-03 0.02 0.02 0.03 0.01 0.02 | 0.583 | | | | Altered Disordered interface  Altered Metal binding  Loss of Loop  Gain of Strand  Altered Transmembrane protein  Loss of Proteolytic cleavage at D53  Loss of Sulfation at Y51 | | 3.6e-03 7.2e-03 0.04 0.04 1.5e-03 2.0e-03 0.02 | | 0.745 | | Altered Metal binding  Altered Disordered interface  Altered Ordered interface Altered Transmembrane protein Loss of Proteolytic cleavage at D53  Gain of Pyrrolidone carboxylic acid at Q56  Loss of Sulfation at Y51 | | 5.7e-04 5.4e-03 0.03 9.9e-03 0.03 0.02 0.02 |
| **Q96P** | | | | | | | | | | | | | | | |
| **Isoform 5** | | | | **Isoform 6** | | | | | | | **Isoform 7** | | | | |
| **Mutpred score** | **Molecular mechanism** | **P-value** | | **Mutpred score** | | | **Molecular mechanism** | | **P-value** | | **Mutpred score** | | **Molecular mechanism** | | **P-value** |
| 0.619 | Loss of Helix  Altered Transmembrane protein  Altered DNA binding  Gain of Ubiquitylation at K92  Gain of Proteolytic cleavage at R99  Loss of Pyrrolidone carboxylic acid at Q96 | 9.3e-03 4.2e-04 6.6e-03 0.04 0.02  0.01 | | 0.529 | | | Loss of Helix  Gain of Loop  Altered Transmembrane protein  Gain of Relative solvent accessibility | | 0.01 0.01 6.9e-04 0.03 | | 0.683 | | Loss of Helix  Altered DNA binding  Altered Transmembrane protein  Altered Disordered interface  Gain of Proteolytic cleavage at R99  Loss of Pyrrolidone carboxylic acid at Q96 | | 8.4e-03 3.1e-03 2.4e-03 0.04 0.02 0.01 |
| **L102P** | | | | | | | | | | | | | | | |
| **Isoform 5** | | | | | **Isoform 6** | | | | | | **Isoform 7** | | | | |
| **Mutpred score** | **Molecular mechanism** | **P-value** | | | **Mutpred score** | | **Molecular mechanism** | | **P-value** | | **Mutpred score** | | **Molecular mechanism** | | **P-value** |
| 0.667 | Altered Disordered interface  Gain of Intrinsic disorder  Altered Transmembrane protein  Loss of Helix  Altered DNA binding  Altered Stability  Loss of Proteolytic cleavage at R99 | 6.7e-03 0.02 1.1e-04 8.3e-03 0.01 0.01 0.02 | | | 0.693 | | Gain of Intrinsic disorder  Altered Disordered interface  Loss of Helix  Gain of Loop  Altered Transmembrane protein  Altered Stability | | 4.0e-03 0.02 0.01 9.1e-03 1.2e-03 0.01 | | 0.798 | | **Gain of Intrinsic disorder**  **Loss of Helix**  **Altered Transmembrane protein** Altered Disordered interface  **Altered DNA binding**  Loss of Proteolytic cleavage at R106  Altered Stability | | 1.7e-03 4.0e-03  4.7e-04 0.04 7.2e-03 0.02 0.03 |
| **L105P** | | | | | | | | | | | | | | | |
| **Isoform 5** | | | | | | **Isoform 6** | | | | | **Isoform 7** | | | | |
| **Mutpred score** | **Molecular mechanism** | **P-value** | | | | **Mutpred score** | **Molecular mechanism** | | **P-value** | | **Mutpred score** | | **Molecular mechanism** | | **P-value** |
| 0.688 | Altered Disordered interface  Loss of Helix  Altered Transmembrane protein  Altered Ordered interface  Altered Stability  Loss of Proteolytic cleavage at R106  Gain of N-linked glycosylation at N110 | 9.3e-03 9.2e-03 4.9e-04 0.01 0.01 0.04 0.02 | | | | 0.843 | **Gain of Intrinsic disorder** Altered Disordered interface Loss of Helix  Altered Ordered interface  **Altered Transmembrane protein**  Altered Stability  Gain of N-linked glycosylation at N110 | | 8.3e-03 0.02 0.02 0.02 2.4e-03 0.01 0.01 | | 0.812 | | **Gain of Intrinsic disorder**  **Loss of Helix  Altered Transmembrane protein**  Altered Ordered interface  Altered Disordered interface  Altered DNA binding  Altered Stability  Loss of Proteolytic cleavage at R106  Gain of N-linked glycosylation at N110 Loss of Sulfation at Y109 | | 2.2e-03 4.6e-03 6.9e-04 0.04 0.05 0.04 0.02 0.02 0.03 0.04 |

**Supplementary Table 14.** The effects of each of the most deleterious predicted SNPs on native HLA-G 5 isoform structure and the difference in physicochemical properties of amino acids of wild type and mutated residue as predicted by HOPE. The 3D structures for HLA-G5 were not predicted by HOPE due to unknown reason.

| **Prediction of protein structure (Isoform 5) in deleterious nsSNP by Project HOPE** | | |
| --- | --- | --- |
| **M29K** | **Amino acid properties** | The mutant residue is bigger than the wild-type residue.  The wild-type amino acid has a neutral charge while the mutant amino acid has a positive charge.  The mutation introduces a charge; this can cause repulsion of ligands or other residues with the same charge.  The wild-type residue is more hydrophobic than the mutant residue.  Hydrophobic interactions will be lost either in the core of the protein or on the surface. |
|  | **Structure** | The mutation is located within a stretch of residues annotated in UniProt as a special region: Alpha-1. The diversities in residue characteristics can distort this region and distort its function. |
| **R30S** | **Amino acid properties** | The wild-type amino acid has a positive charge while the mutant amino acid has a neutral charge.  The charge of the wild-type residue will be lost; this can cause loss of interactions with other molecules or residues.  The mutant residue is smaller; this might lead to loss of interactions.  The mutation introduces a more hydrophobic residue at this position which can result in loss of hydrogen bonds and/or disturb appropriate folding. |
|  | **Structure** | The mutation is located within a stretch of residues annotated in UniProt as a special region: Alpha-1. The diversities in residue characteristics can distort this region and distort its function. |
| **F32C** | **Amino acid properties** | The mutant residue is smaller than the wild-type residue, which will cause a possible loss of external interactions. |
|  | **Structure** | The mutation is located within a stretch of residues annotated in UniProt as a special region: Alpha-1. The diversities in residue characteristics can distort this region and distort its function. |
| **P44L** | **Amino acid properties** | The mutant residue is bigger; this might lead to bumps.  Prolines are known to have a very rigid structure, sometimes forcing the the backbone in a specific conformation. Possibly, this mutation changes proline with such a function into another residue, thereby disturbing the local structure. |
|  | **Structure** | The mutation is located within a stretch of residues annotated in UniProt as a special region: Alpha-1. The diversities in residue characteristics can distort this region and distort its function.  The wild-type residue is proline. Prolines are known to be very rigid and therefore induce a special backbone conformation that might be required at this position. The mutation can disturb this special conformation. |
| **Y51C** | **Amino acid properties** | The mutant residue is smaller than the wild-type residue.  The mutant residue is more hydrophobic than the wild-type residue.  The mutation introduces a more hydrophobic residue at this position which can result in loss of hydrogen bonds and/or disturb appropriate folding. |
|  | **Structure** | The mutation is located within a stretch of residues annotated in UniProt as a special region: Alpha-1. The diversities in residue characteristics can distort this region and distort its function. |
| **D53N** | **Amino acid properties** | The wild-type residue charge was negative while the mutant residue charge is neutral.  The charge of the wild-type residue will be lost, this can cause loss of interactions with other molecules or residues. |
|  | **Structure** | The mutation is located within a stretch of residues annotated in UniProt as a special region: Alpha-1. The diversities in residue characteristics can distort this region and distort its function. |
| **D53Y** | **Amino acid properties** | The wild-type residue charge was negative; the mutant residue charge is neutral.  The charge of the wild-type residue will be lost; this can cause loss of interactions with other molecules or residues.  The mutant residue is bigger; this might lead to bumps.  The mutant residue is more hydrophobic than the wild-type residue.  This can result in loss of hydrogen bonds and/or disturb correct folding. |
|  | **Structure** | The mutation is located within a stretch of residues annotated in UniProt as a special region: Alpha-1. The diversities in residue characteristics can distort this region and distort its function. |
| **D54V** | **Amino acid properties** | The wild-type residue charge was negative; the mutant residue charge is neutral.  The charge of the wild-type residue will be lost; this can cause loss of interactions with other molecules or residues.  The mutant residue is smaller; this might lead to loss of interactions.  The mutant residue is more hydrophobic than the wild-type residue.  The mutation introduces a more hydrophobic residue at this position which can result in loss of hydrogen bonds and/or disturb appropriate folding. |
|  | **Structure** | The mutation is located within a stretch of residues annotated in UniProt as a special region: Alpha-1. The diversities in residue characteristics can distort this region and distort its function. |
| **D54Y** | **Amino acid properties** | The wild-type residue charge was negative; the mutant residue charge is neutral.  The charge of the wild-type residue will be lost; this can cause loss of interactions with other molecules or residues.  The mutant residue is bigger; this might lead to bumps.  The mutation introduces a more hydrophobic residue at this position which can result in loss of hydrogen bonds and/or disturb appropriate folding. |
|  | **Structure** | The mutation is located within a stretch of residues annotated in UniProt as a special region: Alpha-1. The diversities in residue characteristics can distort this region and distort its function. |
| **Q96P** | **Amino acid properties** | The mutant residue is smaller; this might lead to loss of interactions.  The mutant residue is more hydrophobic than the wild-type residue.  The mutation introduces a more hydrophobic residue at this position which can result in loss of hydrogen bonds and/or disturb appropriate folding. |
|  | **Structure** | The mutation is located within a stretch of residues annotated in UniProt as a special region: Alpha-1. The diversities in residue characteristics can distort this region and distort its function.  In the case of the mutation, the helix will be disturbed and this can have severe effects on the structure of the protein. |
| **L102P** | **Amino acid properties** | The mutant residue is smaller; this might lead to loss of interactions. |
|  | **Structure** | The mutation is located within a stretch of residues annotated in UniProt as a special region: Alpha-1. The diversities in residue characteristics can distort this region and distort its function.  In the case of the mutation, the helix will be disturbed and this can have severe effects on the structure of the protein. |
| **L105Q** | **Amino acid properties** | The mutant residue is bigger; this might lead to bumps.  The wild-type residue is more hydrophobic than the mutant residue.  Hydrophobic interactions will be lost either in the core of the protein or on the surface. |
|  | **Structure** | The mutation is located within a stretch of residues annotated in UniProt as a special region: Alpha-1. The diversities in residue characteristics can distort this region and distort its function. |
| **L105P** | **Amino acid properties** | The mutant residue is smaller; this might lead to loss of interactions. |
|  | **Structure** | The mutation is located within a stretch of residues annotated in UniProt as a special region: Alpha-1. The diversities in residue characteristics can distort this region and distort its function.  In the case of the mutation, the helix will be disturbed and this can have severe effects on the structure of the protein. |
| **E113V** | **Amino acid properties** | The wild-type residue charge was negative; the mutant residue charge is neutral.  The charge of the wild-type residue will be lost; this can cause loss of interactions with other molecules or residues.  The mutant residue is smaller; this might lead to loss of interactions.  The mutation introduces a more hydrophobic residue at this position which can result in loss of hydrogen bonds and/or disturb appropriate folding. |
|  | **Structure** | The mutation is located within a stretch of residues annotated in UniProt as a special region: Alpha-1. The diversities in residue characteristics can distort this region and distort its function. |
| **H117P** | **Amino acid properties** | The mutant residue is smaller; this might lead to loss of interactions.  The mutation introduces a more hydrophobic residue at this position which can result in loss of hydrogen bonds and/or disturb appropriate folding. |
|  | **Structure** | The mutation is located within a stretch of residues annotated in UniProt as a special region: Alpha-2. The diversities in residue characteristics can distort this region and distort its function. |
| **H117L** | **Amino acid properties** | The mutant residue is smaller; this might lead to loss of interactions.  The mutation introduces a more hydrophobic residue at this position which can result in loss of hydrogen bonds and/or disturb appropriate folding. |
|  | **Structure** | The mutation is located within a stretch of residues annotated in UniProt as a special region: Alpha-2. The diversities in residue characteristics can distort this region and distort its function. |
| **D130H** | **Amino acid properties** | The wild-type residue charge was negative; the mutant residue charge is neutral.  The charge of the wild-type residue will be lost; this can cause loss of interactions with other molecules or residues.  The mutant residue is bigger, this might lead to bumps. |
|  | **Structure** | The mutation is located within a stretch of residues annotated in UniProt as a special region: Alpha-2. The diversities in residue characteristics can distort this region and distort its function. |
| **Y142C** | **Amino acid properties** | The mutant residue is smaller; this might lead to loss of interactions.  The mutation introduces a more hydrophobic residue at this position which can result in loss of hydrogen bonds and/or disturb appropriate folding. |
|  | **Structure** | The mutation is located within a stretch of residues annotated in UniProt as a special region: Alpha-2. The diversities in residue characteristics can distort this region and distort its function. |
| **Y142H** | **Amino acid properties** | The mutant residue is smaller; this might lead to loss of interactions.  The wild-type residue is more hydrophobic than the mutant residue.  Hydrophobic interactions will be lost either in the core of the protein or on the surface. |
|  | **Structure** | The mutation is located within a stretch of residues annotated in UniProt as a special region: Alpha-2. The diversities in residue characteristics can distort this region and distort its function. |
| **D143N** | **Amino acid properties** | The wild-type residue charge was negative; the mutant residue charge is neutral.  The charge of the wild-type residue will be lost; this can cause loss of interactions with other molecules or residues. |
|  | **Structure** | The mutation is located within a stretch of residues annotated in UniProt as a special region: Alpha-2. The diversities in residue characteristics can distort this region and distort its function. |
| **D143H** | **Amino acid properties** | The wild-type residue charge was negative; the mutant residue charge is neutral.  The charge of the wild-type residue will be lost; this can cause loss of interactions with other molecules or residues.  The mutant residue is bigger, this might lead to bumps. |
|  | **Structure** | The mutation is located within a stretch of residues annotated in UniProt as a special region: Alpha-2. The diversities in residue characteristics can distort this region and distort its function. |
| **D153G** | **Amino acid properties** | The wild-type residue charge was negative; the mutant residue charge is neutral.  The charge of the wild-type residue will be lost; this can cause loss of interactions with other molecules or residues.  The mutant residue is smaller; this might lead to loss of interactions.  The mutation introduces a more hydrophobic residue at this position which can result in loss of hydrogen bonds and/or disturb appropriate folding. |
|  | **Structure** | The mutation is located within a stretch of residues annotated in UniProt as a special region: Alpha-2. The diversities in residue characteristics can distort this region and distort its function.  The mutation introduces a glycine at this position. Glycines are very flexible and can disturb the required rigidity of the protein at this position. |
| **W157R** | **Amino acid properties** | The wild-type amino acid has a neutral charge while the mutant amino acid has a positive charge.  The mutation introduces a charge; this can cause repulsion of ligands or other residues with the same charge.  The mutant residue is smaller; this might lead to loss of interactions.  The wild-type residue is more hydrophobic than the mutant residue.  Hydrophobic interactions will be lost either in the core of the protein or on the surface. |
|  | **Structure** | The mutation is located within a stretch of residues annotated in UniProt as a special region: Alpha-2. The diversities in residue characteristics can distort this region and distort its function. |
| **T158P** | **Amino acid properties** | The mutation introduces a more hydrophobic residue at this position which can result in loss of hydrogen bonds and/or disturb appropriate folding. |
|  | **Structure** | The mutation is located within a stretch of residues annotated in UniProt as a special region: Alpha-2. The diversities in residue characteristics can distort this region and distort its function. |
| **C188S** | **Amino acid properties** | The wild-type residue is more hydrophobic than the mutant residue.  Hydrophobic interactions will be lost either in the core of the protein or on the surface. |
|  | **Structure** | The mutation is located within a stretch of residues annotated in UniProt as a special region: Alpha-2. The diversities in residue characteristics can distort this region and distort its function. |
| **L196P** | **Amino acid properties** | The mutant residue is smaller; this might lead to loss of interactions. |
|  | **Structure** | The mutation is located within a stretch of residues annotated in UniProt as a special region: Alpha-2. The diversities in residue characteristics can distort this region and distort its function.  In the case of the mutation, the helix will be disturbed and this can have severe effects on the structure of the protein. |
| **R205S** | **Amino acid properties** | The wild-type amino acid has a positive charge while the mutant amino acid has a neutral charge.  The charge of the wild-type residue will be lost; this can cause loss of interactions with other molecules or residues.  The mutant residue is smaller; this might lead to loss of interactions.  The mutation introduces a more hydrophobic residue at this position which can result in loss of hydrogen bonds and/or disturb appropriate folding. |
|  | **Structure** | The mutation is located within a stretch of residues annotated in UniProt as a special region: Alpha-2. The diversities in residue characteristics can distort this region and distort its function. |
| **R205L** | **Amino acid properties** | The wild-type amino acid has a positive charge while the mutant amino acid has a neutral charge.  The charge of the wild-type residue will be lost; this can cause loss of interactions with other molecules or residues.  The mutant residue is smaller; this might lead to loss of interactions.  The mutation introduces a more hydrophobic residue at this position which can result in loss of hydrogen bonds and/or disturb appropriate folding. |
|  | **Structure** | The mutation is located within a stretch of residues annotated in UniProt as a special region: Alpha-2. The diversities in residue characteristics can distort this region and distort its function. |
| **P209A** | **Amino acid properties** | The mutant residue is smaller; this might lead to loss of interactions.  Prolines are known to have a very rigid structure, sometimes forcing the backbone in a specific conformation. possibly, your mutation changes a proline with such a function into another residue, thereby disturbing the local structure. |
|  | **Structure** | The mutation is located within a domain, annotated in UniProt as Ig-like C1-type  The mutant amino acid has diverse characteristics than the wild amino acid that can distort this domain and abolish its function.  The mutation is located within a stretch of residues annotated in UniProt as a special region: Alpha-3. The diversities in residue characteristics can distort this region and distort its function.  The wild-type residue is proline. Prolines are known to be very rigid and therefore induce a special backbone conformation that might be required at this position. This special conformation can be disturbed due to the mutation. |
| **P209R** | **Amino acid properties** | The wild-type amino acid has a neutral charge while the mutant amino acid has a positive charge.  The mutation introduces a charge; this can cause repulsion of ligands or other residues with the same charge.  The mutant residue is bigger; this might lead to bumps.  The wild-type residue is more hydrophobic than the mutant residue.  Hydrophobic interactions, either in the core of the protein or on the surface, will be lost. |
|  | **Structure** | The mutation is located within a domain, annotated in UniProt as Ig-like C1-type  The mutant amino acid has diverse characteristics than the wild amino acid that can distort this domain and abolish its function.  The mutation is located within a stretch of residues annotated in UniProt as a special region: Alpha-3. The diversities in residue characteristics can distort this region and distort its function.  The wild-type residue is proline. Prolines are known to be very rigid and therefore induce a special backbone conformation that might be required at this position. The mutation can disturb this special conformation (the local structure). |
| **C227Y** | **Amino acid properties** | The mutant residue is bigger; this might lead to bumps.  The wild-type residue is more hydrophobic than the mutant residue.  Hydrophobic interactions will be lost either in the core of the protein or on the surface. |
|  | **Structure** | The mutation is located within a domain, annotated in UniProt as Ig-like C1-type  The mutant amino acid has diverse characteristics than the wild amino acid that can distort this domain and abolish its function.  The mutation is located within a stretch of residues annotated in UniProt as a special region: Alpha-3. The diversities in residue characteristics can distort this region and distort its function. |
| **C227F** | **Amino acid properties** | The mutant residue is bigger, this might lead to bumps. |
|  | **Structure** | The mutation is located within a domain, annotated in UniProt as Ig-like C1-type  The mutant amino acid has diverse characteristics than the wild amino acid that can distort this domain and abolish its function.  The mutation is located within a stretch of residues annotated in UniProt as a special region: Alpha-3. The diversities in residue characteristics can distort this region and distort its function. |
| **A229D** | **Amino acid properties** | The wild-type amino acid has a neutral charge while the mutant amino acid has a negative charge.  The mutation introduces a charge; this can cause repulsion of ligands or other residues with the same charge.  The mutant residue is bigger; this might lead to bumps.  The wild-type residue is more hydrophobic than the mutant residue.  Hydrophobic interactions will be lost either in the core of the protein or on the surface. |
|  | **Structure** | The mutation is located within a domain, annotated in UniProt as Ig-like C1-type  The mutant amino acid has diverse characteristics than the wild amino acid that can distort this domain and abolish its function.  The mutation is located within a stretch of residues annotated in UniProt as a special region: Alpha-3. The diversities in residue characteristics can distort this region and distort its function. |
| **P234T** | **Amino acid properties** | The wild-type residue is more hydrophobic than the mutant residue.  Hydrophobic interactions will be lost either in the core of the protein or on the surface. |
|  | **Structure** | The mutation is located within a domain, annotated in UniProt as Ig-like C1-type  The mutant amino acid has diverse characteristics than the wild amino acid that can distort this domain and abolish its function.  The mutation is located within a stretch of residues annotated in UniProt as a special region: Alpha-3. The diversities in residue characteristics can distort this region and distort its function.  The wild-type residue is proline. Prolines are known to be very rigid and therefore induce a special backbone conformation that might be required at this position. This special conformation can be disturbed due to the mutation. |
| **P234L** | **Amino acid properties** | The mutant residue is bigger, this might lead to bumps. |
|  | **Structure** | The mutation is located within a domain, annotated in UniProt as Ig-like C1-type  The mutant amino acid has diverse characteristics than the wild amino acid that can distort this domain and abolish its function.  The mutation is located within a stretch of residues annotated in UniProt as a special region: Alpha-3. The diversities in residue characteristics can distort this region and distort its function.  The wild-type residue is proline. Prolines are known to be very rigid and therefore induce a special backbone conformation that might be required at this position. This special conformation can be disturbed due to the mutation. |
| **I237F** | **Amino acid properties** | The mutant residue is bigger, this might lead to bumps. |
|  | **Structure** | The mutation is located within a domain, annotated in UniProt as Ig-like C1-type  The mutant amino acid has diverse characteristics than the wild amino acid that can distort this domain and abolish its function.  The mutation is located within a stretch of residues annotated in UniProt as a special region: Alpha-3. The diversities in residue characteristics can distort this region and distort its function. |
| **P259H** | **Amino acid properties** | The mutant residue is bigger; this might lead to bumps.  The wild-type residue is more hydrophobic than the mutant residue.  Hydrophobic interactions will be lost either in the core of the protein or on the surface. |
|  | **Structure** | The mutation is located within a domain, annotated in UniProt as Ig-like C1-type  The mutant amino acid has diverse characteristics than the wild amino acid that can distort this domain and abolish its function.  The mutation is located within a stretch of residues annotated in UniProt as a special region: Alpha-3. The diversities in residue characteristics can distort this region and distort its function.  The wild-type residue is proline. Prolines are known to be very rigid and therefore induce a special backbone conformation that might be required at this position. This special conformation can be disturbed due to the mutation. |
| **Q266L** | **Amino acid properties** | The mutant residue is smaller; this might lead to loss of interactions.  The mutant residue is more hydrophobic than the wild-type residue.  The mutation introduces a more hydrophobic residue at this position which can result in loss of hydrogen bonds and/or disturb appropriate folding. |
|  | **Structure** | The mutation is located within a domain, annotated in UniProt as Ig-like C1-type  The mutant amino acid has diverse characteristics than the wild amino acid that can distort this domain and abolish its function.  The mutation is located within a stretch of residues annotated in UniProt as a special region: Alpha-3. The diversities in residue characteristics can distort this region and distort its function. |
| **H287Y** | **Amino acid properties** | The mutant residue is bigger; this might lead to bumps.  The mutant residue is more hydrophobic than the wild-type residue.  The mutation introduces a more hydrophobic residue at this position which can result in loss of hydrogen bonds and/or disturb appropriate folding. |
|  | **Structure** | The mutation is located within a domain, annotated in UniProt as Ig-like C1-type  The mutant amino acid has diverse characteristics than the wild amino acid that can distort this domain and abolish its function.  The mutation is located within a stretch of residues annotated in UniProt as a special region: Alpha-3. The diversities in residue characteristics can distort this region and distort its function. |
| **W298R** | **Amino acid properties** | The wild-type amino acid has a neutral charge while the mutant amino acid has a positive charge.  The mutation introduces a charge; this can cause repulsion of ligands or other residues with the same charge.  The mutant residue is smaller; this might lead to loss of interactions.  The wild-type residue is more hydrophobic than the mutant residue.  Hydrophobic interactions will be lost either in the core of the protein or on the surface. |
|  | **Structure** | The mutation is located within a domain, annotated in UniProt as Ig-like C1-type  The mutant amino acid has diverse characteristics than the wild amino acid that can distort this domain and abolish its function.  The mutation is located within a stretch of residues annotated in UniProt as a special region: Alpha-3. The diversities in residue characteristics can distort this region and distort its function. |

**Supplementary Table 15.** Data on the effects of each the common most deleterious predicted SNPs on structures of native membrane bound isoforms and the difference in physicochemical properties of wild type and mutated residue as predicted by HOPE. Each close-up Image represents the 3D structures of the membrane bound isoforms (gray color) with its wild type residue (green color) and mutant residue (red color).

| **Prediction of protein structure (membrane bound isoforms) in most deleterious nsSNPs by Project HOPE** | | | |  |  |
| --- | --- | --- | --- | --- | --- |
| **Isoform 1** | **Isoform 2** | **Isoform 3** | **Isoform 4** |  |  |
| **M29K** | **Image** | 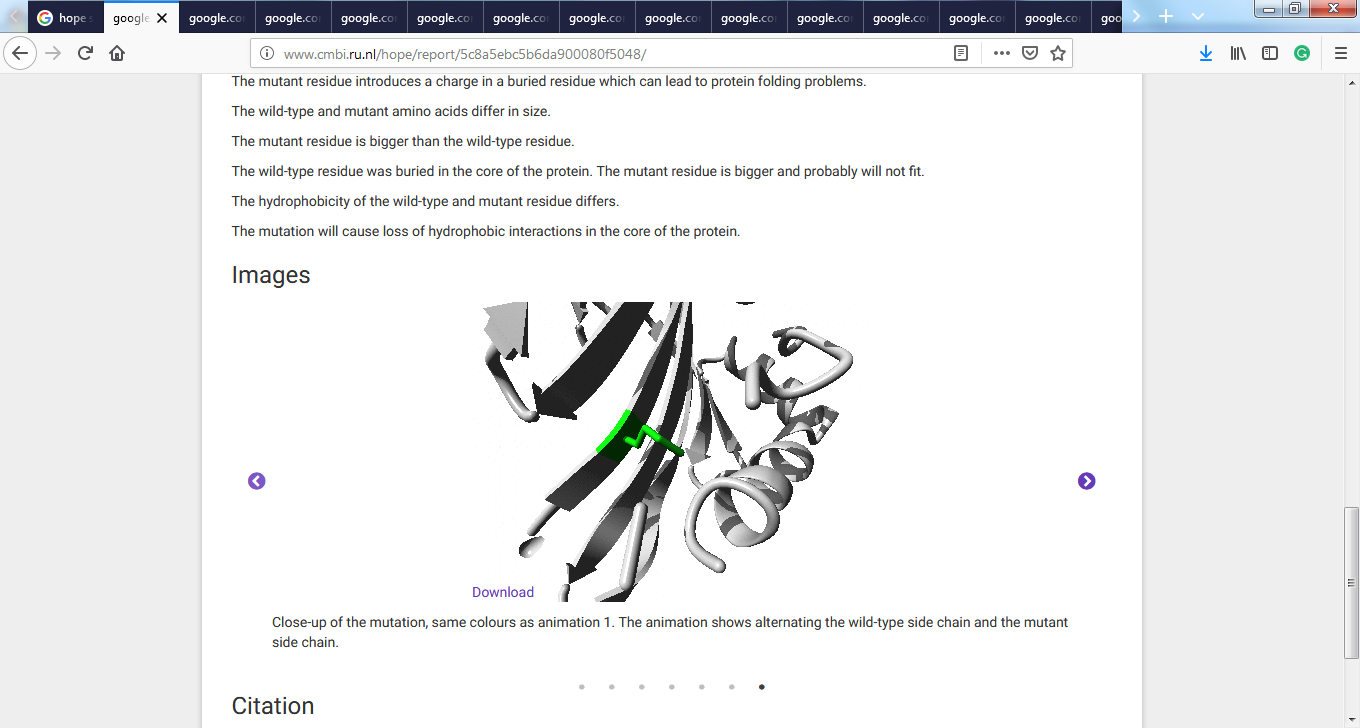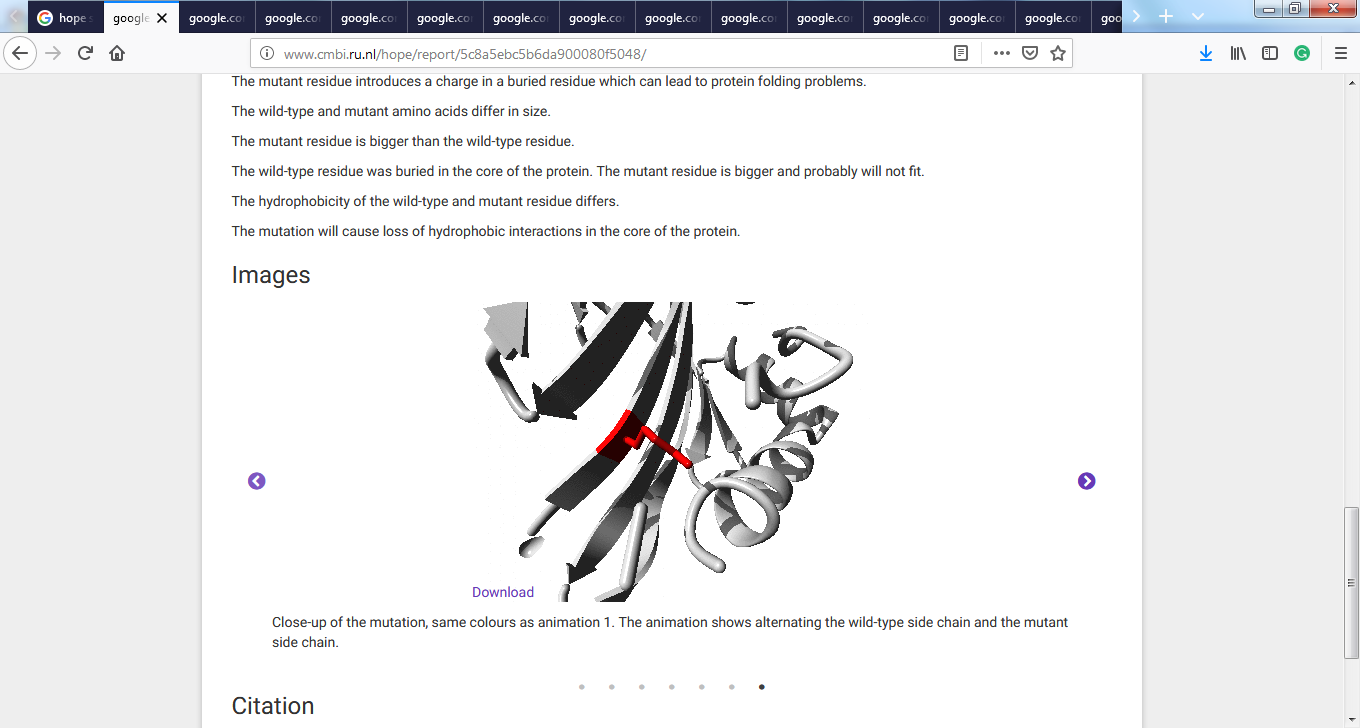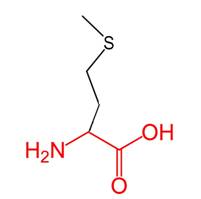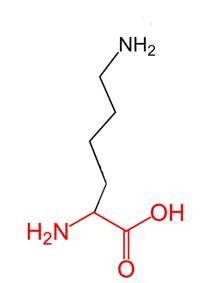 | 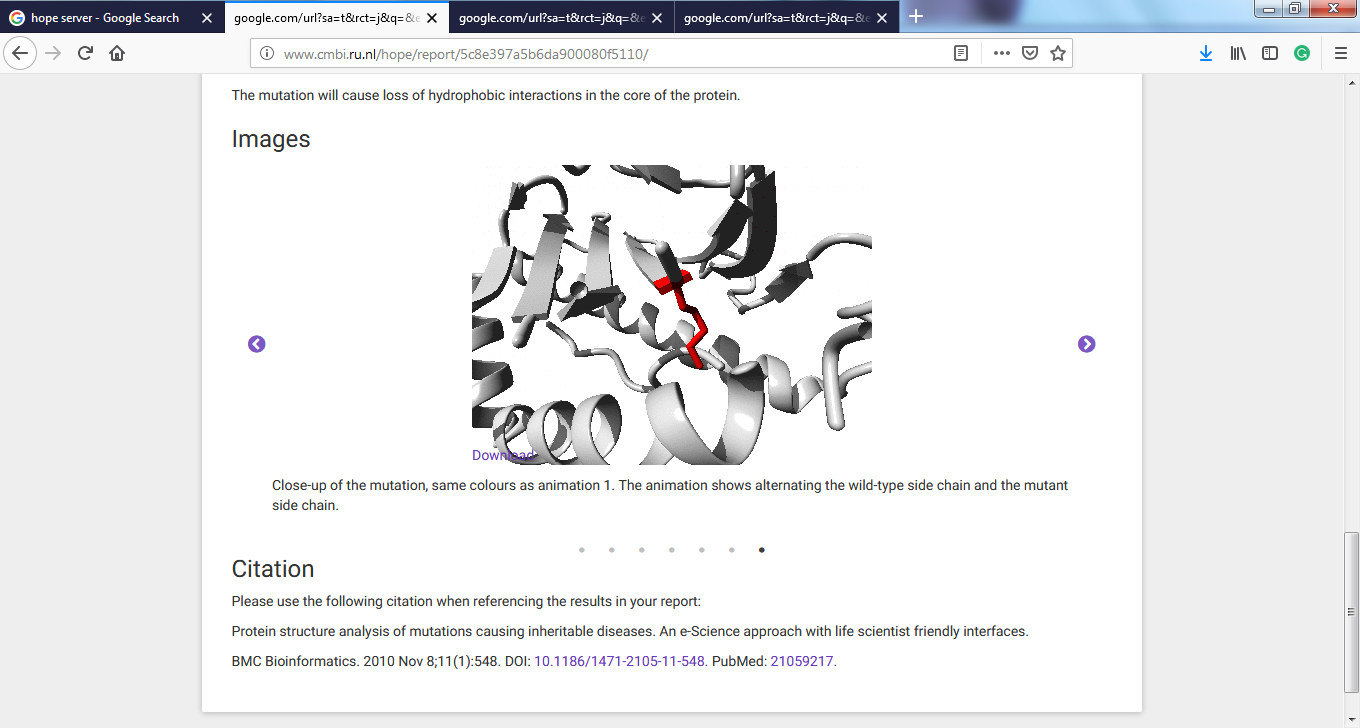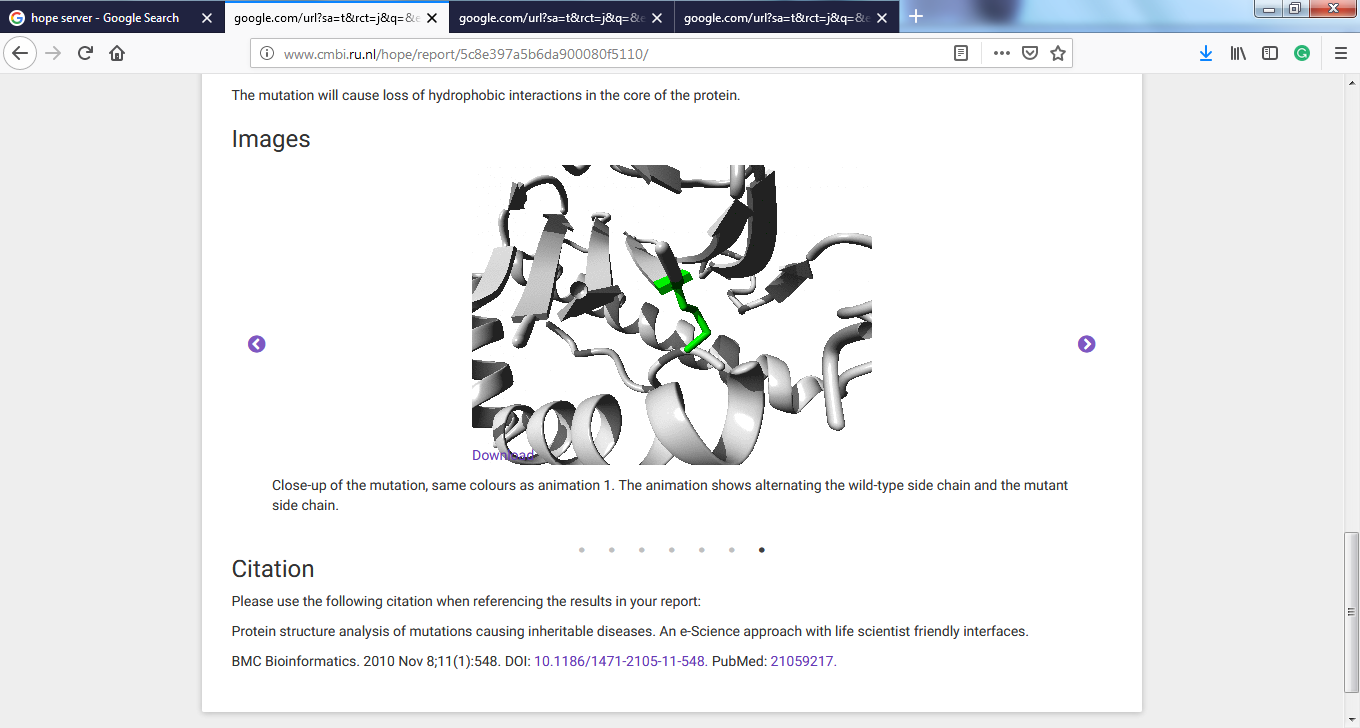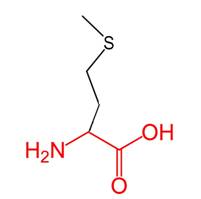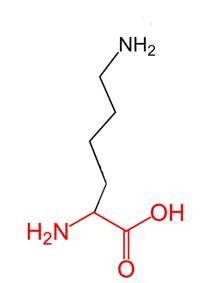 | 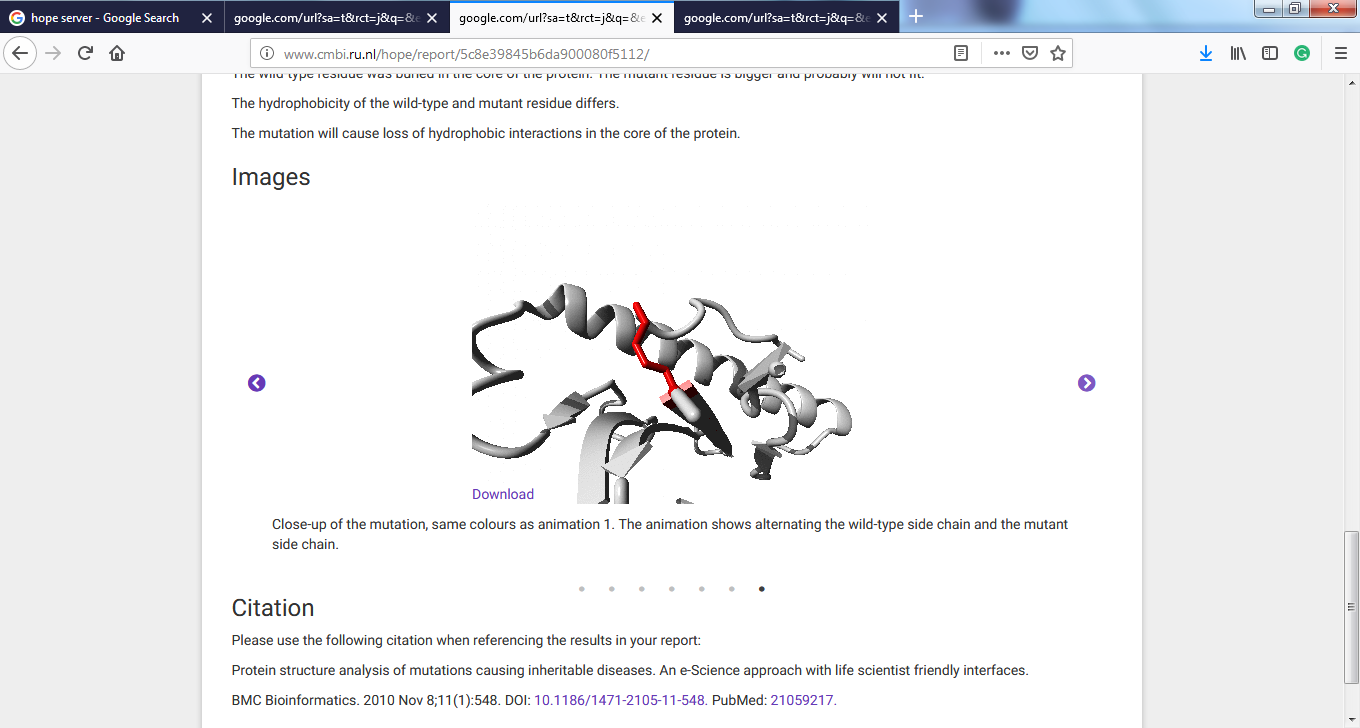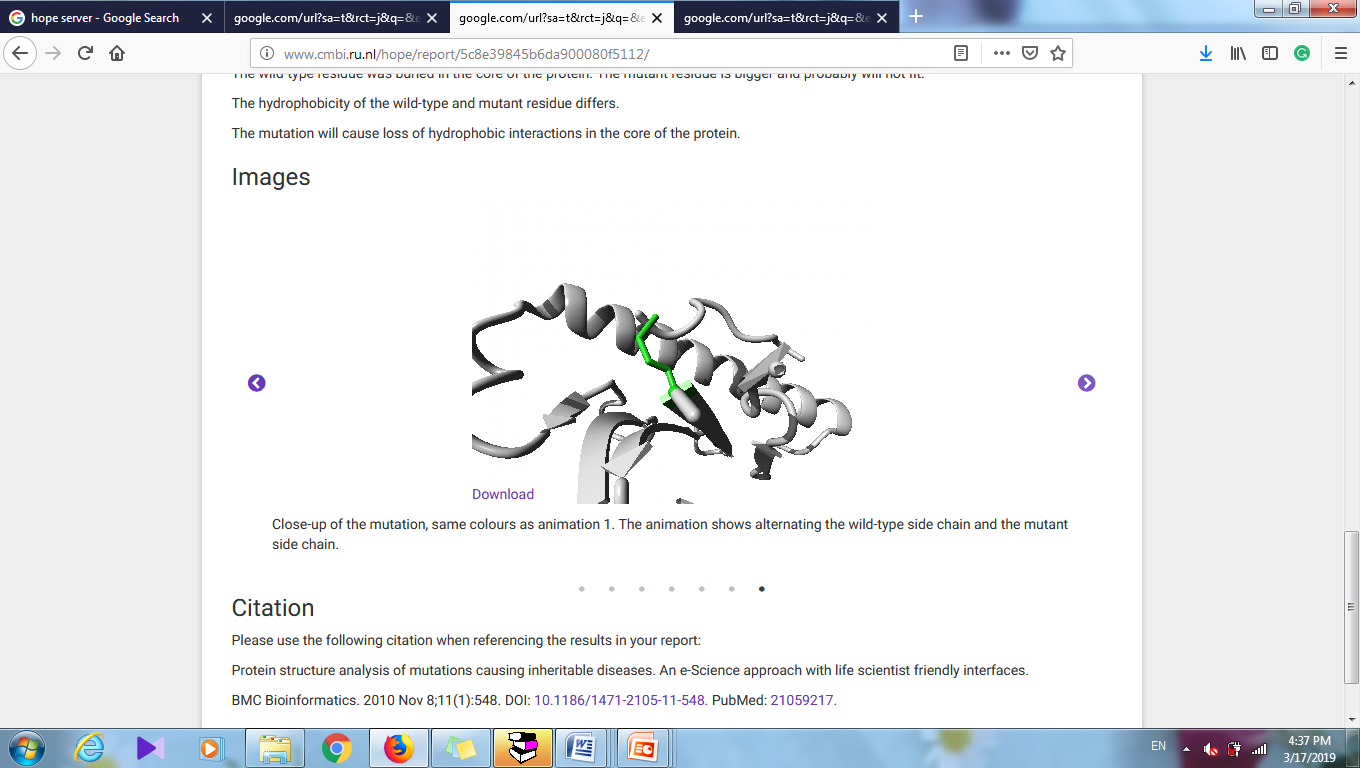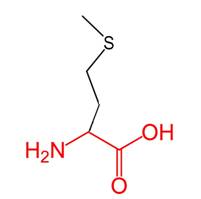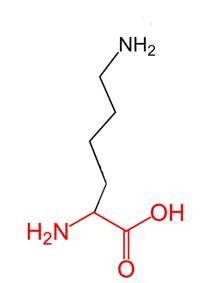 | 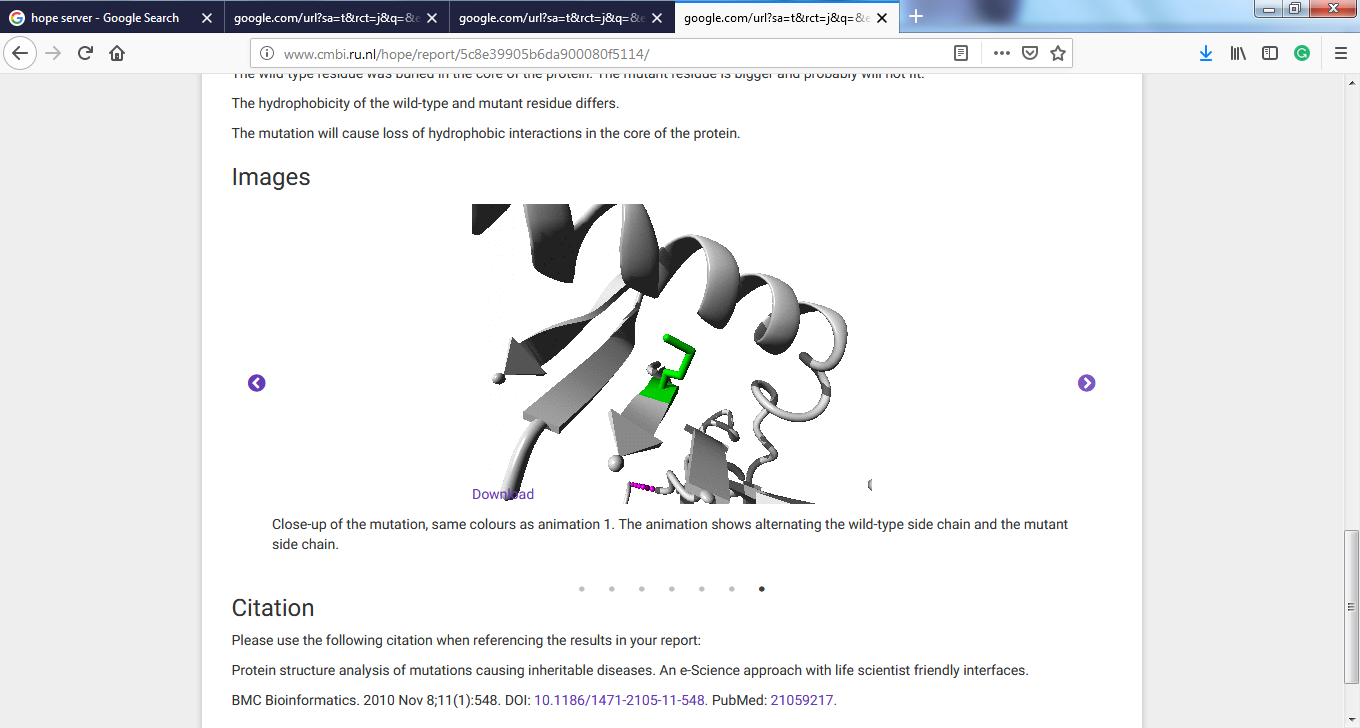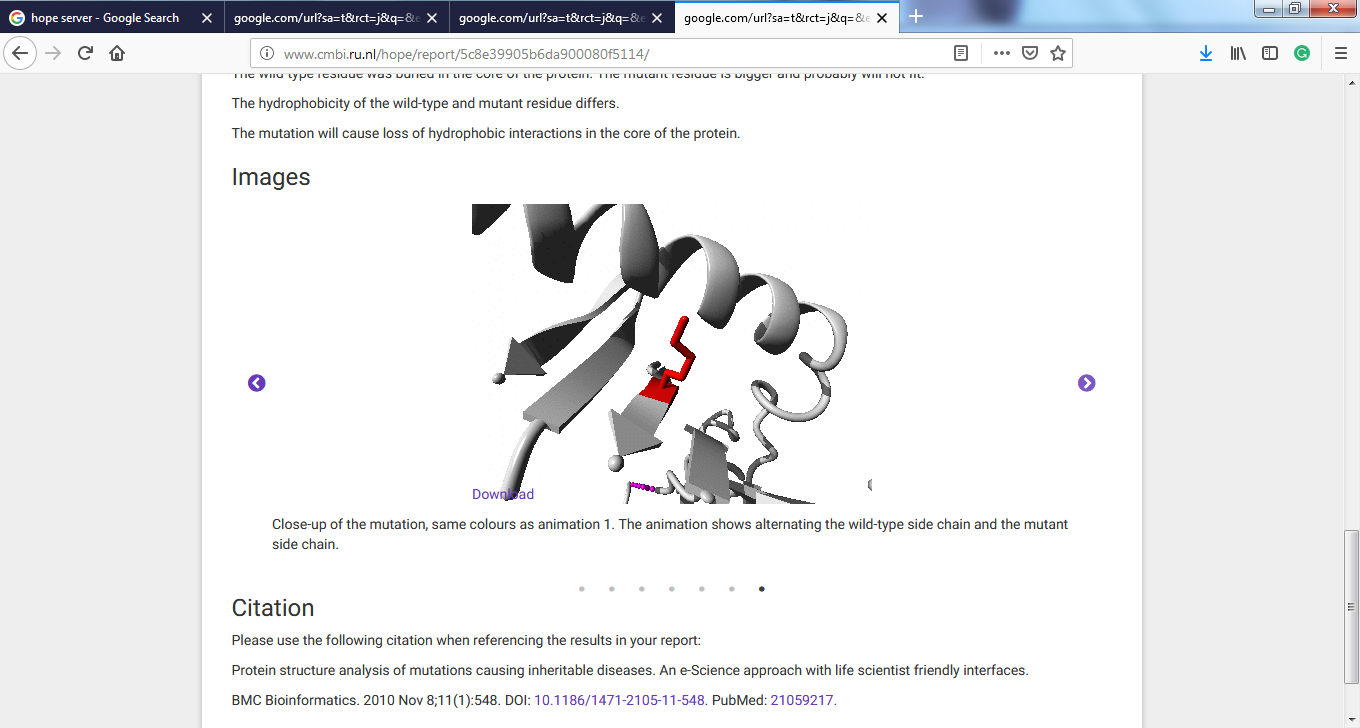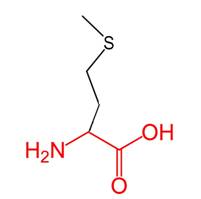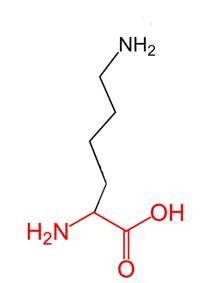 |
|  | **Amino acid properties** | The mutant residue is bigger than the wild-type residue.  The wild-type residue was buried in the core of the protein. The mutant residue is bigger and probably will not fit.  The wild-type amino acid has a neutral charge while the mutant amino acid has a positive charge.  The mutant residue introduces a charge in a buried residue which can lead to protein folding problems.  The wild-type residue is more hydrophobic than the mutant residue.  The hydrophobic interactions in the core of the protein will lose due to the mutation. | | | |
|  | **Structure** | The mutation is located within a stretch of residues annotated in UniProt as a special region: Alpha-1. The diversities in residue characteristics can distort this region and distort its function. | - | The mutation is located within a stretch of residues annotated in UniProt as a special region: Alpha-1. The diversities in residue characteristics can distort this region and distort its function. | The mutation is located within a stretch of residues annotated in UniProt as a special region: Alpha-1. The diversities in residue characteristics can distort this region and distort its function. |
| **R30S** | **Image** | 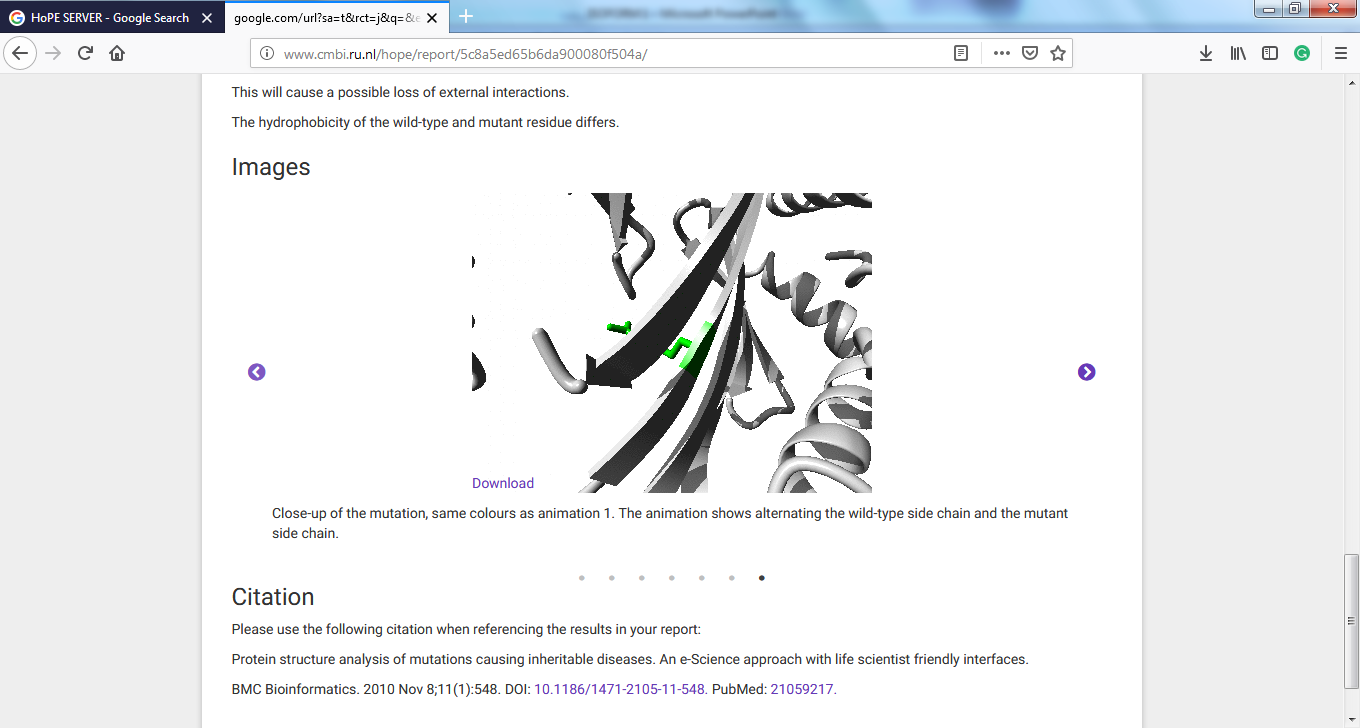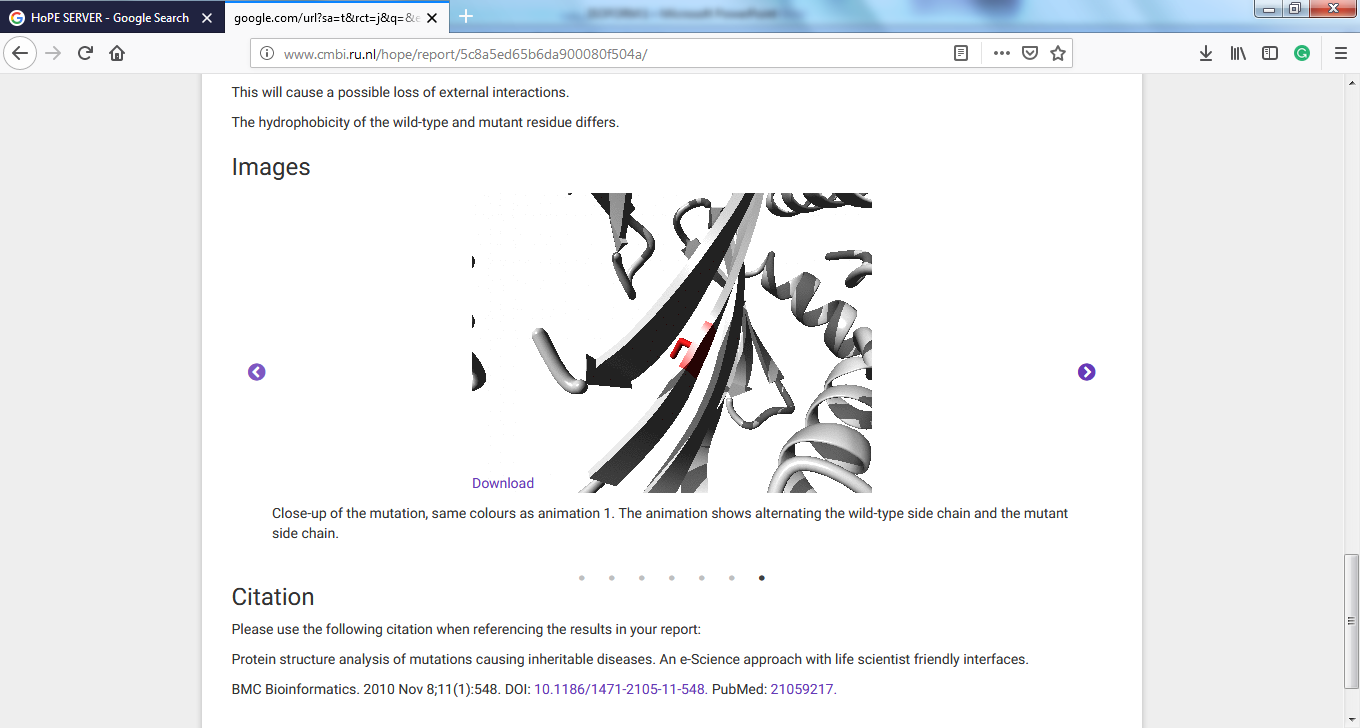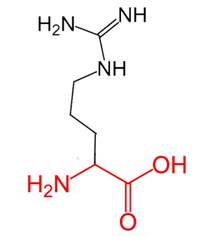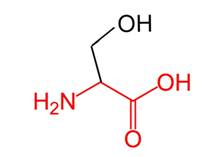 | 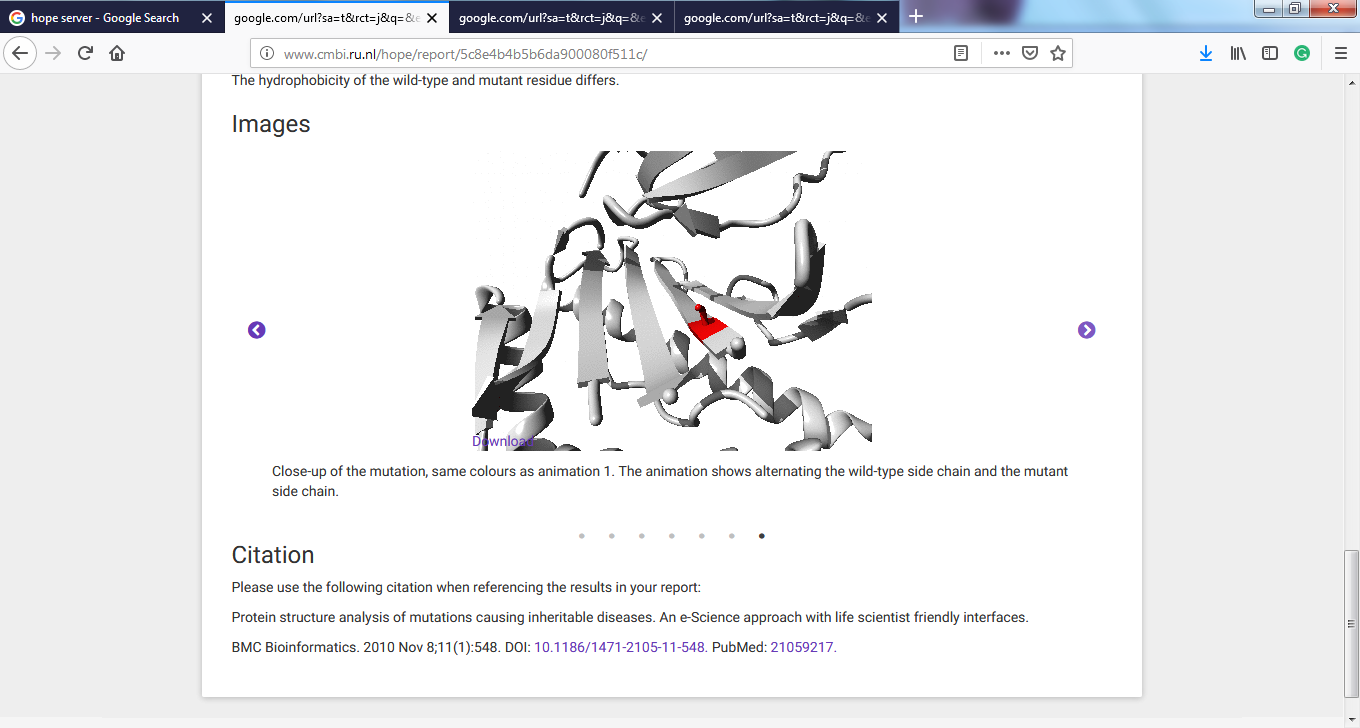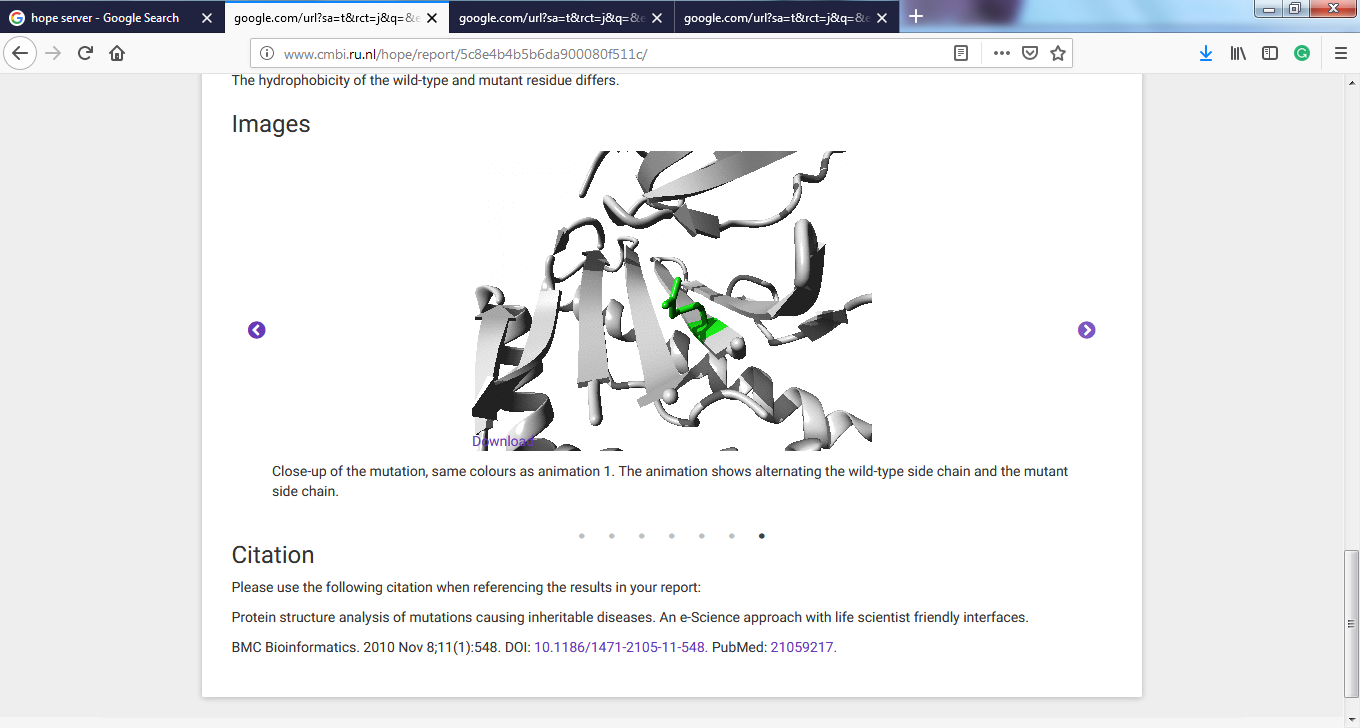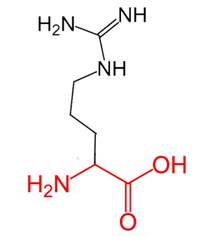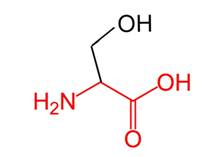 | 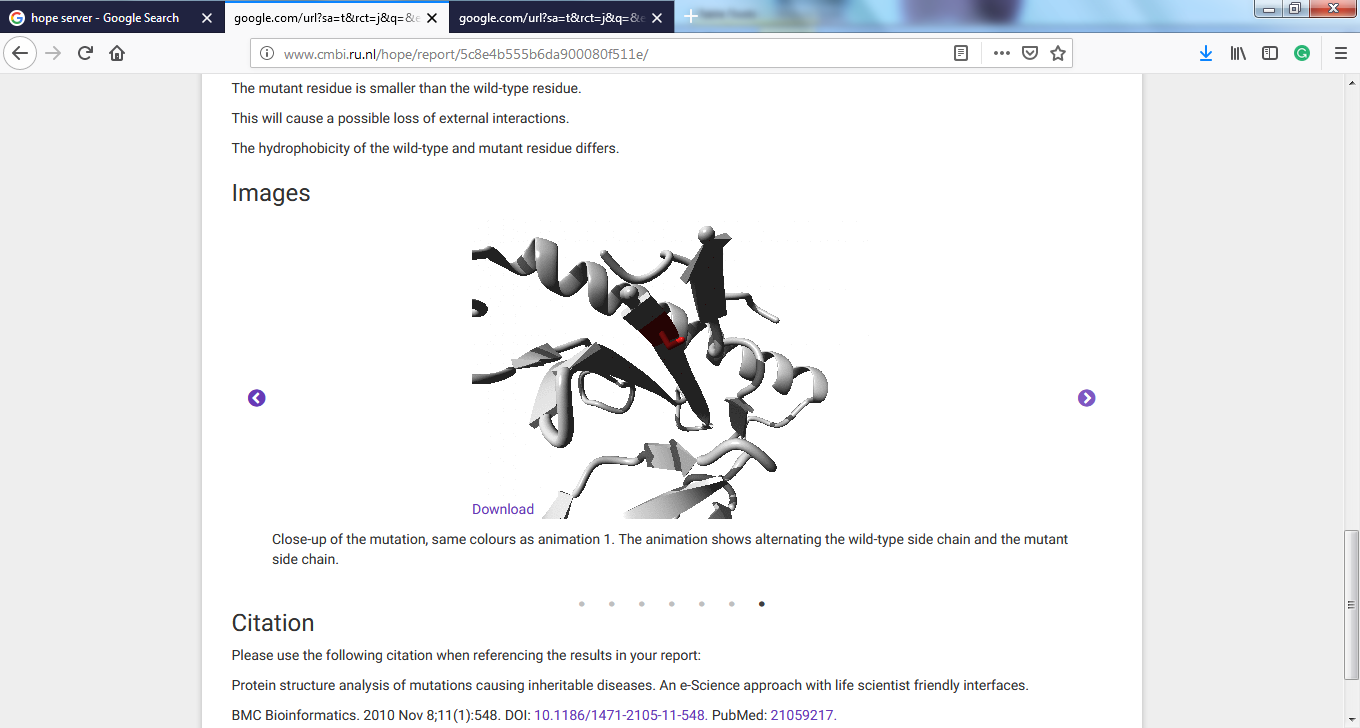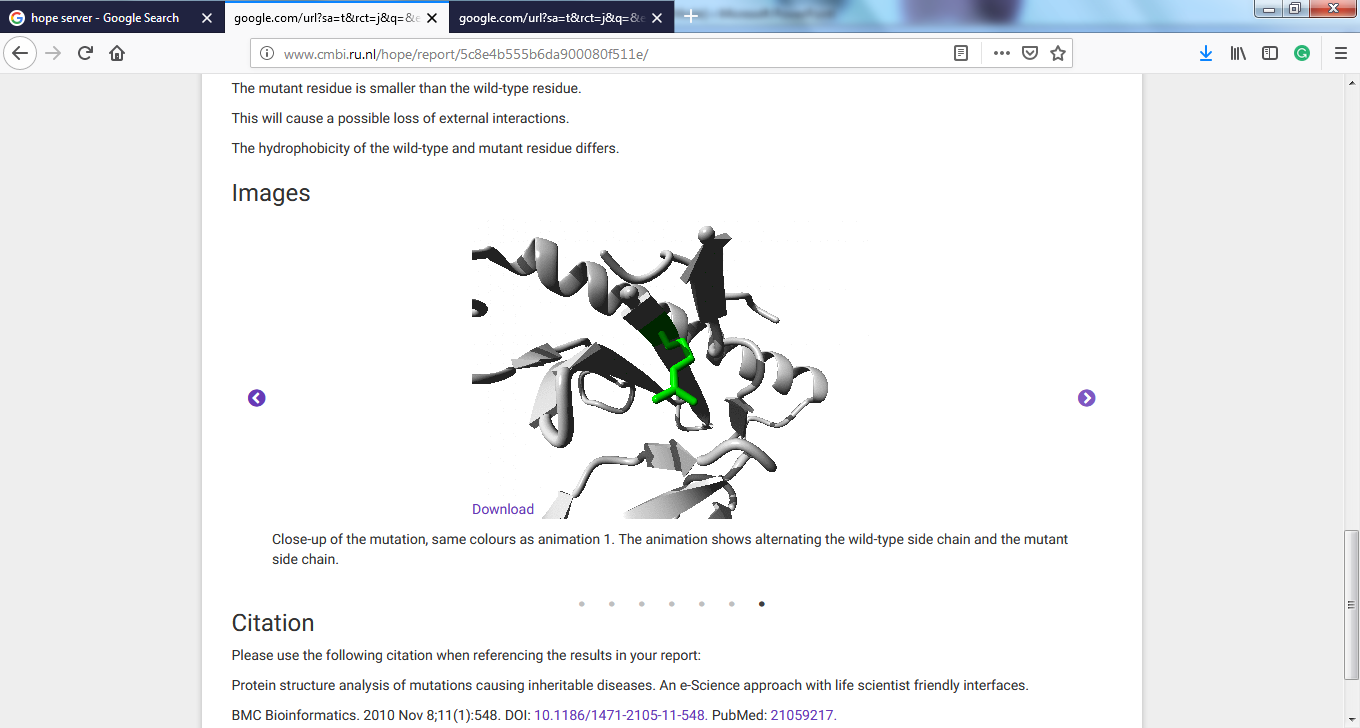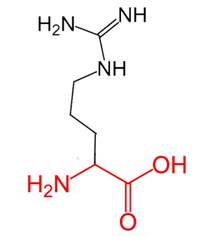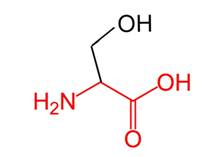 | 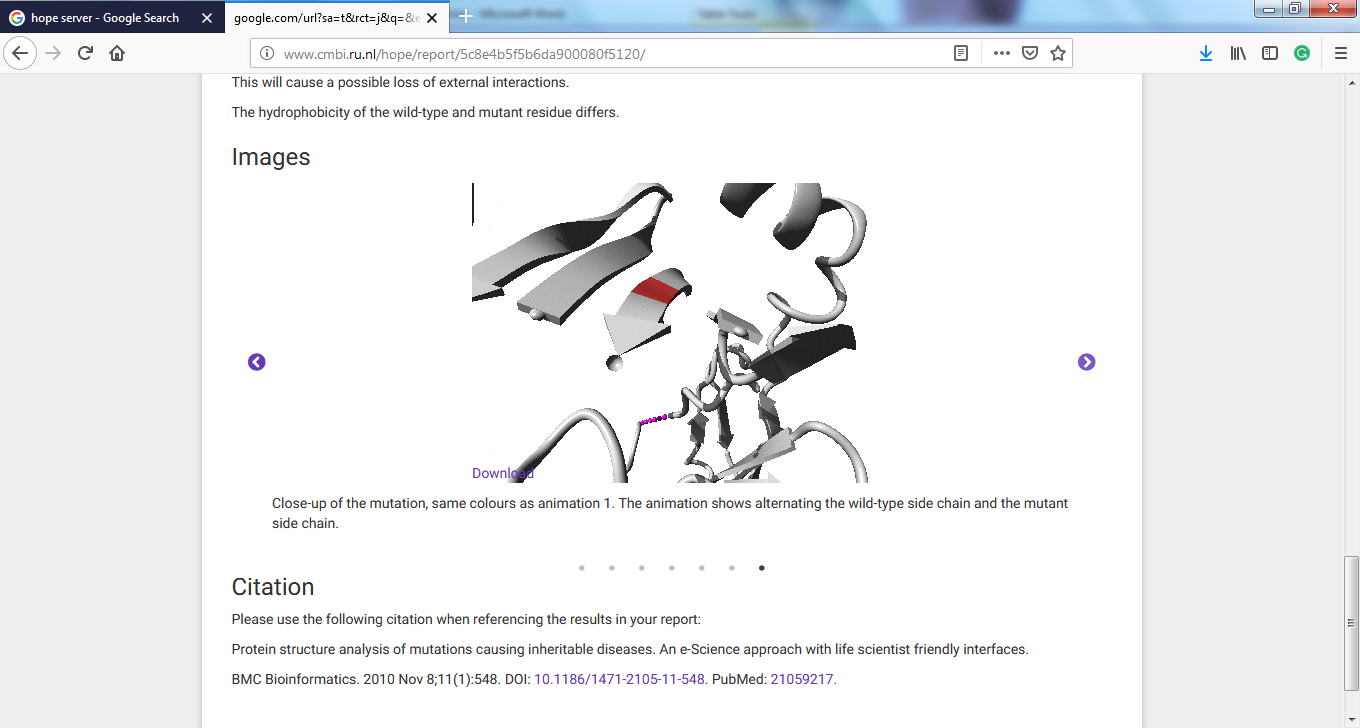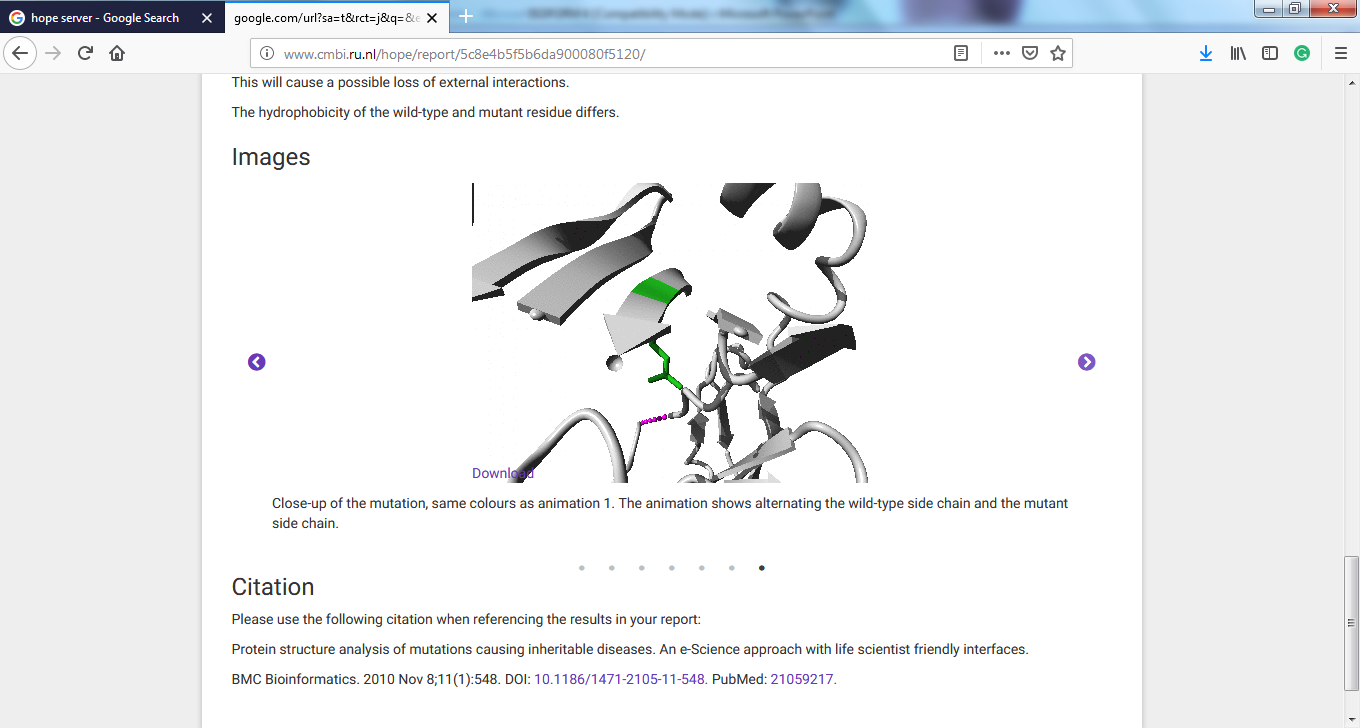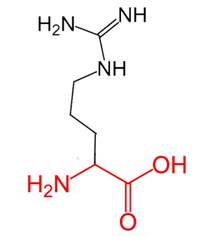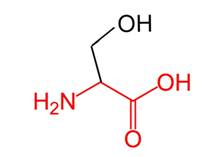 |
|  | **Amino acid properties** | The mutant residue is smaller than the wild-type residue, which will cause a possible loss of external interactions.  The wild-type amino acid has a positive charge while the mutant amino acid has a neutral charge.  The charge of the wild-type residue is lost by this mutation. This can cause loss of interactions with other molecules.  The mutant residue is more hydrophobic than the wild-type residue. | | | |
|  | **Structure** | The mutation is located within a stretch of residues annotated in UniProt as a special region: Alpha-1. The diversities in residue characteristics can distort this region and distort its function. | - | The mutation is located within a stretch of residues annotated in UniProt as a special region: Alpha-1. The diversities in residue characteristics can distort this region and distort its function. | The mutation is located within a stretch of residues annotated in UniProt as a special region: Alpha-1. The diversities in residue characteristics can distort this region and distort its function. |
| **Y51C** | **Image** | 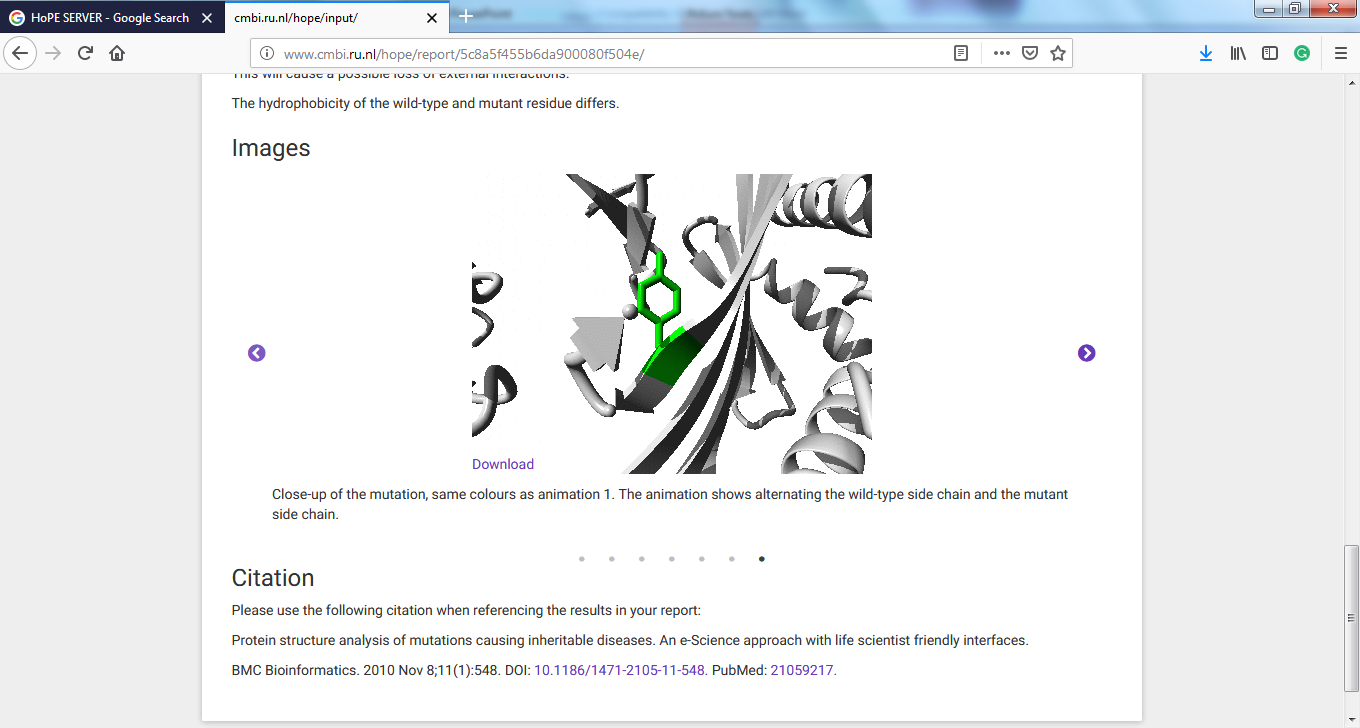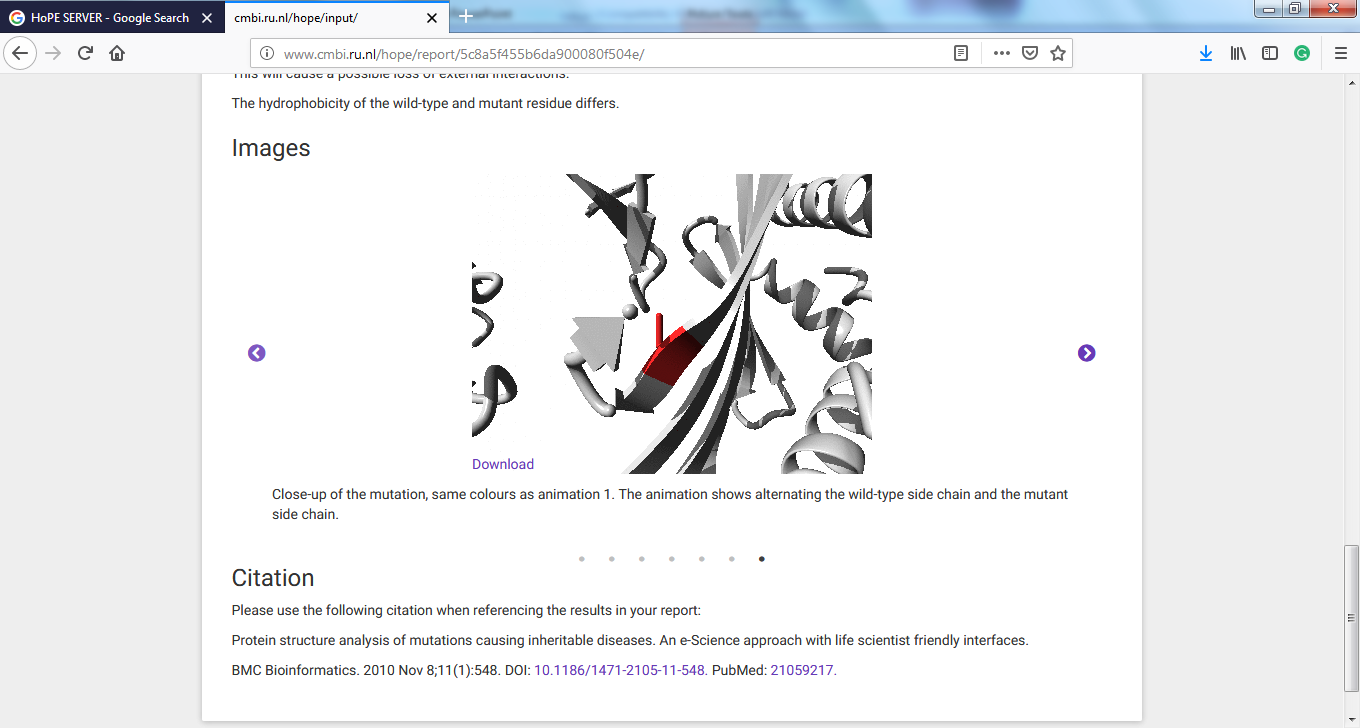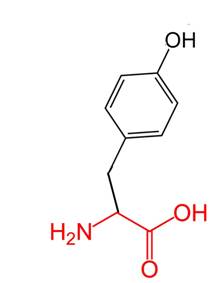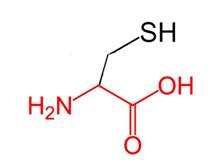 | 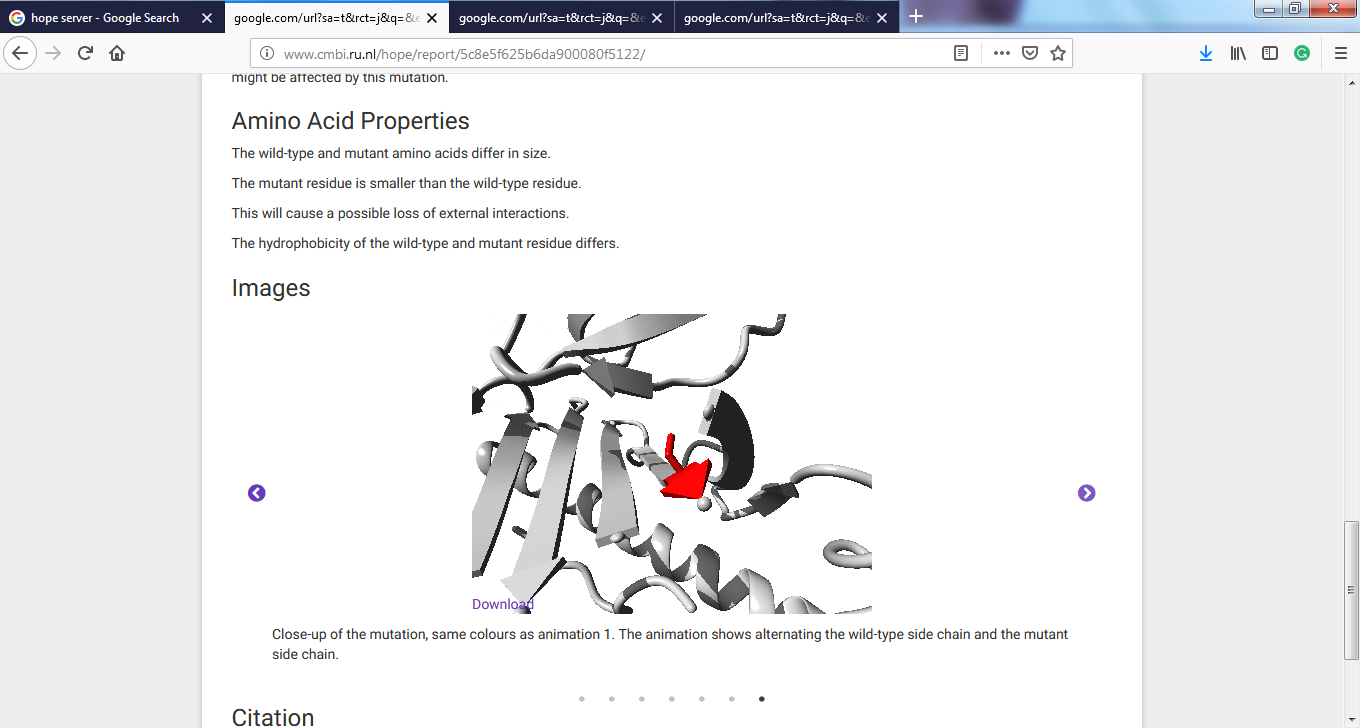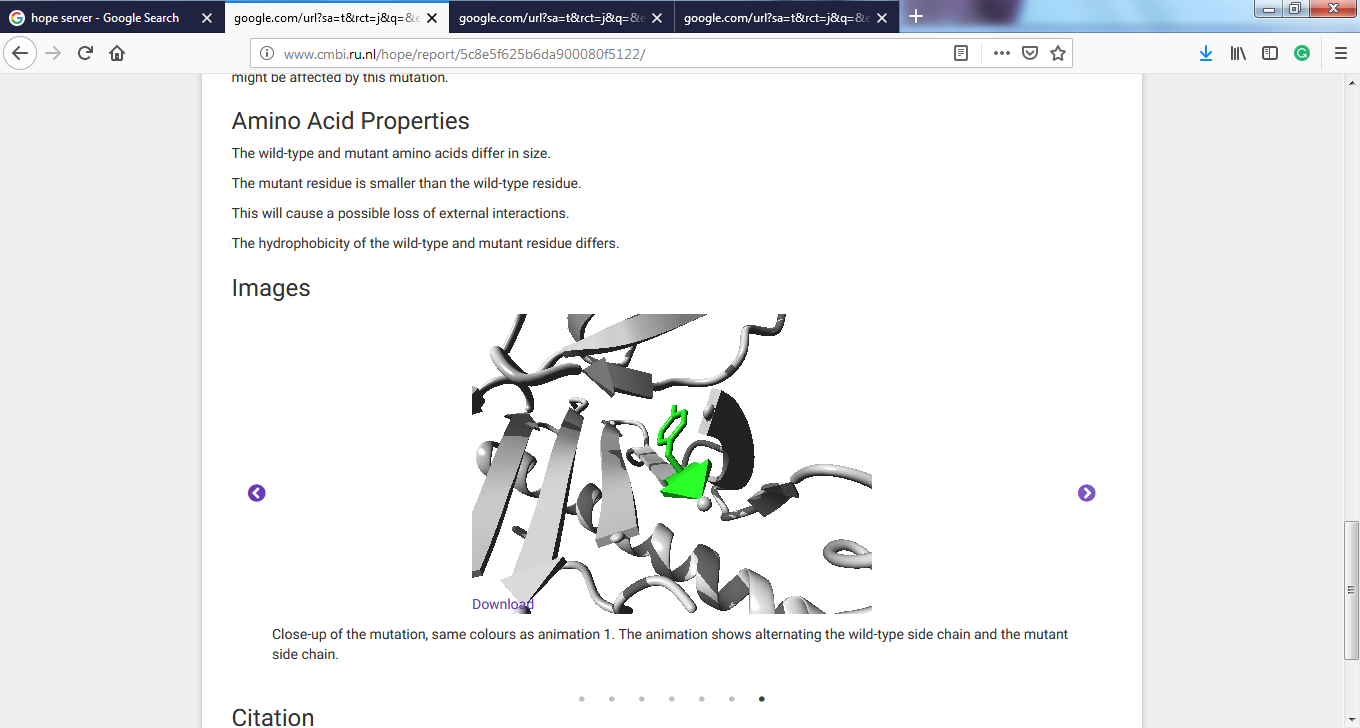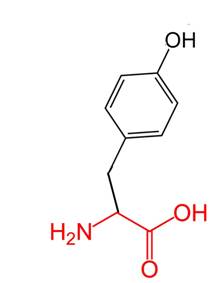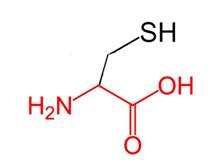 | 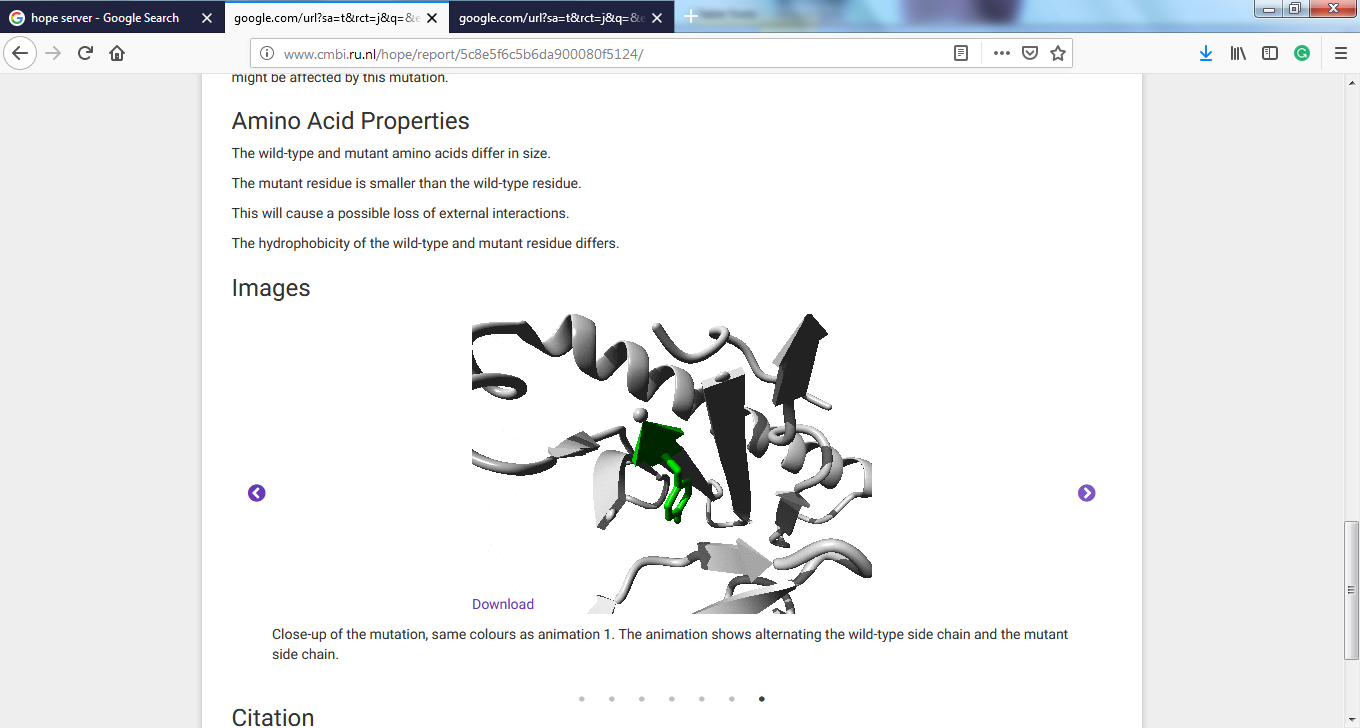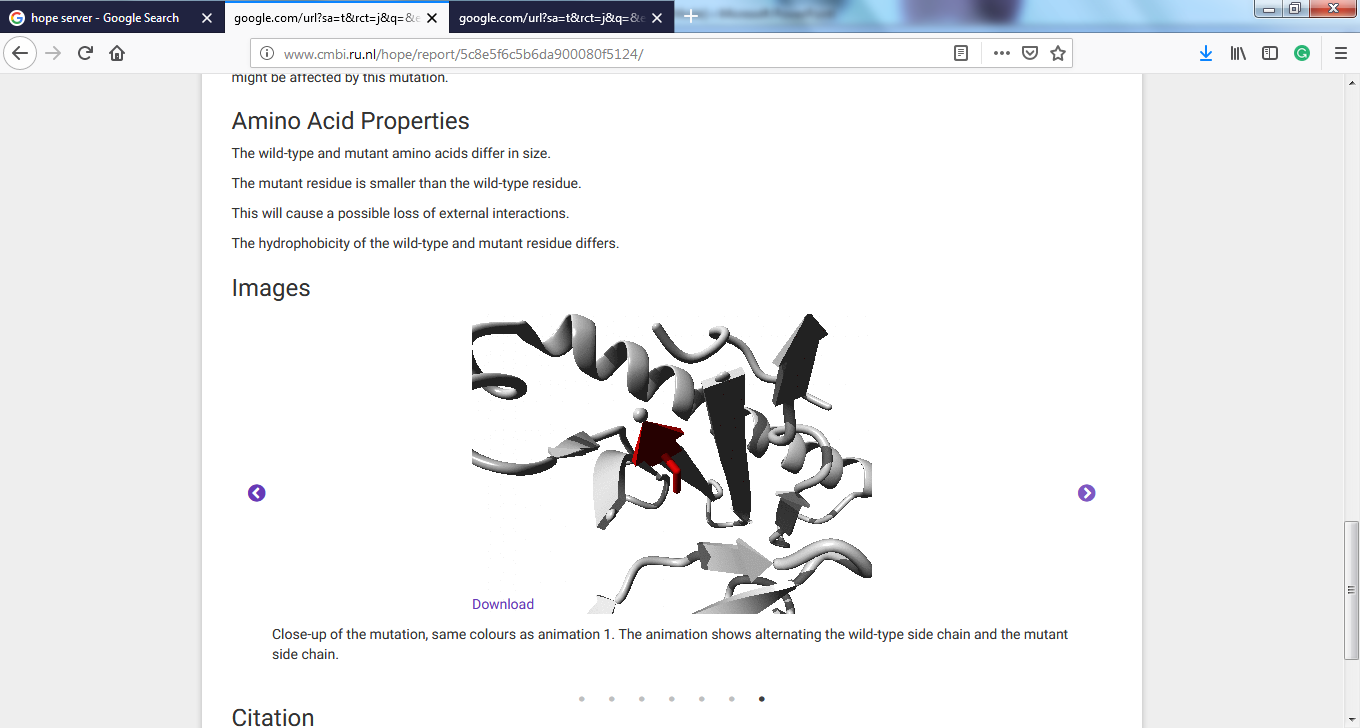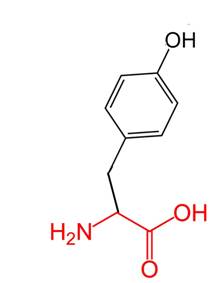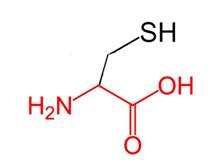 | 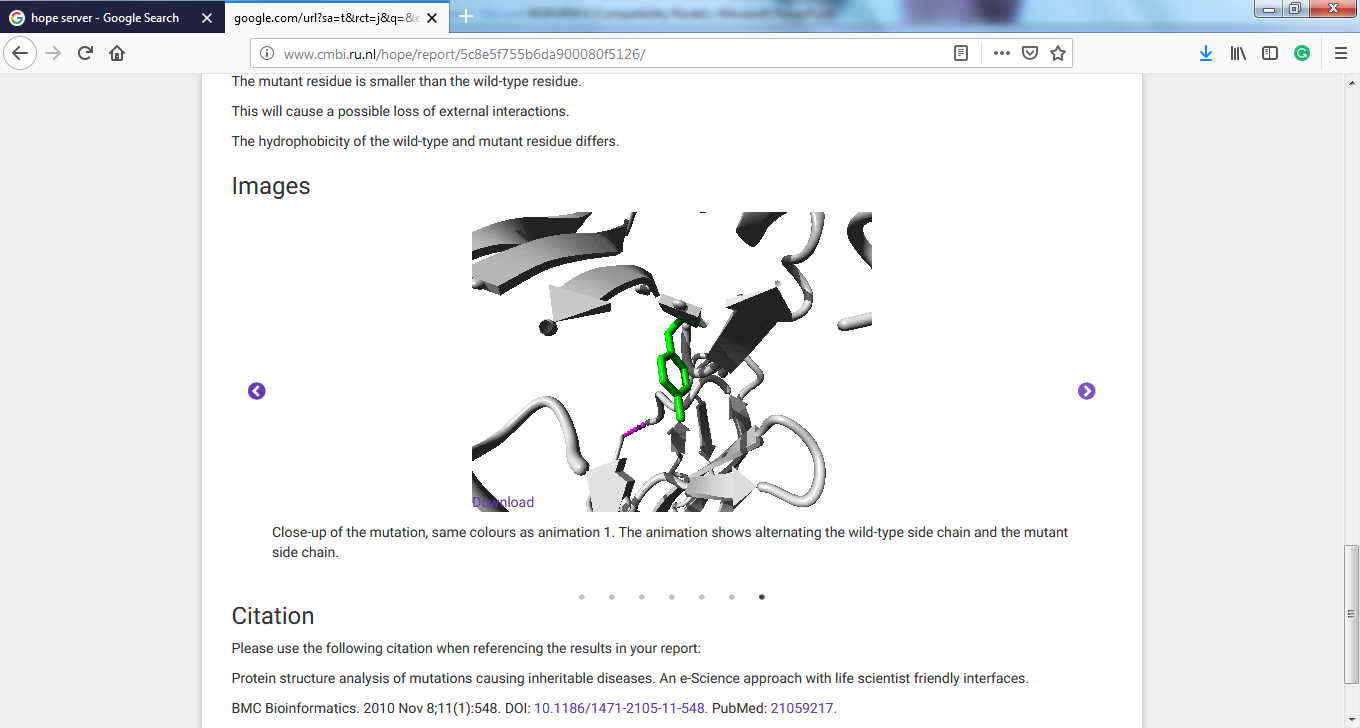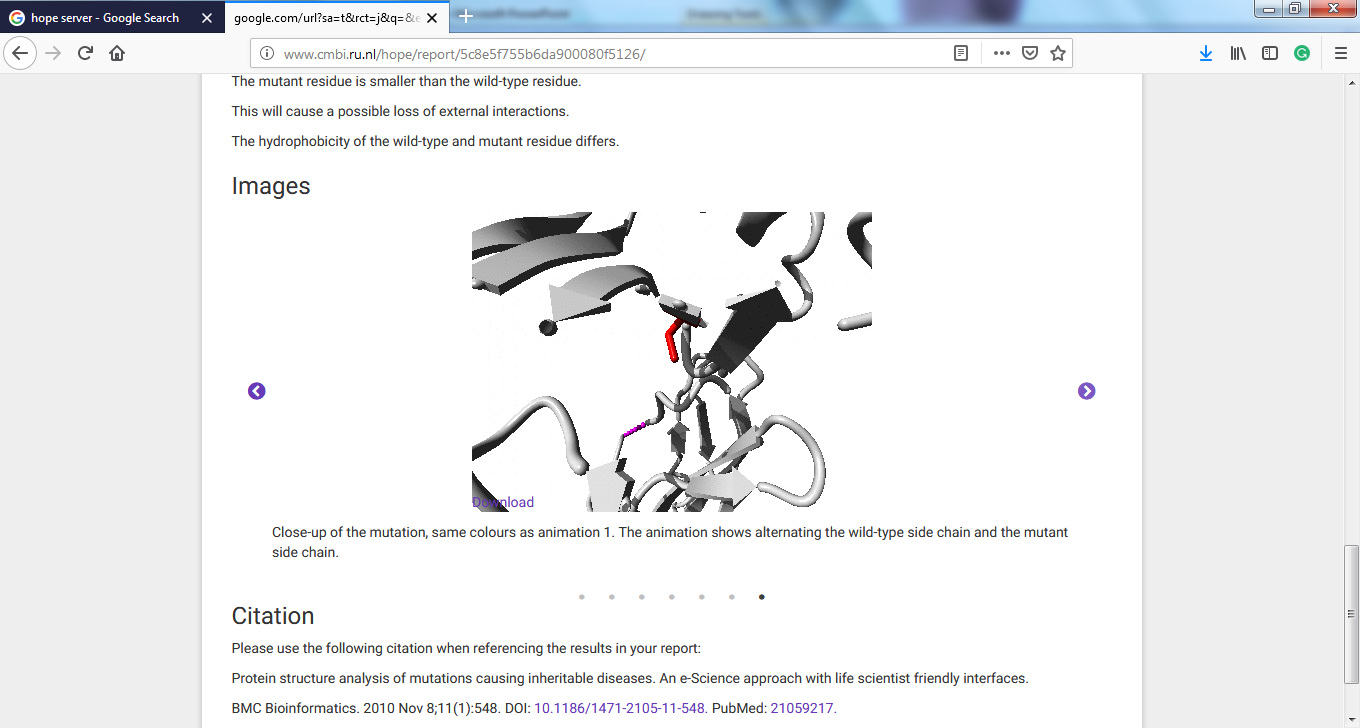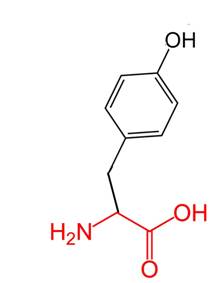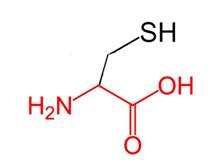 |
|  | **Amino acid properties** | The mutant residue is smaller than the wild-type residue, which will cause a possible loss of external interactions**.**  The mutant residue is more hydrophobic than the wild-type residue. | | | |
|  | **Structure** | The mutation is located within a stretch of residues annotated in UniProt as a special region: Alpha-1. The diversities in residue characteristics can distort this region and distort its function. | In the 3D-structure can be seen that the wild-type residue is located in its preferred secondary structure, a β-strand. The mutant residue prefers to be in another secondary structure; therefore the local conformation will be slightly destabilized. | The mutation is located within a stretch of residues annotated in UniProt as a special region: Alpha-1. The diversities in residue characteristics can distort this region and distort its function. | The mutation is located within a stretch of residues annotated in UniProt as a special region: Alpha-1. The diversities in residue characteristics can distort this region and distort its function. |
| **D53N** | **Image** | 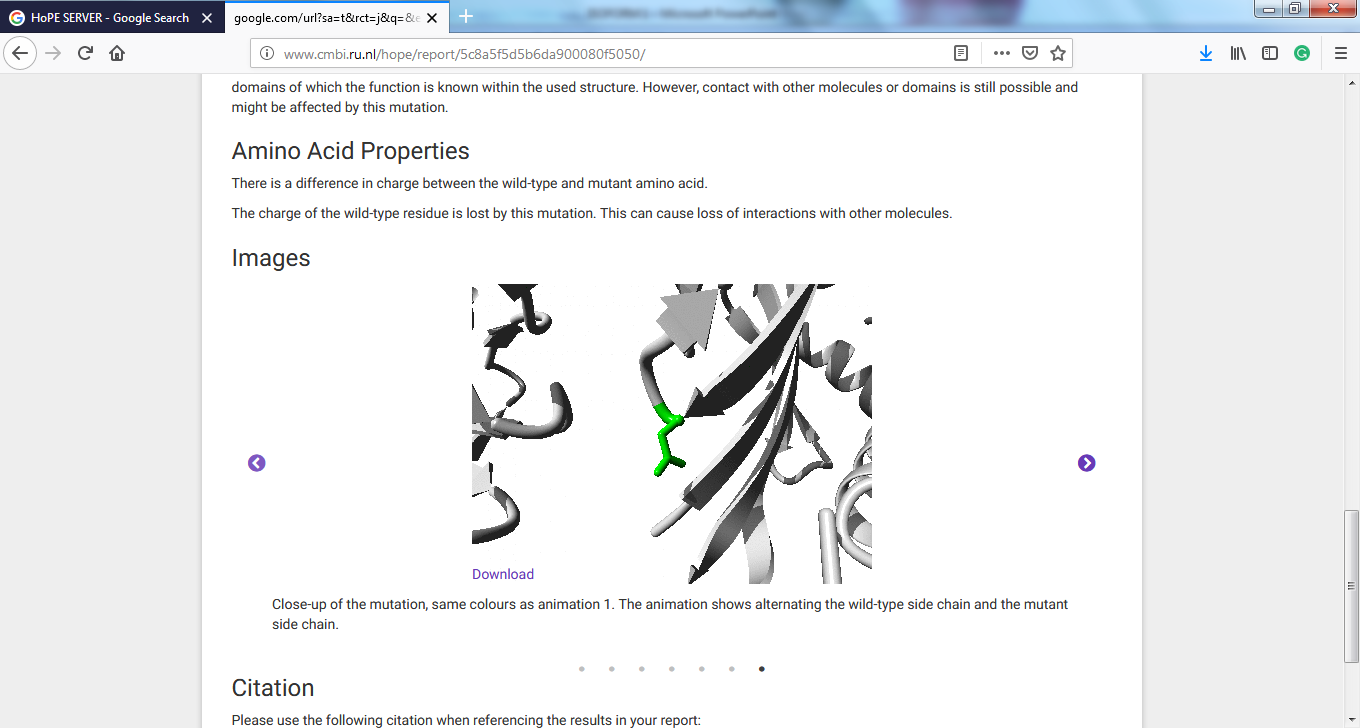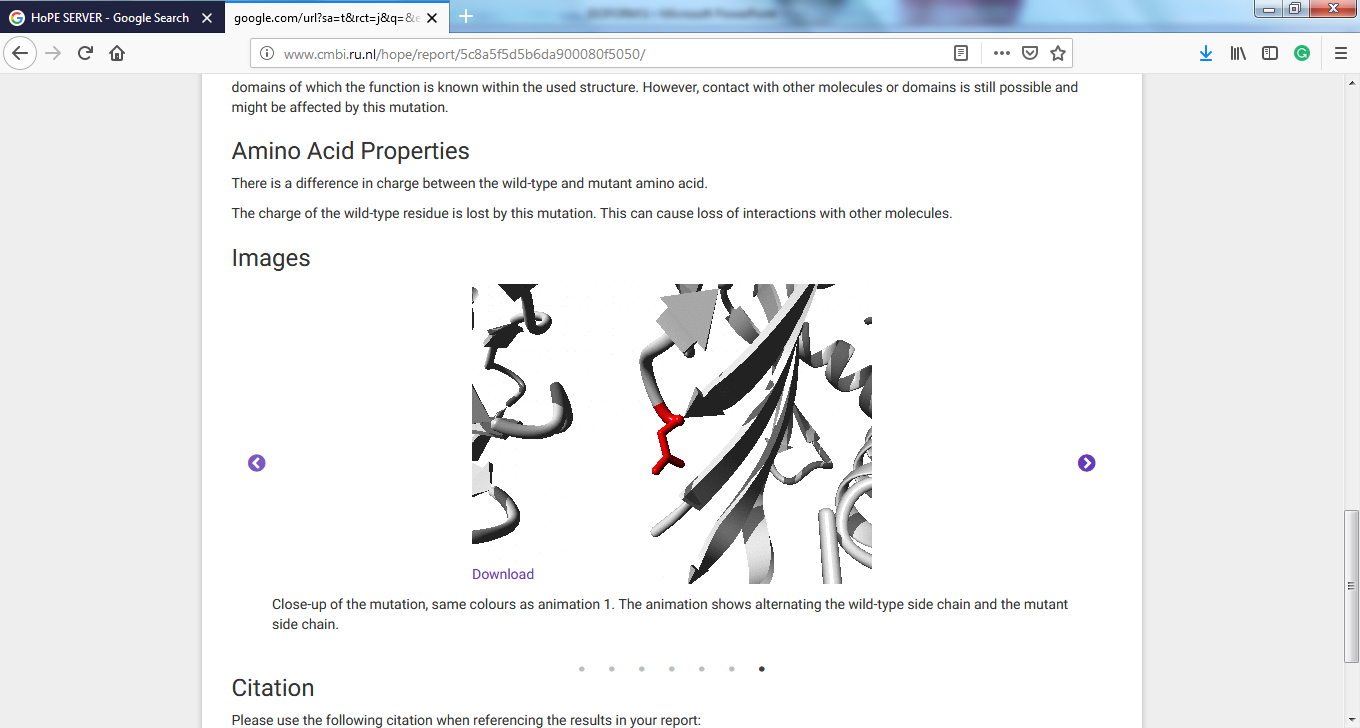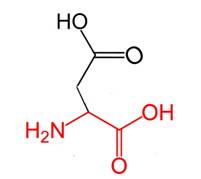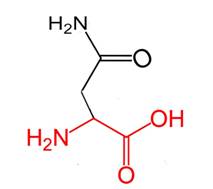 | 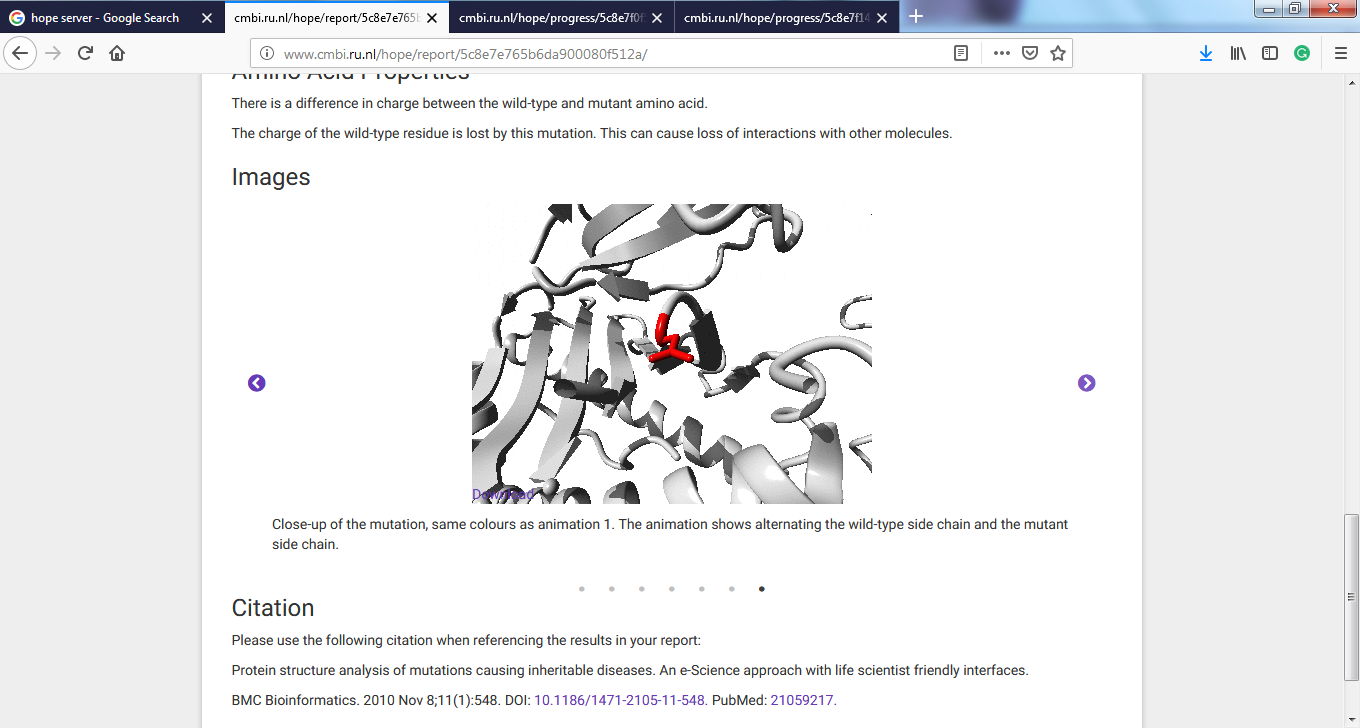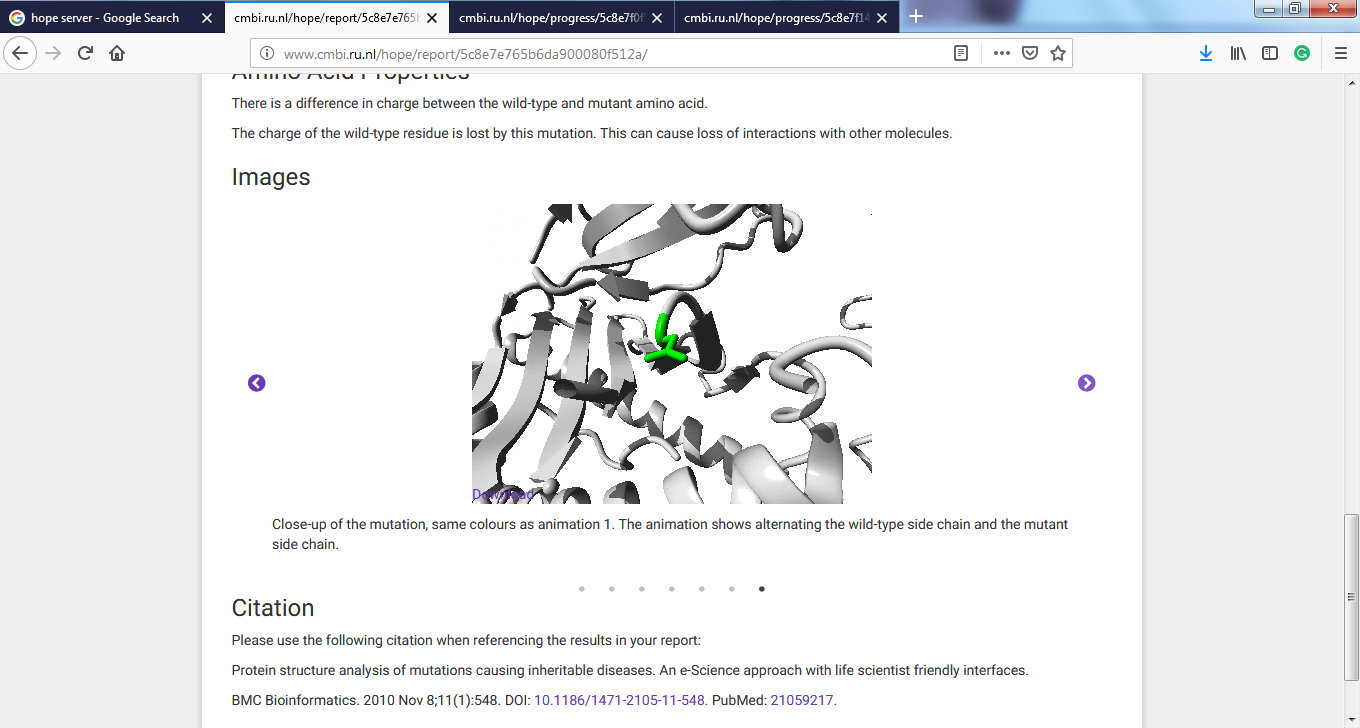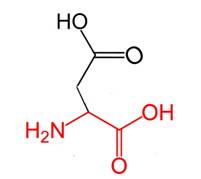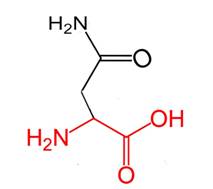 | 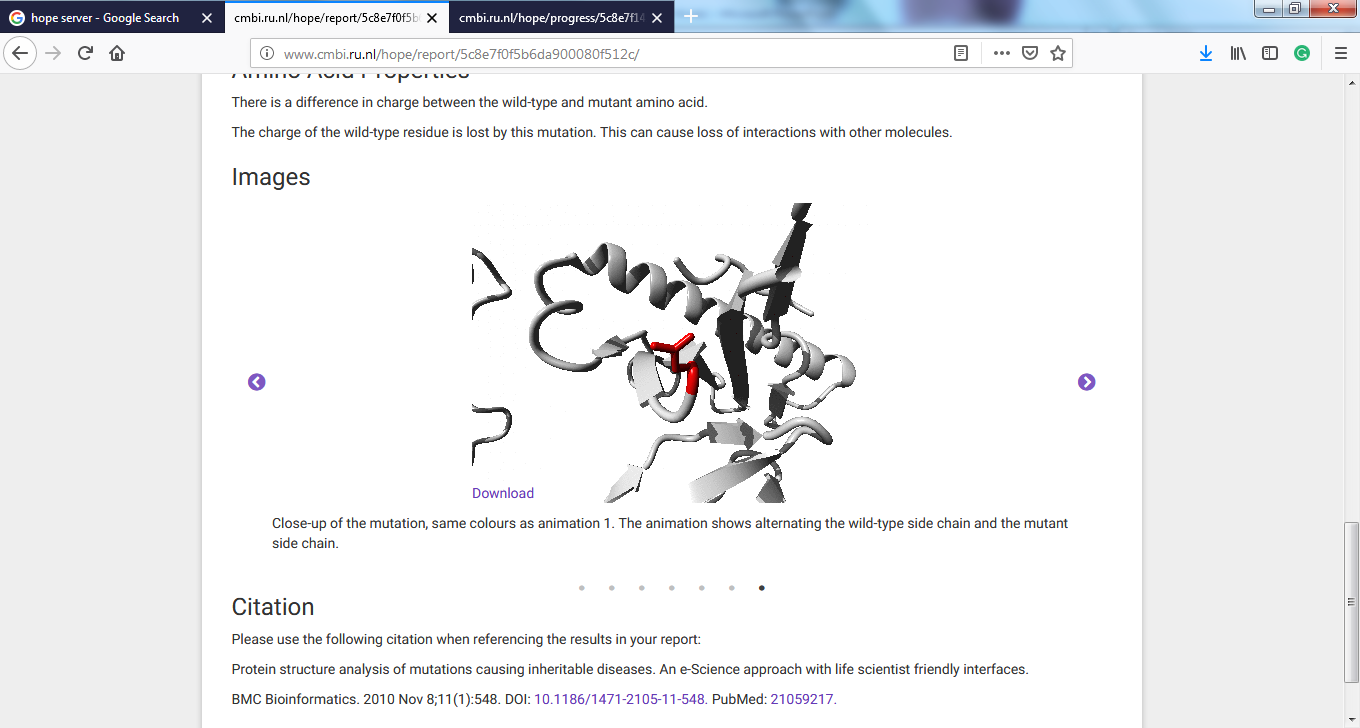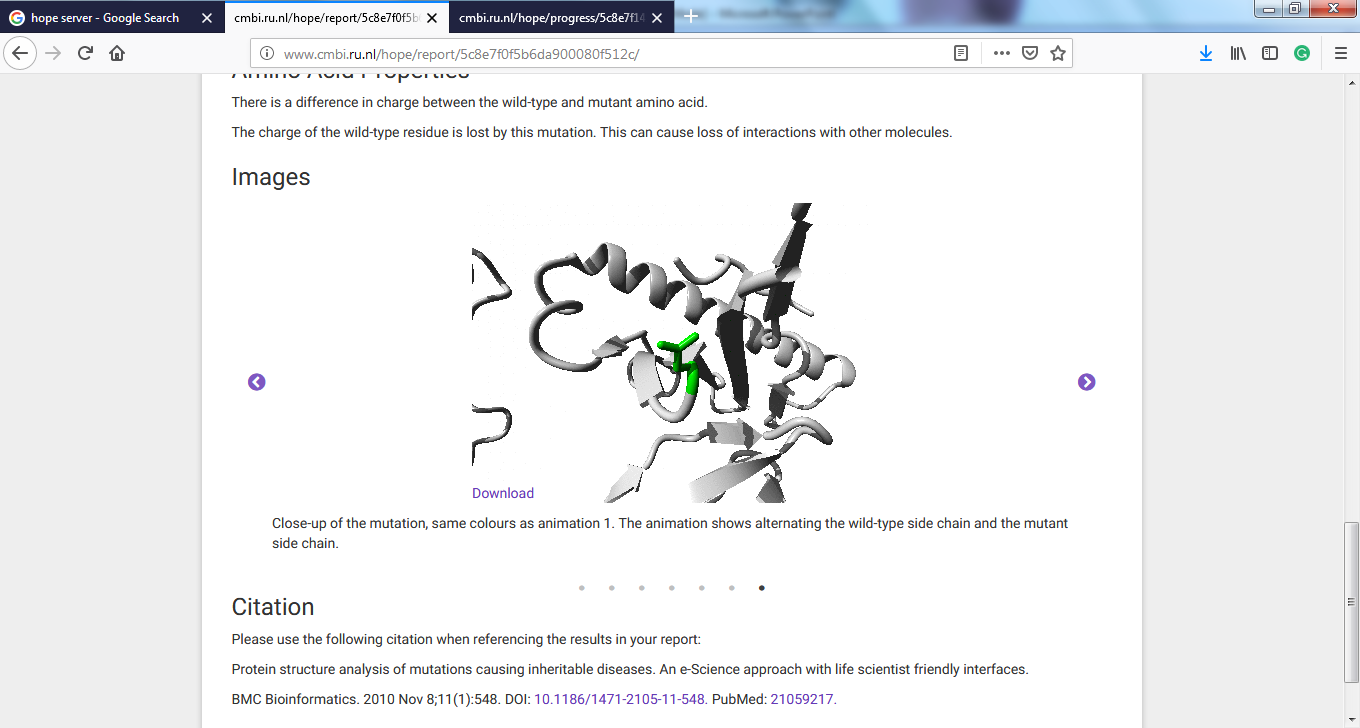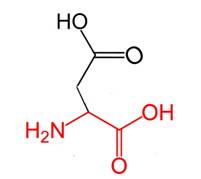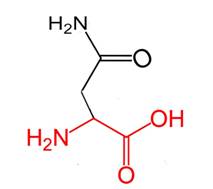 | 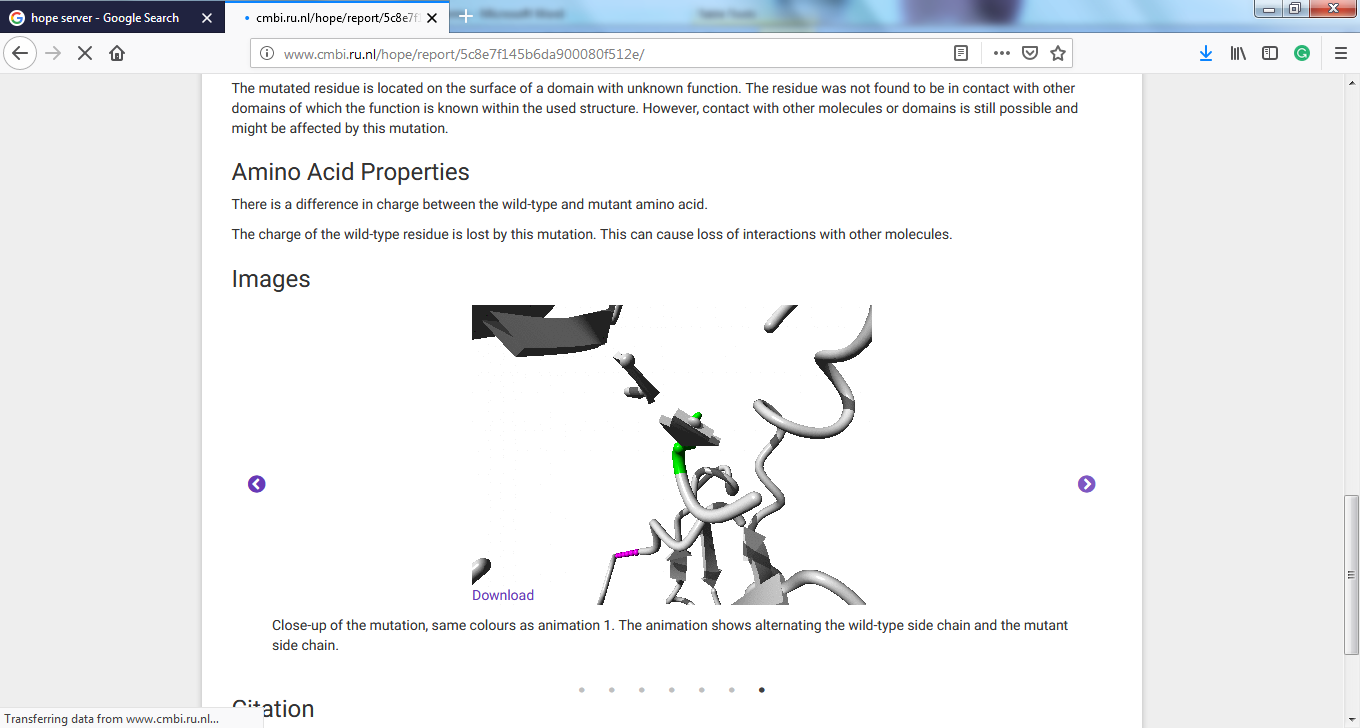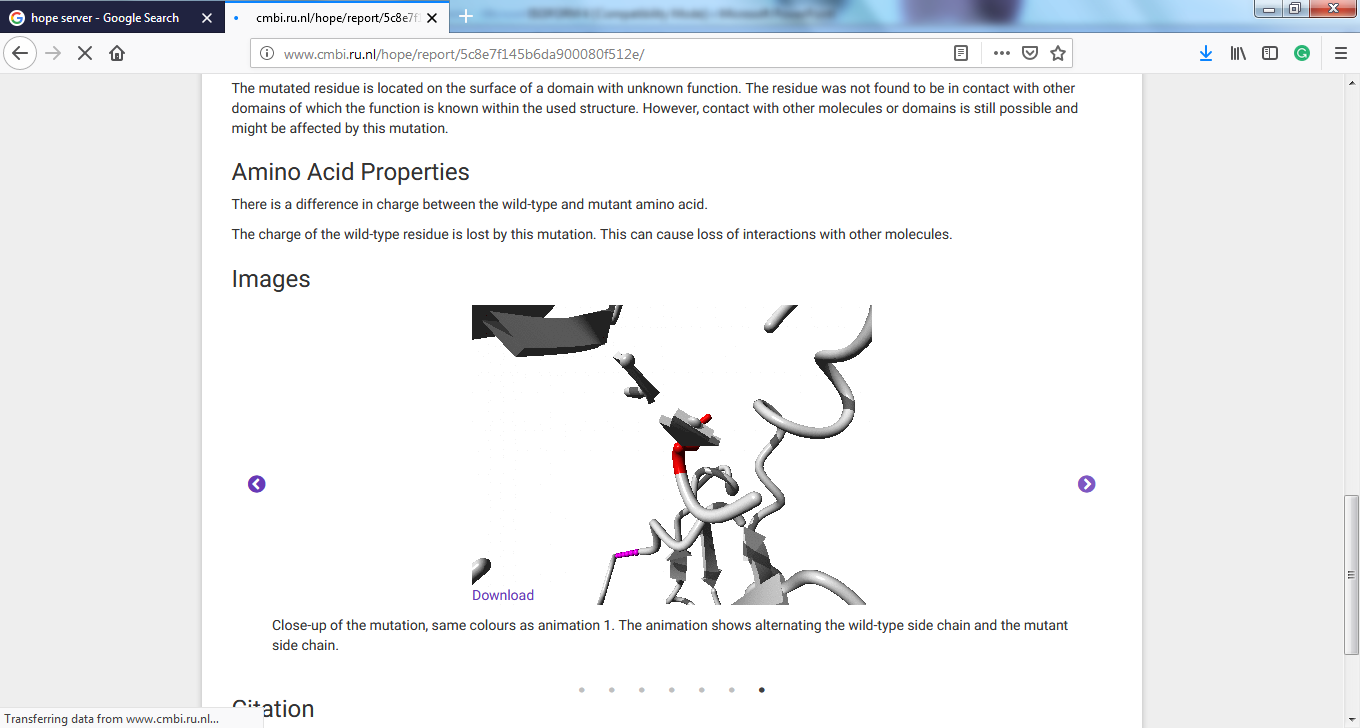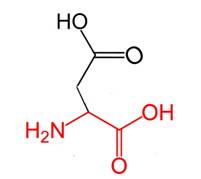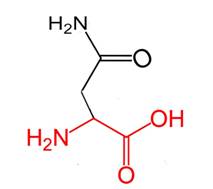 |
|  | **Amino acid properties** | The wild-type residue charge was negative; the mutant residue charge is neutral.  The charge of the wild-type residue is lost by this mutation. This can cause loss of interactions with other molecules. | | | |
|  | **Structure** | The mutation is located within a stretch of residues annotated in UniProt as a special region: Alpha-1. The diversities in residue characteristics can distort this region and distort its function. | - | The mutation is located within a stretch of residues annotated in UniProt as a special region: Alpha-1. The diversities in residue characteristics can distort this region and distort its function. | The mutation is located within a stretch of residues annotated in UniProt as a special region: Alpha-1. The diversities in residue characteristics can distort this region and distort its function.. |
| **D53Y** | **Image** | 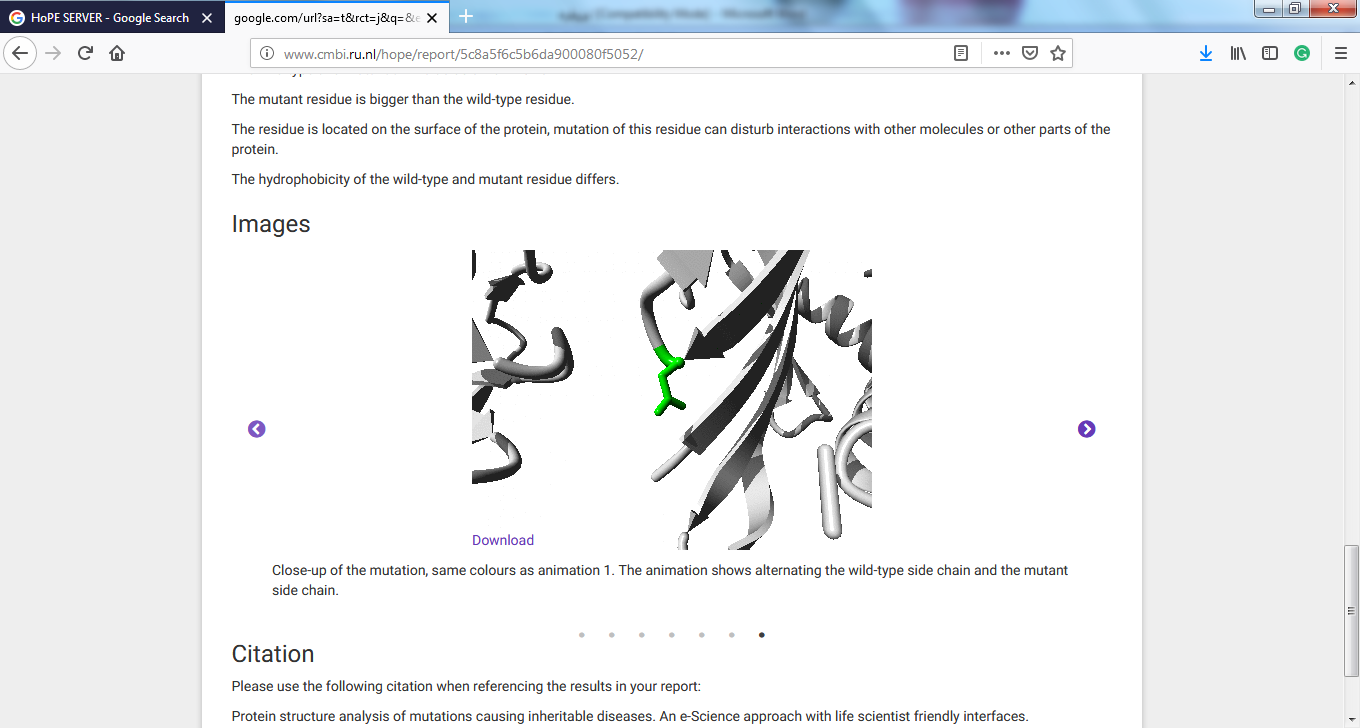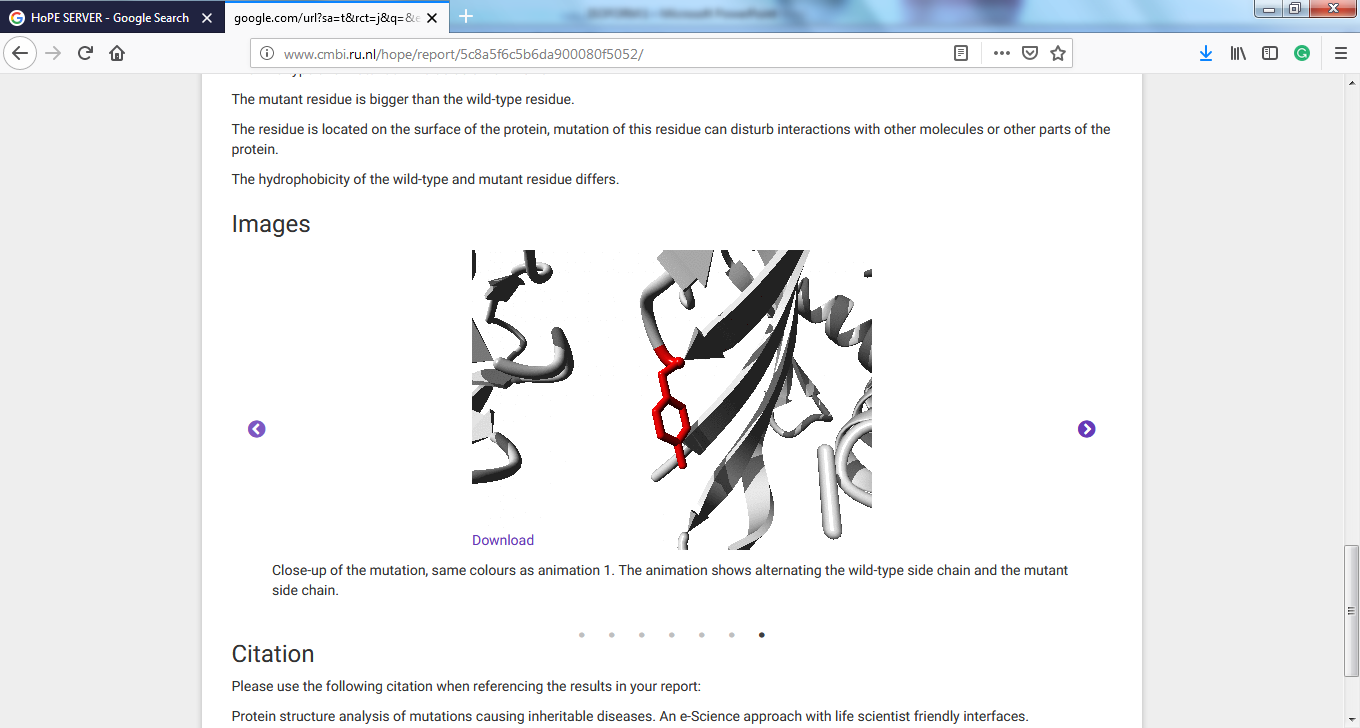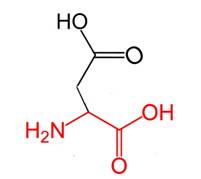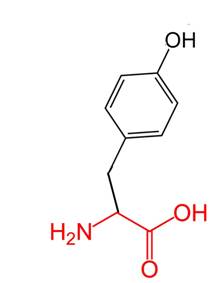 | 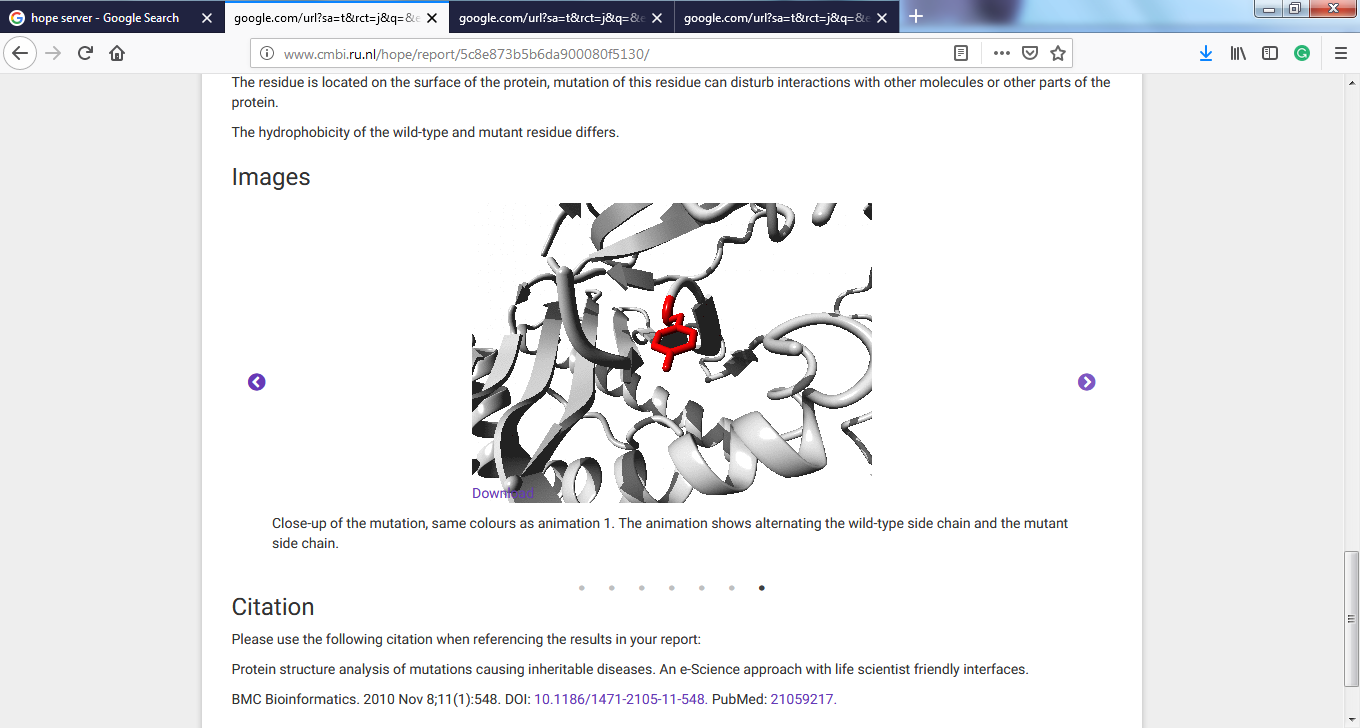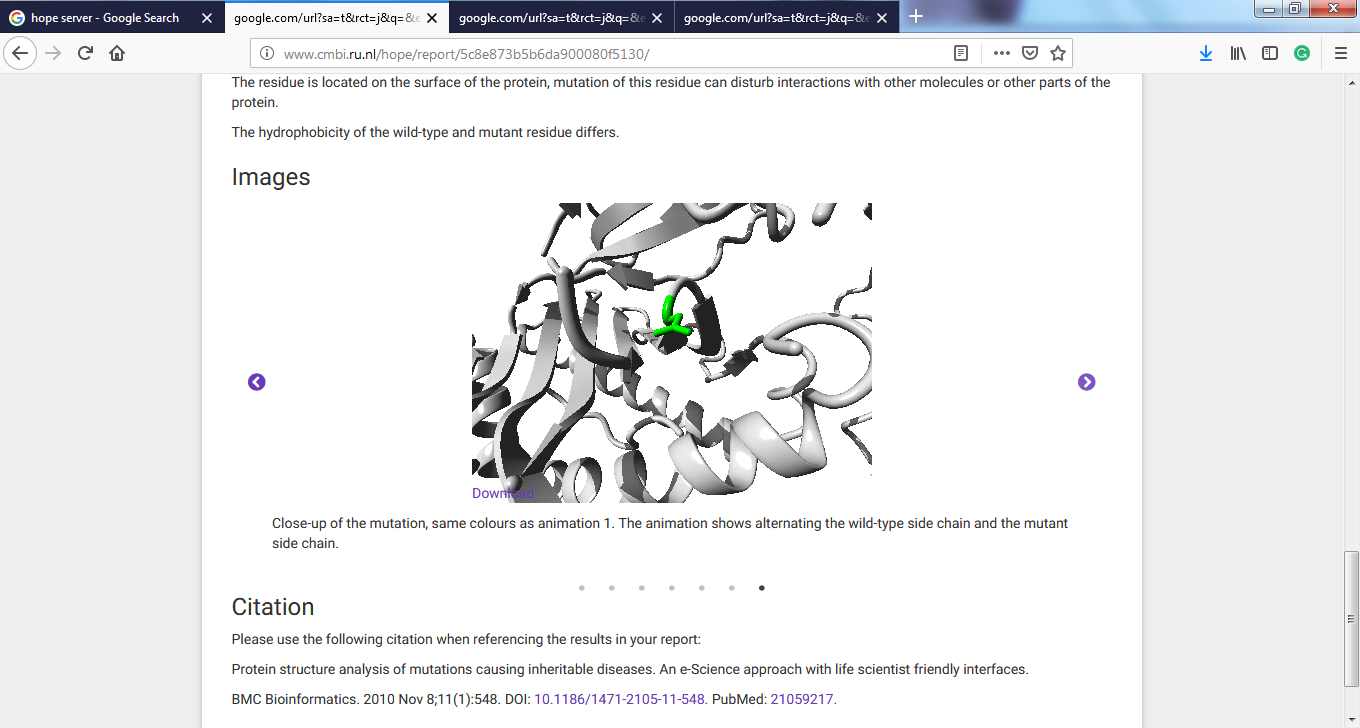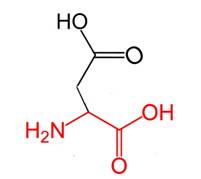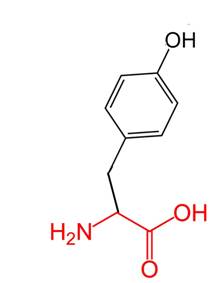 | 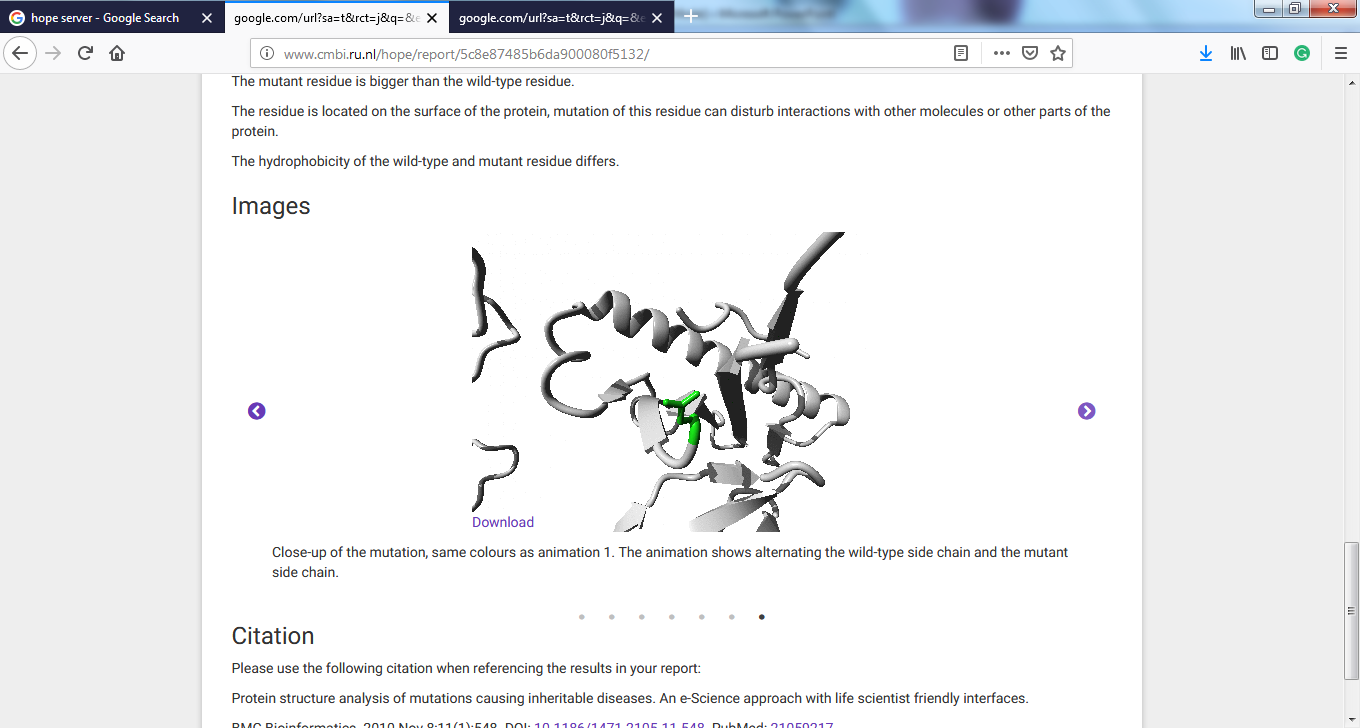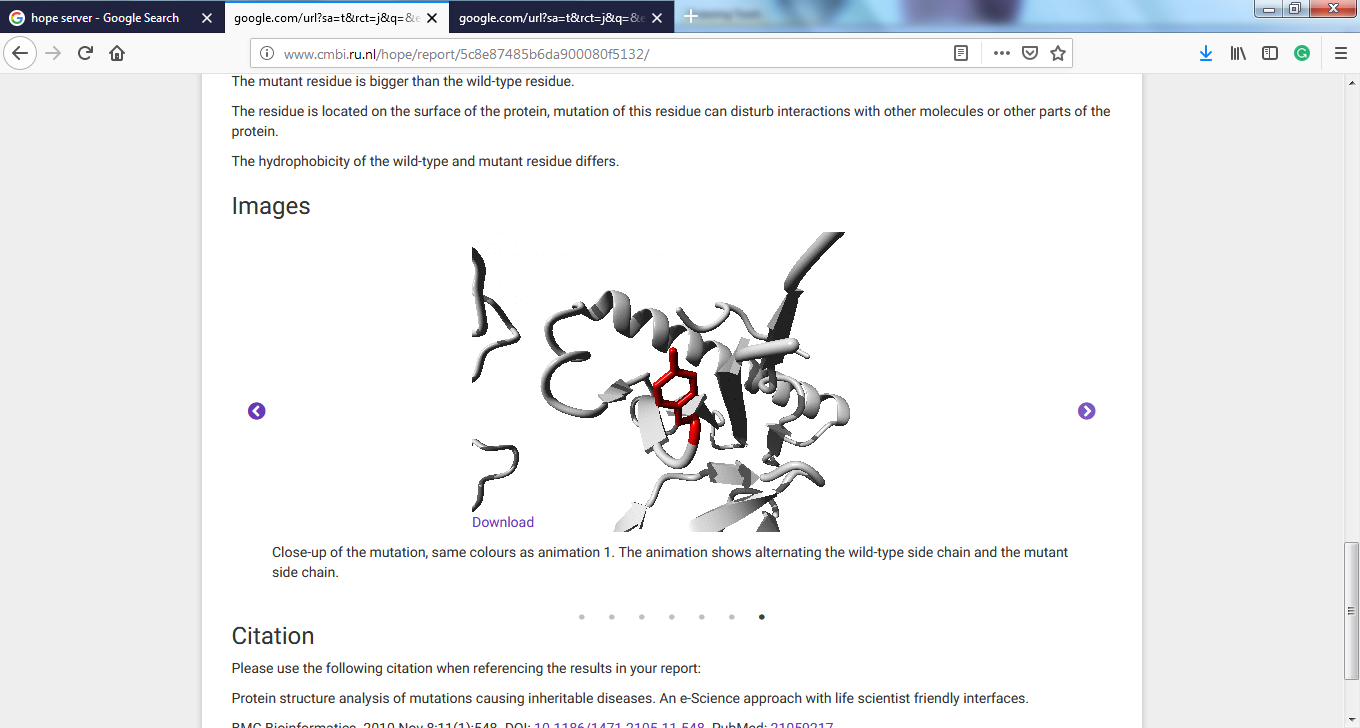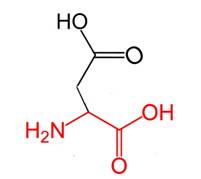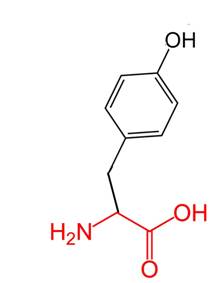 | 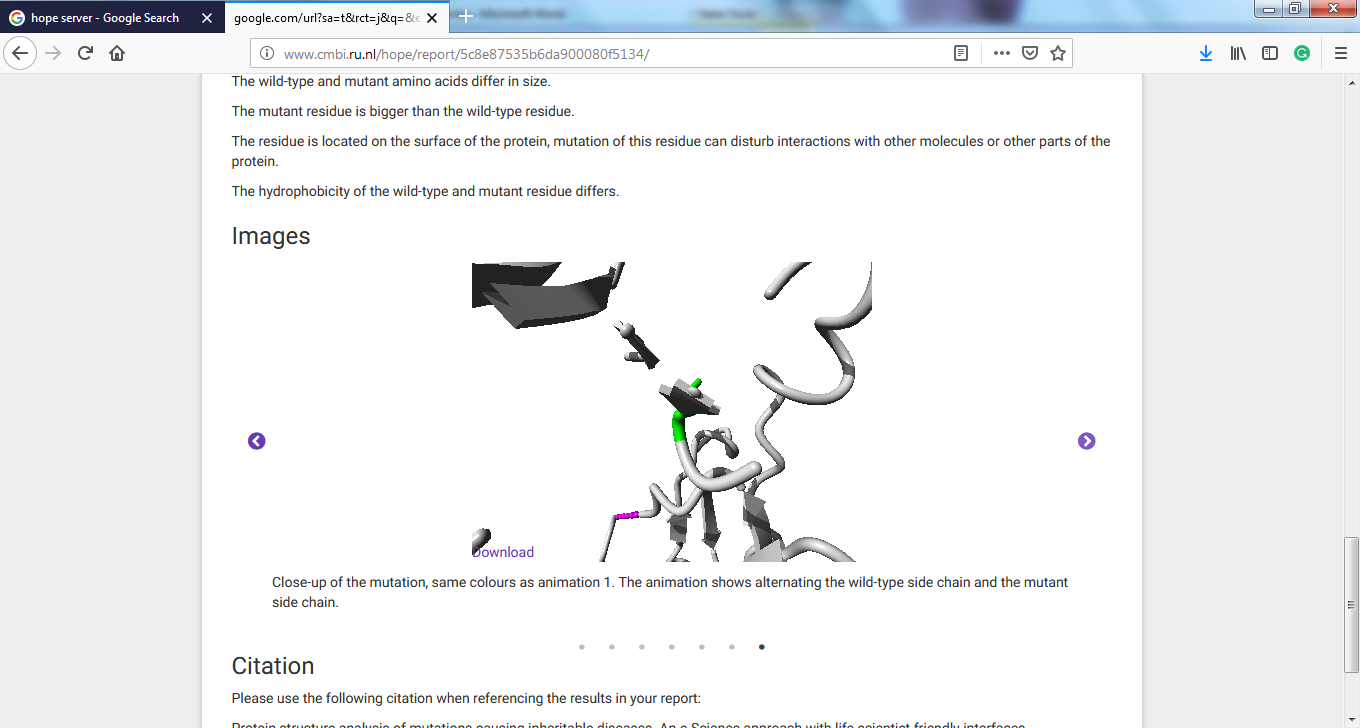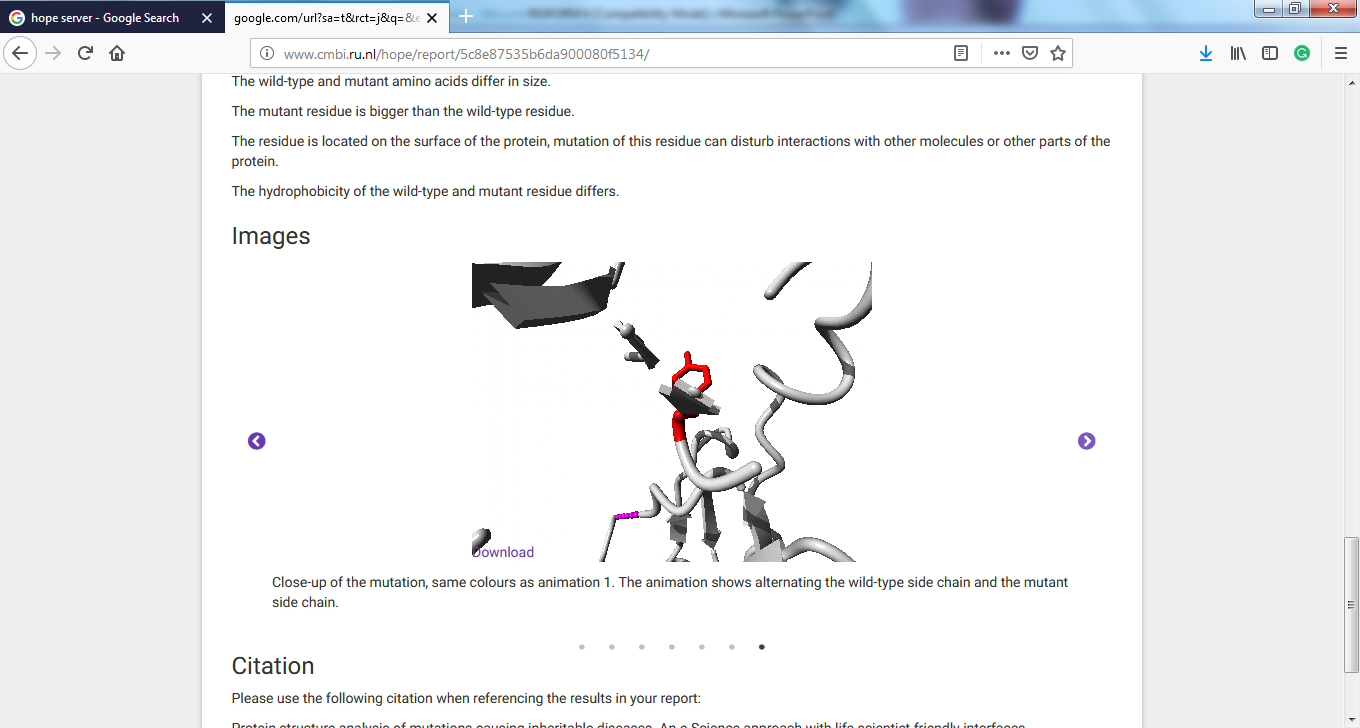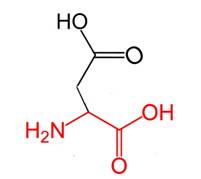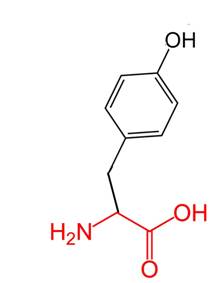 |
|  | **Amino acid properties** | The mutant residue is bigger than the wild-type residue.  The amino acid is located on the surface of the protein; mutation of this amino acid can distort interactions with other molecules or other sections of the protein.  The wild-type residue charge was negative; the mutant residue charge is neutral.  The charge of the wild-type residue is lost by this mutation. This can cause loss of interactions with other molecules.  The mutant residue is more hydrophobic than the wild-type residue. | | | |
|  | **Structure** | The mutation is located within a stretch of residues annotated in UniProt as a special region: Alpha-1. The diversities in residue characteristics can distort this region and distort its function. | In the 3D-structure can be seen that the wild-type residue is located in its preferred secondary structure, a turn. The mutant residue prefers to be in another secondary structure; therefore the local conformation will be slightly destabilized. | The mutation is located within a stretch of residues annotated in UniProt as a special region: Alpha-1. The diversities in residue characteristics can distort this region and distort its function. | The mutation is located within a stretch of residues annotated in UniProt as a special region: Alpha-1. The diversities in residue characteristics can distort this region and distort its function. |
| **D54V** | **Image** | 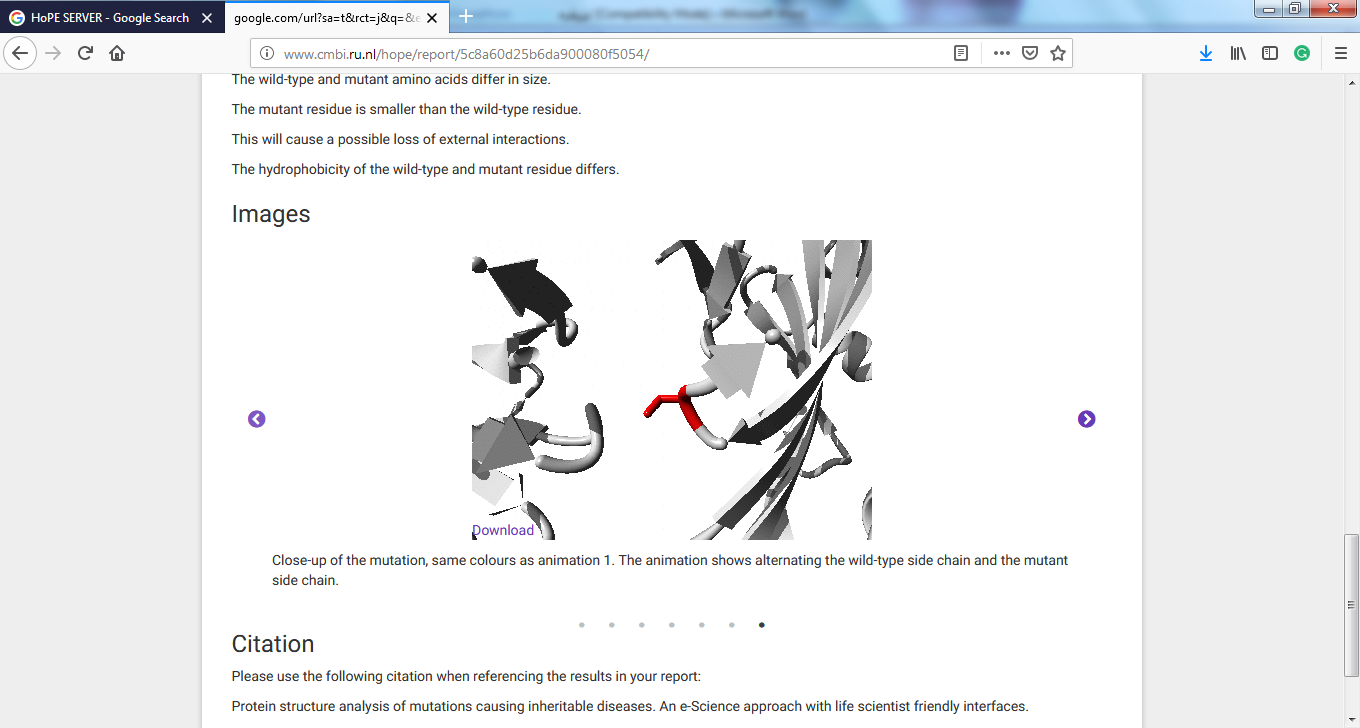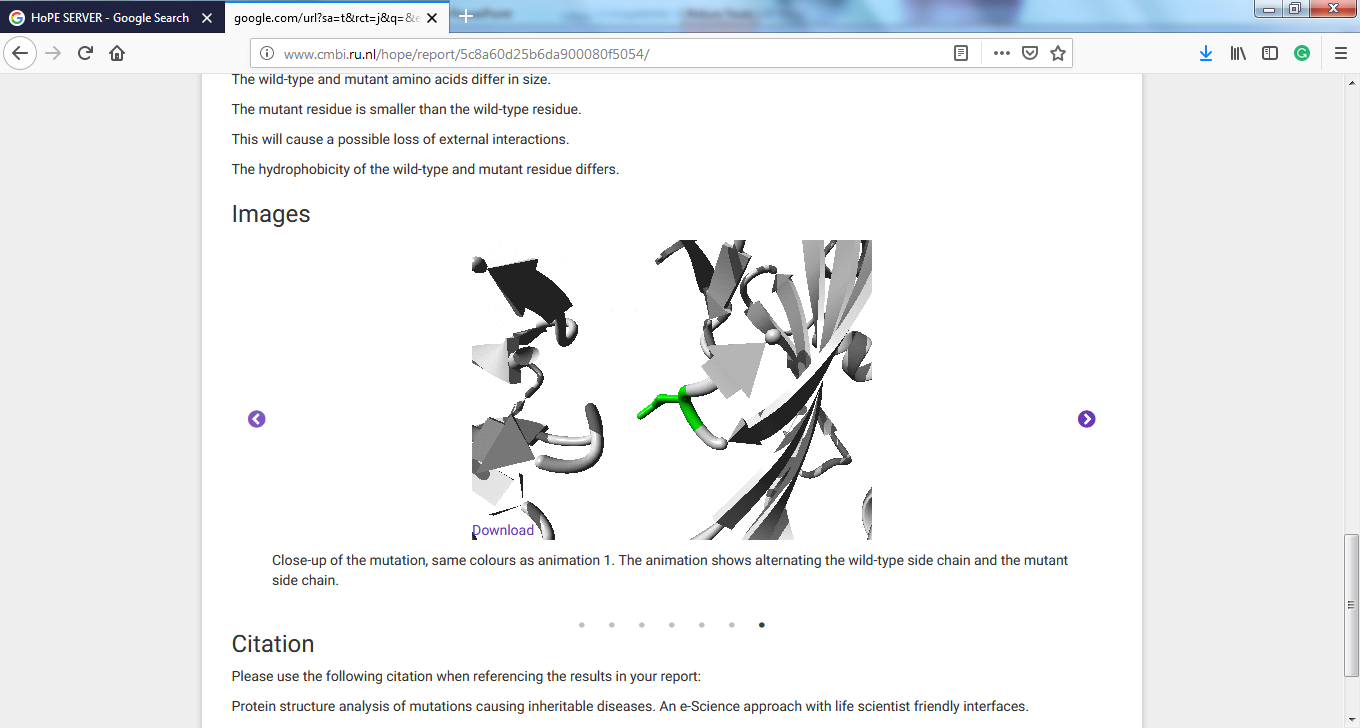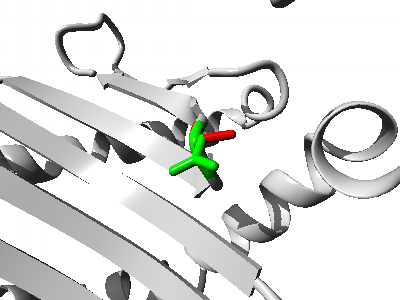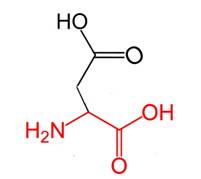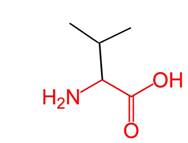 | 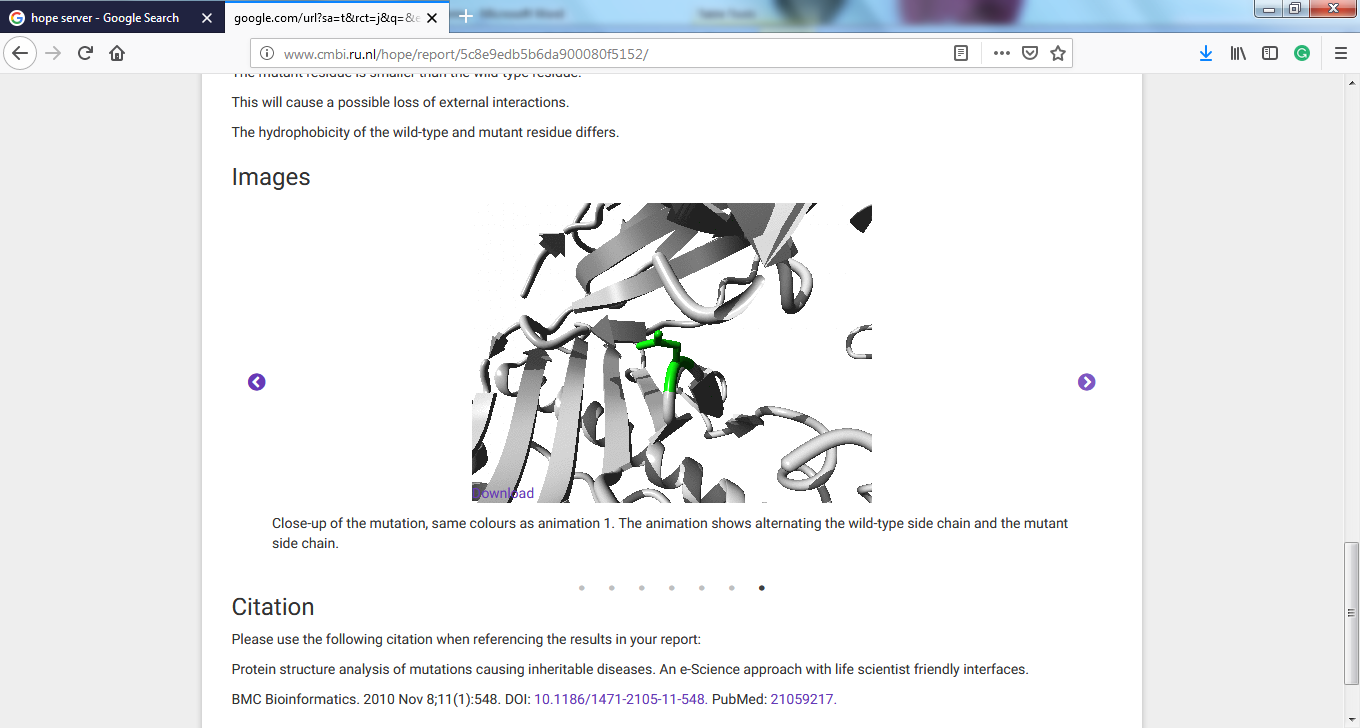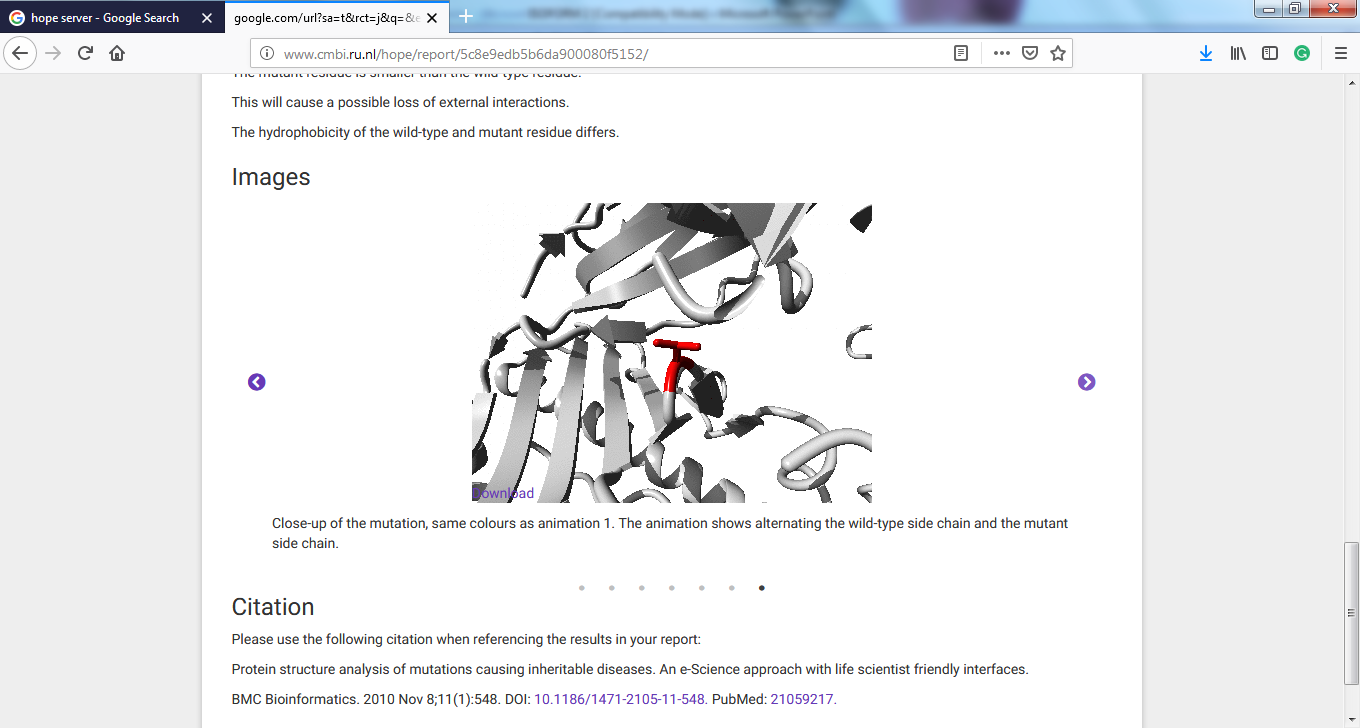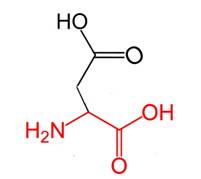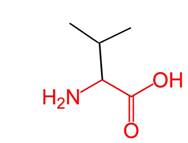 | 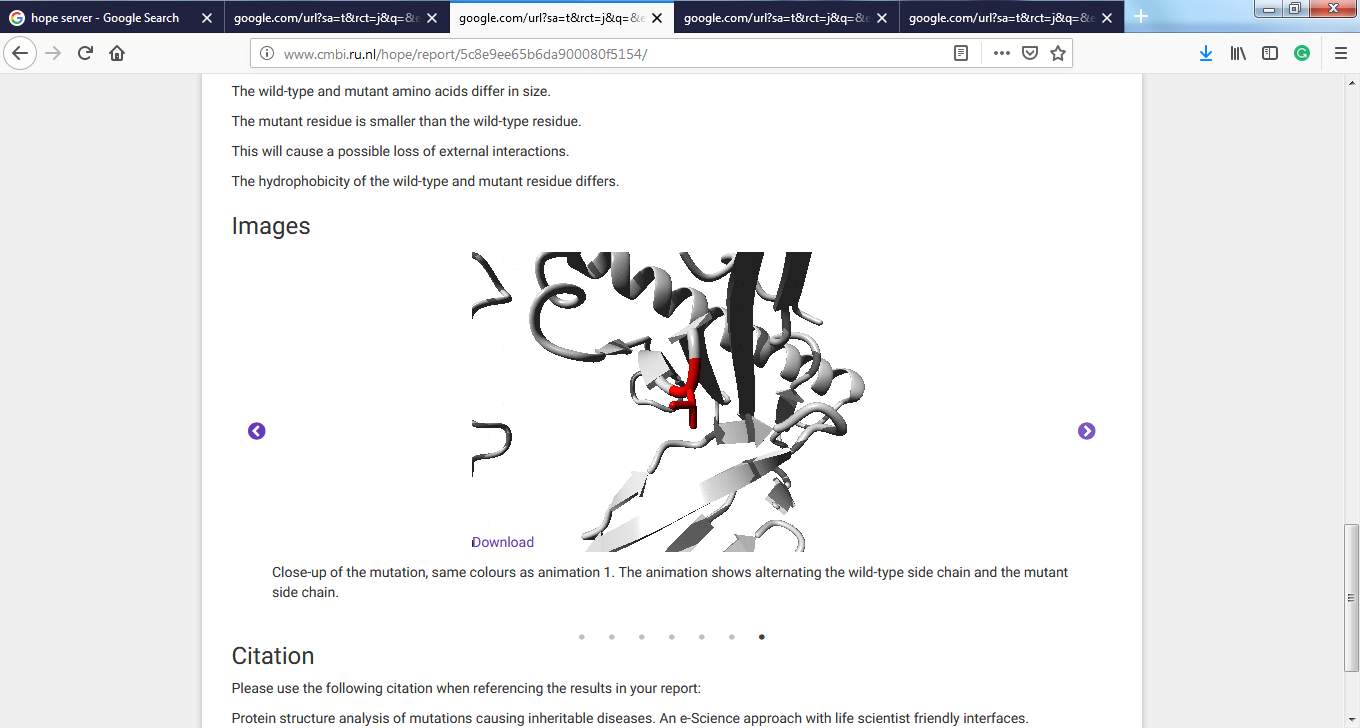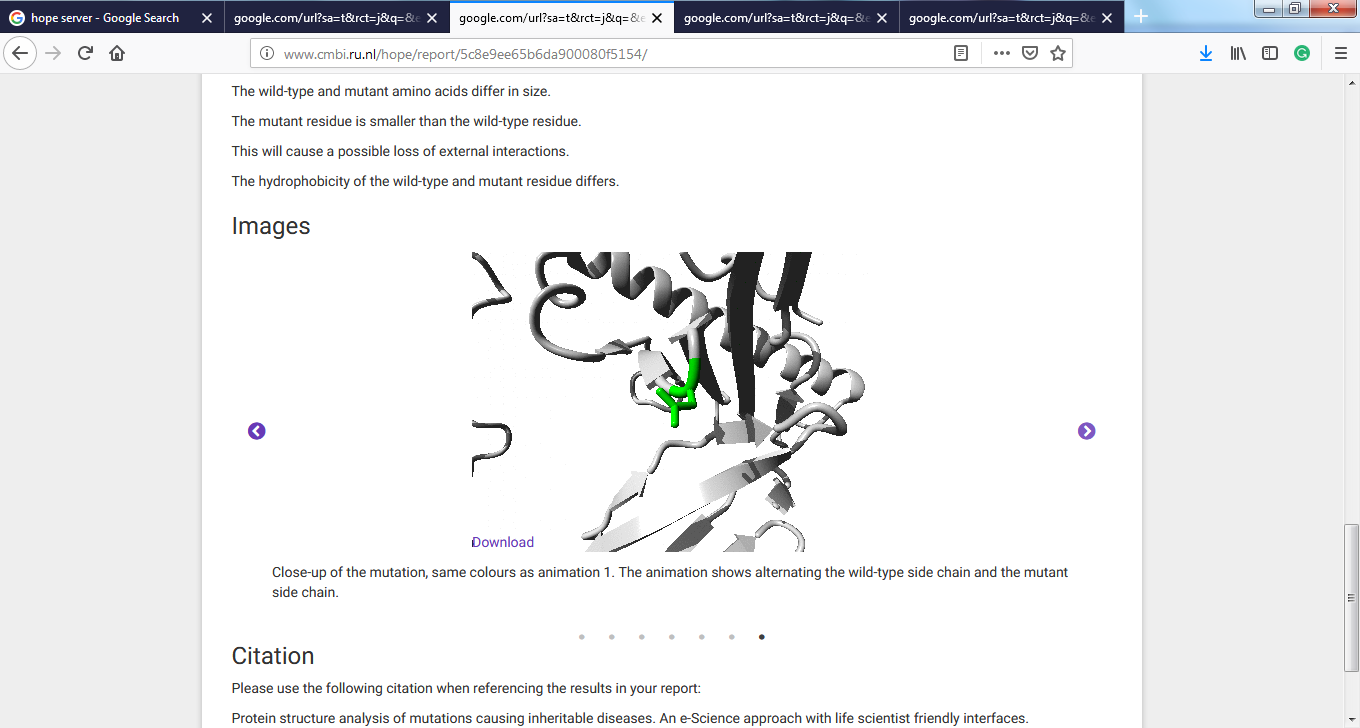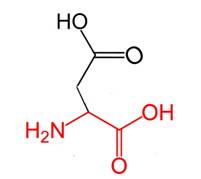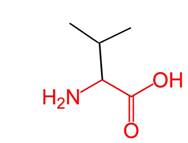 | 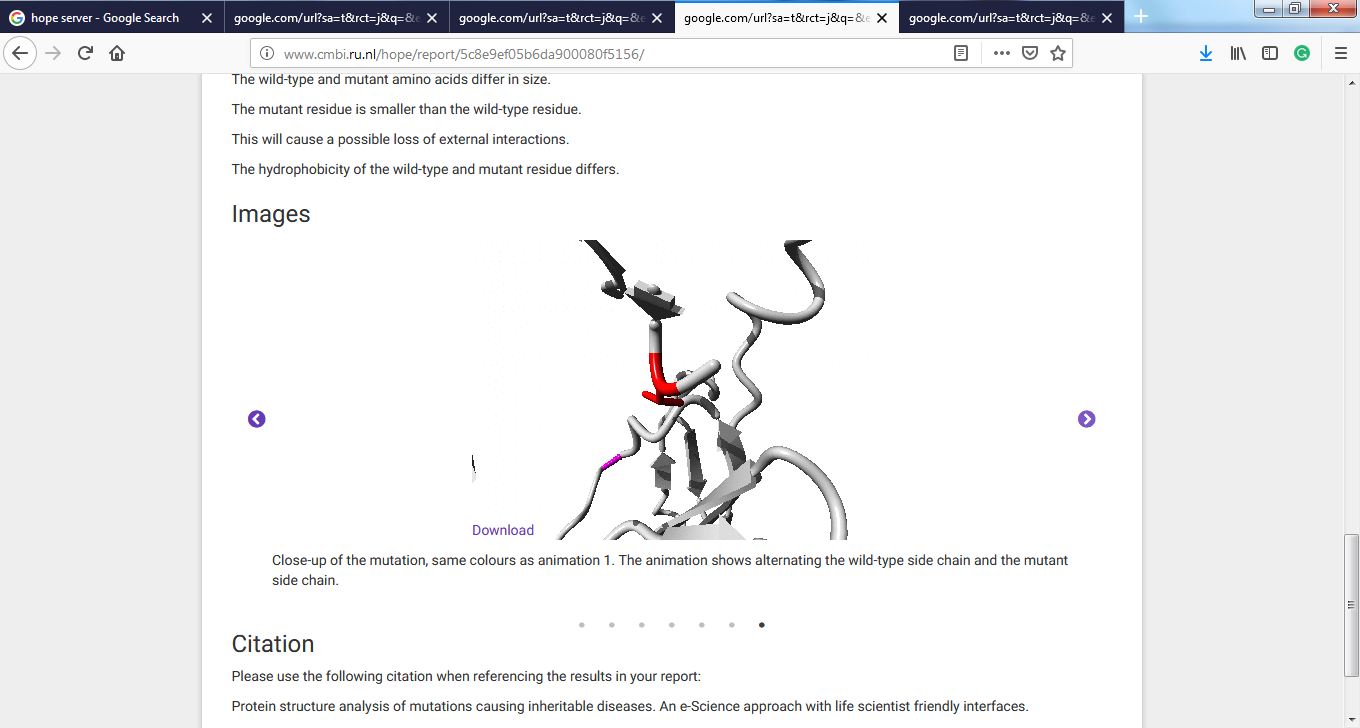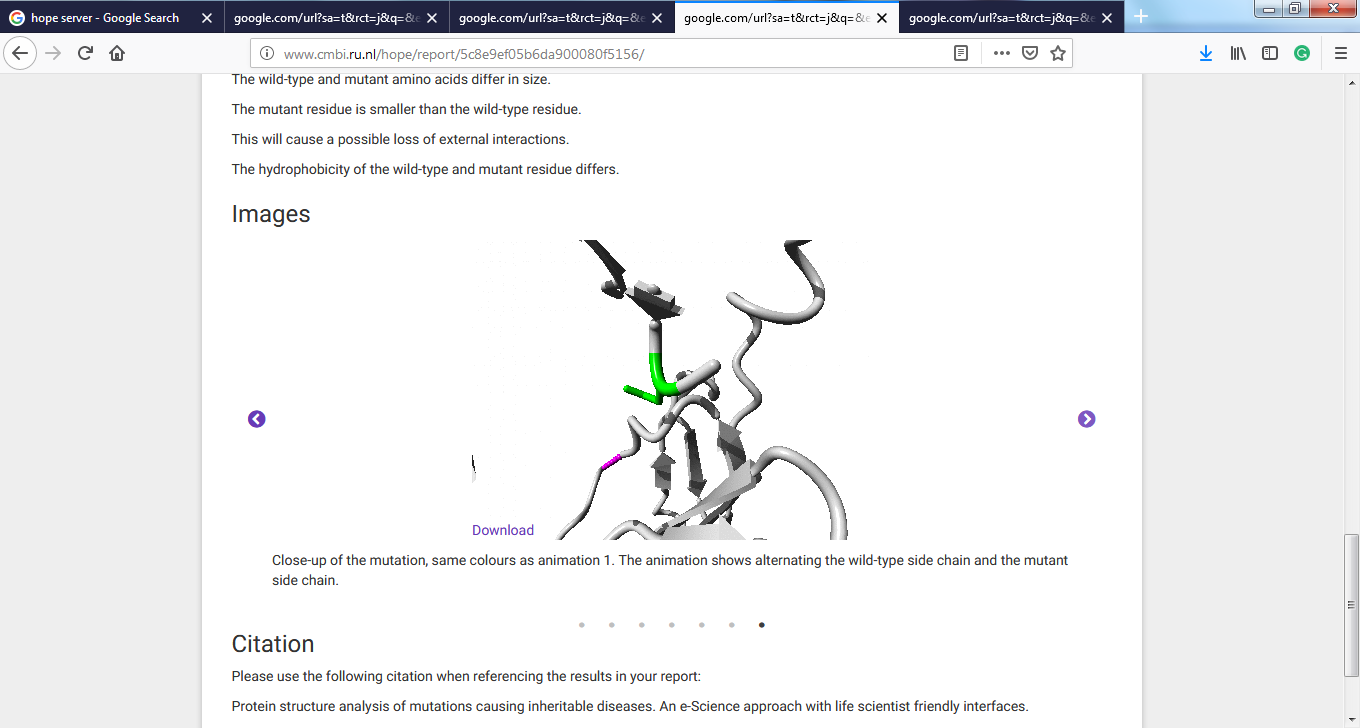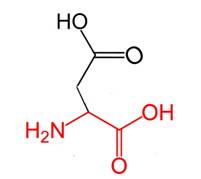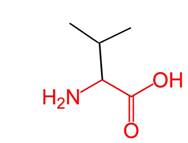 |
|  | **Amino acid properties** | The wild-type residue charge was negative; the mutant residue charge is neutral.  The charge of the wild-type residue is lost by this mutation. This can cause loss of interactions with other molecules.  The mutant residue is smaller than the wild-type residue, which will cause a possible loss of external interactions.  The mutant residue is more hydrophobic than the wild-type residue. | | | |
|  | **Structure** | The mutation is located within a stretch of residues annotated in UniProt as a special region: Alpha-1. The diversities in residue characteristics can distort this region and distort its function. | In the 3D-structure can be seen that the wild-type residue is located in its preferred secondary structure, a turn. The mutant residue prefers to be in another secondary structure; therefore the local conformation will be slightly destabilized. | The mutation is located within a stretch of residues annotated in UniProt as a special region: Alpha-1. The diversities in residue characteristics can distort this region and distort its function. | The mutation is located within a stretch of residues annotated in UniProt as a special region: Alpha-1. The diversities in residue characteristics can distort this region and distort its function. |
| **Q96P** | **Image** | 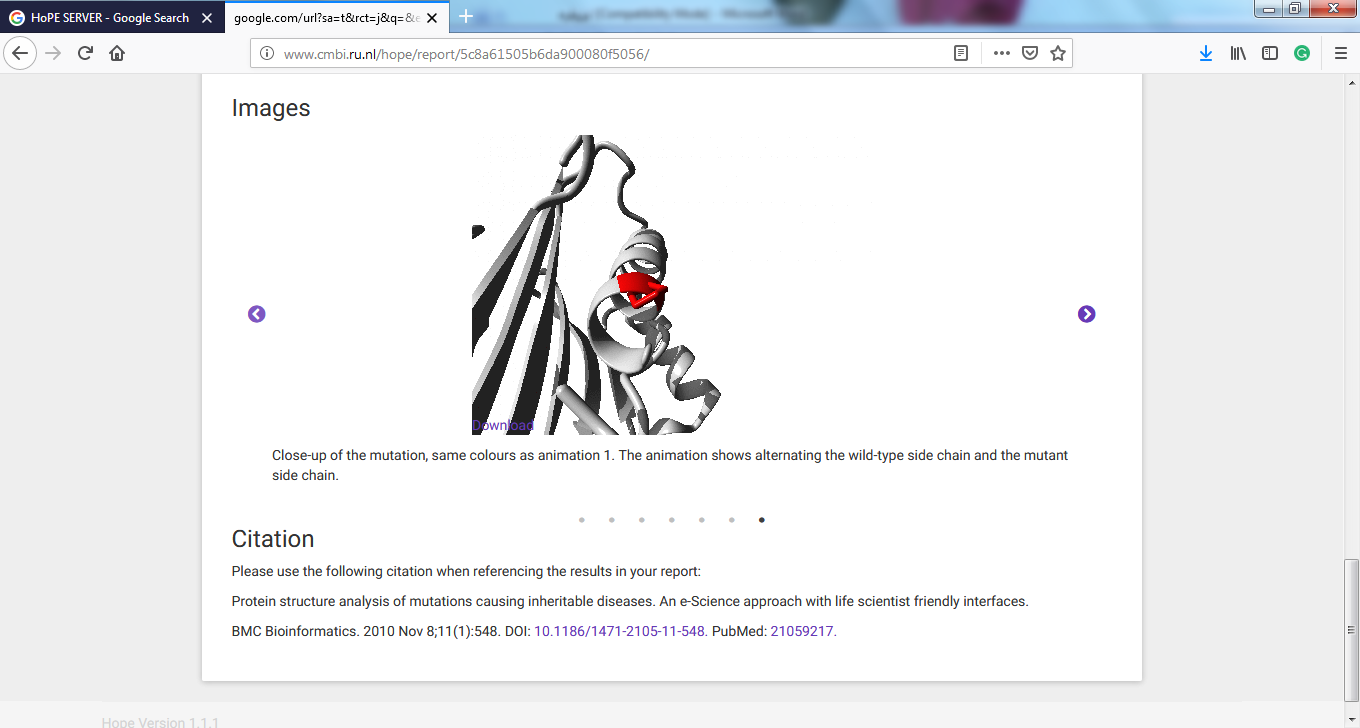 |  |  |  |
|  | **Amino acid properties** | The mutant residue is smaller than the wild-type residue, which will cause a possible loss of external interactions.  The mutant residue is more hydrophobic than the wild-type residue. | | | |
|  | **Structure** | The mutation is located within a stretch of residues annotated in UniProt as a special region: Alpha-1. The diversities in residue characteristics can distort this region and distort its function.  In the case of the mutation, the helix will be disturbed and this can have severe effects on the structure of the protein. | In the case of the mutation, the helix will be disturbed and this can have severe effects on the structure of the protein. | The mutation is located within a stretch of residues annotated in UniProt as a special region: Alpha-1. The diversities in residue characteristics can distort this region and distort its function.  In the case of the mutation, the helix will be disturbed and this can have severe effects on the structure of the protein. | The mutation is located within a stretch of residues annotated in UniProt as a special region: Alpha-1. The diversities in residue characteristics can distort this region and distort its function.  In the case of the mutation, the helix will be disturbed and this can have severe effects on the structure of the protein. |
| **L102P** | **Image** |  |  |  |  |
|  | **Amino acid properties** | The mutant residue is smaller than the wild-type residue, which will cause an empty space in the core of the protein. | | | |
|  | **Structure** | The mutation is located within a stretch of residues annotated in UniProt as a special region: Alpha-1. The diversities in residue characteristics can distort this region and distort its function.  In the case of the mutation, the helix will be disturbed and this can have severe effects on the structure of the protein. | In the case of the mutation, the helix will be disturbed and this can have severe effects on the structure of the protein. | The mutation is located within a stretch of residues annotated in UniProt as a special region: Alpha-1. The diversities in residue characteristics can distort this region and distort its function.  In the case of the mutation, the helix will be disturbed and this can have severe effects on the structure of the protein. | The mutation is located within a stretch of residues annotated in UniProt as a special region: Alpha-1. The diversities in residue characteristics can distort this region and distort its function.  In the case of the mutation, the helix will be disturbed and this can have severe effects on the structure of the protein. |
| **L105Q** | **Image** |  |  |  |  |
|  | **Amino acid properties** | The mutant residue is bigger than the wild-type residue.  The wild-type residue was buried in the core of the protein. The mutant residue is bigger and probably will not fit.  The wild-type residue is more hydrophobic than the mutant residue.  The hydrophobic interactions in the core of the protein will lose due to the mutation. | | | |
|  | **Structure** | The mutation is located within a stretch of residues annotated in UniProt as a special region: Alpha-1. The diversities in residue characteristics can distort this region and distort its function. | The mutation converts the wild-type residue in a residue that does not prefer α-helices as a secondary structure. | The mutation is located within a stretch of residues annotated in UniProt as a special region: Alpha-1. The diversities in residue characteristics can distort this region and distort its function. | The mutation is located within a stretch of residues annotated in UniProt as a special region: Alpha-1. The diversities in residue characteristics can distort this region and distort its function. |
| **L105P** | **Image** |  |  |  |  |
|  | **Amino acid properties** | The mutant residue is smaller than the wild-type residue, which will cause an empty space in the core of the protein. | | | |
|  | **Structure** | The mutation is located within a stretch of residues annotated in UniProt as a special region: Alpha-1. The diversities in residue characteristics can distort this region and distort its function.  In the case of the mutation, the helix will be disturbed and this can have severe effects on the structure of the protein. | In the case of the mutation, the helix will be disturbed and this can have severe effects on the structure of the protein. | The mutation is located within a stretch of residues annotated in UniProt as a special region: Alpha-1. The diversities in residue characteristics can distort this region and distort its function.  In the case of the mutation, the helix will be disturbed and this can have severe effects on the structure of the protein. | The mutation is located within a stretch of residues annotated in UniProt as a special region: Alpha-1. The diversities in residue characteristics can distort this region and distort its function.  In the case of the mutation, the helix will be disturbed and this can have severe effects on the structure of the protein. |

**Supplementary Table 16.** Data on the effects of each the common most deleterious predicted SNPs on structures of native soluble isoforms and the difference in physicochemical properties of amino acids of wild type and mutated residue as predicted by HOPE. Each close-up Image represents the 3D structures of the soluble isoform (gray color) with its wild type residue (green color) and mutant residue (red color).

| **Prediction of protein structure (soluble isoforms) in most deleterious nsSNPs by Project HOPE** | | |  |  |
| --- | --- | --- | --- | --- |
| **Isoform 5** | **Isoform 6** | **Isoform 7** |  |  |
| **M29K** | **Image** | The 3D structures for HLA-G5 were not predicted by HOPE due to unknown reason. |  |  |
|  | **Amino acid properties** | The mutant residue is bigger than the wild-type residue.  The wild-type amino acid has a neutral charge while the mutant amino acid has a positive charge.  The mutation introduces a charge; this can cause repulsion of ligands or other residues with the same charge.  The wild-type residue is more hydrophobic than the mutant residue.  Hydrophobic interactions will be lost either in the core of the protein or on the surface. | The mutant residue is bigger than the wild-type residue.  The wild-type residue was buried in the core of the protein. The mutant residue is bigger and probably will not fit.  The wild-type amino acid has a neutral charge while the mutant amino acid has a positive charge.  The mutant residue introduces a charge in a buried residue which can lead to protein folding problems.  The wild-type residue is more hydrophobic than the mutant residue.  The hydrophobic interactions in the core of the protein will lose due to the mutation. | |
|  | **Structure** | The mutation is located within a stretch of residues annotated in UniProt as a special region: Alpha-1. The diversities in residue characteristics can distort this region and distort its function. | | |
| **F32C** | **Image** | The 3D structures for HLA-G5 were not predicted by HOPE due to unknown reason. |  |  |
|  | **Amino acid properties** | The mutant residue is smaller than the wild-type residue, which will cause a possible loss of external interactions. | | |
|  | **Structure** | The mutation is located within a stretch of residues annotated in UniProt as a special region: Alpha-1. The diversities in residue characteristics can distort this region and distort its function. | | |
| **Y51C** | **Image** | The 3D structures for HLA-G5 were not predicted by HOPE due to unknown reason. |  |  |
|  | **Amino acid  properties** | The mutant residue is smaller than the wild-type residue.  The mutant residue is more hydrophobic than the wild-type residue.  The mutation introduces a more hydrophobic residue at this position which can result in loss of hydrogen bonds and/or disturb appropriate folding. | The mutant residue is smaller than the wild-type residue, which will cause a possible loss of external interactions.  The mutant residue is more hydrophobic than the wild-type residue. | The mutant residue is smaller than the wild-type residue, which will cause a possible loss of external interactions.  The mutant residue is more hydrophobic than the wild-type residue. |
|  | **Structure** | The mutation is located within a stretch of residues annotated in UniProt as a special region: Alpha-1. The diversities in residue characteristics can distort this region and distort its function. | | |
| **D53N** | **Image** | The 3D structures for HLA-G5 were not predicted by HOPE due to unknown reason. |  |  |
|  | **Amino acid properties** | The wild-type residue charge was negative; the mutant residue charge is neutral.  The charge of the wild-type residue will be lost; this can cause loss of interactions with other molecules or residues. | | |
|  | **Structure** | The mutation is located within a stretch of residues annotated in UniProt as a special region: Alpha-1. The diversities in residue characteristics can distort this region and distort its function. | | |
| **D53Y** | **Image** | The 3D structures for HLA-G5 were not predicted by HOPE due to unknown reason. |  |  |
|  | **Amino acid properties** | The wild-type residue charge was negative; the mutant residue charge is neutral.  The charge of the wild-type residue will be lost; this can cause loss of interactions with other molecules or residues.  The mutant residue is bigger; this might lead to bumps.  The mutant residue is more hydrophobic than the wild-type residue which can result in loss of hydrogen bonds and/or disturb appropriate folding.. | The wild-type residue charge was negative; the mutant residue charge is neutral.  The charge of the wild-type residue is lost by this mutation. This can cause loss of interactions with other molecules.  The mutant residue is bigger than the wild-type residue.  The amino acid is located on the surface of the protein; mutation of this amino acid can distort interactions with other molecules or other sections of the protein.  The mutant residue is more hydrophobic than the wild-type residue. | |
|  | **Structure** | The mutation is located within a stretch of residues annotated in UniProt as a special region: Alpha-1. The diversities in residue characteristics can distort this region and distort its function. | | |
| **D54V** | **Image** | The 3D structures for HLA-G5 were not predicted by HOPE due to unknown reason. |  |  |
|  | **Amino acid properties** | The wild-type residue charge was negative; the mutant residue charge is neutral.  The charge of the wild-type residue will be lost; this can cause loss of interactions with other molecules or residues.  The mutant residue is smaller; this might lead to loss of interactions.  The mutant residue is more hydrophobic than the wild-type residue.  The mutation introduces a more hydrophobic residue at this position which can result in loss of hydrogen bonds and/or disturb appropriate folding. | The wild-type residue charge was negative; the mutant residue charge is neutral.  The charge of the wild-type residue is lost by this mutation, which can cause loss of interactions with other molecules.  The mutant residue is smaller; this might lead to loss of interactions.  The mutant residue is more hydrophobic than the wild-type residue. | |
|  | **Structure** | The mutation is located within a stretch of residues annotated in UniProt as a special region: Alpha-1. The diversities in residue characteristics can distort this region and distort its function. | | |
| **Q96P** | **Image** | The 3D structures for HLA-G5 were not predicted by HOPE due to unknown reason. |  |  |
|  | **Amino acid properties** | The mutant residue is smaller; this might lead to loss of interactions.  The mutant residue is more hydrophobic than the wild-type residue.  The mutation introduces a more hydrophobic residue at this position. This can result in loss of hydrogen bonds and/or disturb correct folding. | The mutant residue is smaller; this might lead to loss of interactions.  The mutant residue is more hydrophobic than the wild-type residue. | |
|  | **Structure** | The mutation is located within a stretch of residues annotated in UniProt as a special region: Alpha-1. The diversities in residue characteristics can distort this region and distort its function. In the case of the mutation, the helix will be disturbed and this can have severe effects on the structure of the protein. | | |
| **L102P** | **Image** | The 3D structures for HLA-G5 were not predicted by HOPE due to unknown reason. |  |  |
|  | **Amino acid properties** | The mutant residue is smaller; this might lead to loss of interactions. | The mutant residue is smaller than the wild-type residue, which will cause an empty space in the core of the protein. | The mutant residue is smaller than the wild-type residue.  The mutation will cause an empty space in the core of the protein. |
|  | **Structure** | The mutation is located within a stretch of residues annotated in UniProt as a special region: Alpha-1. The diversities in residue characteristics can distort this region and distort its function. In the case of the mutation, the helix will be disturbed and this can have severe effects on the structure of the protein. | | |
| **L105P** | **Image** | The 3D structures for HLA-G5 were not predicted by HOPE due to unknown reason. |  |  |
|  | **Amino acid properties** | The mutant residue is smaller; this might lead to loss of interactions. | The mutant residue is smaller than the wild-type residue, which will cause an empty space in the core of the protein. | |
|  | **Structure** | The mutation is located within a stretch of residues annotated in UniProt as a special region: Alpha-1. The diversities in residue characteristics can distort this region and distort its function.  In the case of the mutation, the helix will be disturbed and this can have severe effects on the structure of the protein. | | |

**Supplementary Table 17.** Top 10 threading templates used by I-TASSER to construct the great quality models for HLA-G isoforms. “Ident1” is the percentage sequence identity of the templates in the threading aligned part with the query sequence. “Ident2” is the percentage sequence identity of the complete template chains with query sequence. “Cov” shows the coverage of the threading alignment. “Norm. 𝑍-score” is the normalized 𝑍-score of the threading alignments. Normalized 𝑍-score > 1 means a well alignment and conversely (https://zhanglab.ccmb.med.umich.edu/I-TASSER/example/).

| **Isoform1** | | | | | | **Isoform2** | | | | | | | | | | | | | | | | | | | |
| --- | --- | --- | --- | --- | --- | --- | --- | --- | --- | --- | --- | --- | --- | --- | --- | --- | --- | --- | --- | --- | --- | --- | --- | --- | --- |
| **Rank** | **PDB hit** | **Iden1** | **Iden2** | **Cov.** | **Norm. Z-score** | **Rank** | | | | **PDB hit** | | | **Iden1** | | | **Iden2** | | | | **Cov.** | **Norm. Z-score** | | | | |
| 1 | [2bckA](http://www.rcsb.org/pdb/explore/explore.do?structureId=2bck) | 0.904 | 0.55 | 0.808 | 0.911 | 1 | [2qriA](http://www.rcsb.org/pdb/explore/explore.do?structureId=2qri) | | | | 0.43 | | | 0.58 | | | | 0.82 | | | | 1.77 | | | |
| 2 | [1zvsA](http://www.rcsb.org/pdb/explore/explore.do?structureId=1zvs) | 0.866 | 1.18 | 0.813 | 0.885 | 2 | [1lnuA](http://www.rcsb.org/pdb/explore/explore.do?structureId=1lnu) | | | | 0.23 | | | 0.24 | | | | 0.83 | | | | 3.06 | | | |
| 3 | [3bw9A](http://www.rcsb.org/pdb/explore/explore.do?structureId=3bw9) | 0.864 | 0.90 | 0.822 | 0.879 | 3 | [2qrtA](http://www.rcsb.org/pdb/explore/explore.do?structureId=2qrt) | | | | 0.44 | | | 0.57 | | | | 0.81 | | | | 3.59 | | | |
| 4 | [3bzfC](http://www.rcsb.org/pdb/explore/explore.do?structureId=3bzf) | 0.862 | 0.97 | 0.783 | 0.879 | 4 | [1mhc](http://www.rcsb.org/pdb/explore/explore.do?structureId=1mhc) | | | | 0.46 | | | 0.59 | | | | 0.82 | | | | 1.21 | | | |
| 5 | [1i4fA](http://www.rcsb.org/pdb/explore/explore.do?structureId=1i4f) | 0.858 | 0.97 | 0.822 | 0.876 | 5 | [2bck](http://www.rcsb.org/pdb/explore/explore.do?structureId=2bck) | | | | 0.42 | | | 0.71 | | | | 0.97 | | | | 0.97 | | | |
| 6 | [4nt6A](http://www.rcsb.org/pdb/explore/explore.do?structureId=4nt6) | 0.857 | 0.88 | 0.847 | 0.873 | 6 | [3usaD](http://www.rcsb.org/pdb/explore/explore.do?structureId=3usa) | | | | 0.20 | | | 0.21 | | | | 0.82 | | | | 2.50 | | | |
| 7 | [3c8kA](http://www.rcsb.org/pdb/explore/explore.do?structureId=3c8k) | 0.852 | 1.02 | 0.693 | 0.873 | 7 | [2bck](http://www.rcsb.org/pdb/explore/explore.do?structureId=2bck) | | | | 0.81 | | | 0.71 | | | | 0.85 | | | | 1.71 | | | |
| 8 | [2qriA](http://www.rcsb.org/pdb/explore/explore.do?structureId=2qri) | 0.848 | 1.32 | 0.692 | 0.879 | 8 | [4nt6A](http://www.rcsb.org/pdb/explore/explore.do?structureId=4nt6) | | | | 0.54 | | | 0.70 | | | | 0.82 | | | | 3.80 | | | |
| 9 | [3buyA](http://www.rcsb.org/pdb/explore/explore.do?structureId=3buy) | 0.845 | 1.32 | 0.698 | 0.876 | 9 | [3usaD](http://www.rcsb.org/pdb/explore/explore.do?structureId=3usa) | | | | 0.19 | | | 0.21 | | | | 0.84 | | | | 1.81 | | | |
| 10 | [1mhcA](http://www.rcsb.org/pdb/explore/explore.do?structureId=1mhc) | 0.842 | 1.79 | 0.670 | 0.879 | 10 | [1hdmB](http://www.rcsb.org/pdb/explore/explore.do?structureId=1hdm) | | | | 0.17 | | | 0.20 | | | | 0.82 | | | | 1.87 | | | |
| **Isoform3** | | | | | | **Isoform4** | | | | | | | | | | | | | | | | | | |  |
| 1 | [1mhcA](http://www.rcsb.org/pdb/explore/explore.do?structureId=1mhc) | 0.49 | 0.52 | 0.99 | 2.82 | 1 | | [2qriA](http://www.rcsb.org/pdb/explore/explore.do?structureId=2qri) | | | 0.57 | | | 0.59 | | | | | 1.00 | | | | 3.15 | | |
| 2 | [3am8A](http://www.rcsb.org/pdb/explore/explore.do?structureId=3am8) | 0.53 | 0.58 | 0.99 | 3.21 | 2 | | [3qq3A](http://www.rcsb.org/pdb/explore/explore.do?structureId=3qq3) | | | 0.71 | | | 0.58 | | | | | 0.82 | | | | 4.00 | | |
| 3 | [3bo8A](http://www.rcsb.org/pdb/explore/explore.do?structureId=3bo8) | 0.61 | 0.66 | 1.00 | 4.93 | 3 | | [3bo8A](http://www.rcsb.org/pdb/explore/explore.do?structureId=3bo8) | | | 0.68 | | | 0.68 | | | | | 1.00 | | | | 4.85 | | |
| 4 | [3p73](http://www.rcsb.org/pdb/explore/explore.do?structureId=3p73) | 0.29 | 0.34 | 0.98 | 1.71 | 4 | | [2bck](http://www.rcsb.org/pdb/explore/explore.do?structureId=2bck) | | | 0.69 | | | 0.70 | | | | | 0.99 | | | | 1.72 | | |
| 5 | [2bck](http://www.rcsb.org/pdb/explore/explore.do?structureId=2bck) | 0.58 | 0.64 | 1.00 | 1.30 | 5 | | [2bck](http://www.rcsb.org/pdb/explore/explore.do?structureId=2bck) | | | 0.68 | | | 0.70 | | | | | 1.00 | | | | 1.29 | | |
| 6 | [1zs8A](http://www.rcsb.org/pdb/explore/explore.do?structureId=1zs8) | 0.30 | 0.35 | 0.96 | 3.34 | 6 | | [1mhcA](http://www.rcsb.org/pdb/explore/explore.do?structureId=1mhc) | | | 0.54 | | | 0.56 | | | | | 1.00 | | | | 3.61 | | |
| 7 | [2bck](http://www.rcsb.org/pdb/explore/explore.do?structureId=2bck) | 0.62 | 0.64 | 0.98 | 1.97 | 7 | | [2bck](http://www.rcsb.org/pdb/explore/explore.do?structureId=2bck) | | | 0.67 | | | 0.70 | | | | | 1.00 | | | | 1.99 | | |
| 8 | [4prhA](http://www.rcsb.org/pdb/explore/explore.do?structureId=4prh) | 0.55 | 0.58 | 0.98 | 2.75 | 8 | | [4prhA](http://www.rcsb.org/pdb/explore/explore.do?structureId=4prh) | | | 0.70 | | | 0.66 | | | | | 0.93 | | | | 4.28 | | |
| 9 | [1zs8A](http://www.rcsb.org/pdb/explore/explore.do?structureId=1zs8) | 0.29 | 0.35 | 0.97 | 2.64 | 9 | | [5cnzA](http://www.rcsb.org/pdb/explore/explore.do?structureId=5cnz) | | | 0.45 | | | 0.36 | | | | | 0.79 | | | | 3.40 | | |
| 10 | [1i4fA](http://www.rcsb.org/pdb/explore/explore.do?structureId=1i4f) | 0.58 | 0.64 | 1.00 | 1.69 | 10 | | [1i4fA](http://www.rcsb.org/pdb/explore/explore.do?structureId=1i4f) | | | 0.67 | | | 0.69 | | | | | 1.00 | | | | 3.37 | | |
| **Isoform5** | | | | | | **Isoform6** | | | | | | | | | | | | | | | | | | | |
| 1 | [2bckA](http://www.rcsb.org/pdb/explore/explore.do?structureId=2bck) | 0.82 | 0.79 | 0.97 | 3.39 | 1 | | | [4iiqC](http://www.rcsb.org/pdb/explore/explore.do?structureId=4iiq) | | | 0.23 | | | 0.35 | | 0.87 | | | | | | | 1.88 | |
| 2 | [1zvsA](http://www.rcsb.org/pdb/explore/explore.do?structureId=1zvs) | 0.81 | 0.76 | 0.94 | 4.79 | 2 | | | [1lnuA](http://www.rcsb.org/pdb/explore/explore.do?structureId=1lnu) | | | 0.23 | | | 0.26 | | 0.90 | | | | | | | 3.12 | |
| 3 | [2bckA](http://www.rcsb.org/pdb/explore/explore.do?structureId=2bck) | 0.81 | 0.79 | 0.97 | 5.31 | 3 | | | [2qrtA](http://www.rcsb.org/pdb/explore/explore.do?structureId=2qrt) | | | 0.44 | | | 0.63 | | 0.89 | | | | | | | 3.44 | |
| 4 | [2bck](http://www.rcsb.org/pdb/explore/explore.do?structureId=2bck) | 0.81 | 0.79 | 0.97 | 1.57 | 4 | | | [2bck](http://www.rcsb.org/pdb/explore/explore.do?structureId=2bck) | | | 0.80 | | | 0.78 | | 0.96 | | | | | | | 1.18 | |
| 5 | [2bck](http://www.rcsb.org/pdb/explore/explore.do?structureId=2bck) | 0.81 | 0.79 | 0.97 | 1.28 | 5 | | | [2bck](http://www.rcsb.org/pdb/explore/explore.do?structureId=2bck) | | | 0.75 | | | 0.78 | | 0.96 | | | | | | | 1.07 | |
| 6 | [2bckA](http://www.rcsb.org/pdb/explore/explore.do?structureId=2bck) | 0.82 | 0.79 | 0.97 | 4.14 | 6 | | | [3usaD](http://www.rcsb.org/pdb/explore/explore.do?structureId=3usa) | | | 0.20 | | | 0.23 | | 0.90 | | | | | | | 2.54 | |
| 7 | [2bck](http://www.rcsb.org/pdb/explore/explore.do?structureId=2bck) | 0.82 | 0.79 | 0.96 | 1.88 | 7 | | | [2bck](http://www.rcsb.org/pdb/explore/explore.do?structureId=2bck) | | | 0.77 | | | 0.78 | | 0.95 | | | | | | | 1.50 | |
| 8 | [4nt6A](http://www.rcsb.org/pdb/explore/explore.do?structureId=4nt6) | 0.85 | 0.79 | 0.93 | 6.94 | 8 | | | [4nt6A](http://www.rcsb.org/pdb/explore/explore.do?structureId=4nt6) | | | 0.85 | | | 0.77 | | 0.90 | | | | | | | 4.08 | |
| 9 | [2bckA](http://www.rcsb.org/pdb/explore/explore.do?structureId=2bck) | 0.81 | 0.79 | 0.97 | 3.25 | 9 | | | [3usaD](http://www.rcsb.org/pdb/explore/explore.do?structureId=3usa) | | | 0.19 | | | 0.23 | | 0.91 | | | | | | | 1.82 | |
| 10 | [1mhcA](http://www.rcsb.org/pdb/explore/explore.do?structureId=1mhc) | 0.67 | 0.63 | 0.94 | 3.36 | 10 | | | [1hdmB](http://www.rcsb.org/pdb/explore/explore.do?structureId=1hdm) | | | 0.17 | | | 0.22 | | 0.90 | | | | | | | 2.03 | |
| **Isoform7** | | | | | |  |  |  |  |  |  |  |  |  |  |  |  |  |  |  |  |  |  |  |  |
| 1 | [2qriA](http://www.rcsb.org/pdb/explore/explore.do?structureId=2qri) | 0.65 | 0.64 | 1.00 | 3.38 |  |  |  |  |  |  |  |  |  |  |  |  |  |  |  |  |  |  |  |  |
| 2 | [3am8A](http://www.rcsb.org/pdb/explore/explore.do?structureId=3am8) | 0.72 | 0.72 | 1.00 | 2.76 |  |  |  |  |  |  |  |  |  |  |  |  |  |  |  |  |  |  |  |  |
| 3 | [1nezA](http://www.rcsb.org/pdb/explore/explore.do?structureId=1nez) | 0.58 | 0.46 | 0.78 | 4.63 |  |  |  |  |  |  |  |  |  |  |  |  |  |  |  |  |  |  |  |  |
| 4 | [2bck](http://www.rcsb.org/pdb/explore/explore.do?structureId=2bck) | 0.78 | 0.78 | 1.00 | 1.74 |  |  |  |  |  |  |  |  |  |  |  |  |  |  |  |  |  |  |  |  |
| 5 | [2bck](http://www.rcsb.org/pdb/explore/explore.do?structureId=2bck) | 0.77 | 0.78 | 1.00 | 1.30 |  |  |  |  |  |  |  |  |  |  |  |  |  |  |  |  |  |  |  |  |
| 6 | [4gupA](http://www.rcsb.org/pdb/explore/explore.do?structureId=4gup) | 0.41 | 0.40 | 0.96 | 2.83 |  |  |  |  |  |  |  |  |  |  |  |  |  |  |  |  |  |  |  |  |
| 7 | [2bck](http://www.rcsb.org/pdb/explore/explore.do?structureId=2bck) | 0.78 | 0.78 | 0.99 | 1.96 |  |  |  |  |  |  |  |  |  |  |  |  |  |  |  |  |  |  |  |  |
| 8 | [4prhA](http://www.rcsb.org/pdb/explore/explore.do?structureId=4prh) | 0.74 | 0.74 | 0.99 | 2.19 |  |  |  |  |  |  |  |  |  |  |  |  |  |  |  |  |  |  |  |  |
| 9 | [1c16A](http://www.rcsb.org/pdb/explore/explore.do?structureId=1c16) | 0.54 | 0.52 | 0.97 | 2.15 |  |  |  |  |  |  |  |  |  |  |  |  |  |  |  |  |  |  |  |  |
| 10 | [1i4fA](http://www.rcsb.org/pdb/explore/explore.do?structureId=1i4f) | 0.79 | 0.79 | 1.00 | 1.33 |  |  |  |  |  |  |  |  |  |  |  |  |  |  |  |  |  |  |  |  |

**Supplementary Table 18.** Graphical representations of amino acid changes due to the most deleterious SNPs in isoform 5

| **Graphical representations of amino acid changes due to most deleterious SNPs in isoform 5** | | | | |  |
| --- | --- | --- | --- | --- | --- |
| **SNP ID:** **rs555347515** | **SNP ID: rs572025435** | | **SNP ID: rs540632198** | |  |
| **protein position 29 changed from Methionine (green (upper image)) to**  **Lysine (red (bottom image))** |  | **protein position 30 changed from Arginine (green (upper image)) to  Serine (red (bottom image))** |  | **protein position 32 changed from Phenylalanine (green (upper image))  to Cysteine (red (bottom image))** |  |
|  |  |  |  |  |  |
| **protein position 44 changed from Proline (green (upper image)) to  Leucine (red (bottom image))** | **SNP ID: rs1475659109** | **SNP ID: rs1390270595** | | **SNP ID: rs763201540** | |
|  |  | **protein position 51 changed from Tyrosine (green (upper image)) to  Cysteine (red (bottom image))** |  | **protein position 53 changed from Aspartic acid (green (upper image)) to  Asparagine (red (bottom image))** |  |
|  |  |  |  |  |  |
| **protein position 53 changed from Aspartic acid (green (upper image)) to  Tyrosine (red (bottom image))** | **SNP ID: rs763201540** | **SNP ID: rs1414848134** | | **SNP ID: rs138289952** | |
|  |  | **protein position 54 changed from Aspartic acid (green (upper image)) to  Valine (red (bottom image))** |  | **protein position 54 changed from Aspartic acid (green (upper image)) to  Tyrosine (red (bottom image))** |  |
|  |  |  |  |  |  |
| **protein position 96 changed from Glutamine (green (upper image)) to  Proline (red (bottom image))** | **SNP ID: rs1260086927** | **SNP ID: rs770412396** | | **SNP ID: rs1161818149** | |
|  |  | **protein position 102 changed from Leucine (green (upper image)) to  Proline (red (bottom image))** |  | **protein position 105 changed from Leucine (green (upper image)) to  Glutamine (red (bottom image))** |  |
|  |  |  |  |  |  |
| **protein position 105 changed from Leucine (green (upper image)) to  Proline (red (bottom image))** | **SNP ID: rs1161818149** | **SNP ID:** **rs776393668** | | **SNP ID: rs17851921** | |
|  |  | **protein position 113 changed from Glutamic acid (green (upper image)) to Valine (red (bottom image))** |  | **protein position 117 changed from Histidine (green (upper image)) to  Proline (red (bottom image))** |  |
|  |  |  |  |  |  |
| **protein position 117 changed from Histidine (green (upper image)) to  Leucine (red (bottom image))** | **SNP ID: rs17851921** | **SNP ID: rs565858069** | | **SNP ID: rs749006959** | |
|  |  | **protein position 130 changed from Aspartic acid (green (upper image)) to  Histidine (red (bottom image))** |  | **protein position 142 changed from Tyrosine (green (upper image)) to  Cysteine (red (bottom image))** |  |
|  |  |  |  |  |  |
| **protein position 142 changed from Tyrosine (green (upper image)) to  Histidine (red (bottom image))** | **SNP ID: rs772834879** | **SNP ID: rs1317292772** | | **SNP ID: rs1317292772** | |
|  |  | **protein position 143 changed from Aspartic acid (green (upper image)) to  Asparagine (red (bottom image))** |  | **protein position 143 changed from Aspartic acid (green (upper image)) to  Histidine (red (bottom image))** |  |
|  |  |  |  |  |  |
| **protein position 153 changed from Aspartic acid (green (upper image)) to  Glycine (red (bottom image))** | **SNP ID: rs556645753** | **SNP ID: rs867319917** | | **SNP ID: rs748013931** | |
|  |  | **Protein position 157 changed from Tryptophan (green (upper image)) to  Arginine (red (bottom image))** |  | **protein position 158 changed from Threonine (green (upper image)) to  Proline (red (bottom image))** |  |
|  |  |  |  |  |  |
| **protein position 188 changed from Cysteine (green (upper image)) to  Serine (red (bottom image))** | **SNP ID: rs780697086** | **SNP ID: rs1397132797** | | **SNP ID: rs1379742188** | |
|  |  | **protein position 196 changed from Leucine (green (upper image)) to  Proline (red (bottom image))** |  | **protein position 205 changed from Arginine (green (upper image)) to  Serine (red (bottom image))** |  |
|  |  |  |  |  |  |
| **SNP ID: rs1438362414** | | **SNP ID: rs144577485** | | **SNP ID: rs1472538844** | |
| **protein position 205 changed from Arginine (green (upper image)) to  Leucine (red (bottom image))** |  | **protein position 209 changed from Proline (green (upper image)) to  Alanine (red (bottom image))** |  | **protein position 209 changed from Proline (green (upper image)) to  Arginine (red (bottom image))** |  |
|  |  |  |  |  |  |
| **SNP ID: rs770027530** | | **SNP ID: rs770027530** | | **SNP ID: rs1200732770** | |
| **protein position 227 changed from Cysteine (green (upper image)) to  Phenylalanine (red (bottom image))** |  | **Potein position 227 changed from Cysteine (green (upper image)) to  Tyrosine (red (bottom image))** |  | **protein position 229 changed from Alanine (green (upper image)) to  Aspartic acid (red (bottom image))** |  |
|  |  |  |  |  |  |
| **SNP ID: rs142596947** | | **SNP ID: rs1430565057** | | **SNP ID: rs750238738** | |
| **protein position 234 changed from Proline (green (upper image)) to  Threonine (red (bottom image))** |  | **protein position 234 changed from Proline (green (upper image)) to  Leucine (red (bottom image))** |  | **protein position 237 changed from Isoleucine (green (upper image)) to  Phenylalanine (red (bottom image))** |  |
|  |  |  |  |  |  |
| **SNP ID: rs781774818** | | **SNP ID: rs760500349** | | **SNP ID: rs145097667** | |
| **protein position 259 changed from Proline (green (upper image)) to  Histidine (red (bottom image))** |  | **protein position 266 changed from Glutamine (green (upper image)) to  Leucine (red (bottom image))** |  | **protein position 287 changed from Histidine (green (upper image)) to  Tyrosine (red (bottom image))** |  |
|  |  |  |  |  |  |
| **SNP ID: rs765275727** | |  |  |  |  |
| **protein position 298 changed from Tryptophan (green (upper image)) to  Arginine (red (bottom image))** |  |  |  |  |  |
|  |  |  |  |  |  |

**Supplementary Table 19.** Graphical representations of amino acid changes due to the most deleterious SNPs in transmembrane isoforms

| **Graphical representations of amino acid changes due to most deleterious SNPs in transmembrane isoforms** | | | | | |  |
| --- | --- | --- | --- | --- | --- | --- |
| **Isoform 1** | **Isoform 2** | | | **Isoform 3** | **Isoform 4** |  |
| **protein position 9 changed from Methionine (green (upper image)) to Lysine (red (bottom image))** | | | | | |  |
| **SNP ID: rs555347515** |  |  | |  | |  |
|  |  |  | |  | |  |
| **SNP ID: rs572025435** | **protein position 30 changed from Arginine (green (upper image)) to Serine (red (bottom image))** | | | | | |
|  |  |  | |  | |  |
|  |  |  | |  | |  |
| **SNP ID: rs1390270595** | **protein position 51 changed from Tyrosine (green (upper image)) to Cysteine (red (bottom image))** | | | | | |
|  |  |  | |  | |  |
|  |  |  | |  | |  |
| **SNP ID: rs763201540** | **protein position 53 changed from Aspartic acid (green (upper image)) to Asparagine (red (bottom image))** | | | | | |
|  |  |  | |  | |  |
|  |  |  | |  | |  |
| **SNP ID: rs763201540** | **protein position 53 changed from Aspartic acid (green (upper image)) to Tyrosine (red (bottom image))** | | | | | |
|  |  |  | |  | |  |
|  |  |  | |  | |  |
| **SNP ID: rs1414848134** | **protein position 54 changed from Aspartic acid (green (upper image)) to Valine (red (bottom image))** | | | | | |
|  |  |  | |  | |  |
|  |  |  | |  | |  |
| **SNP ID: rs1260086927** | **protein position 96 changed from Glutamine (green (upper image)) to Proline (red (bottom image))** | | | | | |
|  |  | |  |  | |  |
|  |  | |  |  | |  |
| **SNP ID: rs770412396** | **protein position 102 changed from Leucine (green (upper image)) to Proline (red (bottom image))** | | | | | |
|  |  | |  |  | |  |
|  |  | |  |  | |  |
| **SNP ID: rs1161818149** | **protein position 105 changed from Leucine (green (upper image)) to Glutamine (red (bottom image))** | | | | | |
|  |  | |  |  | |  |
|  |  | |  |  | |  |
| **SNP ID: rs1161818149** | **protein position 105 changed from Leucine (green (upper image)) to Proline (red (bottom image))** | | | | | |
|  |  | |  |  | |  |
|  |  | |  |  | |  |

**Supplementary Table 20.** Graphical representations of amino acid changes due to the most deleterious SNPs in soluble isoforms

| **Graphical representations of amino acid changes due to most deleterious SNPs in soluble isoforms** | | |  |
| --- | --- | --- | --- |
| **Isoform 5** | **Isoform 6** | **Isoform 7** |  |
| **protein position 29 changed from Methionine (green (upper image)) to Lysine (red (bottom image))** | | |  |
| **SNP ID:** **rs555347515** |  |  |  |
|  |  |  |  |
| **SNP ID: rs540632198** | **protein position 32 changed from Phenyalanine (green (upper image)) to** [**Cysteine**](https://en.wikipedia.org/wiki/Cysteine) **(red (bottom image))** | | |
|  |  |  |  |
|  |  |  |  |
| **SNP ID: rs1390270595** | **protein position 51 changed from Tyrosine (green (upper image)) to Cysteine (red (bottom image))** | | |
|  |  |  |  |
|  |  |  |  |
| **SNP ID: rs763201540** | **protein position 53 changed from Aspartic acid (green (upper image)) to Asparagine (red (bottom image))** | | |
|  |  |  |  |
|  |  |  |  |
| **SNP ID: rs763201540** | **protein position 53 changed from Aspartic acid (green (upper image)) to Tyrosine (red (bottom image))** | | |
|  |  |  |  |
|  |  |  |  |
| **SNP ID: rs1414848134** | **protein position 54 changed from Aspartic acid (green (upper image)) to Valine (red (bottom image))** | | |
|  |  |  |  |
|  |  |  |  |
| **SNP ID: rs1260086927** | **protein position 96 changed from Glutamine (green (upper image)) to Proline (red (bottom image))** | | |
|  |  |  |  |
|  |  |  |  |
| **SNP ID: rs770412396** | **protein position 102 changed from Leucine (green (upper image)) to Proline (red (bottom image))** | | |
|  |  |  |  |
|  |  |  |  |
| **SNP ID: rs1161818149** | **protein position 105 changed from Leucine (green (upper image)) to Proline (red (bottom image))** | | |
|  |  |  |  |
|  |  |  |  |
