# Supplementary material for: Predicting the most deleterious missense nsSNPs of the protein isoforms of the human HLA-G gene and in silico evaluation of their structural and functional consequences
Source: BMC Genet. 2020 Aug 31;21:94. doi: 10.1186/s12863-020-00890-y (PMC7457528; doi:10.1186/s12863-020-00890-y)
Supplement: Supplementary file 2 — Table 5. Analysis of structural effects of deleterious SNPs on HLA-G1 by Project HOPE [file 12863_2020_890_MOESM2_ESM.doc]

**Table 5.** Analysis of structural effects of deleterious SNPs on HLA-G1 by Project HOPE.

| **M29K** | **Amino acid properties** | The wild-type residue was buried in the core of the protein. The mutant residue is bigger and probably will not fit.  The mutant residue introduces a positive charge in a buried residue which can lead to protein folding problems.  The wild-type residue is more hydrophobic than the mutant residue.  The hydrophobic interactions in the core of the protein will lose due to the mutation. | 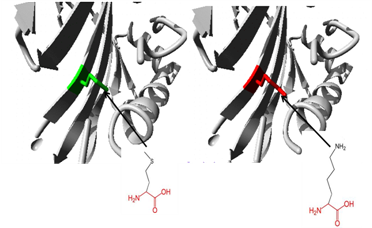 |
| --- | --- | --- | --- |
|  | **Structure** | The mutation is located within a stretch of residues annotated in UniProt as a special region: Alpha-1. The diversities in residue characteristics can distort this region and distort its function. |  |
| **R30S** | **Amino acid properties** | The mutant residue is smaller than the wild-type residue.  The wild-type amino acid has a positive charge while the mutant amino acid has a neutral charge.  The charge of the wild-type residue is lost by this mutation. This can cause loss of interactions with other molecules.  The mutant residue is more hydrophobic than the wild-type residue. | 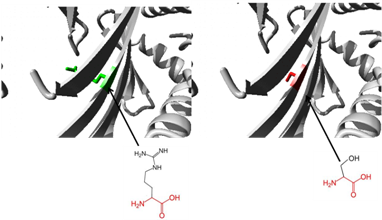 |
|  | **Structure** | The mutation is located within a stretch of residues annotated in UniProt as a special region: Alpha-1. The diversities in residue characteristics can distort this region and distort its function. |  |
| **P39L** | **Amino acid properties** | The mutant residue is bigger than the wild-type residue.  The amino acid is located on the surface of the protein; mutation of this amino acid can distort interactions with other molecules or other sections of the protein.  The torsion angles for the wild-type residue are within the small range for proline allowed angles. Possibly, the proline is required at this position for rigidity and stabilization of the structure. Mutation into a more flexible residue will destabilize the structure. | 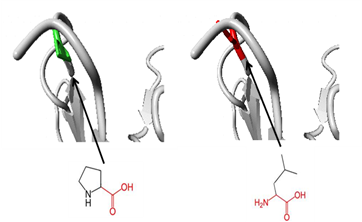 |
|  | **Structure** | The mutation is located within a stretch of residues annotated in UniProt as a special region: Alpha-1. The diversities in residue characteristics can distort this region and distort its function.  The wild-type residue is proline. Prolines are known to be very rigid and therefore induce a special backbone conformation that might be required at this position. The mutation can disturb this special conformation. |  |
| **Y51C** | **Amino acid properties** | The mutant residue is smaller than the wild-type residue, which will cause a possible loss of external interactions.  The mutant residue is more hydrophobic than the wild-type residue. | 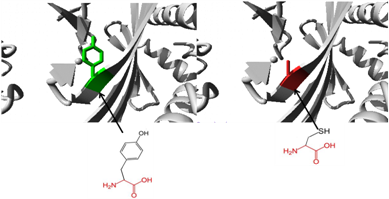 |
|  | **Structure** | The mutation is located within a stretch of residues annotated in UniProt as a special region: Alpha-1. The diversities in residue characteristics can distort this region and distort its function. |  |
| **D53N** | **Amino acid properties** | The wild-type residue charge was negative; the mutant residue charge is neutral.  The charge of the wild-type residue is lost by this mutation. This can cause loss of interactions with other molecules. | 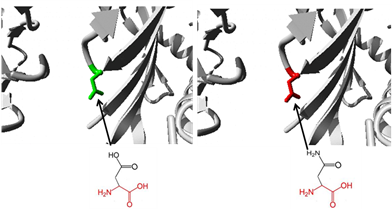 |
|  | **Structure** | The mutation is located within a stretch of residues annotated in UniProt as a special region: Alpha-1. The diversities in residue characteristics can distort this region and distort its function. |  |
| **D53Y** | **Amino acid  properties** | The mutant residue is bigger than the wild-type residue.  The amino acid is located on the surface of the protein; mutation of this amino acid can distort interactions with other molecules or other sections of the protein.  The wild-type residue charge was negative; the mutant residue charge is neutral.  The charge of the wild-type residue is lost by this mutation. This can cause loss of interactions with other molecules.  The mutant residue is more hydrophobic than the wild-type residue. | 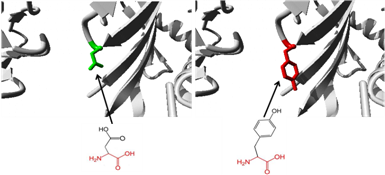 |
|  | **Structure** | The mutation is located within a stretch of residues annotated in UniProt as a special region: Alpha-1. The diversities in residue characteristics can distort this region and distort its function. |  |
| **D54V** | **Amino acid properties** | The mutant residue is smaller than the wild-type residue, which will cause a possible loss of external interactions.  The wild-type residue charge was negative; the mutant residue charge is neutral.  The charge of the wild-type residue is lost by this mutation. This can cause loss of interactions with other molecules.  The mutant residue is more hydrophobic than the wild-type residue. | 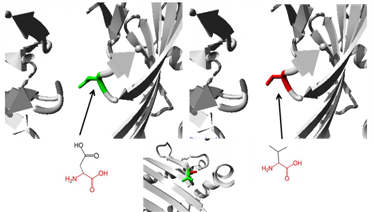 |
|  | **Structure** | The mutation is located within a stretch of residues annotated in UniProt as a special region: Alpha-1. The diversities in residue characteristics can distort this region and distort its function. |  |
| **Q96P** | **Amino acid properties** | The mutant residue is smaller than the wild-type residue, which will cause a possible loss of external interactions.  The mutant residue is more hydrophobic than the wild-type residue. | 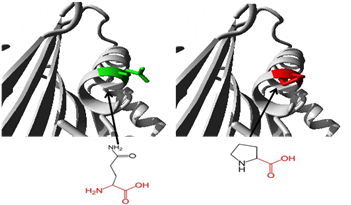 |
|  | **Structure** | The mutation is located within a stretch of residues annotated in UniProt as a special region: Alpha-1. The diversities in residue characteristics can distort this region and distort its function. |  |
| **L102P** | **Amino acid properties** | The mutant residue is smaller than the wild-type residue, which will cause an empty space in the core of the protein. | 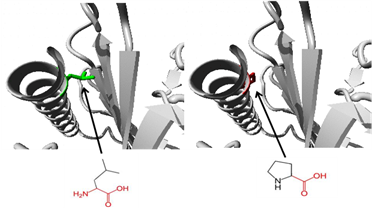 |
|  | **Structure** | The mutation is located within a stretch of residues annotated in UniProt as a special region: Alpha-1. The diversities in residue characteristics can distort this region and distort its function.  In the case of the mutation, the helix will be disturbed and this can have severe effects on the structure of the protein. |  |
| **L105Q** | **Amino acid properties** | The mutant residue is bigger than the wild-type residue.  The wild-type residue was buried in the core of the protein. The mutant residue is bigger and probably will not fit.  The wild-type residue is more hydrophobic than the mutant residue.  The hydrophobic interactions in the core of the protein will lose due to the mutation. | 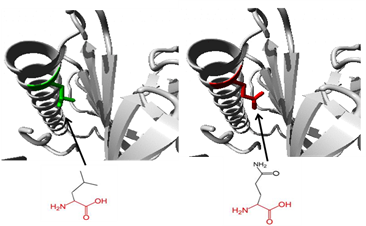 |
|  | **Structure** | The mutation is located within a stretch of residues annotated in UniProt as a special region: Alpha-1. The diversities in residue characteristics can distort this region and distort its function. |  |
| **L105P** | **Amino acid properties** | The mutant residue is smaller than the wild-type residue, which will cause an empty space in the core of the protein. | 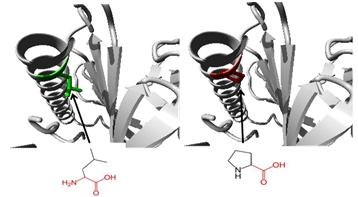 |
|  | **Structure** | The mutation is located within a stretch of residues annotated in UniProt as a special region: Alpha-1. The diversities in residue characteristics can distort this region and distort its function.  Alpha -helix is disrupted when proline not located at one of the first three positions of that helix.  In case of the mutation at hand, the helix will be disturbed and this can have severe effects on the structure of the protein. |  |
| **H117P** | **Amino acid  properties** | The mutant residue is smaller than the wild-type residue, which will cause an empty space in the core of the protein.  The mutant residue is more hydrophobic than the wild-type residue.  The mutation will cause loss of hydrogen bonds in the core of the protein and as a result, disturb correct folding. | 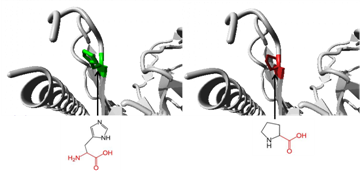 |
|  | **Structure** | The mutation is located within a stretch of residues annotated in UniProt as a special region: Alpha-2. The diversities in residue characteristics can distort this region and distort its function. |  |
| **H117L** | **Amino acid properties** | The mutant residue is smaller than the wild-type residue, which will cause an empty space in the core of the protein.  The mutant residue is more hydrophobic than the wild-type residue.  The mutation will cause loss of hydrogen bonds in the core of the protein and as a result, disturb correct folding. | 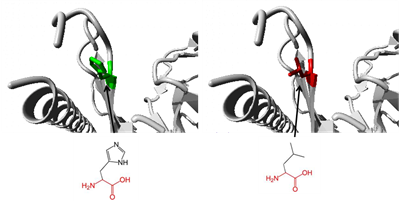 |
|  | **Structure** | The mutation is located within a stretch of residues annotated in UniProt as a special region: Alpha-2. The diversities in residue characteristics can distort this region and distort its function. |  |
| **D130H** | **Amino acid properties** | The mutant residue is bigger than the wild-type residue.  The amino acid is located on the surface of the protein; mutation of this amino acid can distort interactions with other molecules or other sections of the protein.  The wild-type residue charge was negative; the mutant residue charge is neutral.  The charge of the wild-type residue is lost by this mutation. This can cause loss of interactions with other molecules. | 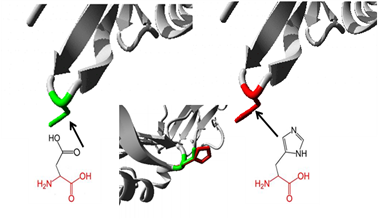 |
|  | **Structure** | The mutation is located within a stretch of residues annotated in UniProt as a special region: Alpha-2. The diversities in residue characteristics can distort this region and distort its function. |  |
| **Y142C** | **Amino acid properties** | The mutant residue is smaller than the wild-type residue, which will cause an empty space in the core of the protein.  The mutant residue is more hydrophobic than the wild-type residue.  The mutation will cause loss of hydrogen bonds in the core of the protein and as a result disturb correct folding. | 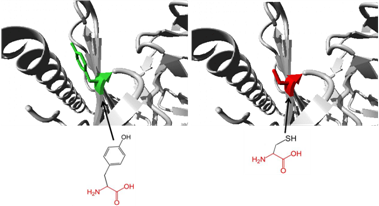 |
|  | **Structure** | The mutation is located within a stretch of residues annotated in UniProt as a special region: Alpha-2. The diversities in residue characteristics can distort this region and distort its function. |  |
| **Y142H** | **Amino acid properties** | The mutant residue is smaller than the wild-type residue, which will cause an empty space in the core of the protein  The wild-type residue is more hydrophobic than the mutant residue.  The hydrophobic interactions in the core of the protein will lose due to the mutation. | 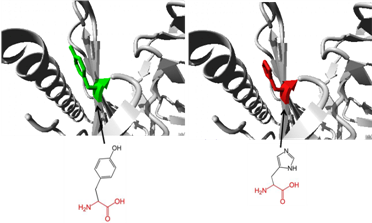 |
|  | **Structure** | The mutation is located within a stretch of residues annotated in UniProt as a special region: Alpha-2. The differences in amino acid properties can disturb this region and disturb its function. |  |
| **D143N** | **Amino acid properties** | The wild-type residue charge was negative; the mutant residue charge is neutral.  The charge of the wild-type residue is lost by this mutation. This can cause loss of interactions with other molecules. | 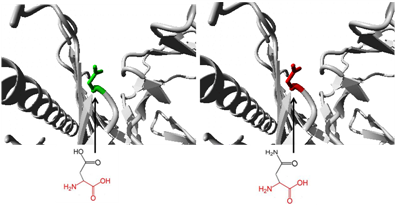 |
|  | **Structure** | The mutation is located within a stretch of residues annotated in UniProt as a special region: Alpha-2. The diversities in residue characteristics can distort this region and distort its function. |  |
| **D143H** | **Amino acid  properties** | The mutant residue is bigger than the wild-type residue.  The amino acid is located on the surface of the protein; mutation of this amino acid can distort interactions with other molecules or other sections of the protein.  The wild-type residue charge was negative; the mutant residue charge is neutral.  The charge of the wild-type residue is lost by this mutation. This can cause loss of interactions with other molecules. | 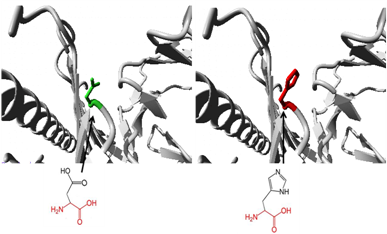 |
|  | **Structure** | The mutation is located within a stretch of residues annotated in UniProt as a special region: Alpha-2. The diversities in residue characteristics can distort this region and distort its function. |  |
| **D153G** | **Amino acid  properties** | The mutant residue is smaller than the wild-type residue, which will cause a possible loss of external interactions.  The wild-type residue charge was negative; the mutant residue charge is neutral.  The charge of the wild-type residue is lost by this mutation. This can cause loss of interactions with other molecules.  The mutant residue is more hydrophobic than the wild-type residue. | 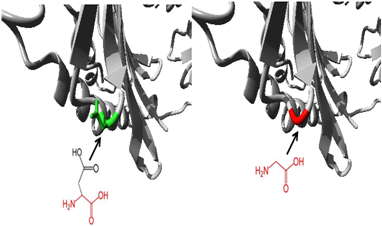 |
|  | **Structure** | The mutation is located within a stretch of residues annotated in UniProt as a special region: Alpha-2. The diversities in residue characteristics can distort this region and distort its function.  The mutation introduces a glycine at this position. Glycines are very flexible and can disturb the required rigidity of the protein at this position. |  |
| **W157R** | **Amino acid  properties** | The mutant residue is smaller than the wild-type residue, which will cause an empty space in the core of the protein.  The wild-type amino acid has a neutral charge while the mutant amino acid has a positive charge.  The mutant residue introduces a charge in a buried residue which can lead to protein folding problems.  The wild-type residue is more hydrophobic than the mutant residue.  The hydrophobic interactions in the core of the protein will lose due to the mutation. | 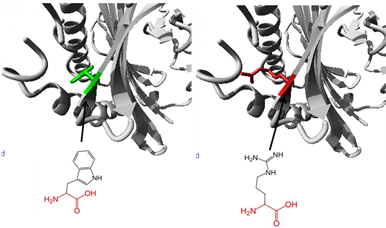 |
|  | **Structure** | The mutation is located within a stretch of residues annotated in UniProt as a special region: Alpha-2. The diversities in residue characteristics can distort this region and distort its function. |  |
| **T158P** | **Amino acid  properties** | The mutant residue is more hydrophobic than the wild-type residue. | 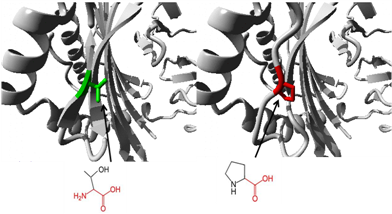 |
|  | **Structure** | The mutation is located within a stretch of residues annotated in UniProt as a special region: Alpha-2. The diversities in residue characteristics can distort this region and distort its function. |  |
| **C188S** | **Amino acid  properties** | The wild-type residue is more hydrophobic than the mutant residue.  The hydrophobic interactions in the core of the protein will lose due to the mutation. | 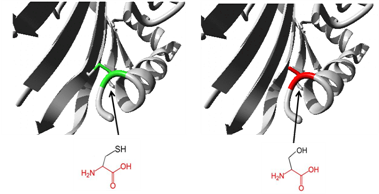 |
|  | **Structure** | The mutation is located within a stretch of residues annotated in UniProt as a special region: Alpha-2. The diversities in residue characteristics can distort this region and distort its function. |  |
| **L196P** | **Amino acid  properties** | The mutant residue is smaller than the wild-type residue, which will cause an empty space in the core of the protein. | 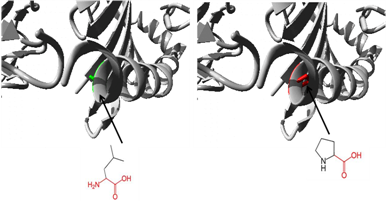 |
|  | **Structure** | The mutation is located within a stretch of residues annotated in UniProt as a special region: Alpha-2. The diversities in residue characteristics can distort this region and distort its function.  In case of the mutation, the helix will be disturbed and this can have severe effects on the structure of the protein. |  |
| **R205S** | **Amino acid  properties** | The mutant residue is smaller than the wild-type residue, which will cause a possible loss of external interactions.  The wild-type amino acid has a positive charge while the mutant amino acid has a neutral charge.  The charge of the wild-type residue is lost by this mutation. This can cause loss of interactions with other molecules.  The mutant residue is more hydrophobic than the wild-type residue. | 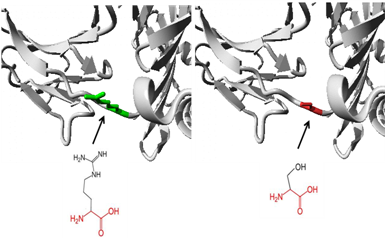 |
|  | **Structure** | The mutation is located within a stretch of residues annotated in UniProt as a special region: Alpha-2. The diversities in residue characteristics can distort this region and distort its function. |  |
| **P209A** | **Amino acid  properties** | The mutant residue is smaller than the wild-type residue, which will cause an empty space in the core of the protein. | 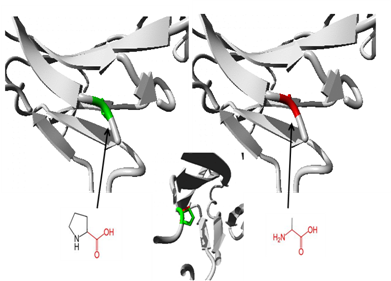 |
|  | **Structure** | The mutation is located within a domain, annotated in UniProt as Ig-like C1-type  The mutant amino acid has diverse characteristics than the wild amino acid that can distort this domain and abolish its function.  The mutation is located within a stretch of residues annotated in UniProt as a special region: Alpha-3. The diversities in residue characteristics can distort this region and distort its function.  The wild-type residue is proline. Prolines are known to be very rigid and therefore induce a special backbone conformation that might be required at this position. This special conformation can be disturbed due to the mutation. |  |
| **P209R** | **Amino acid  properties** | The mutant residue is bigger than the wild-type residue.  The wild-type residue was buried in the core of the protein. The mutant residue is bigger and probably will not fit.  The wild-type amino acid has a neutral charge while the mutant amino acid has a positive charge.  The mutant residue introduces a charge in a buried residue which can lead to protein folding problems.  The wild-type residue is more hydrophobic than the mutant residue.  The hydrophobic interactions in the core of the protein will lose due to the mutation.. | 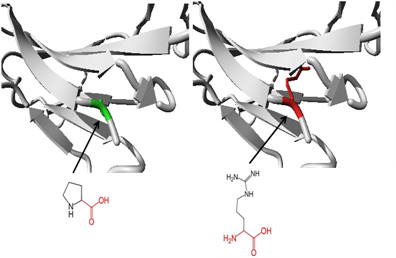 |
|  | **Structure** | The mutation is located within a domain, annotated in UniProt as Ig-like C1-type  The mutant amino acid has diverse characteristics than the wild amino acid that can distort this domain and abolish its function.  The mutation is located within a stretch of residues annotated in UniProt as a special region: Alpha-3. The diversities in residue characteristics can distort this region and distort its function.  The wild-type residue is proline. Prolines are known to be very rigid and therefore induce a special backbone conformation that might be required at this position. This special conformation can be disturbed due to the mutation. |  |
| **C227F** | **Amino acid  properties** | The mutant residue is bigger than the wild-type residue.  The wild-type residue was buried in the core of the protein. The mutant residue is bigger and probably will not fit. | 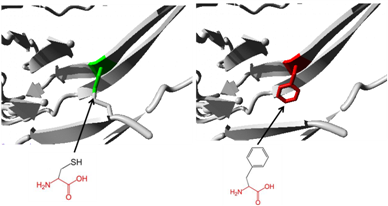 |
|  | **Structure** | The mutation is located within a domain, annotated in UniProt as Ig-like C1-type  The mutant amino acid has diverse characteristics than the wild amino acid that can distort this domain and abolish its function.  The mutation is located within a stretch of residues annotated in UniProt as a special region: Alpha-3. The diversities in residue characteristics can distort this region and distort its function. |  |
| **C227Y** | **Amino acid  properties** | The mutant residue is bigger than the wild-type residue.  The wild-type residue was buried in the core of the protein. The mutant residue is bigger and probably will not fit.  The wild-type residue is more hydrophobic than the mutant residue.  The hydrophobic interactions in the core of the protein will lose due to the mutation. | 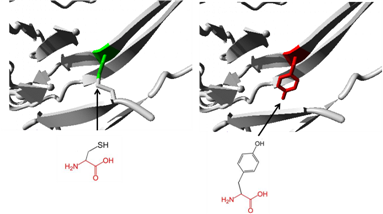 |
|  | **Structure** | The mutation is located within a domain, annotated in UniProt as Ig-like C1-type  The mutant amino acid has diverse characteristics than the wild amino acid that can distort this domain and abolish its function.  The mutation is located within a stretch of residues annotated in UniProt as a special region: Alpha-3. The diversities in residue characteristics can distort this region and distort its function. |  |
| **A229D** | **Amino acid  properties** | The mutant residue is bigger than the wild-type residue.  The wild-type residue was buried in the core of the protein. The mutant residue is bigger and probably will not fit.  The wild-type amino acid has a neutral charge while the mutant amino acid has a negative charge.  The mutant residue introduces a charge in a buried residue which can lead to protein folding problems.  The wild-type residue is more hydrophobic than the mutant residue.  The hydrophobic interactions in the core of the protein will lose due to the mutation. | 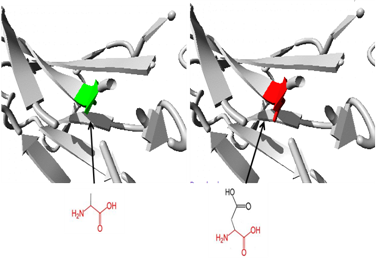 |
|  | **Structure** | The mutation is located within a domain, annotated in UniProt as Ig-like C1-type  The mutant amino acid has diverse characteristics than the wild amino acid that can distort this domain and abolish its function.  The mutation is located within a stretch of residues annotated in UniProt as a special region: Alpha-3. The diversities in residue characteristics can distort this region and distort its function. |  |
| **P234T** | **Amino acid  properties** | The wild-type residue is more hydrophobic than the mutant residue.  The hydrophobic interactions with other molecules on the surface of the protein might lose due to the mutation. | 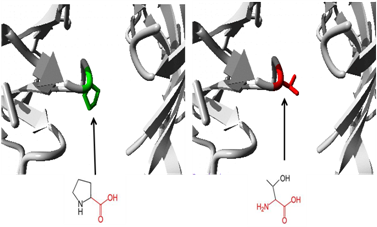 |
|  | **Structure** | The mutation is located within a domain, annotated in UniProt as Ig-like C1-type  The mutant amino acid has diverse characteristics than the wild amino acid that can distort this domain and abolish its function.  The mutation is located within a stretch of residues annotated in UniProt as a special region: Alpha-3. The diversities in residue characteristics can distort this region and distort its function.  The wild-type residue is proline. Prolines are known to be very rigid and therefore induce a special backbone conformation that might be required at this position. The mutation can disturb this special conformation. |  |
| **P234L** | **Amino acid  properties** | The mutant residue is bigger than the wild-type residue.  The amino acid is located on the surface of the protein; mutation of this amino acid can distort interactions with other molecules or other sections of the protein. | 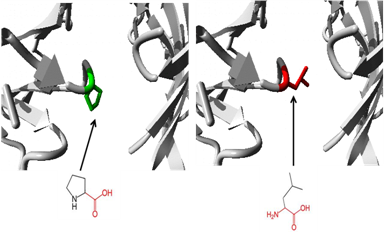 |
|  | **Structure** | The mutation is located within a domain, annotated in UniProt as Ig-like C1-type  The mutant amino acid has diverse characteristics than the wild amino acid that can distort this domain and abolish its function.  The mutation is located within a stretch of residues annotated in UniProt as a special region: Alpha-3. The diversities in residue characteristics can distort this region and distort its function.  The wild-type residue is proline. Prolines are known to be very rigid and therefore induce a special backbone conformation that might be required at this position. This special conformation can be disturbed due to the mutation. |  |
| **I237F** | **Amino acid  properties** | The mutant residue is bigger than the wild-type residue.  The wild-type residue was buried in the core of the protein. The mutant residue is bigger and probably will not fit. | 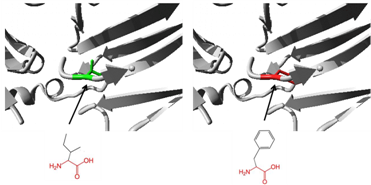 |
|  | **Structure** | The mutation is located within a domain, annotated in UniProt as Ig-like C1-type  The mutant amino acid has diverse characteristics than the wild amino acid that can distort this domain and abolish its function.  The mutation is located within a stretch of residues annotated in UniProt as a special region: Alpha-3. The diversities in residue characteristics can distort this region and distort its function. |  |
| **D244G** | **Amino acid  properties** | The mutant residue is smaller than the wild-type residue, which will cause a possible loss of external interactions.  The wild-type residue charge was negative; the mutant residue charge is neutral.  The charge of the wild-type residue is lost by this mutation. This can cause loss of interactions with other molecules.  The mutant residue is more hydrophobic than the wild-type residue. | 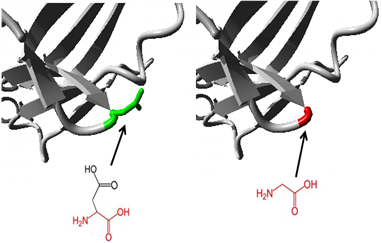 |
|  | **Structure** | The mutation is located within a domain, annotated in UniProt as Ig-like C1-type  The mutant amino acid has diverse characteristics than the wild amino acid that can distort this domain and abolish its function.  The mutation is located within a stretch of residues annotated in UniProt as a special region: Alpha-3. The diversities in residue characteristics can distort this region and distort its function.  The glycine amino acid introduces in this position due to the mutation. Glycines are very flexible and can disturb the required rigidity of the protein at this position. |  |
| **P259H** | **Amino acid  properties** | The mutant residue is bigger than the wild-type residue.  The amino acid is located on the surface of the protein; mutation of this amino acid can distort interactions with other molecules or other sections of the protein.  The wild-type residue is more hydrophobic than the mutant residue.  The hydrophobic interactions with other molecules on the surface of the protein might lose due to the mutation. | 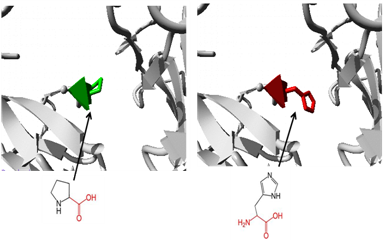 |
|  | **Structure** | The mutation is located within a domain, annotated in UniProt as Ig-like C1-type  The mutant amino acid has diverse characteristics than the wild amino acid that can distort this domain and abolish its function.  The mutation is located within a stretch of residues annotated in UniProt as a special region: Alpha-3. The diversities in residue characteristics can distort this region and distort its function.  The wild-type residue is proline. Prolines are known to be very rigid and therefore induce a special backbone conformation that might be required at this position. The mutation can disturb this special conformation. |  |
| **Q266L** | **Amino acid  properties** | The mutant residue is smaller than the wild-type residue, which will cause a possible loss of external interactions.  The mutant residue is more hydrophobic than the wild-type residue. | 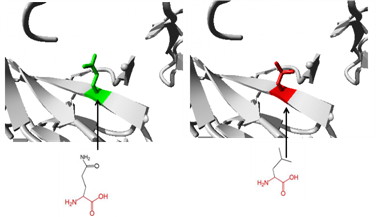 |
|  | **Structure** | The mutation is located within a domain, annotated in UniProt as Ig-like C1-type  The mutant amino acid has diverse characteristics than the wild amino acid that can distort this domain and abolish its function.  The mutation is located within a stretch of residues annotated in UniProt as a special region: Alpha-3. The differences in amino acid properties can disturb this region and disturb its function. |  |
| **V285A** | **Amino acid  properties** | The mutant residue is smaller than the wild-type residue, which will cause an empty space in the core of the protein. | 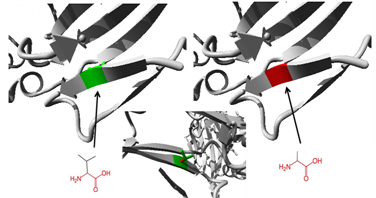 |
|  | **Structure** | The mutation is located within a domain, annotated in UniProt as Ig-like C1-type  The mutant amino acid has diverse characteristics than the wild amino acid that can distort this domain and abolish its function.  The mutation is located within a stretch of residues annotated in UniProt as a special region: Alpha-3. The diversities in residue characteristics can distort this region and distort its function. |  |
| **H287Y** | **Amino acid  properties** | The mutant residue is bigger than the wild-type residue.  The wild-type residue was buried in the core of the protein. The mutant residue is bigger and probably will not fit.  The mutant residue is more hydrophobic than the wild-type residue.  The mutation will cause loss of hydrogen bonds in the core of the protein and as a result disturb correct folding. | 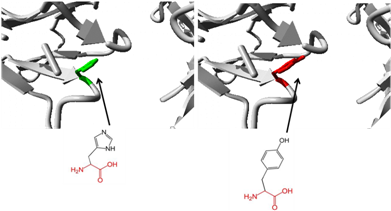 |
|  | **Structure** | The mutation is located within a domain, annotated in UniProt as Ig-like C1-type  The mutant amino acid has diverse characteristics than the wild amino acid that can distort this domain and abolish its function.  The mutation is located within a stretch of residues annotated in UniProt as a special region: Alpha-3. The diversities in residue characteristics can distort this region and distort its function. |  |
| **L290R** | **Amino acid  properties** | The wild-type amino acid has a neutral charge while the mutant amino acid has a positive charge.  The mutant residue introduces a charge in a buried residue which can lead to protein folding problems.  The mutant residue is bigger than the wild-type residue.  The wild-type residue was buried in the core of the protein. The mutant residue is bigger and probably will not fit.  The wild-type residue is more hydrophobic than the mutant residue. The hydrophobic interactions in the core of the protein will lose due to the mutation. | 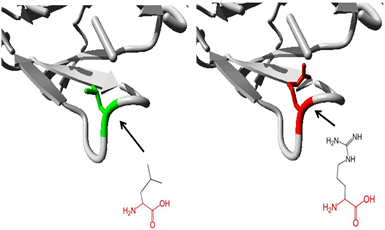 |
|  | **Structure** | The mutation is located within a domain, annotated in UniProt as Ig-like C1-type  The mutant amino acid has diverse characteristics than the wild amino acid that can distort this domain and abolish its function.  The mutation is located within a stretch of residues annotated in UniProt as a special region: Alpha-3. The diversities in residue characteristics can distort this region and distort its function. |  |
| **L294R** | **Amino acid  properties** | The wild-type amino acid has a neutral charge while the mutant amino acid has a positive charge.  The mutation introduces a charge at this position; this can cause repulsion between the mutant residue and neighboring residues.  The mutant residue is bigger than the wild-type residue.  The amino acid is located on the surface of the protein; mutation of this amino acid can distort interactions with other molecules or other sections of the protein.  The wild-type residue is more hydrophobic than the mutant residue.  The hydrophobic interactions with other molecules on the surface of the protein might lose due to the mutation. | 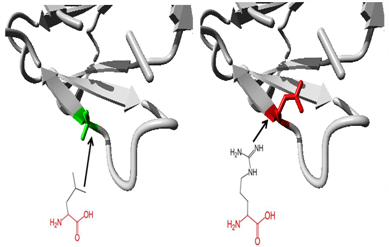 |
|  | **Structure** | The mutation is located within a domain, annotated in UniProt as Ig-like C1-type  The mutant amino acid has diverse characteristics than the wild amino acid that can distort this domain and abolish its function.  The mutation is located within a stretch of residues annotated in UniProt as a special region: Alpha-3. The diversities in residue characteristics can distort this region and distort its function. |  |
| **W298R** | **Amino acid  properties** | The wild-type amino acid has a neutral charge while the mutant amino acid has a positive charge.  The mutation introduces a charge at this position; this can cause repulsion between the mutant residue and neighboring residues.  The mutant residue is smaller than the wild-type residue, which will cause a possible loss of external interactions.  The wild-type residue is more hydrophobic than the mutant residue.  The hydrophobic interactions with other molecules on the surface of the protein might lose due to the mutation. | 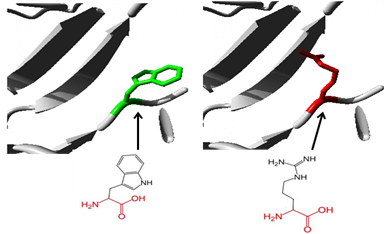 |
|  | **Structure** | The mutation is located within a domain, annotated in UniProt as Ig-like C1-type.  The mutant amino acid has diverse characteristics than the wild amino acid that can distort this domain and abolish its function.  The mutation is located within a stretch of residues annotated in UniProt as a special region: Alpha-3. The diversities in residue characteristics can distort this region and distort its function. |  |

The effects of each of the most deleterious predicted SNPs on native HLA-G 1 isoform structure and the difference in physicochemical properties of amino acids of wild type and mutated residue as predicted by HOPE. Each close-up image represents the 3D structure of the HLA-G1 isoform (gray color) with its wild type residue (green color) and mutant residue (red color).
