# Supplementary material for: Predicting the most deleterious missense nsSNPs of the protein isoforms of the human HLA-G gene and in silico evaluation of their structural and functional consequences
Source: BMC Genet. 2020 Aug 31;21:94. doi: 10.1186/s12863-020-00890-y (PMC7457528; doi:10.1186/s12863-020-00890-y)
Supplement: Supplementary file 3 — Table 6. Five models predicted for each human HLA-G isoform by I-TASSER [file 12863_2020_890_MOESM3_ESM.doc]

**Table 6.** Five models predicted for each human HLA-G isoform by I-TASSER.

| **Isoform 1** | | | | |
| --- | --- | --- | --- | --- |
| **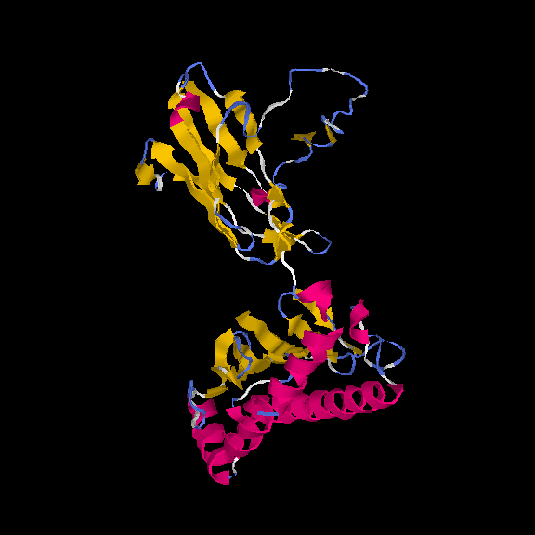** | 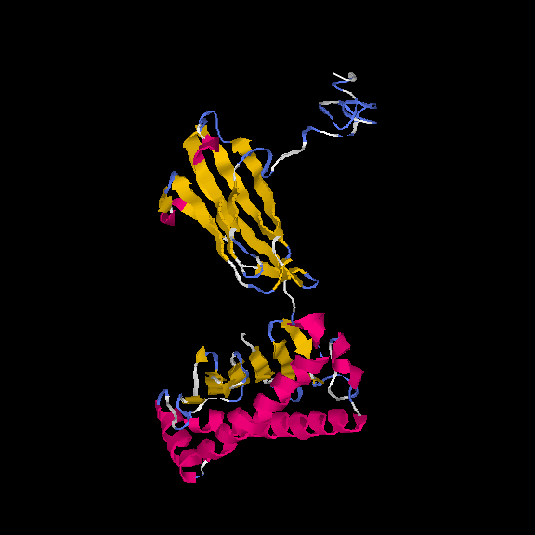 | 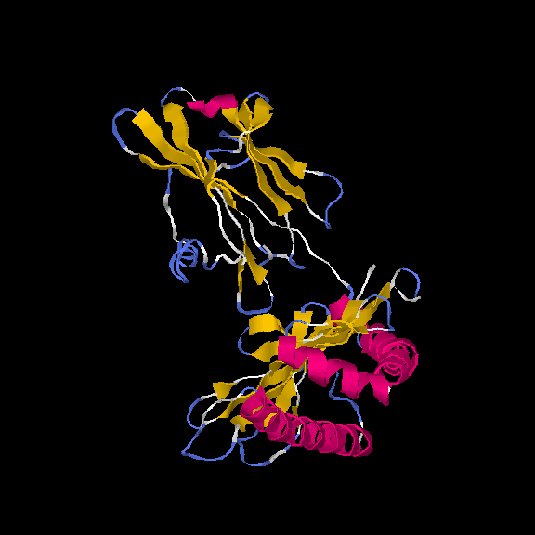 | 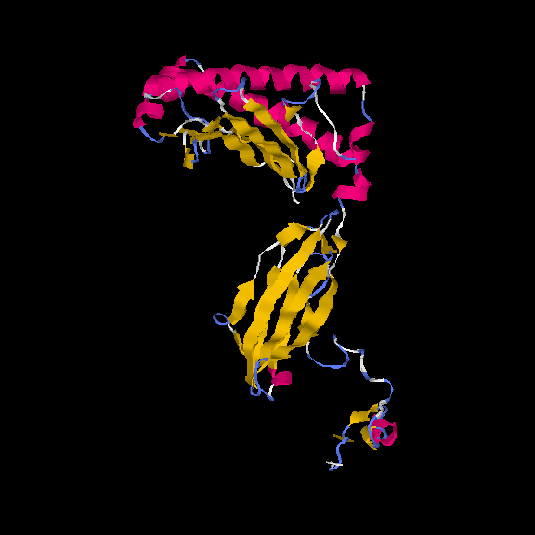 | 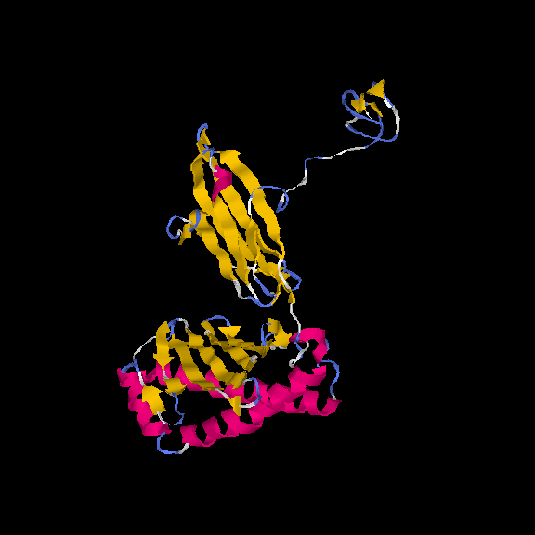 |
| **Model 1 C-score= -0.23  Estimated TM-score = 0.68±0.12 Estimated RMSD = 6.8±4.0Å** | Model 2 C-score = -1.17 | Model 3 C-score = -2.22 | Model 4 C-score = -2.99 | Model 5 C-score = -1.71 |
| **Isoform 2** | | | | |
| **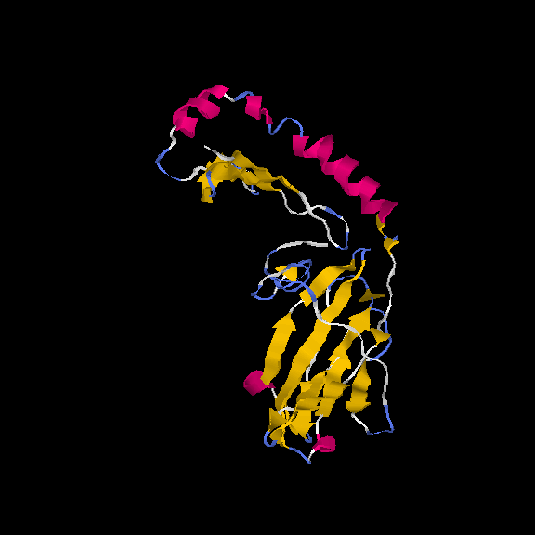** | 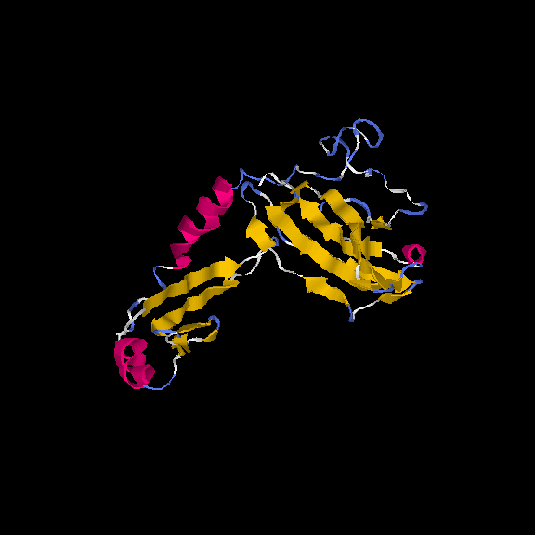 | 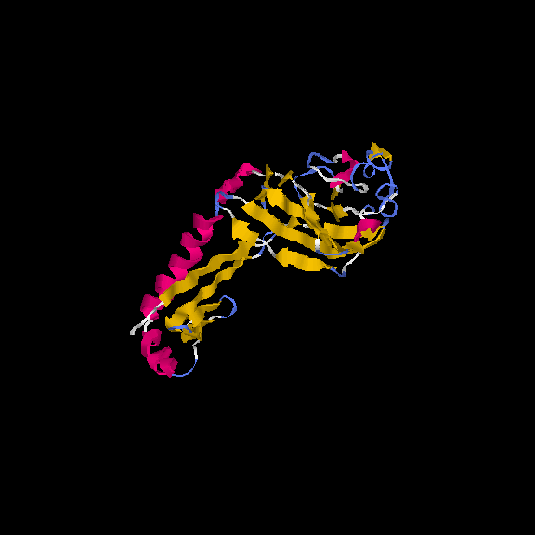 | 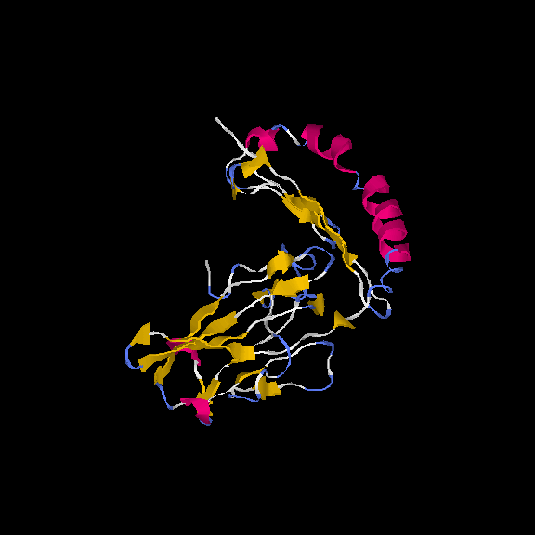 | 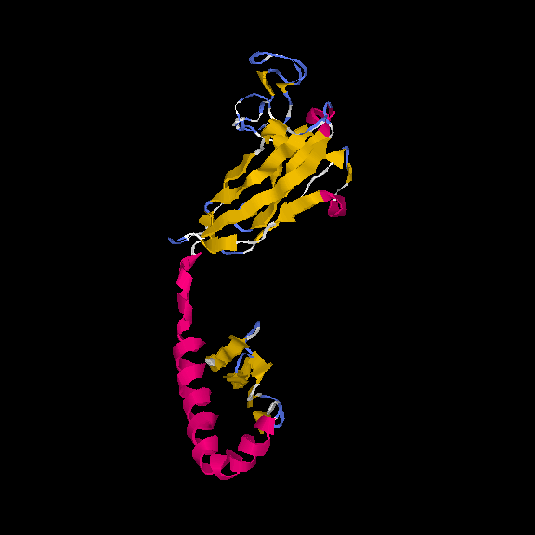 |
| **Model 1 C-score=-1.63  Estimated TM-score = 0.52±0.15 Estimated RMSD = 9.2±4.6Å** | Model2 C-score = -2.56 | Model 3 C-score = -3.01 | Model 4 C-score = -2.58 | Model 5 C-score = -3.12 |
| **Isoform 3** | | | | |
| **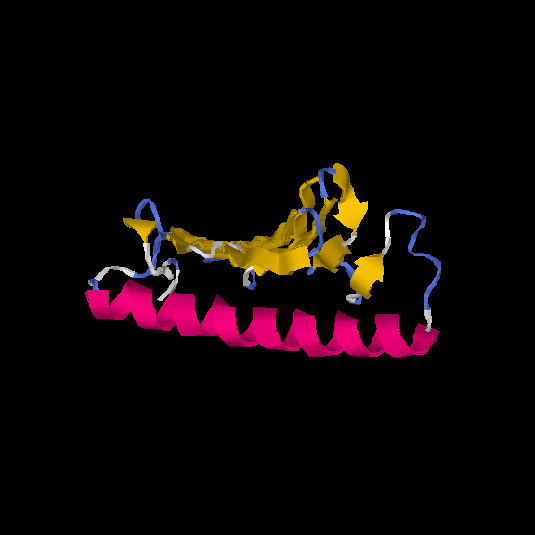** | 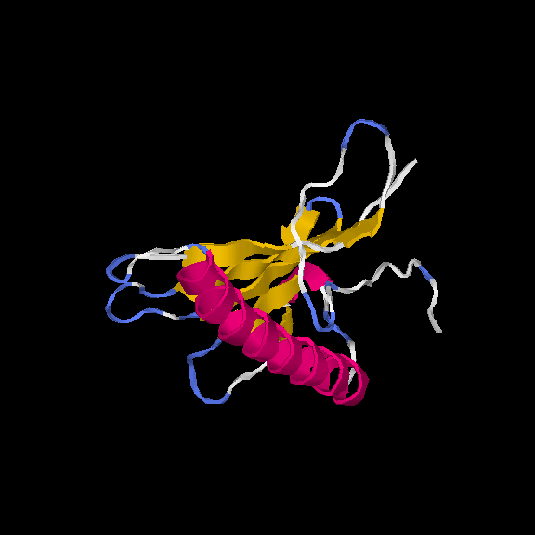 | 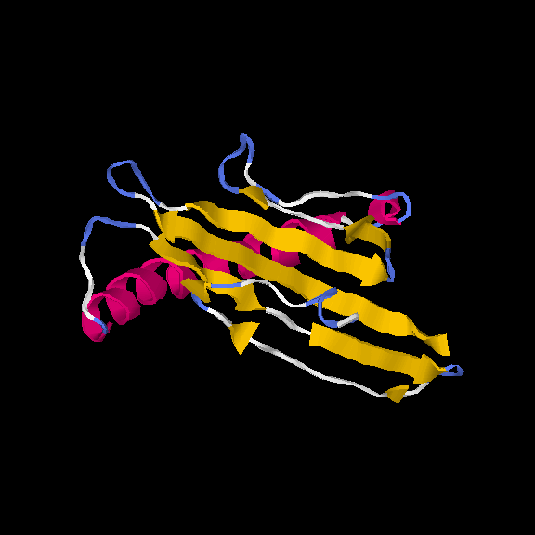 | 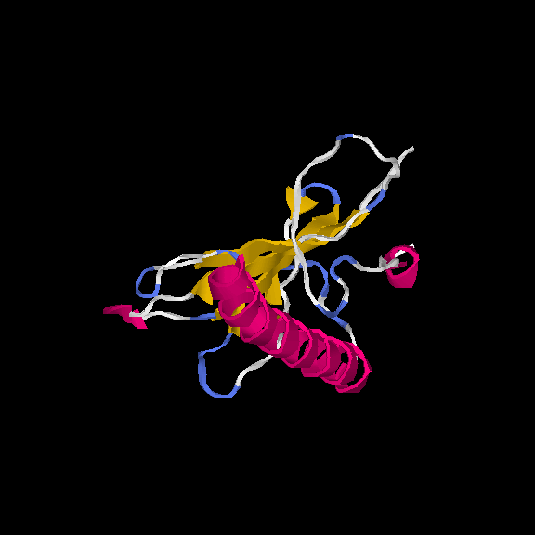 | 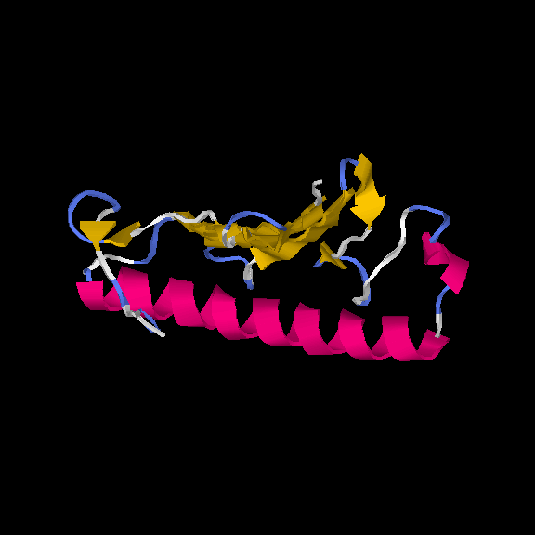 |
| **Model 1 C-score= 0.75  Estimated TM-score = 0.81±0.09 Estimated RMSD = 3.0±2.2Å** | Model2 C-score = -5.00 | Model 3 C-score = -4.92 | Model 4 C-score = -5 | Model 5 C-score = -5 |
| **Isoform 4** | | | | |
| **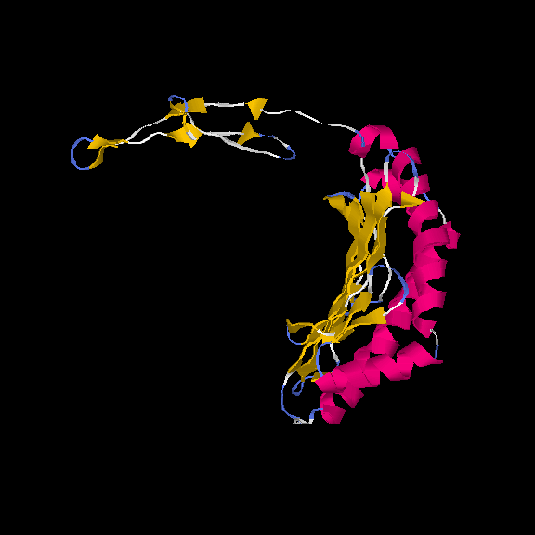** | 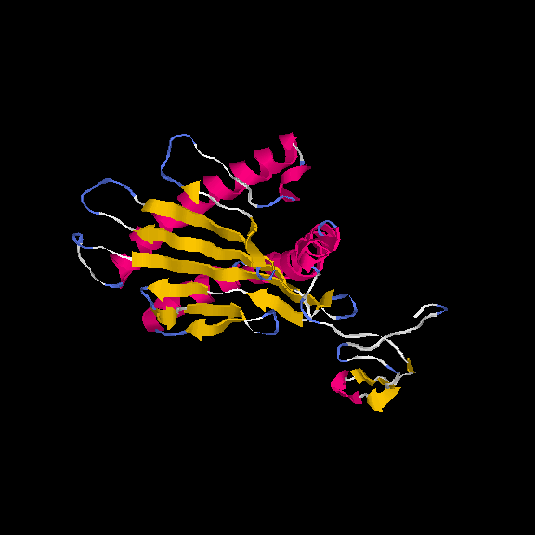 | **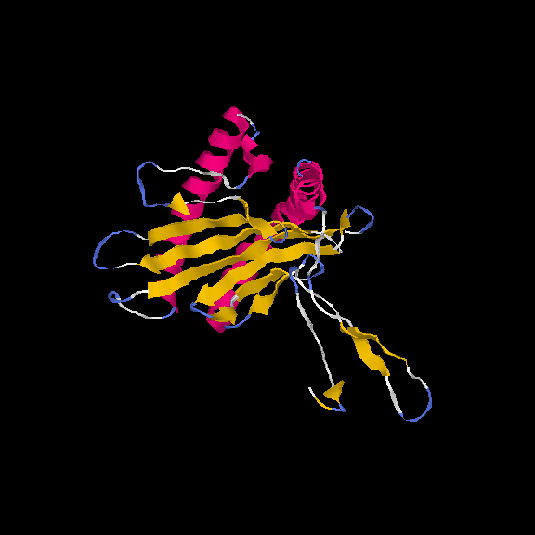** | 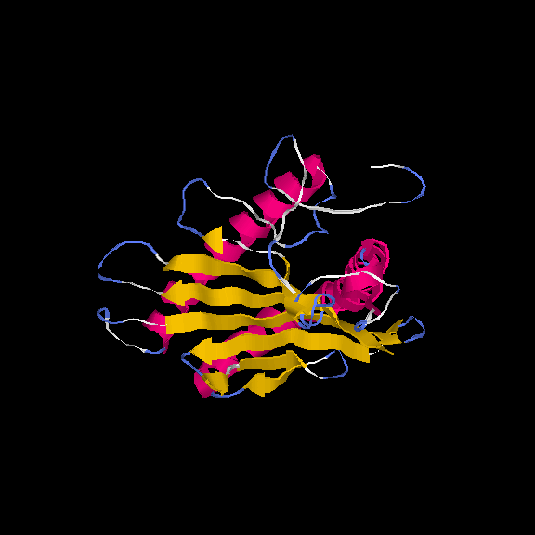 | 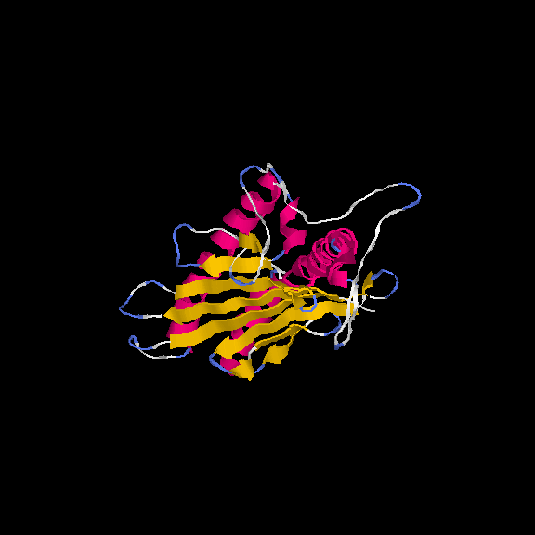 |
| **Model 1 C-score=1.37  Estimated TM-score = 0.90±0.06  Estimated RMSD = 2.9±2.1Å** | Model2 C-score = -4.14 | Model 3 C-score = -5 | Model 4 C-score = -5 | Model 5 C-score = -5 |
| **Isoform 5** | | | | |
| **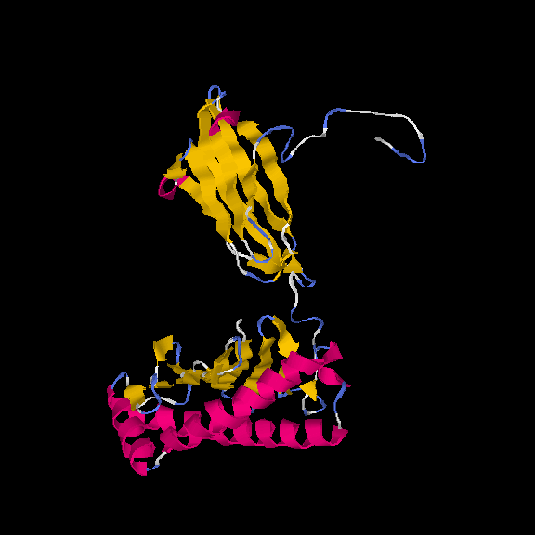** | 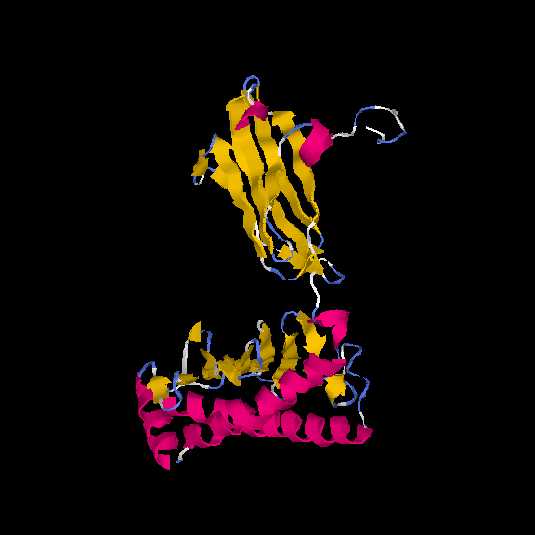 | 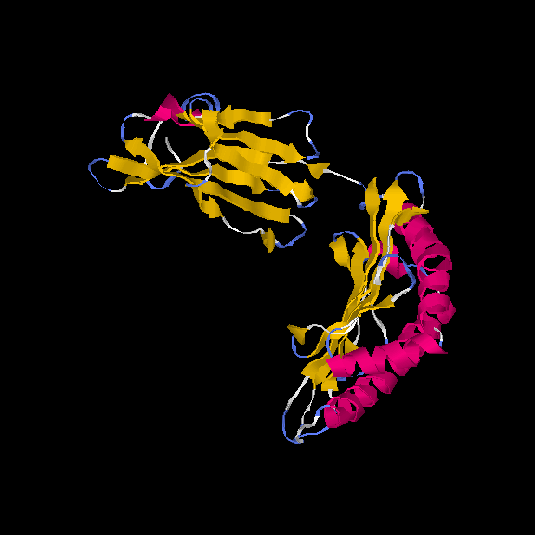 | 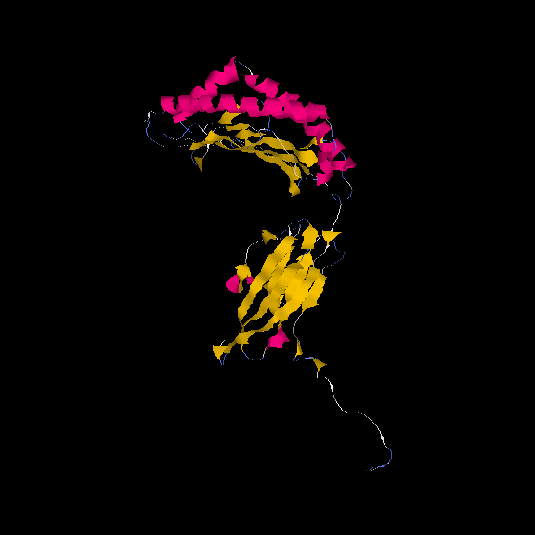 | 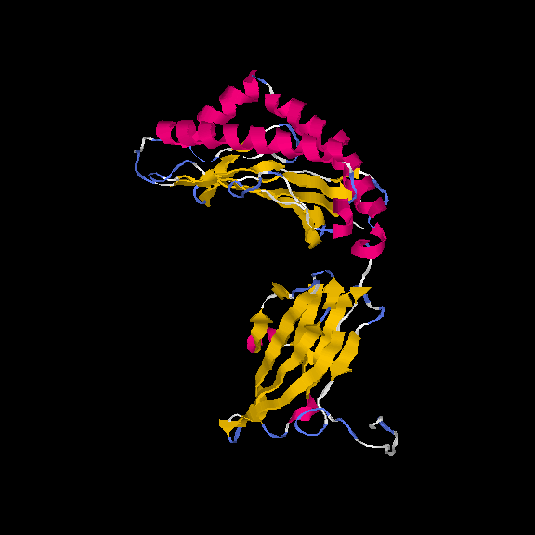 |
| **Model 1 C-score=1.10  Estimated TM-score = 0.86±0.07 Estimated RMSD = 3.9±2.7Å** | Model2 C-score = -1.32 | Model 3 C-score = 0.05 | Model 4 C-score = -5 | Model 5 C-score = -4.53 |
| **Isoform 6** | | | | |
| **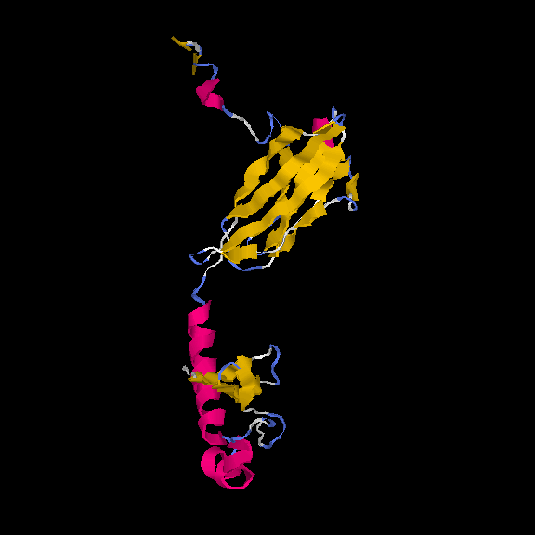** | 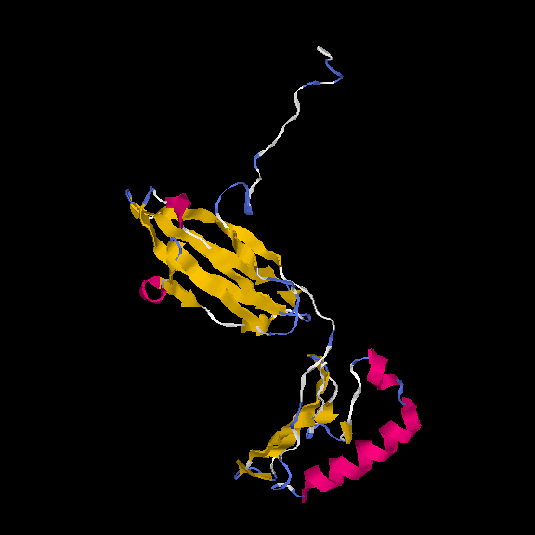 | 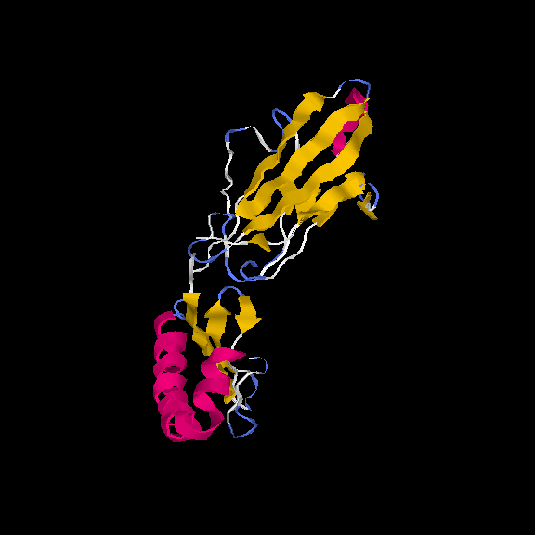 | 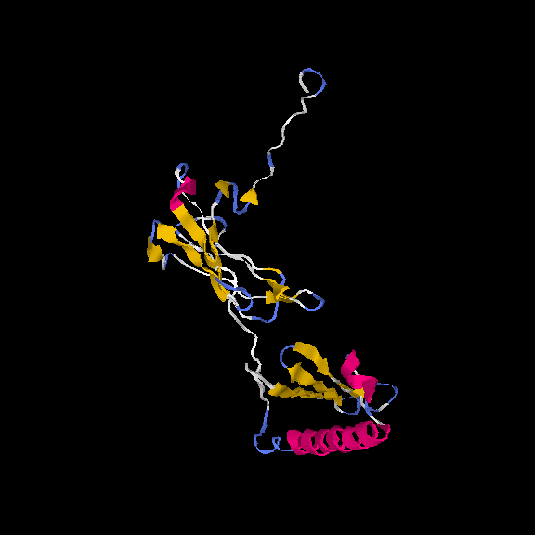 | 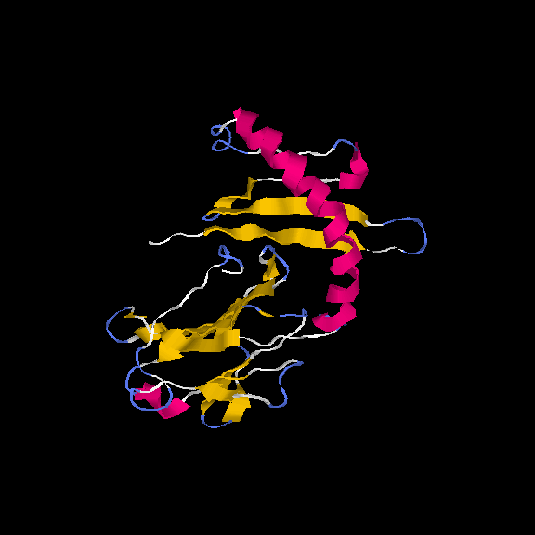 |
| **Model 1 C-score=-1.30  Estimated TM-score = 0.55±0.15 Estimated RMSD = 8.2±4.5Å** | Model2 C-score = -2.43 | Model 3 C-score = -2.18 | Model 4 C-score = -3.17 | Model 5 C-score = -2.95 |
| **Isoform 7** | | | | |
| **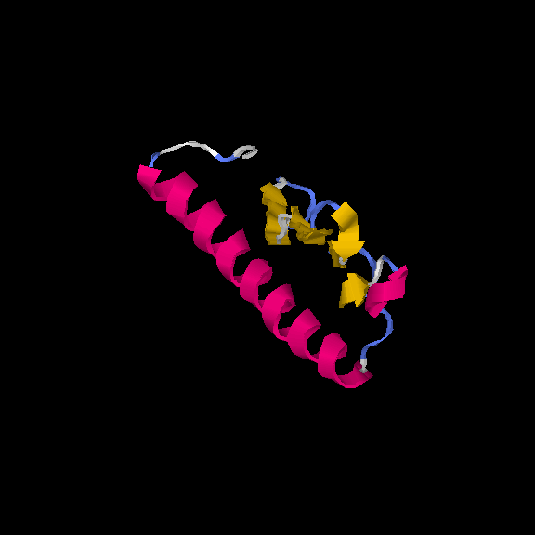** | 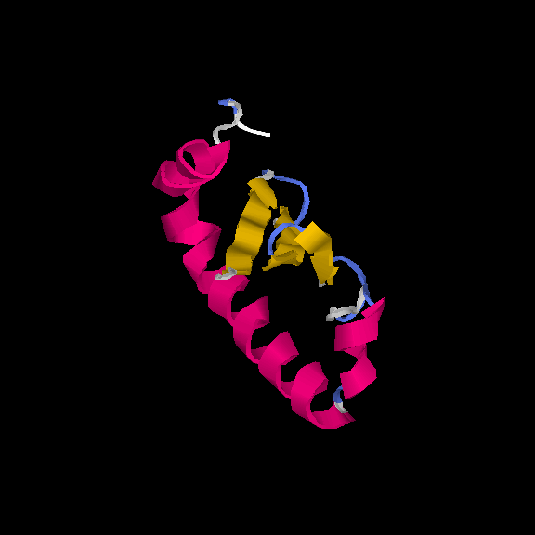 | 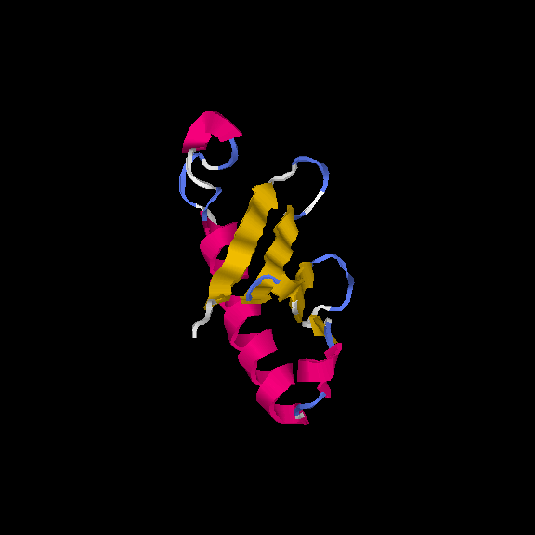 | 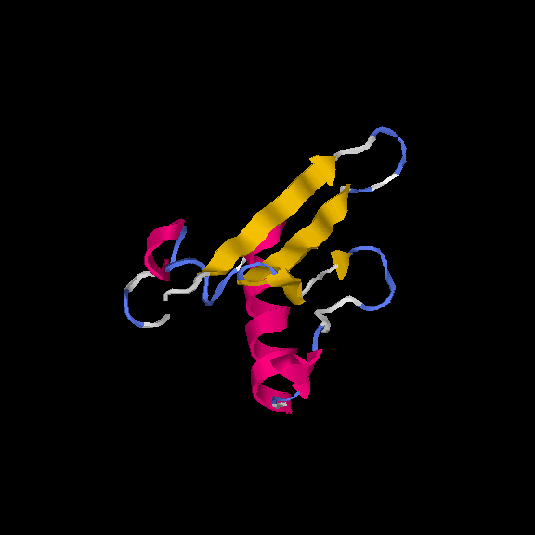 | 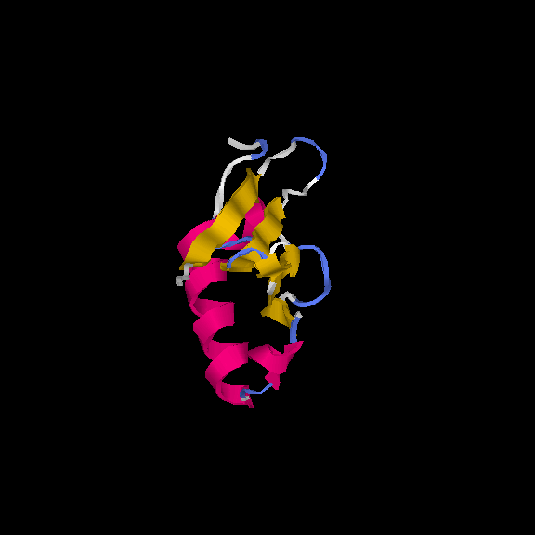 |
| **Model 1 C-score=0.94  Estimated TM-score = 0.84±0.08 Estimated RMSD = 2.0±1.6Å** | Model2 C-score = -4.52 | Model 3 C-score = -5 | Model 4 C-score = -5 | Model 5 C-score = -5 |

The model with the highest confidence score for each isoform is shown in bold font and underline. TM score > 0.5 reveals a model of accurate topology while TM score < 0.17 shows random similarity.
