# Supplementary material for: Predicting the most deleterious missense nsSNPs of the protein isoforms of the human HLA-G gene and in silico evaluation of their structural and functional consequences
Source: BMC Genet. 2020 Aug 31;21:94. doi: 10.1186/s12863-020-00890-y (PMC7457528; doi:10.1186/s12863-020-00890-y)
Supplement: Supplementary file 4 — Table 7. Structural representations of native isoforms of HLA-G predicted with I-TASSE and visualized with UCSF Chimera [file 12863_2020_890_MOESM4_ESM.doc]

**Table 7.** Structural representations of native isoforms of HLA-G predicted with I-TASSE and visualized with UCSF Chimera

| **Structural representations of native transmembrane isoforms of HLA-G predicted with I-TASSE and visualized with UCSF Chimera** | | | | | |
| --- | --- | --- | --- | --- | --- |
| **Isoform 1** | **Isoform 2** | | **Isoform 3** | | **Isoform 4** |
| **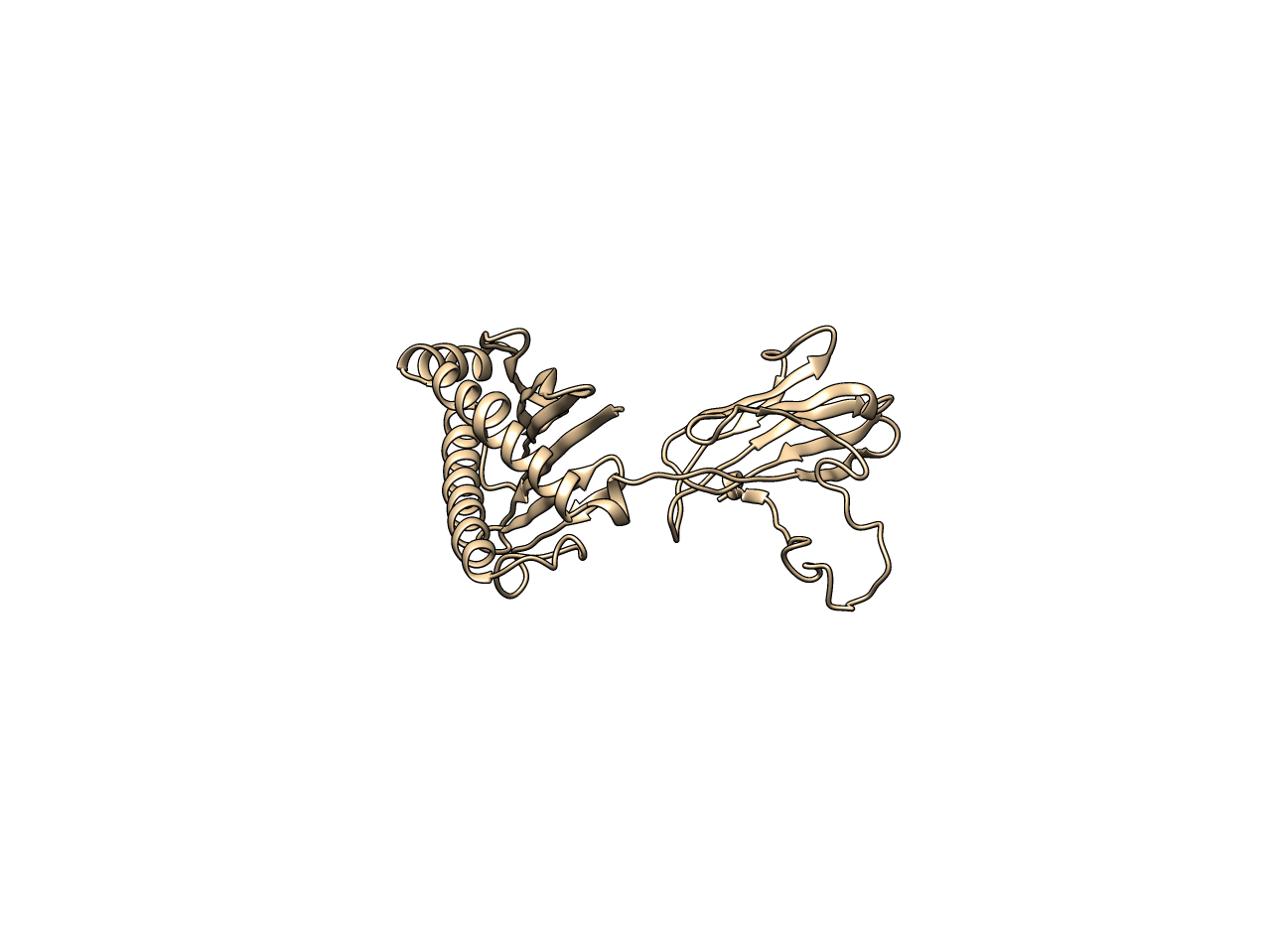** | 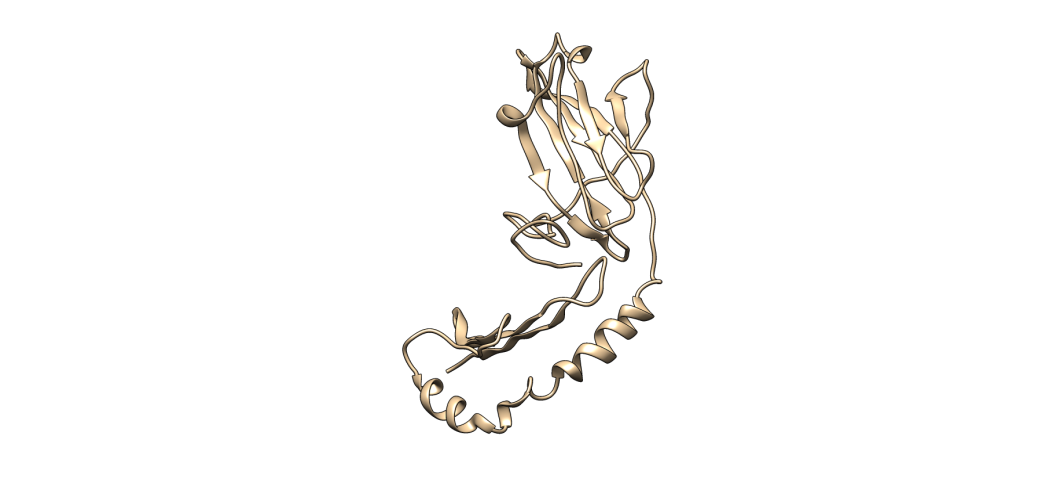 | | 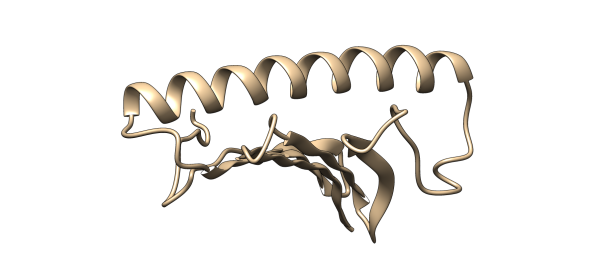 | | 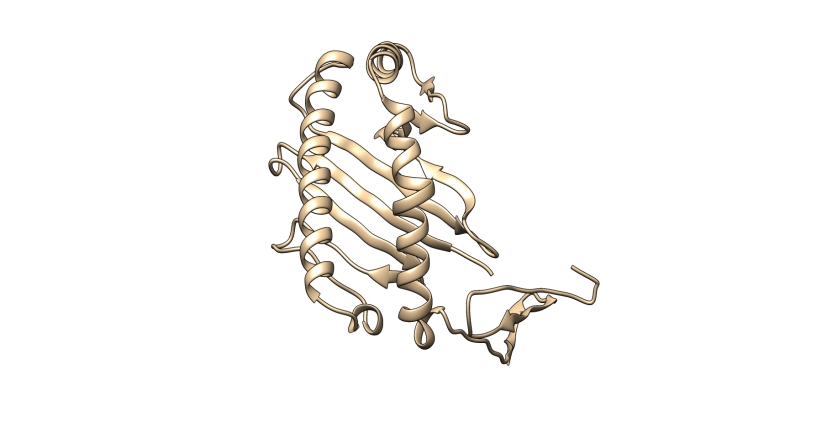 |
| **Structural representations of native soluble isoforms of HLA-G predicted with I-TASSER and visualized with UCSF Chimera** | | | | | |
| **Isoform 5** | | **Isoform 6** | | **Isoform 7** |  |
| **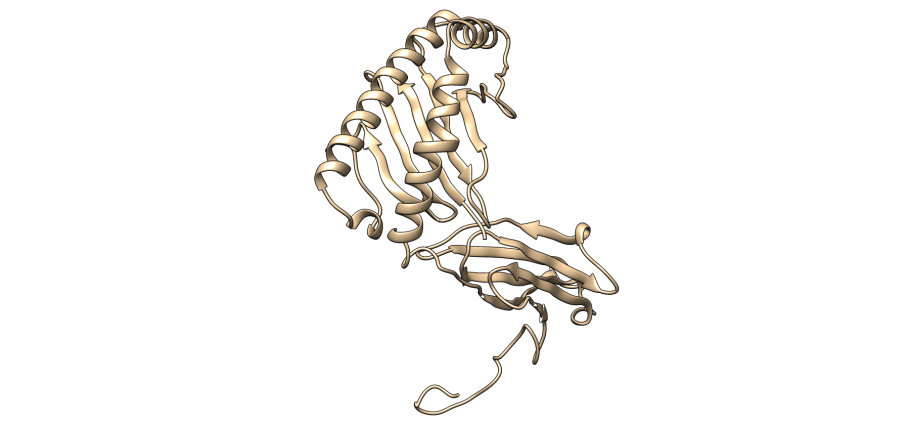** | | 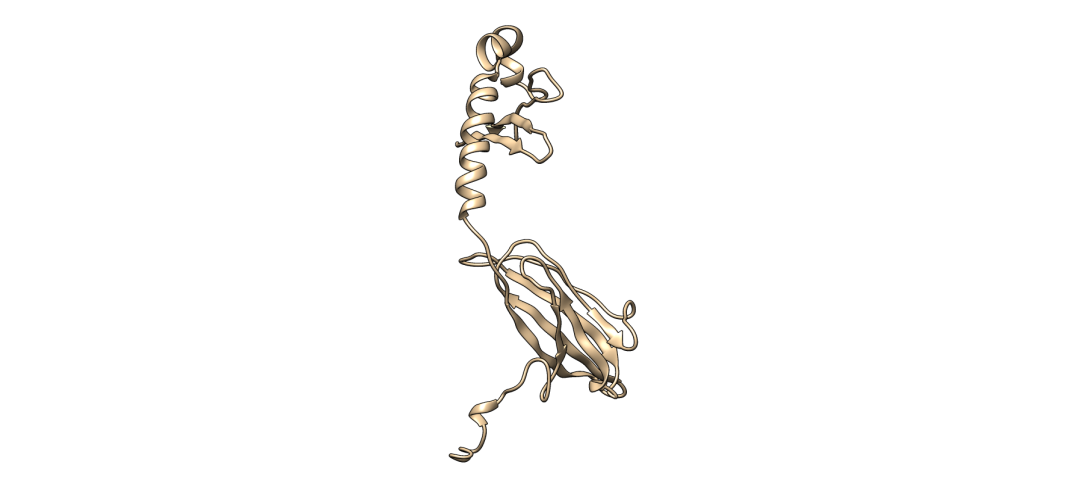 | | 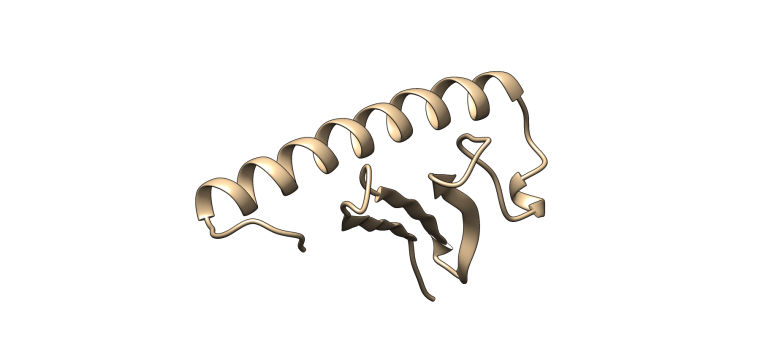 |  |
