# Supplementary material for: Predicting the most deleterious missense nsSNPs of the protein isoforms of the human HLA-G gene and in silico evaluation of their structural and functional consequences
Source: BMC Genet. 2020 Aug 31;21:94. doi: 10.1186/s12863-020-00890-y (PMC7457528; doi:10.1186/s12863-020-00890-y)
Supplement: Supplementary file 5 — Table 8. Graphical representations of amino acid changes due to the most deleterious SNPs in isoform 1 [file 12863_2020_890_MOESM5_ESM.doc]

**Table 8.** Graphical representations of amino acid changes due to the most deleterious SNPs in isoform 1

|  | **SNP ID: rs555347515** | **SNP ID: rs572025435** | | **SNP ID: rs1475659109** | |
| --- | --- | --- | --- | --- | --- |
| **protein position 29 changed from Methionine (green (upper image)) to  Lysine (red (bottom image))** | 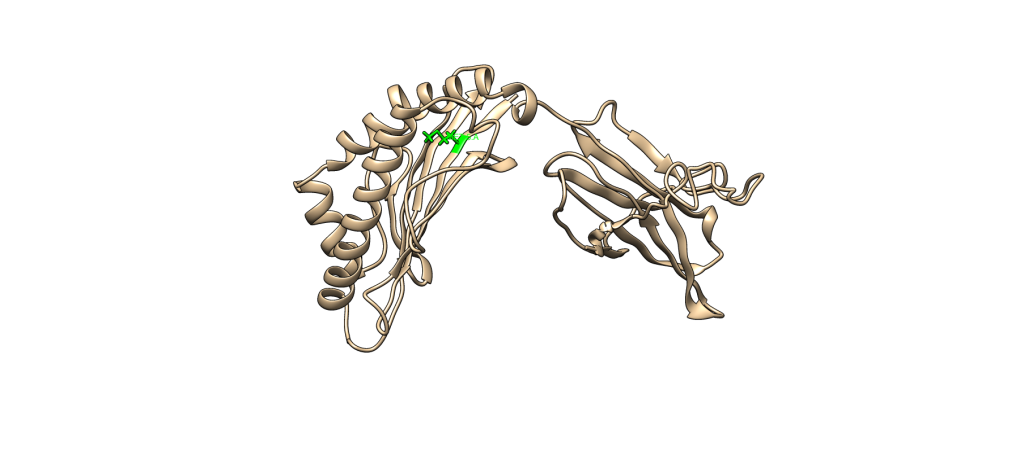 | **protein position 30 changed from Arginine (green (upper image)) to  Serine (red (bottom image))** | 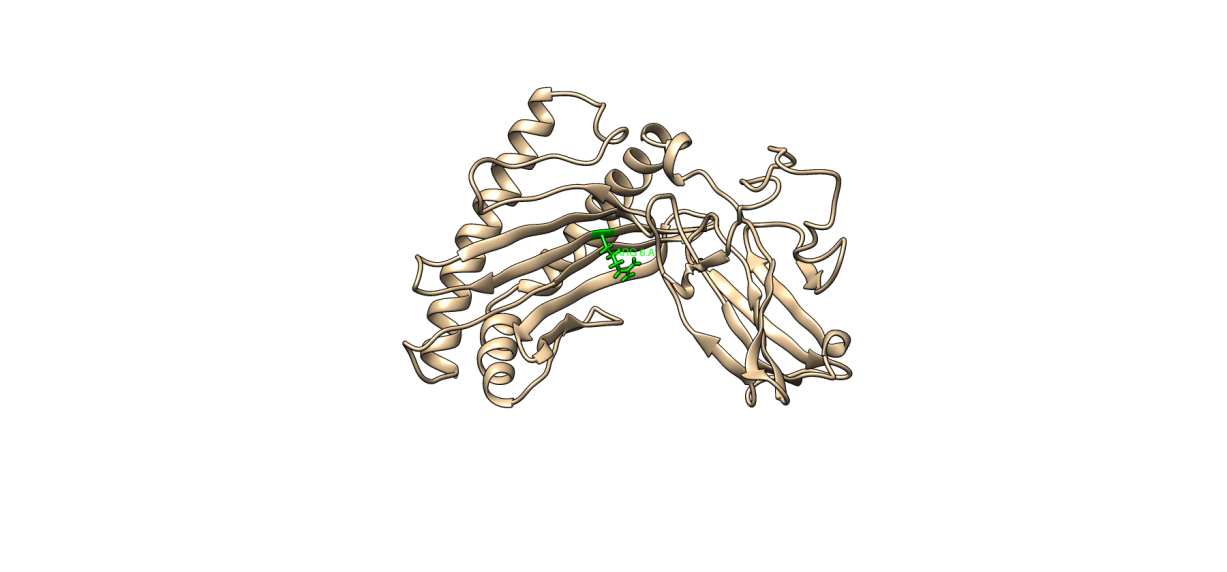 | **protein position 39 changed from Proline (green (upper image)) to  Leucine (red (bottom image))** | 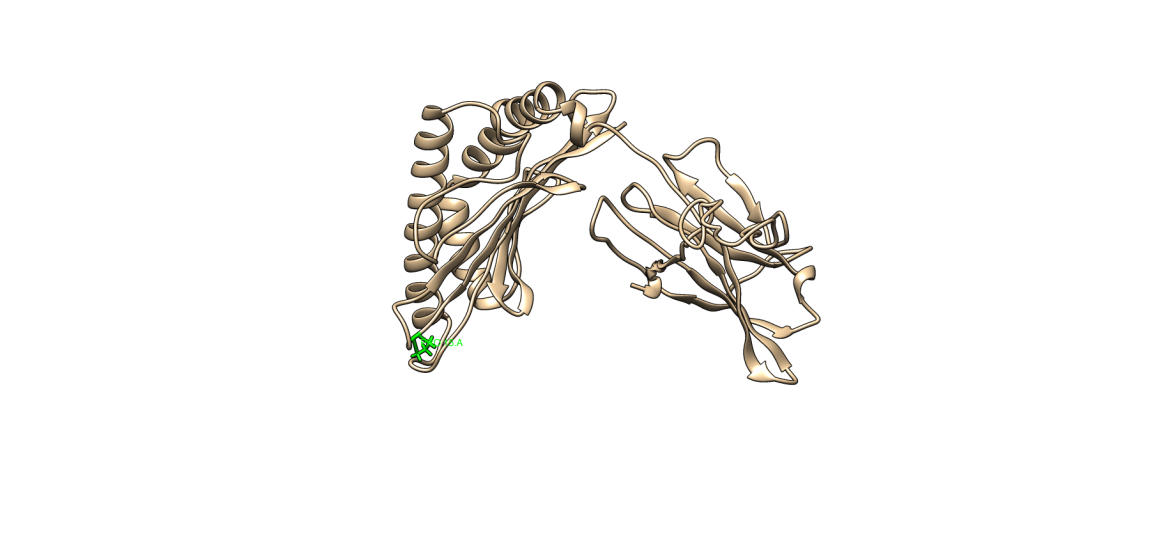 |
|  | 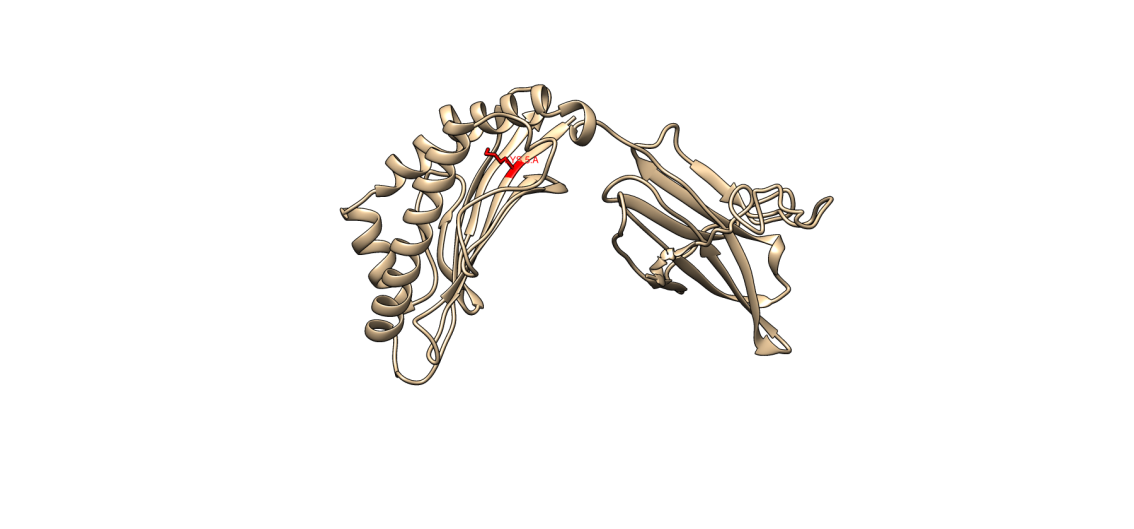 |  | 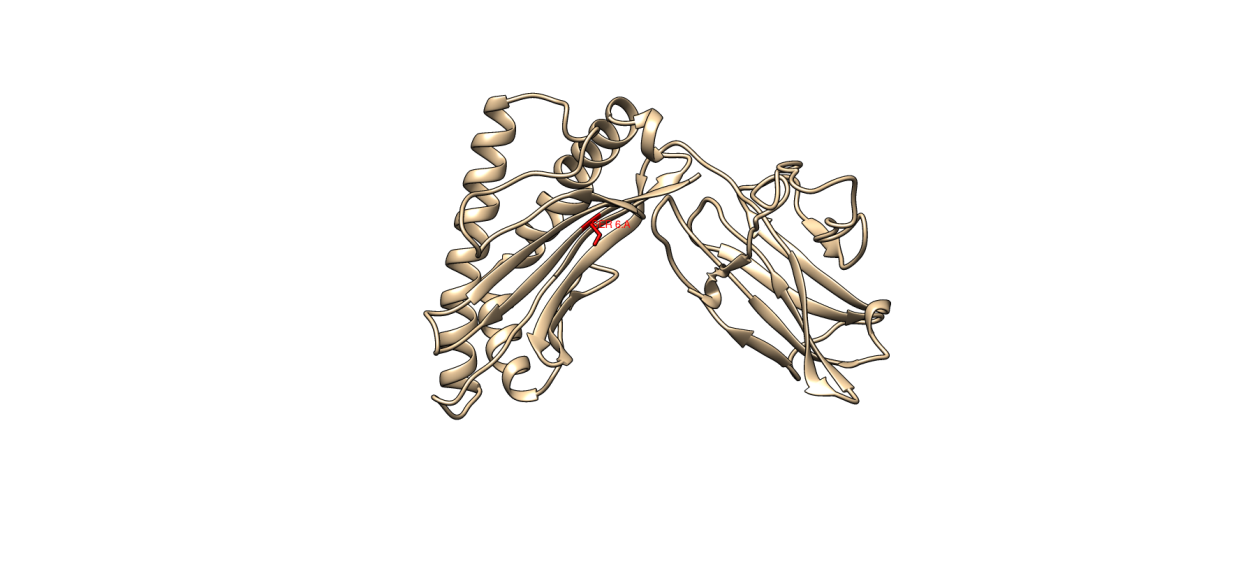 |  | 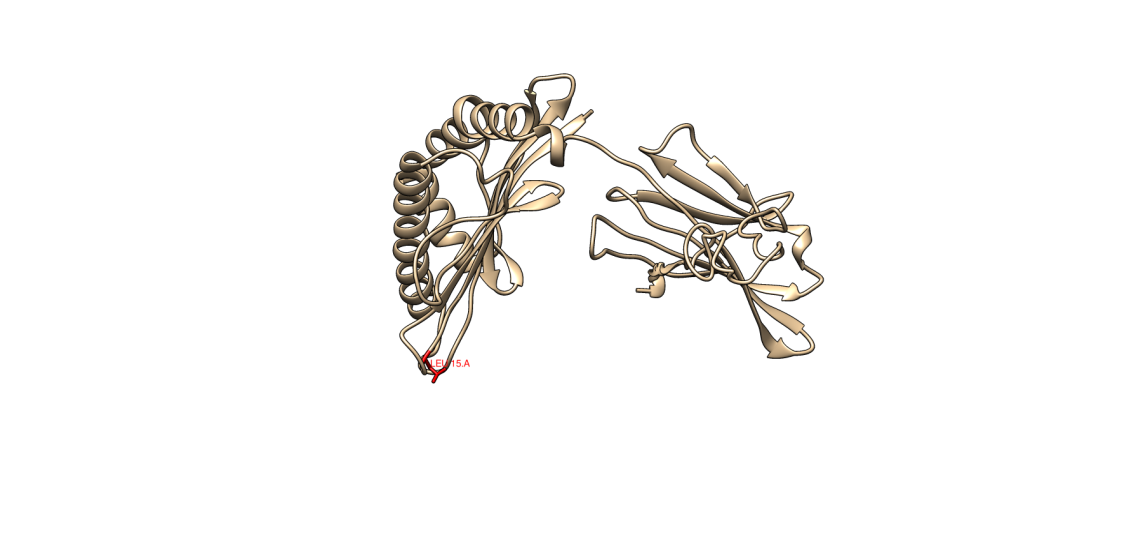 |
| **protein position 51 changed from Tyrosine (green (upper image)) to** [**Cysteine**](https://en.wikipedia.org/wiki/Cysteine) **(red (bottom image))** | **SNP ID: rs1390270595** | **SNP ID: rs763201540** | | **SNP ID: rs763201540** | |
|  | 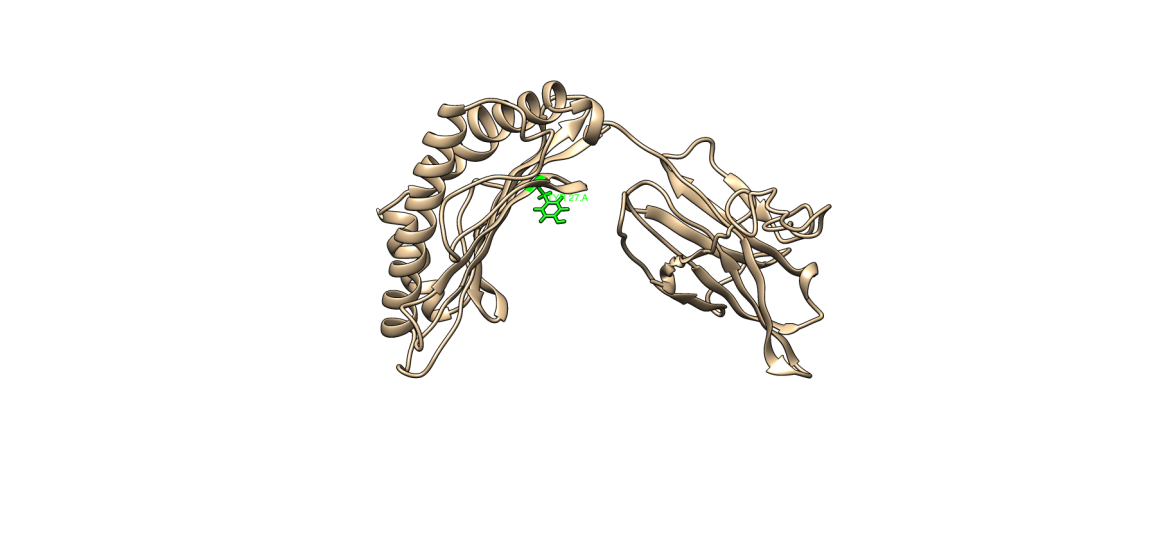 | **protein position 53 changed from Aspartic acid (green (upper image)) to  Asparagine (red (bottom image))** | 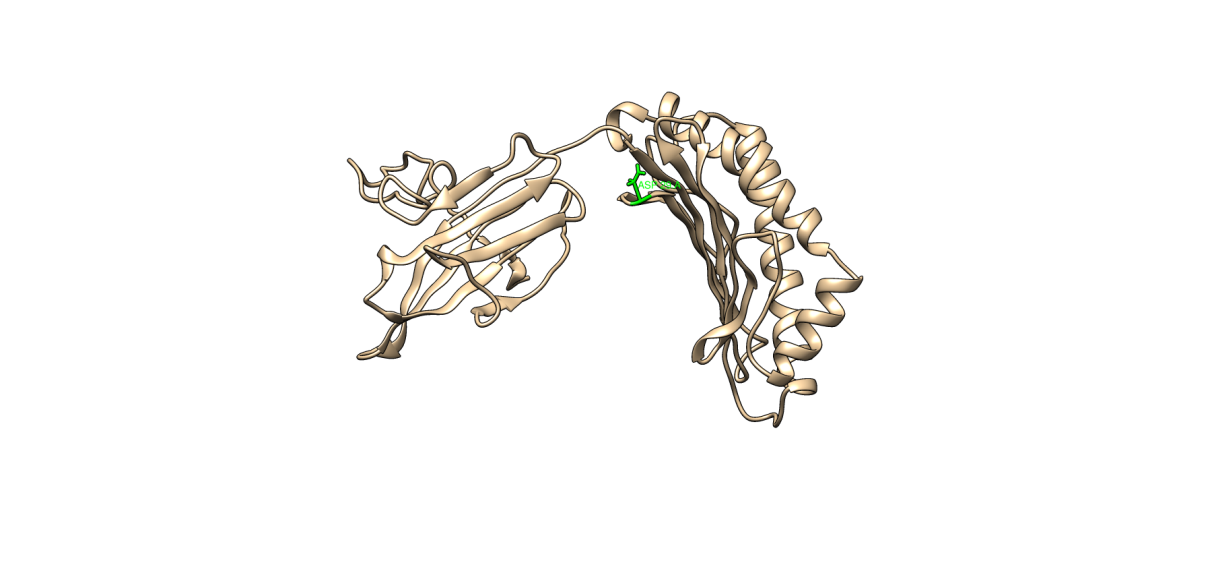 | **protein position 53 changed from Aspartic acid (green (upper image)) to  Tyrosine (red (bottom image))** | 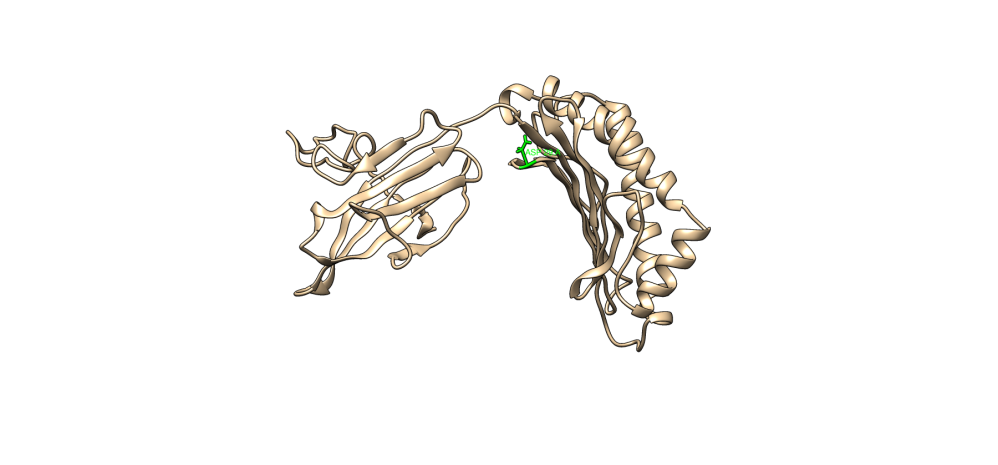 |
|  | 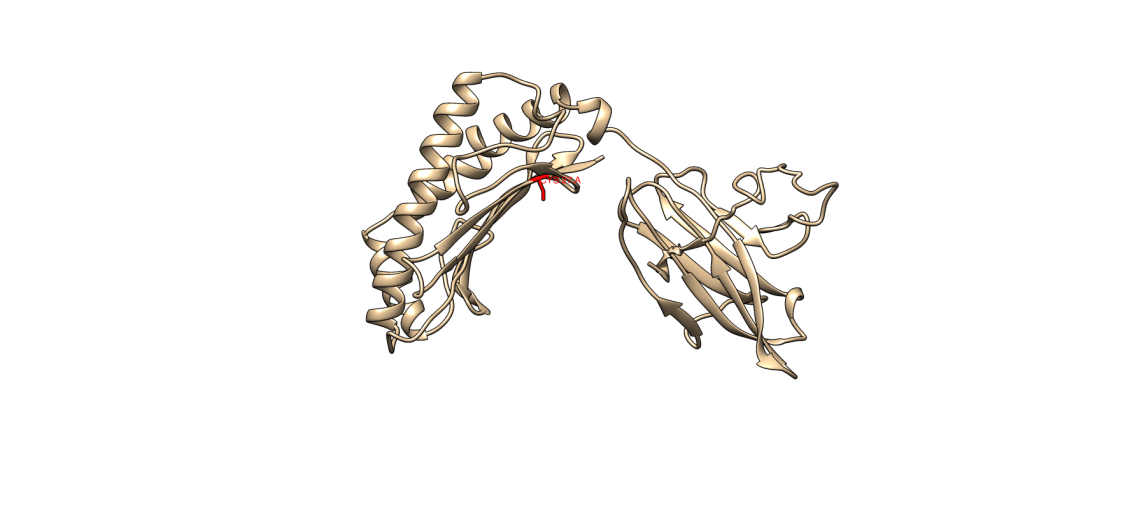 |  | 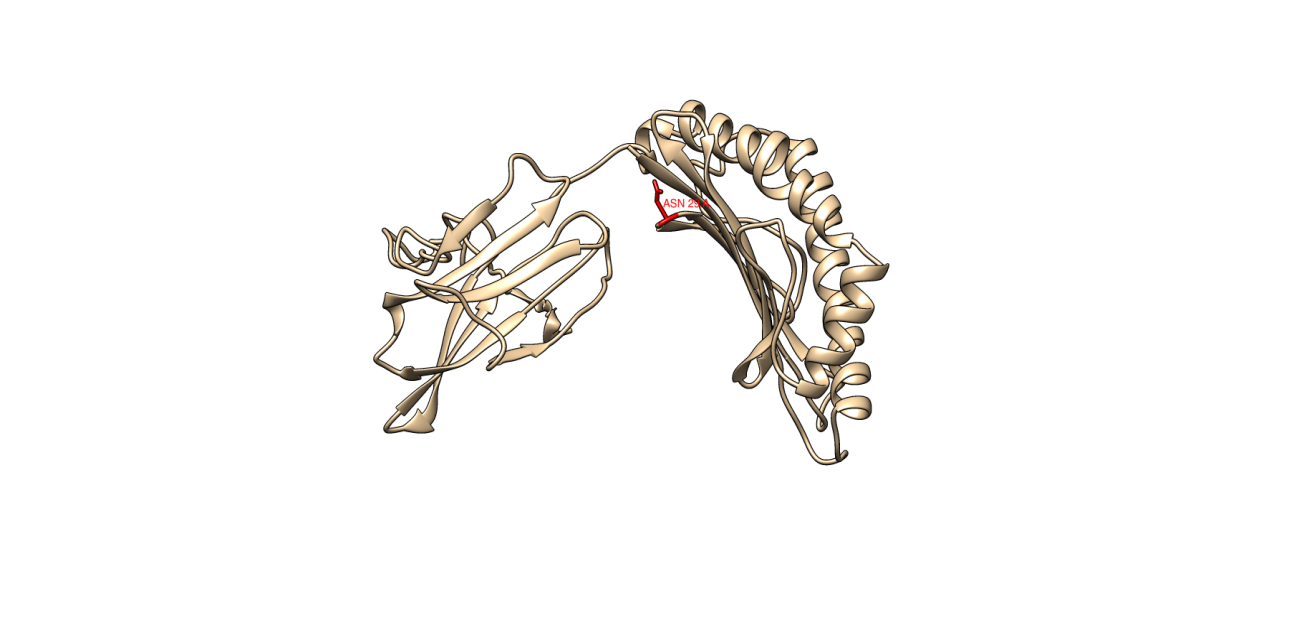 |  | 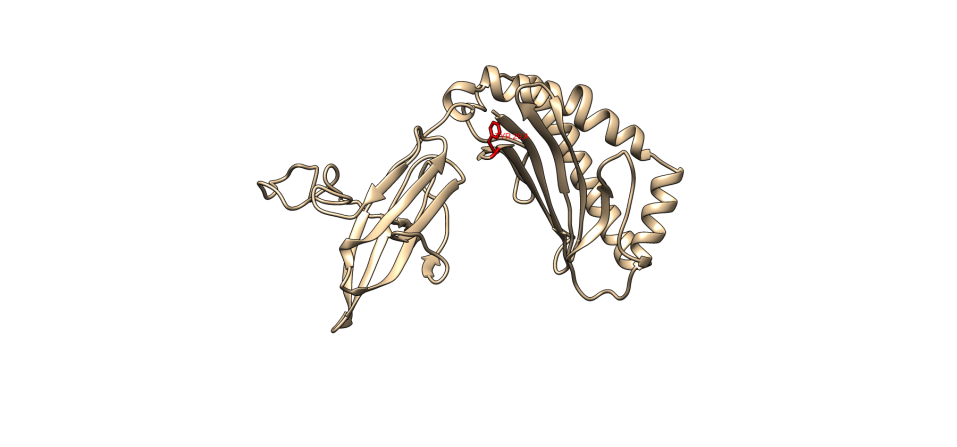 |
| **protein position 54 changed from Aspartic acid (green (upper image)) to  Valine (red (bottom image))** | **SNP ID: rs1414848134** | **SNP ID: rs1260086927** | | **SNP ID: rs770412396** | |
|  | 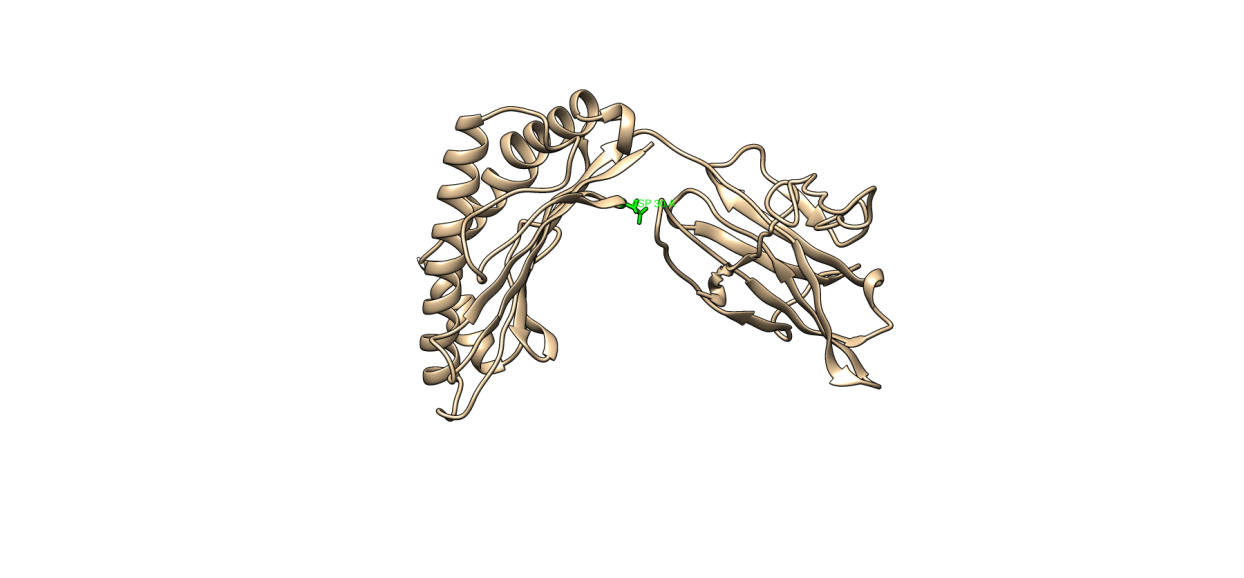 | **protein position 96 changed from Glutamine (green (upper image)) to  Proline (red (bottom image))** | 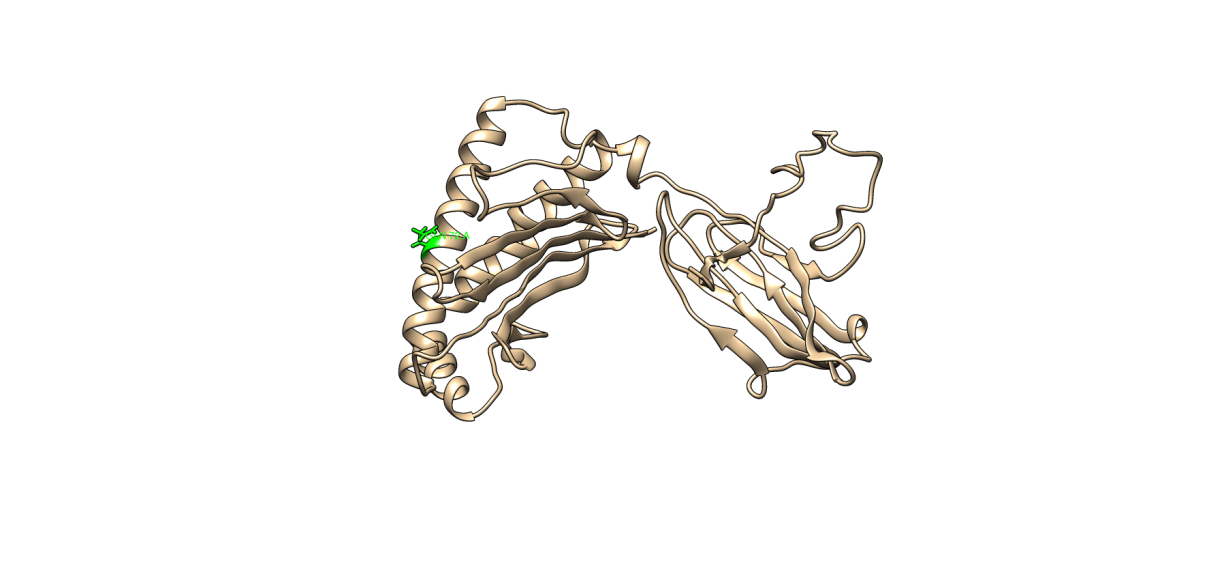 | **protein position 102 changed from Leucine (green (upper image)) to  Proline (red (bottom image))** | 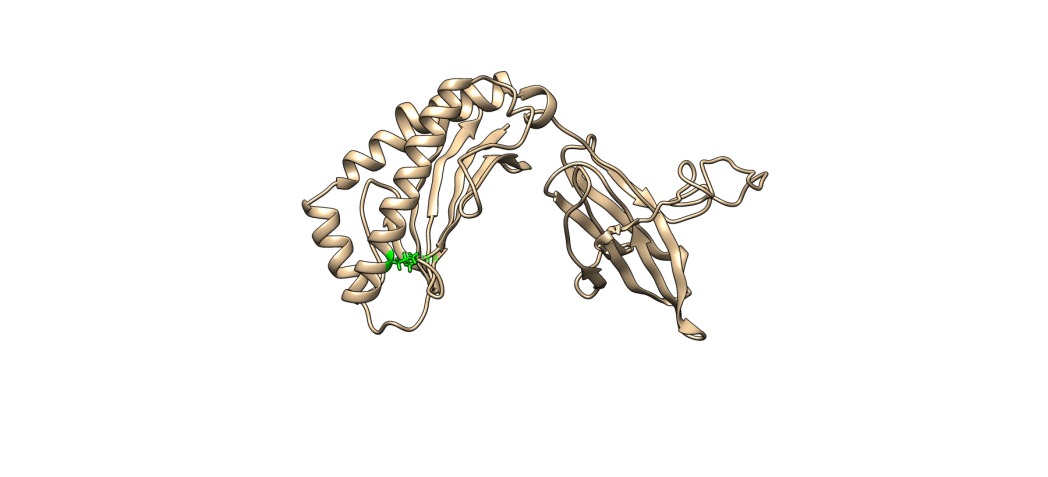 |
|  | 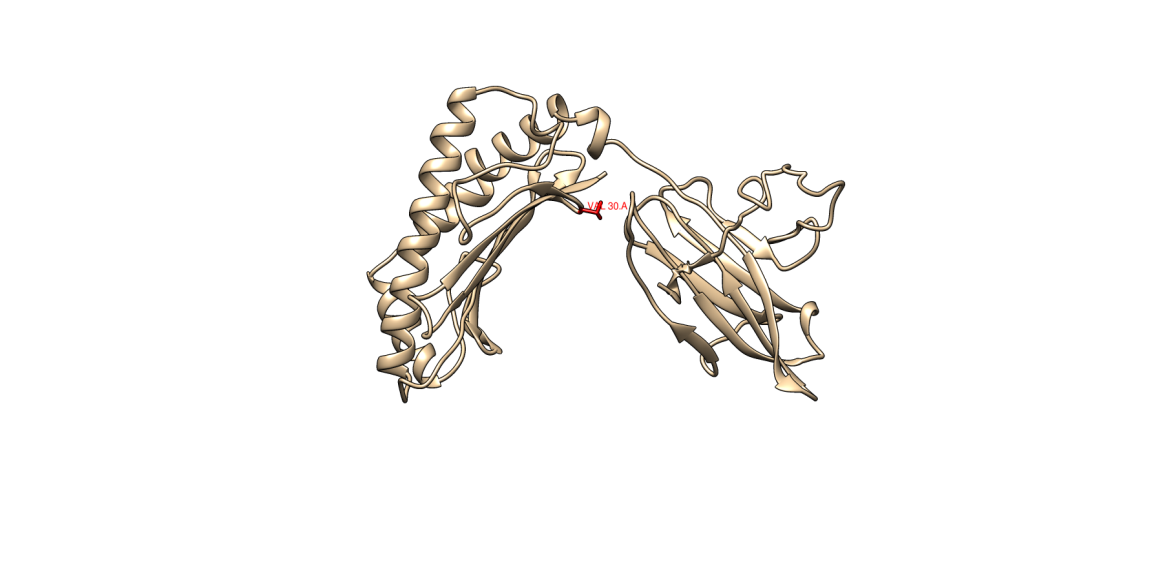 |  | 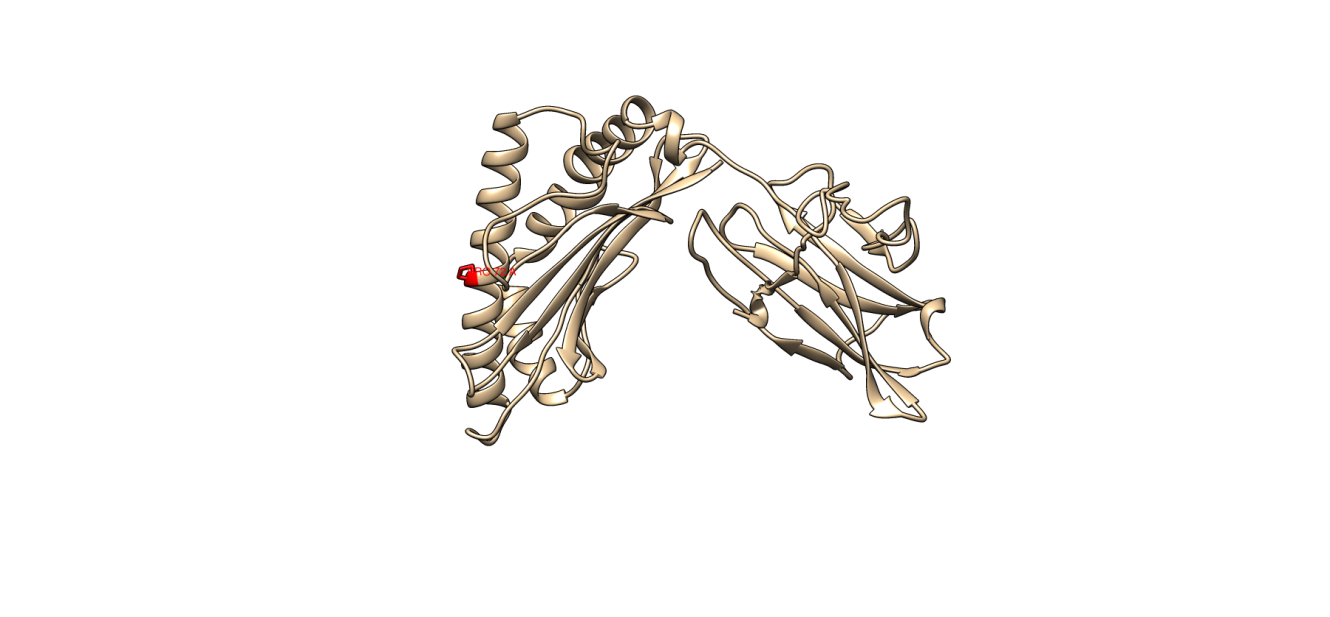 |  | 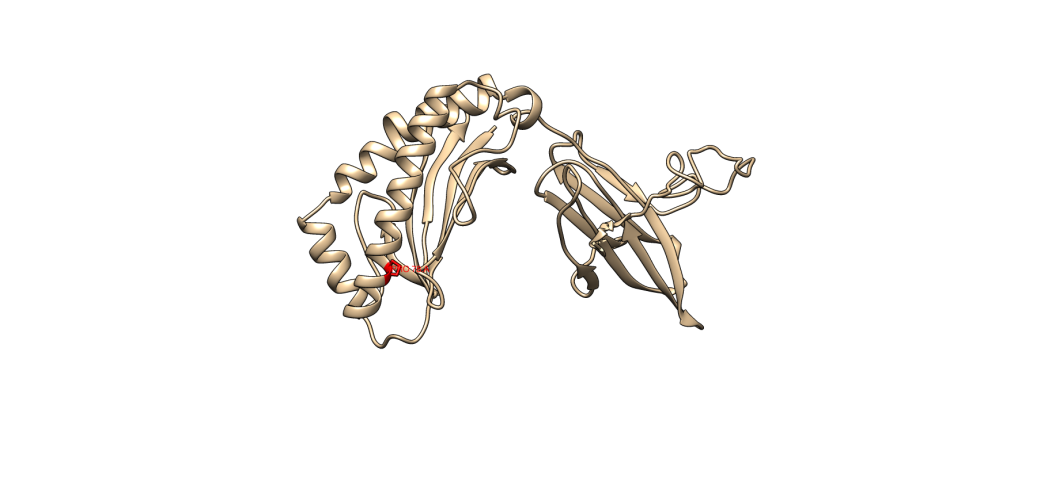 |
| **protein position 105 changed from Leucine (green (upper image)) to  Glutamine (red (bottom image))** | **SNP ID: rs1161818149** | **SNP ID: rs1161818149** | | **SNP ID: rs17851921** | |
|  | 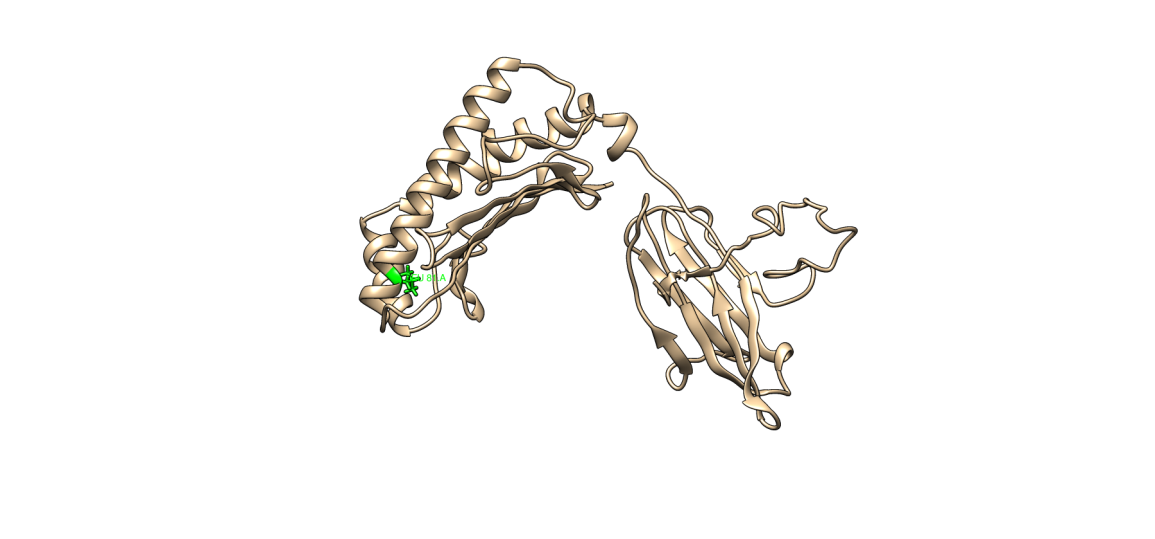 | **protein position 105 changed from Leucine (green (upper image)) to  Proline (red (bottom image))** | 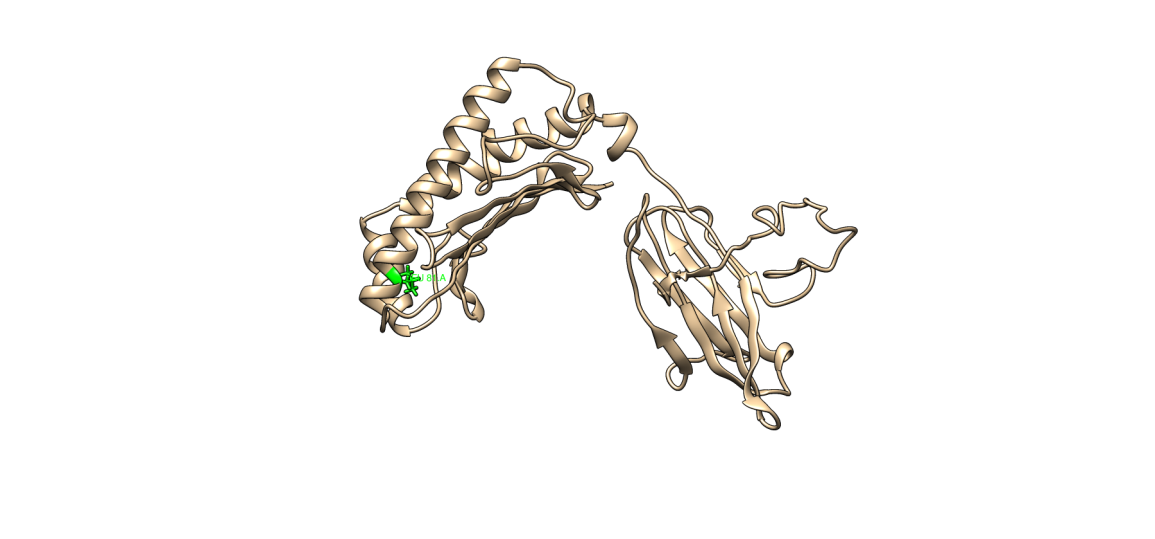 | **protein position 117 changed from Histidine (green (upper image)) to  Proline (red (bottom image))** | 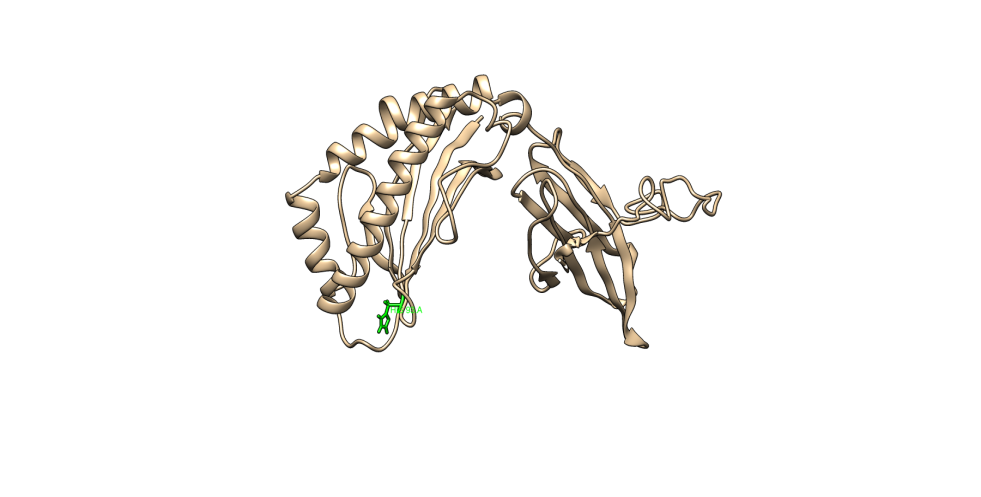 |
|  | 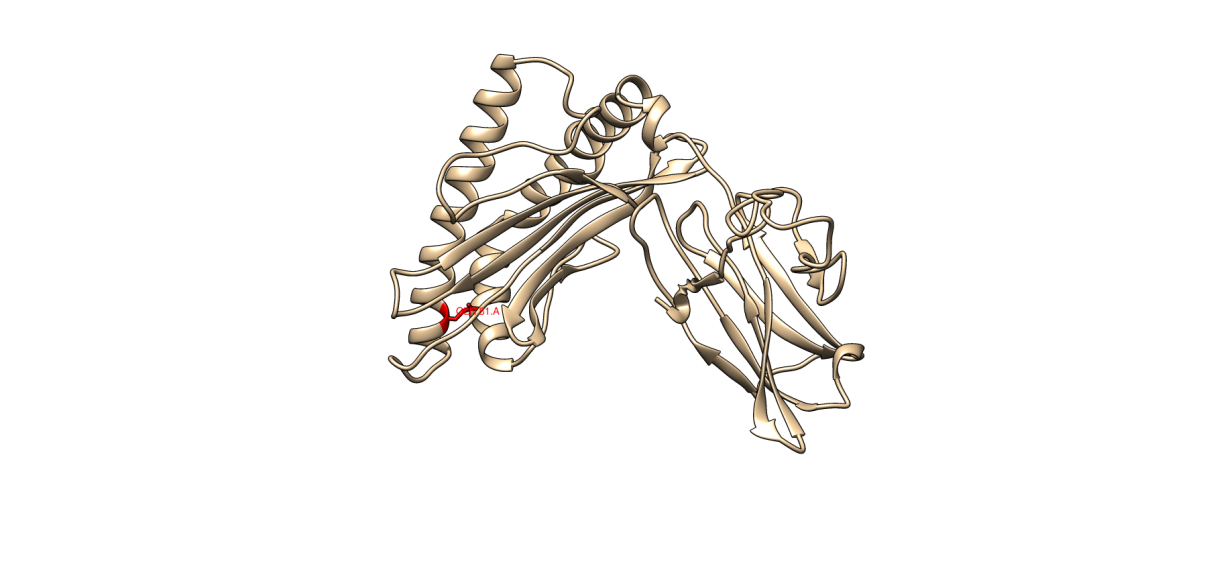 |  | 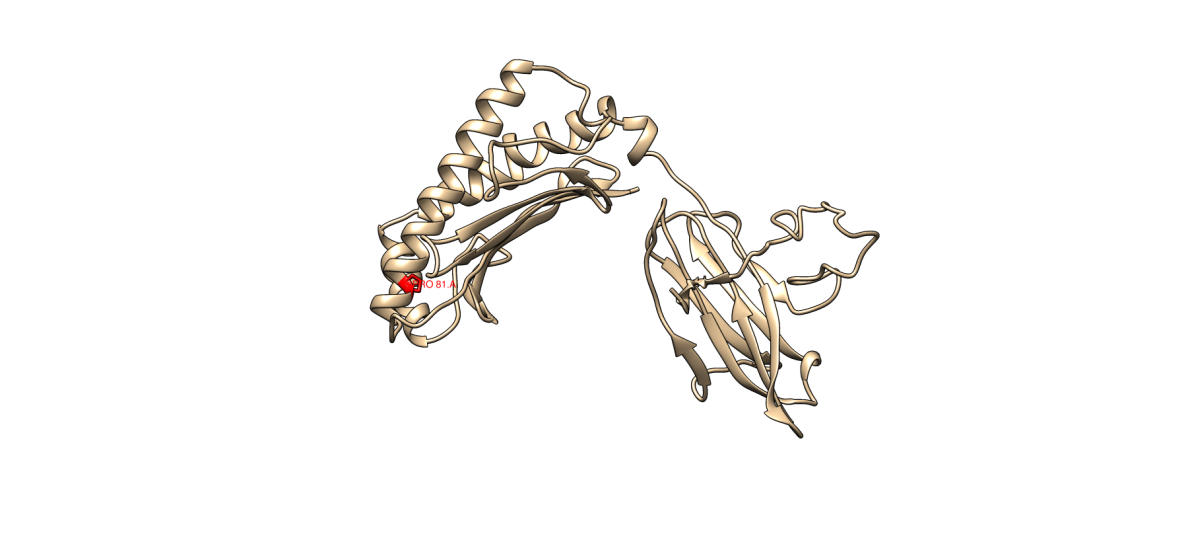 |  | 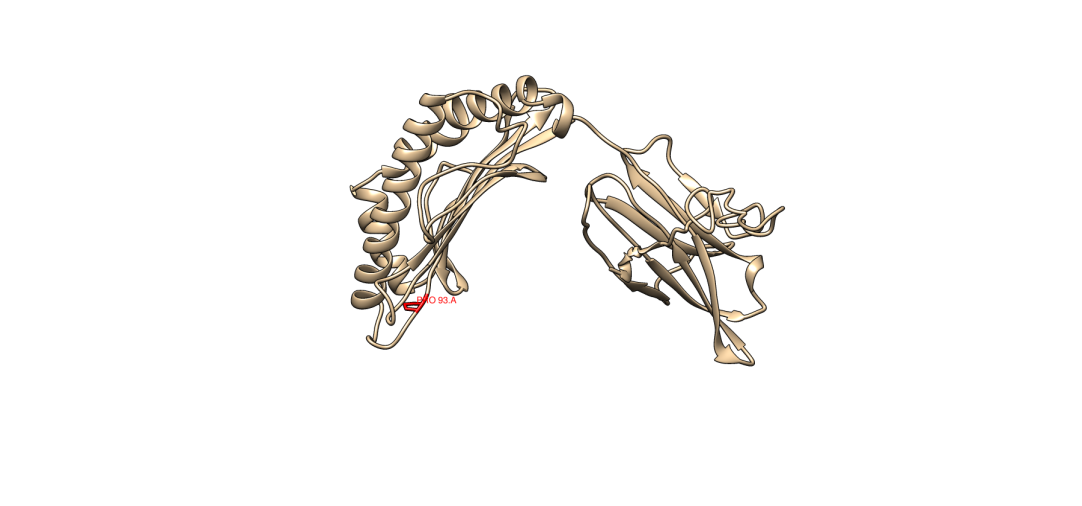 |
| **protein position 117 changed from Histidine (green (upper image)) to  Leucine (red (bottom image))** | **SNP ID: rs17851921** | **SNP ID: rs565858069** | | **SNP ID: rs749006959** | |
|  | 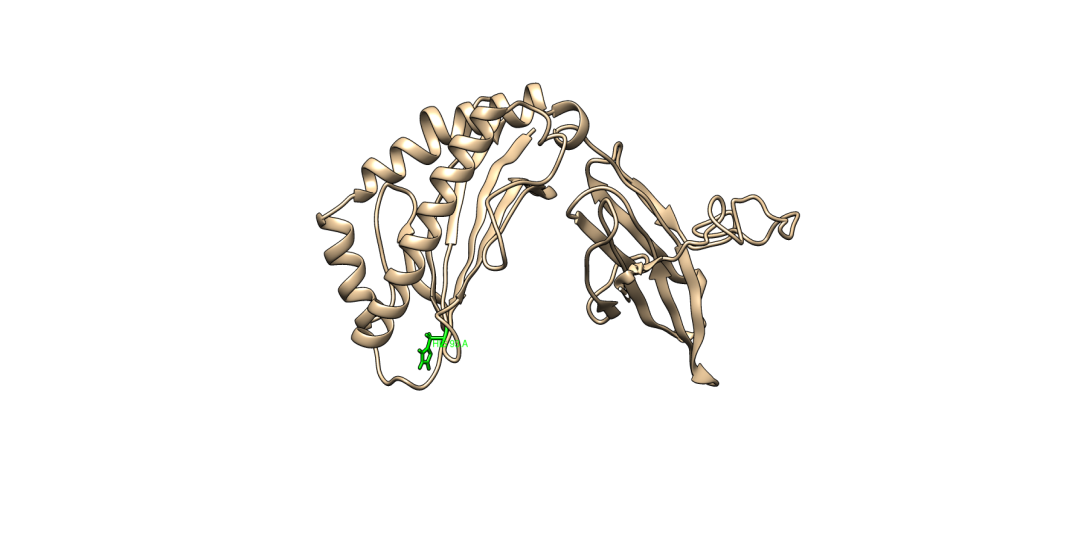 | **protein position 130 changed from Aspartic acid (green (upper image))  to Histidine (red (bottom image))** | 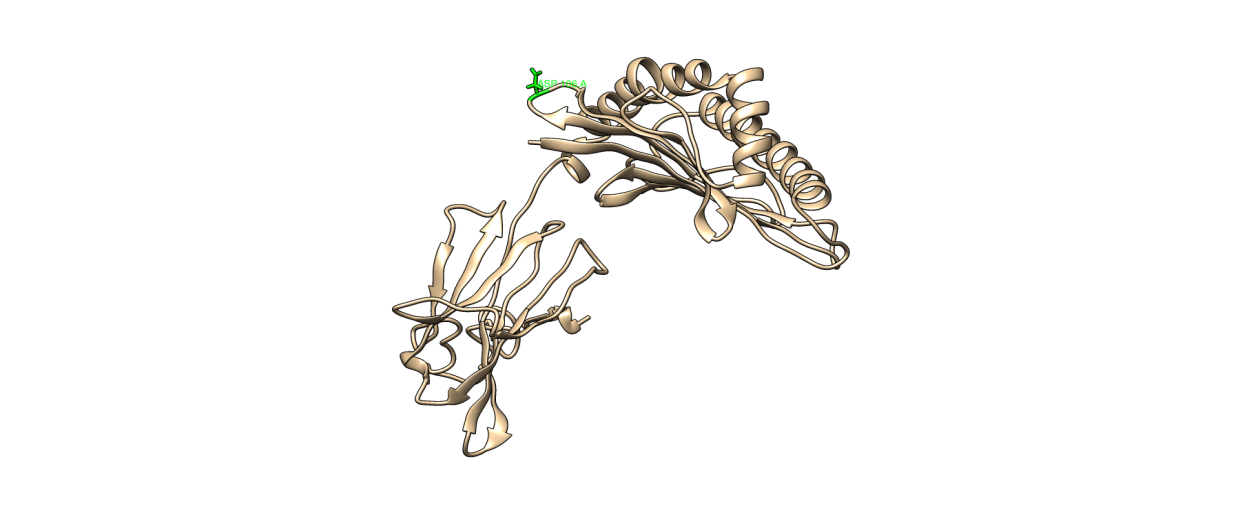 | **protein position 142 changed from Tyrosine (green (upper image)) to  Cysteine (red (bottom image))** | 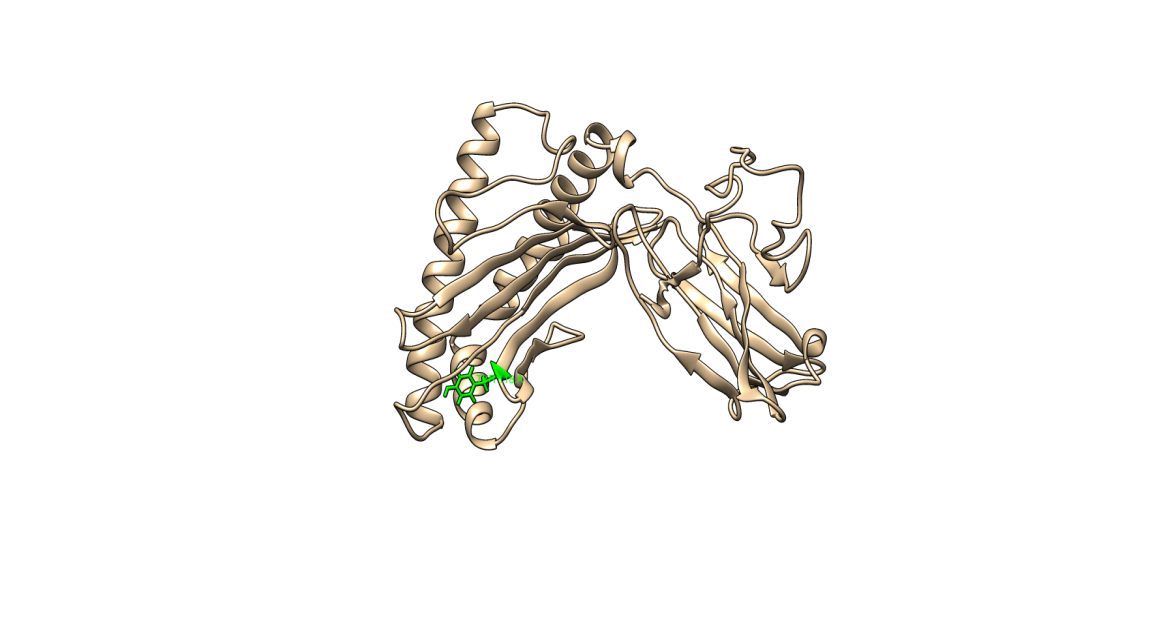 |
|  | 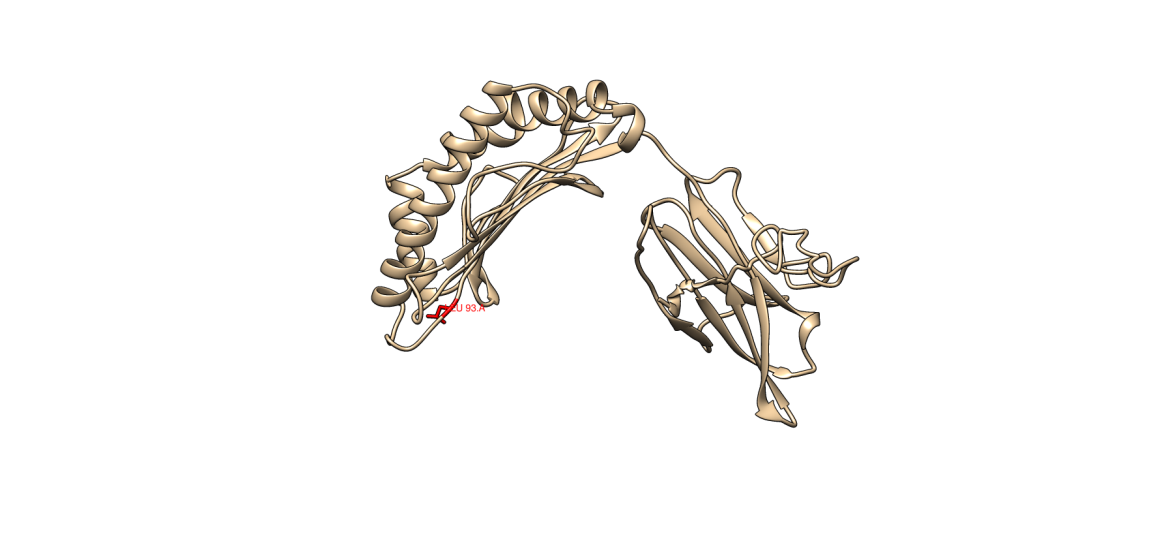 |  | 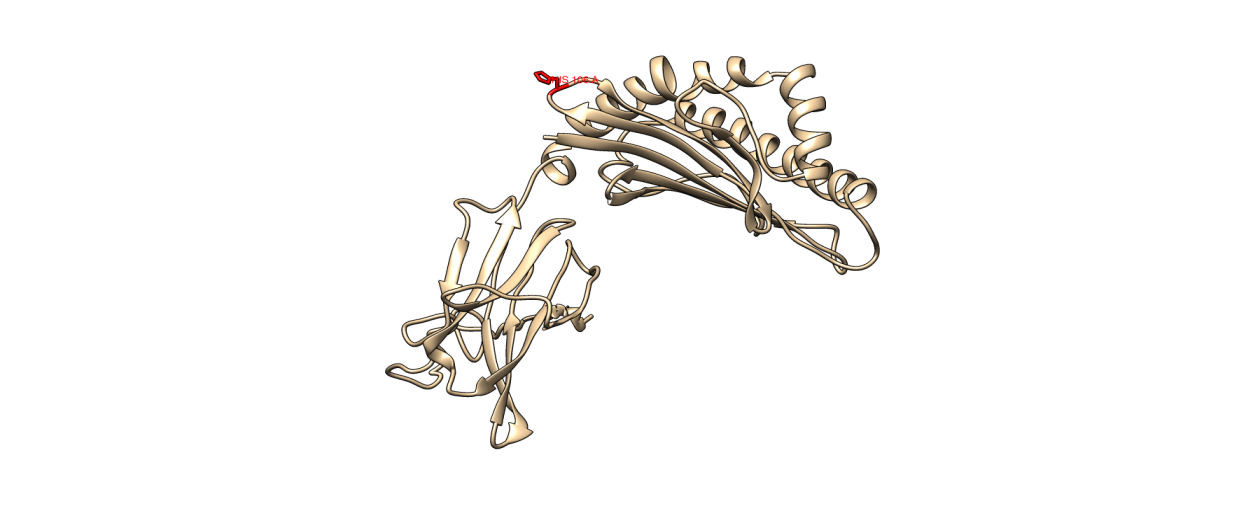 |  | 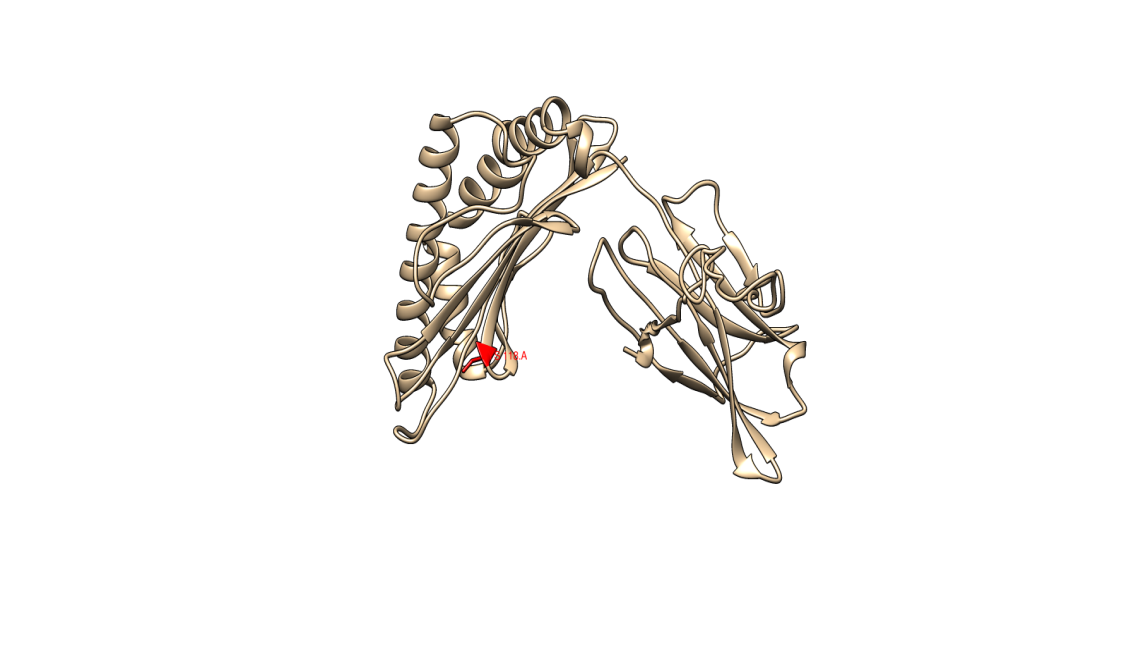 |
| **protein position 142 changed from Tyrosine (green (upper image)) to  Histidine (red (bottom image))** | **SNP ID: rs772834879** | **SNP ID: rs1317292772** | | **SNP ID: rs1317292772** | |
|  | 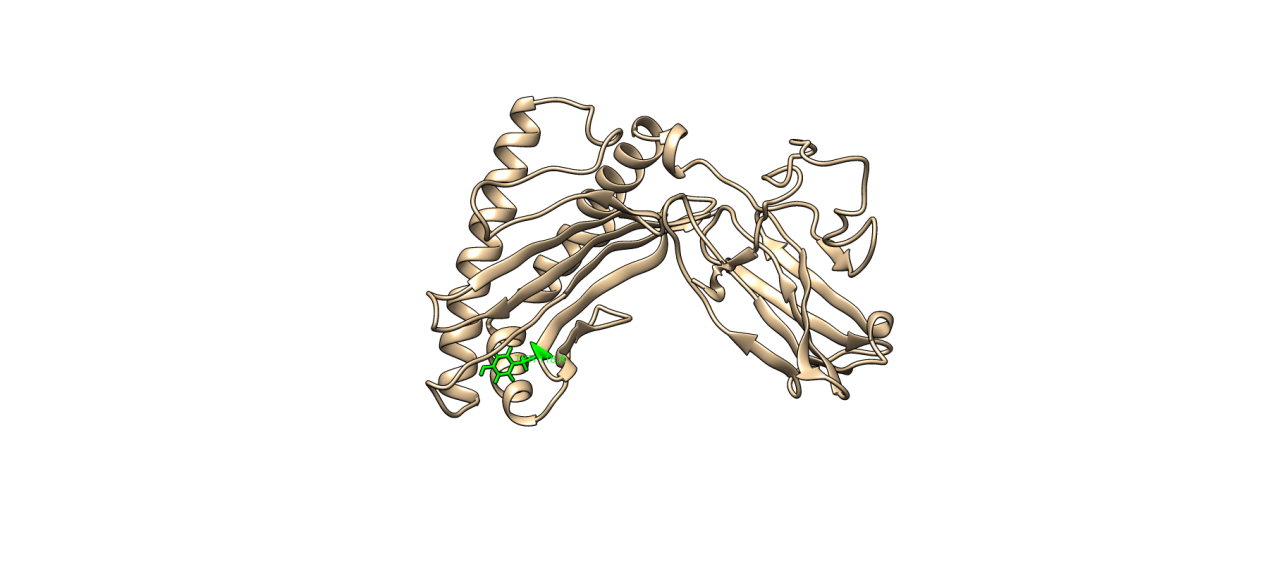 | **protein position 143 changed from Aspartic acid (green (upper image)) to  Asparagine (red (bottom image))** | 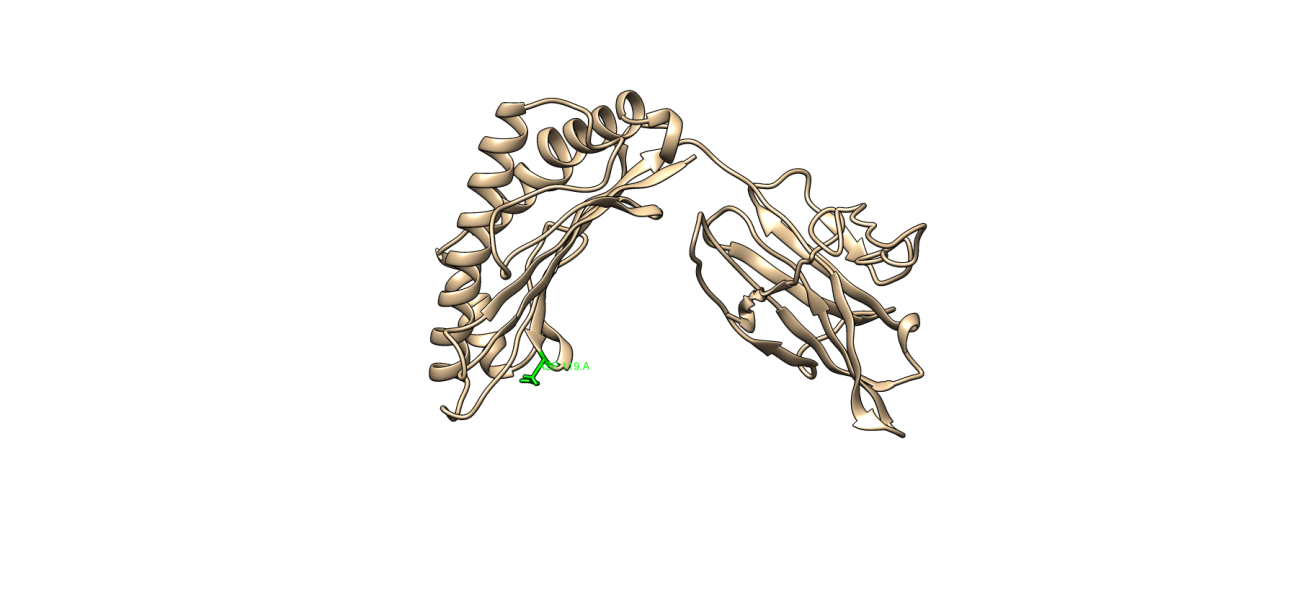 | **protein position 143 changed from Aspartic acid (green (upper image)) to  Histidine (red (bottom image))** | 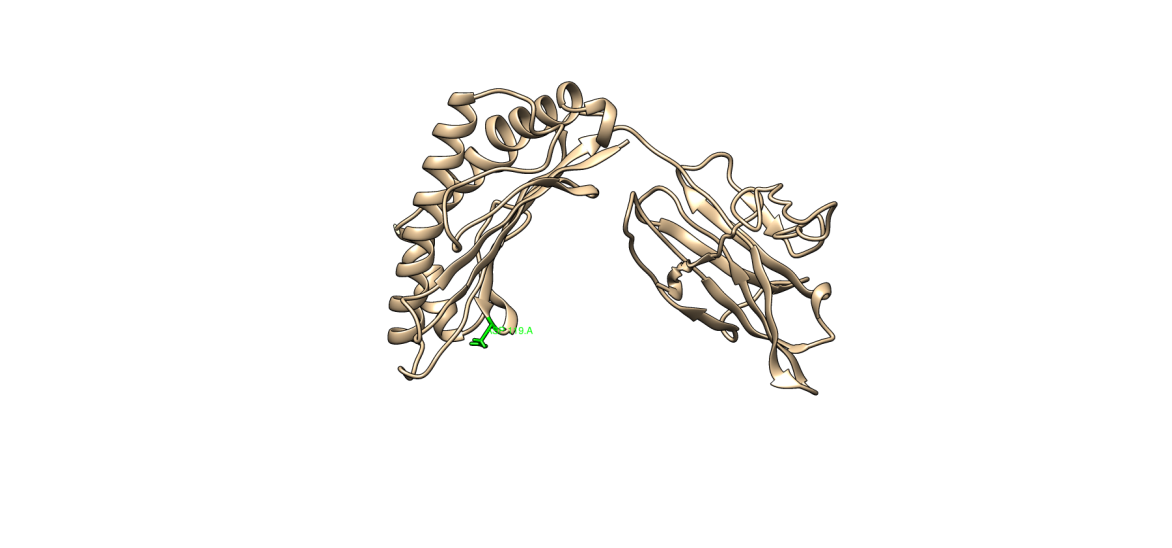 |
|  | 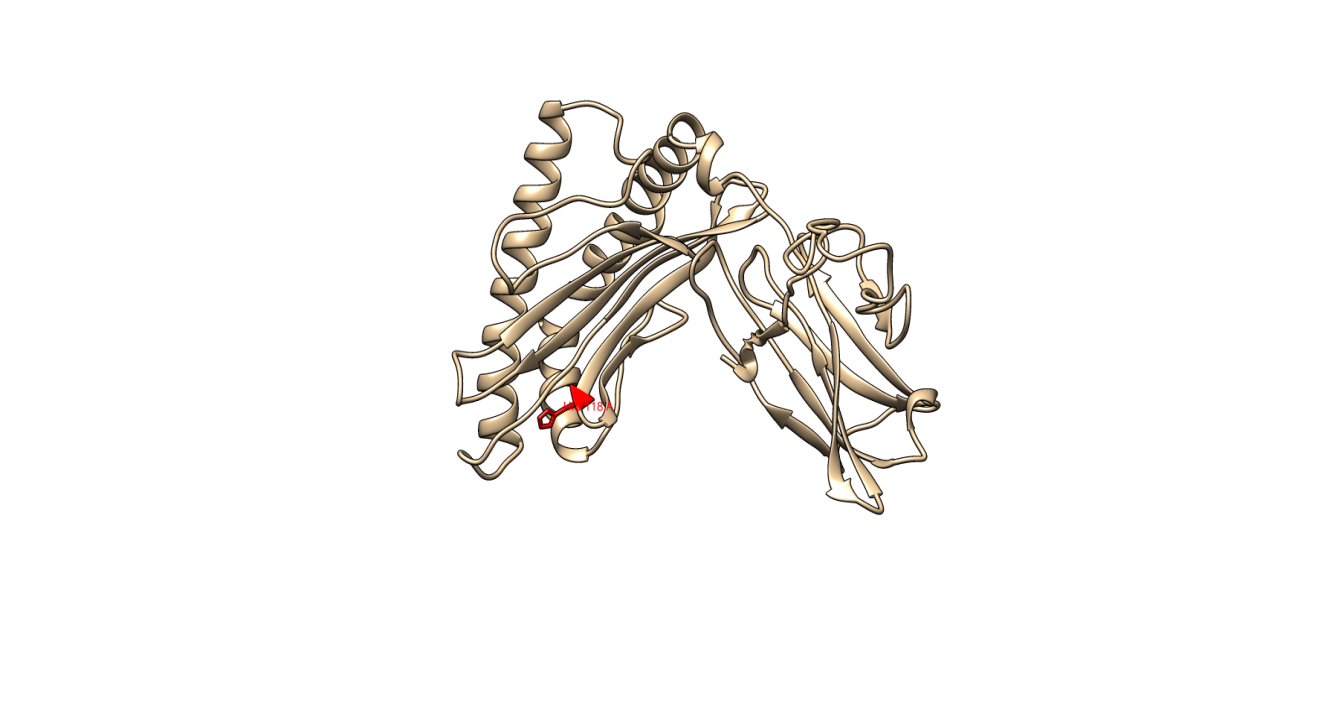 |  | 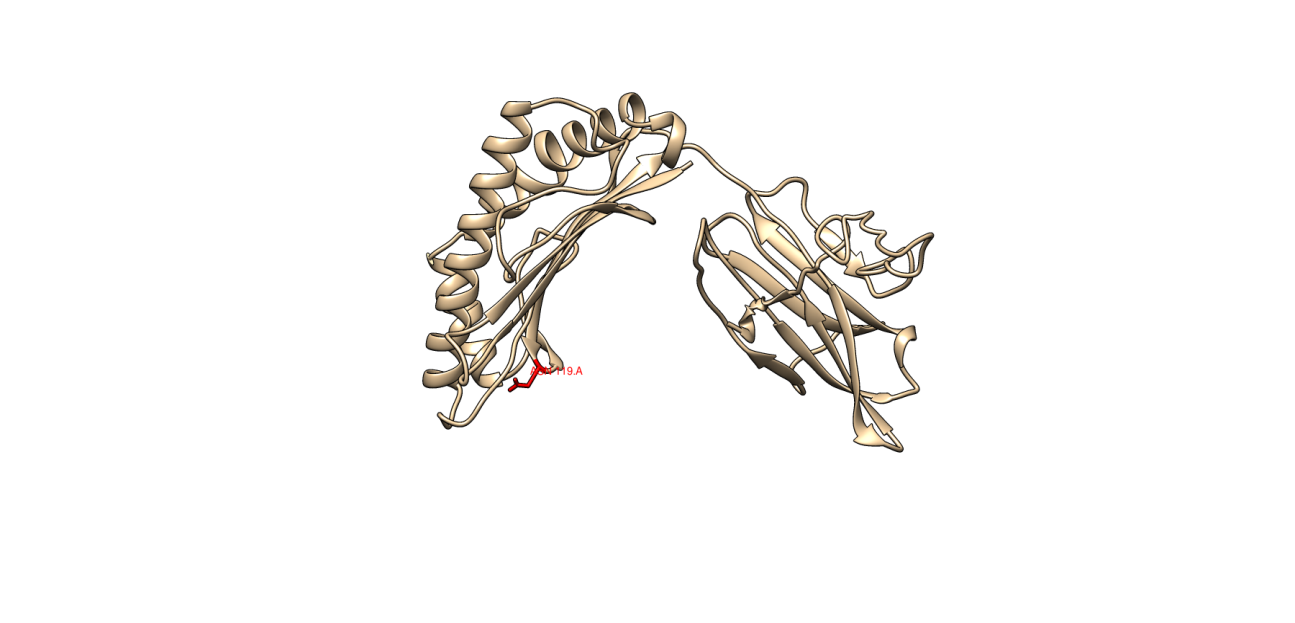 |  | 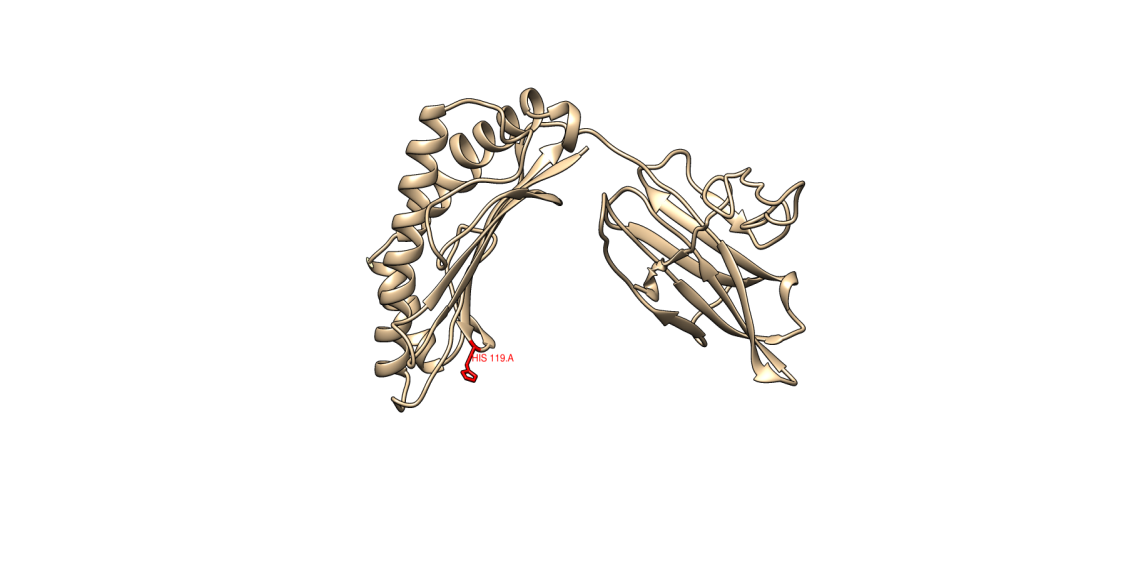 |
| **protein position 153 changed from Aspartic acid (green (upper image)) to  Glycine (red (bottom image))** | **SNP ID: rs556645753** | **SNP ID: rs867319917** | | **SNP ID: rs748013931** | |
|  | 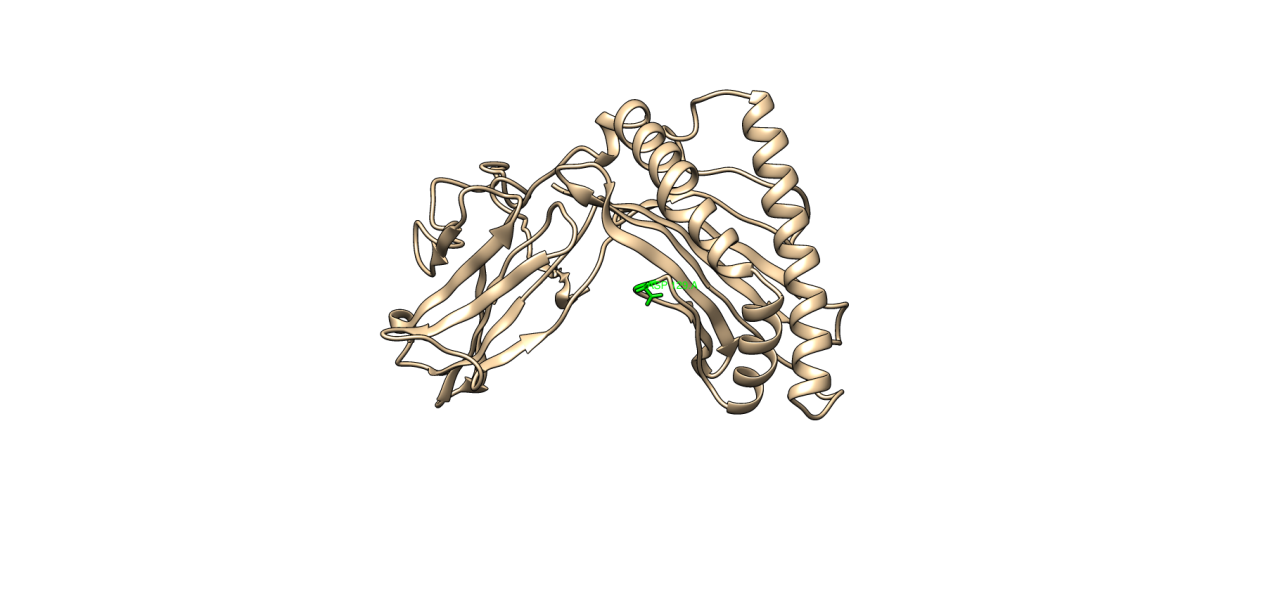 | **protein position 157 changed from Tryptophan (green (upper image)) to  Arginine (red (bottom image))** | 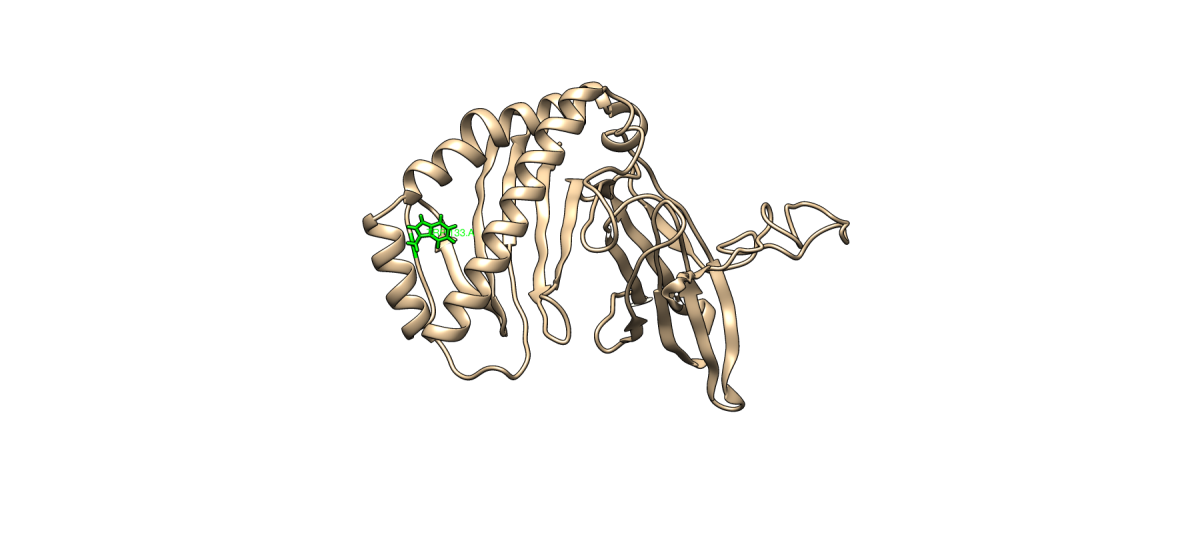 | **protein position 158 changed from Threonine (green (upper image)) to  Proline (red (bottom image))** | 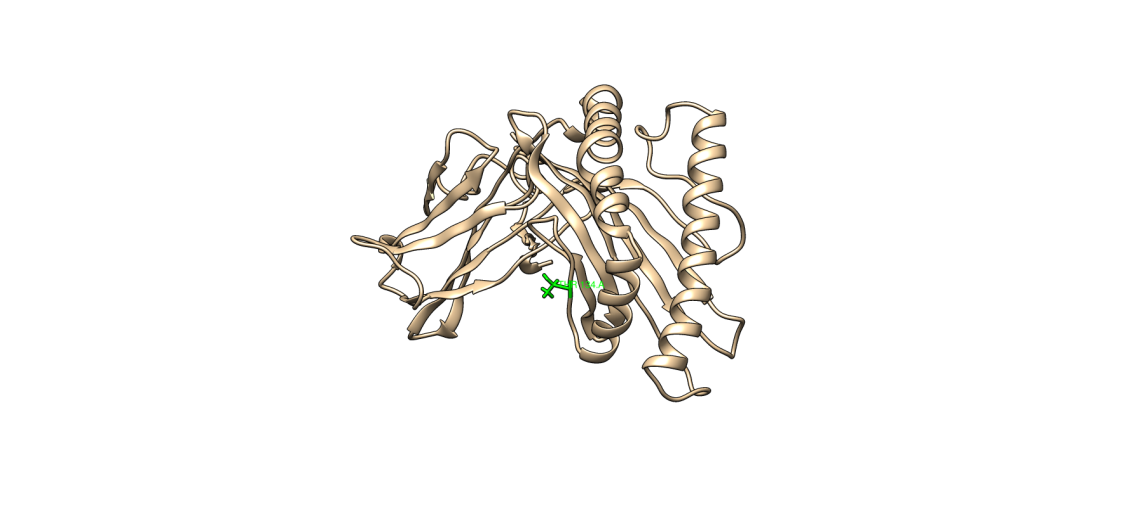 |
|  | 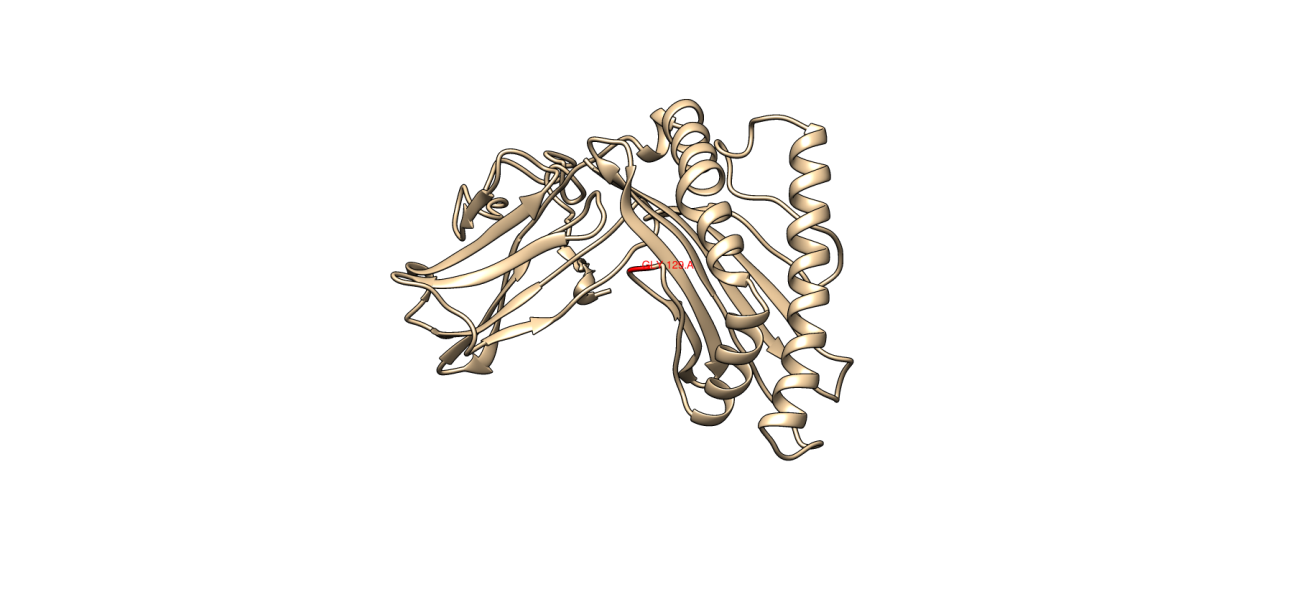 |  | 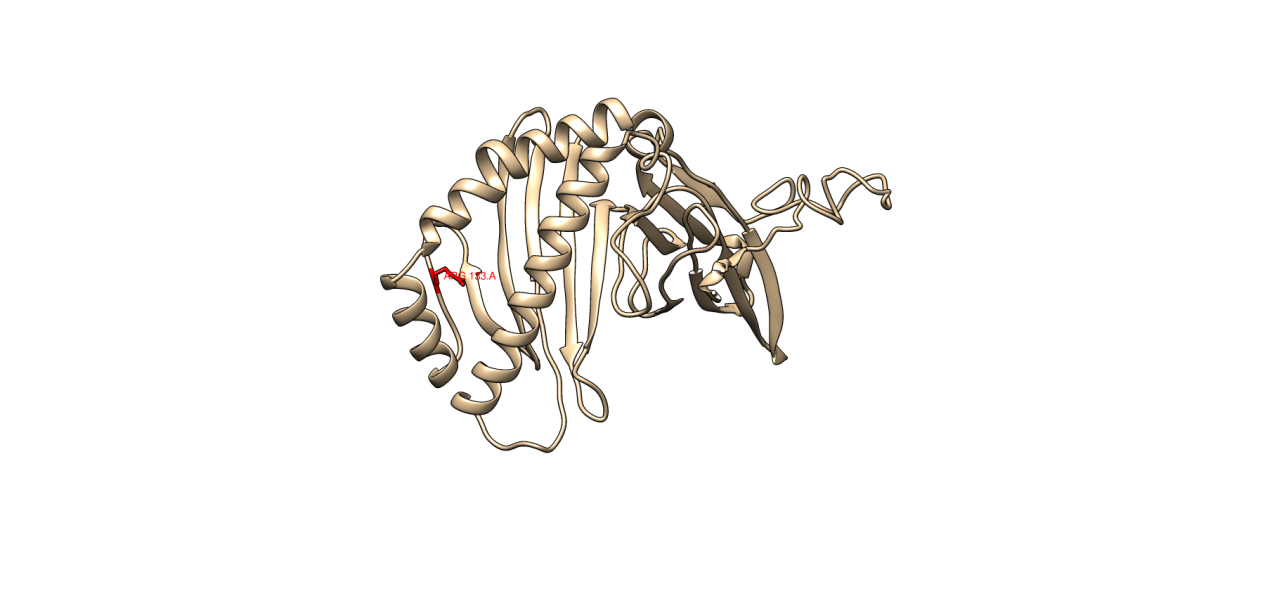 |  | 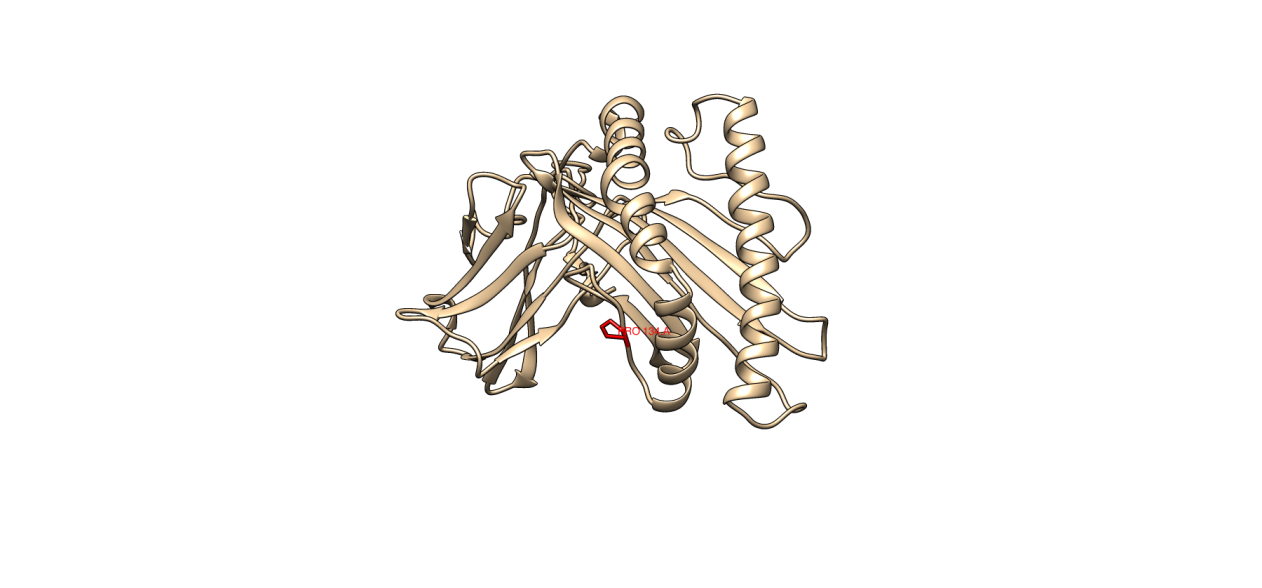 |
| **protein position 188 changed from Cysteine (green (upper image)) to  Serine (red (bottom image))** | **SNP ID: rs780697086** | **SNP ID: rs1397132797** | | **SNP ID: rs1379742188** | |
|  | 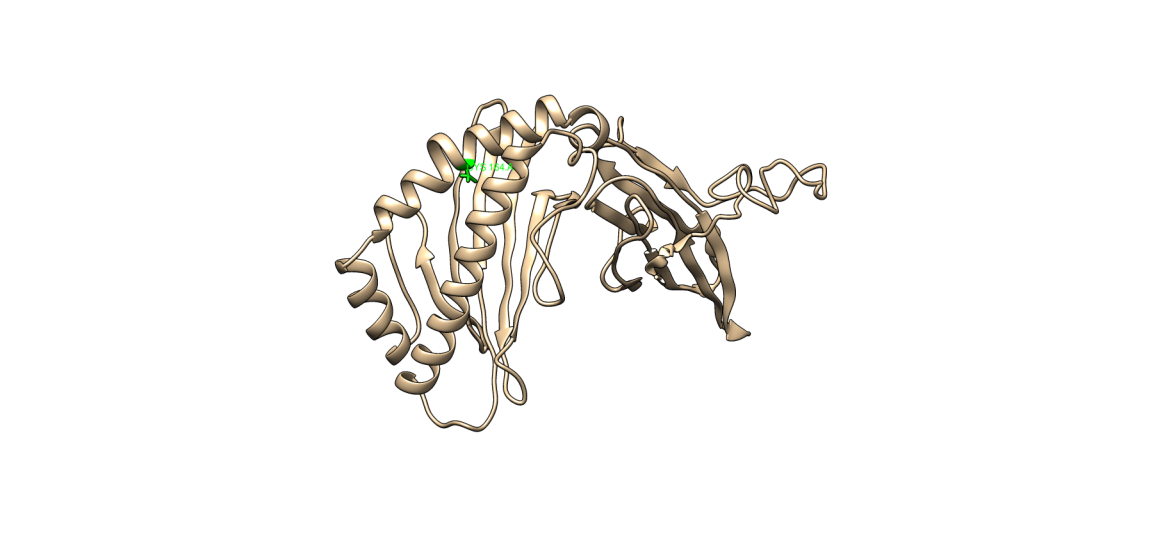 | **protein position 196 changed from Leucine (green (upper image)) to  Proline (red (bottom image))** | 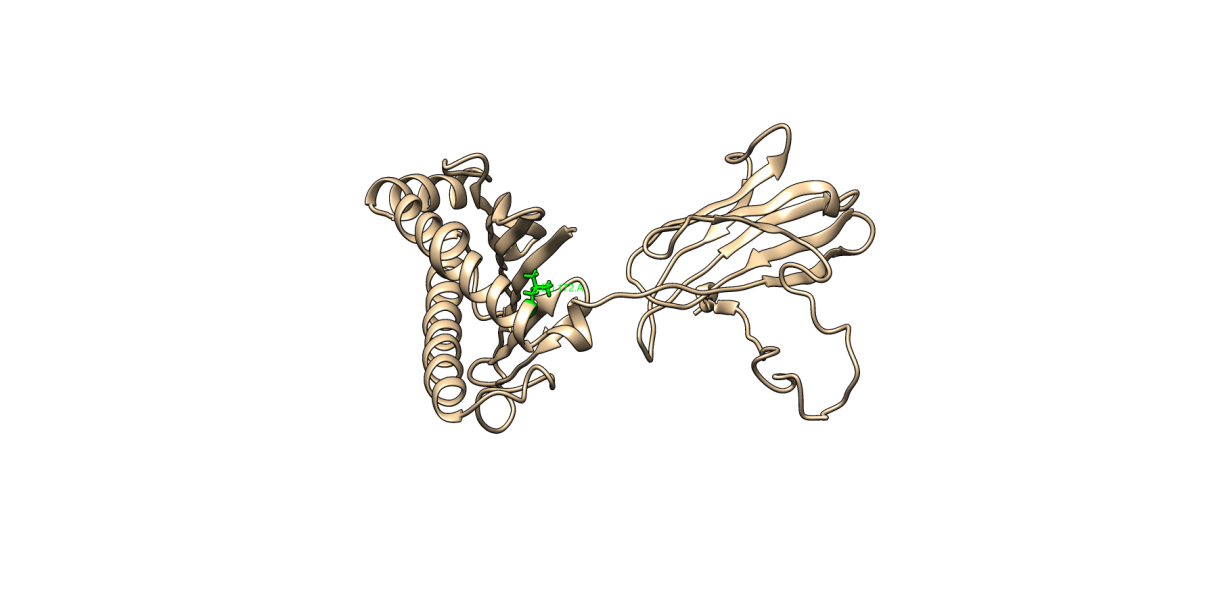 | **protein position 205 changed from Arginine (green (upper image)) to  Serine (red (bottom image))** | 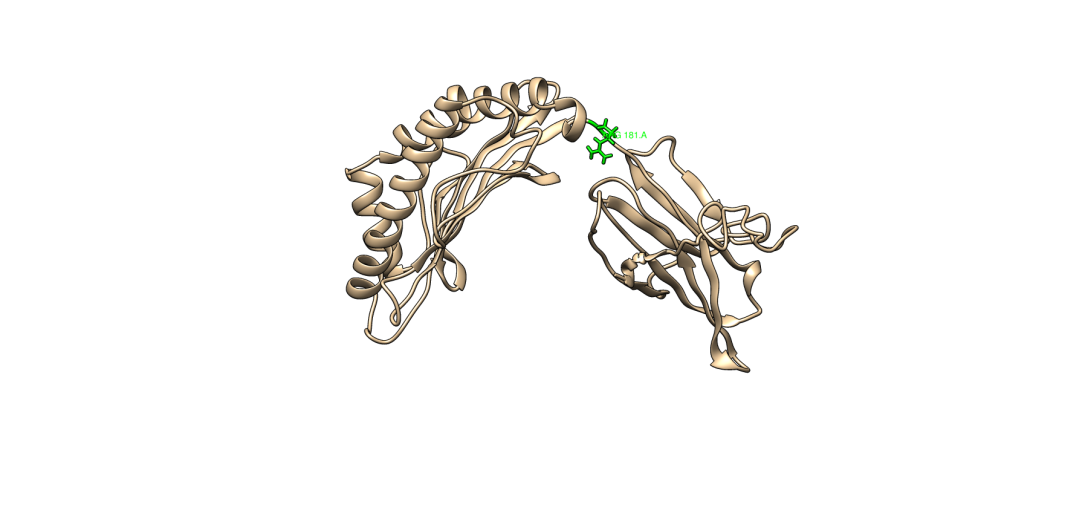 |
|  | 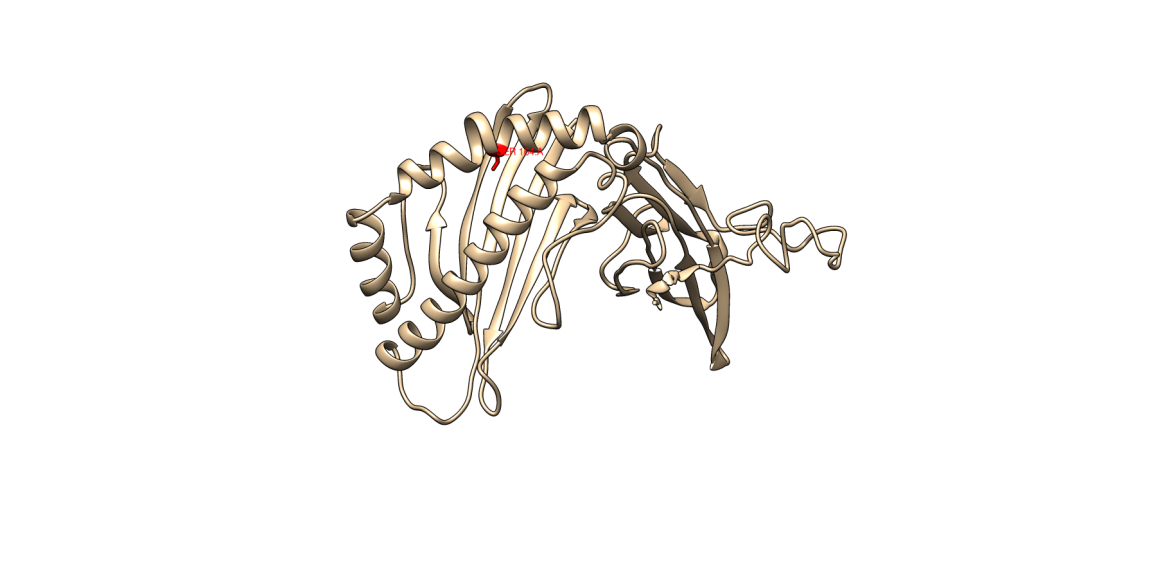 |  | 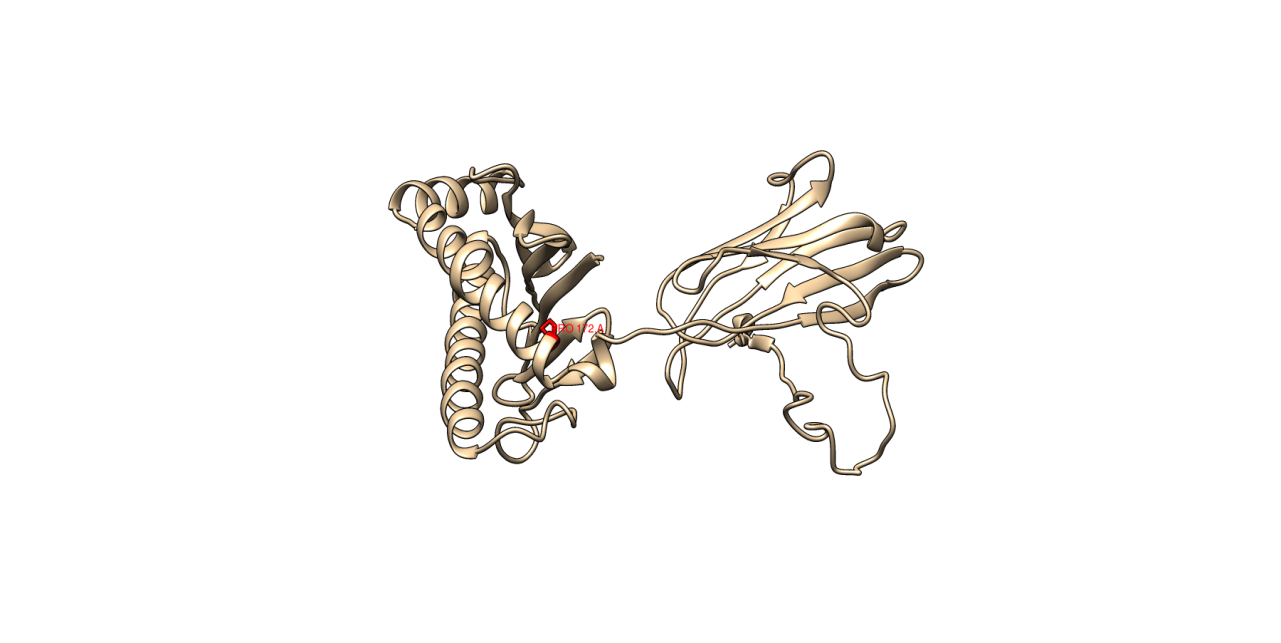 |  | 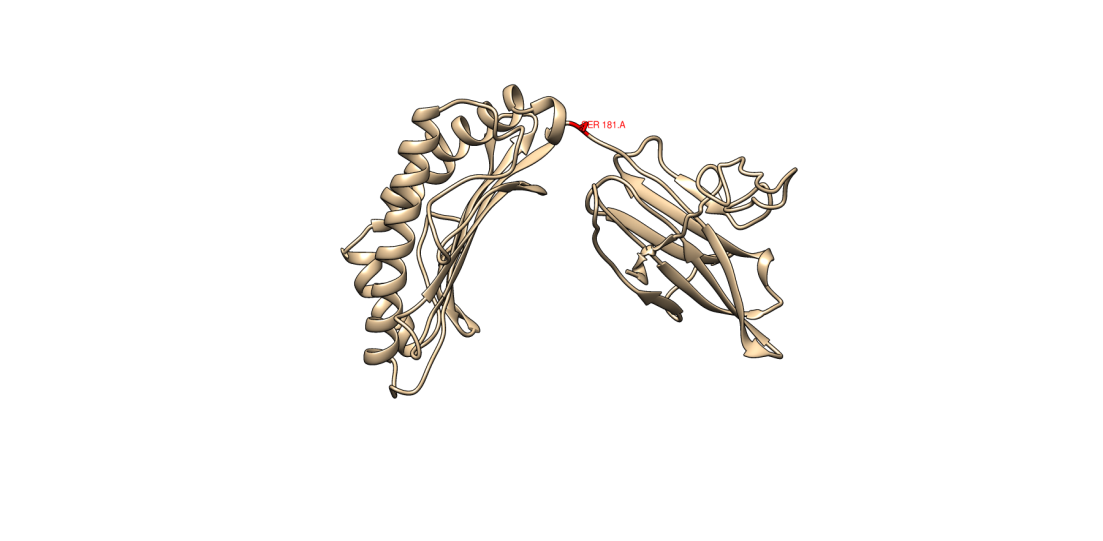 |
| **protein position 209 changed from Proline (green (upper image)) to  Alanine (red (bottom image))** | **SNP ID: rs144577485** | **SNP ID: rs1472538844** | | **SNP ID: rs770027530** | |
|  | 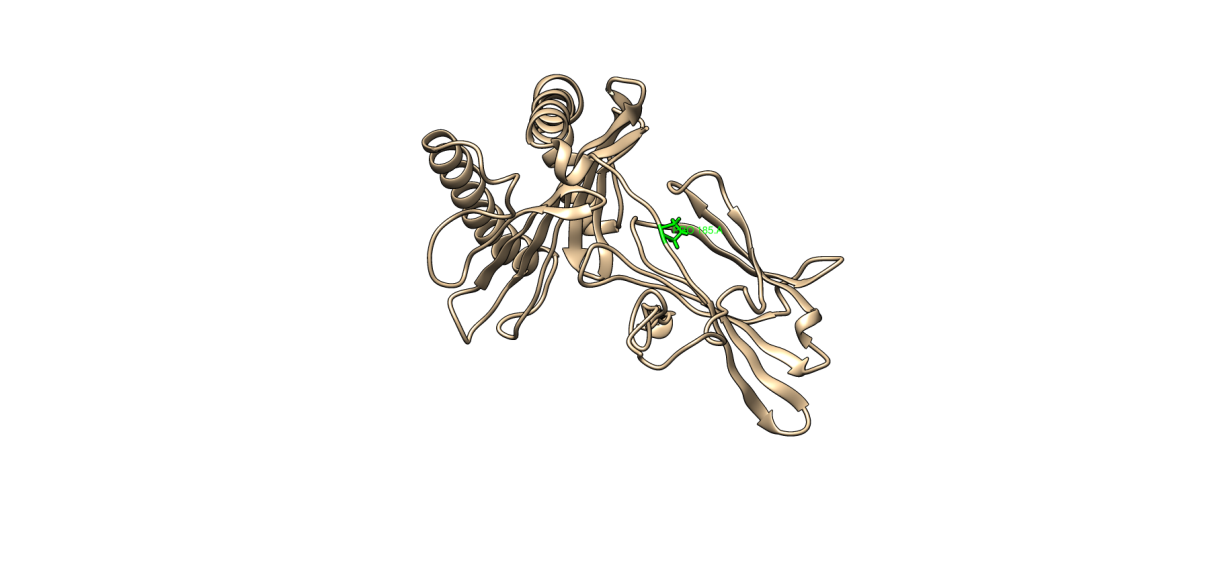 | **protein position 209 changed from Proline (green (upper image)) to  Arginine (red (bottom image))** | 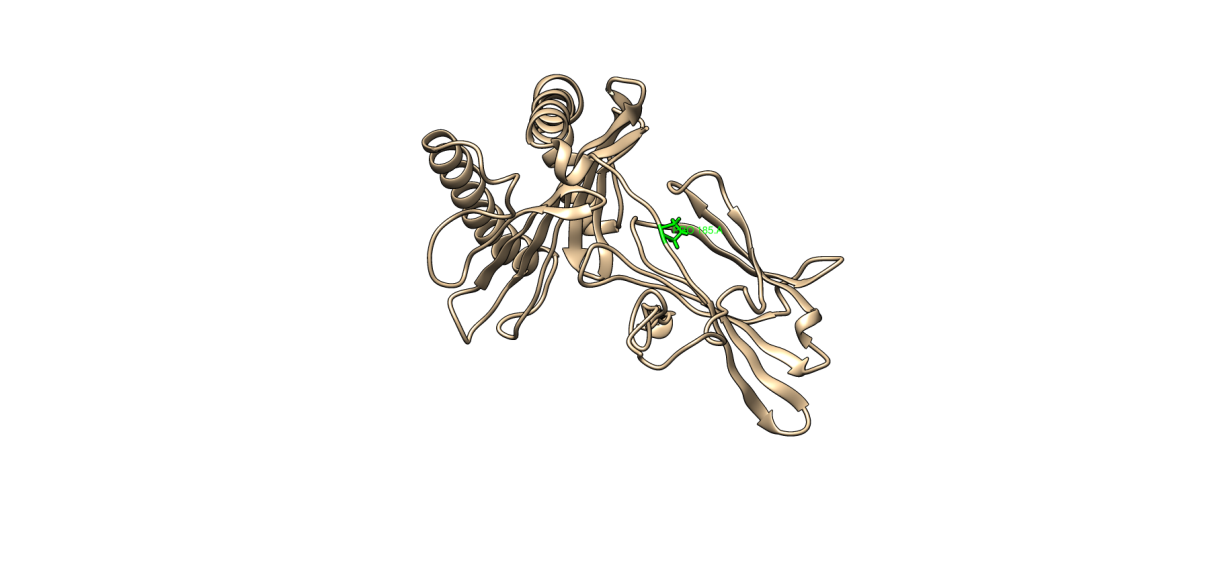 | **protein position 227 changed from Cysteine (green (upper image)) to  Phenylalanine (red (bottom image))** | 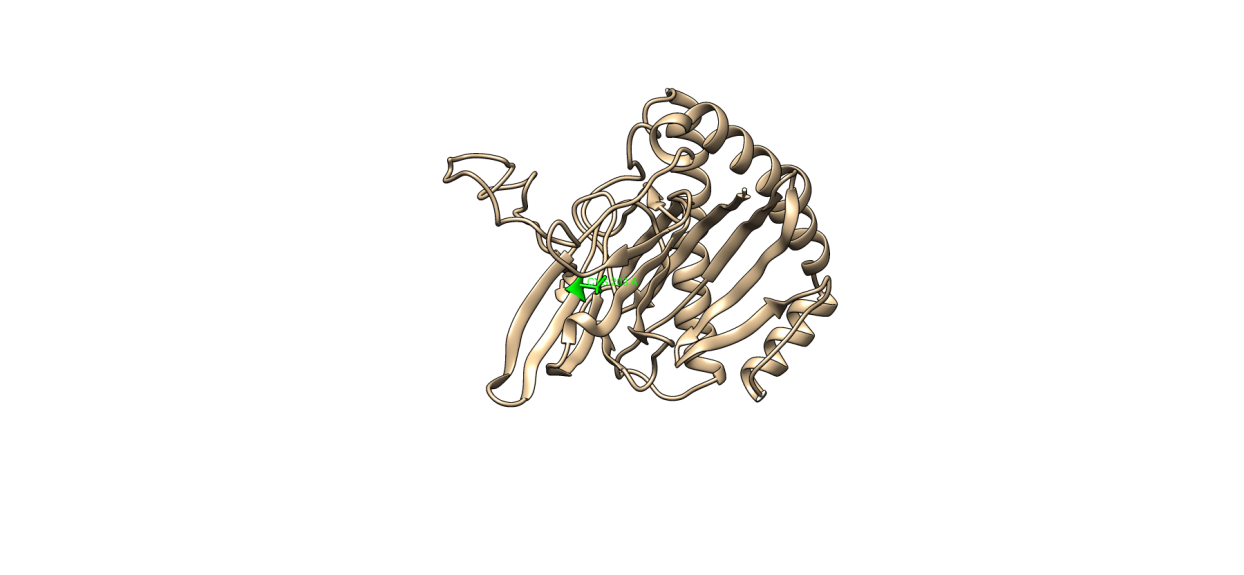 |
|  | 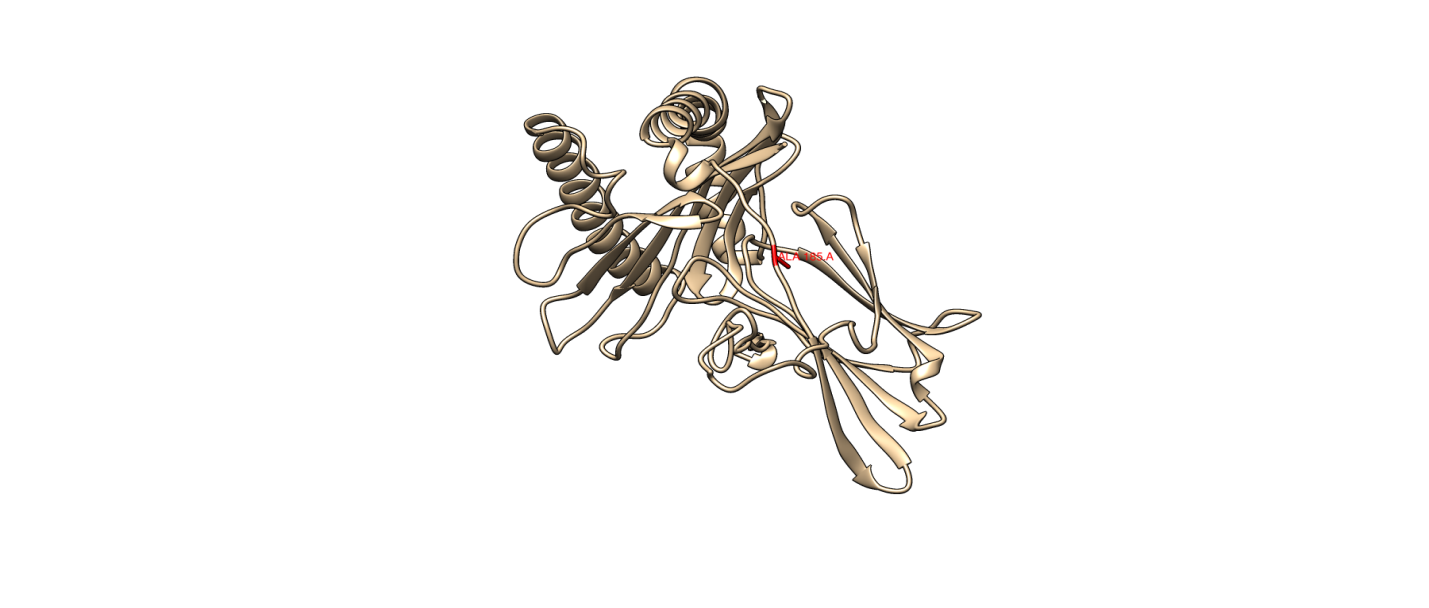 |  | 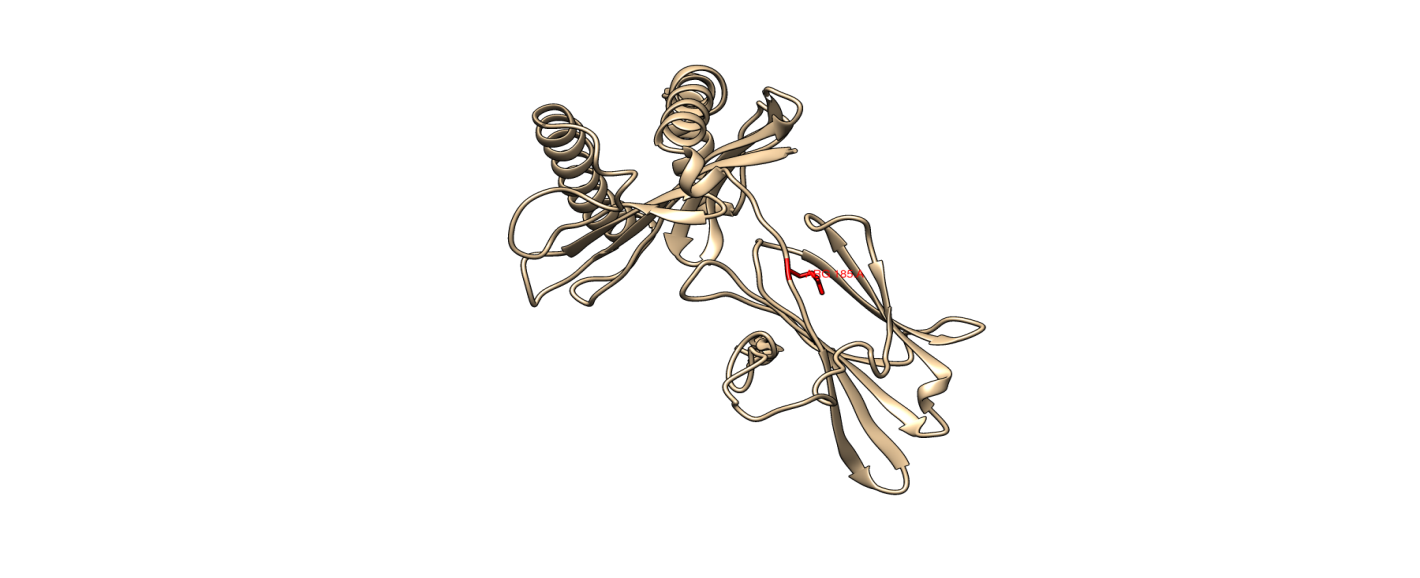 |  | 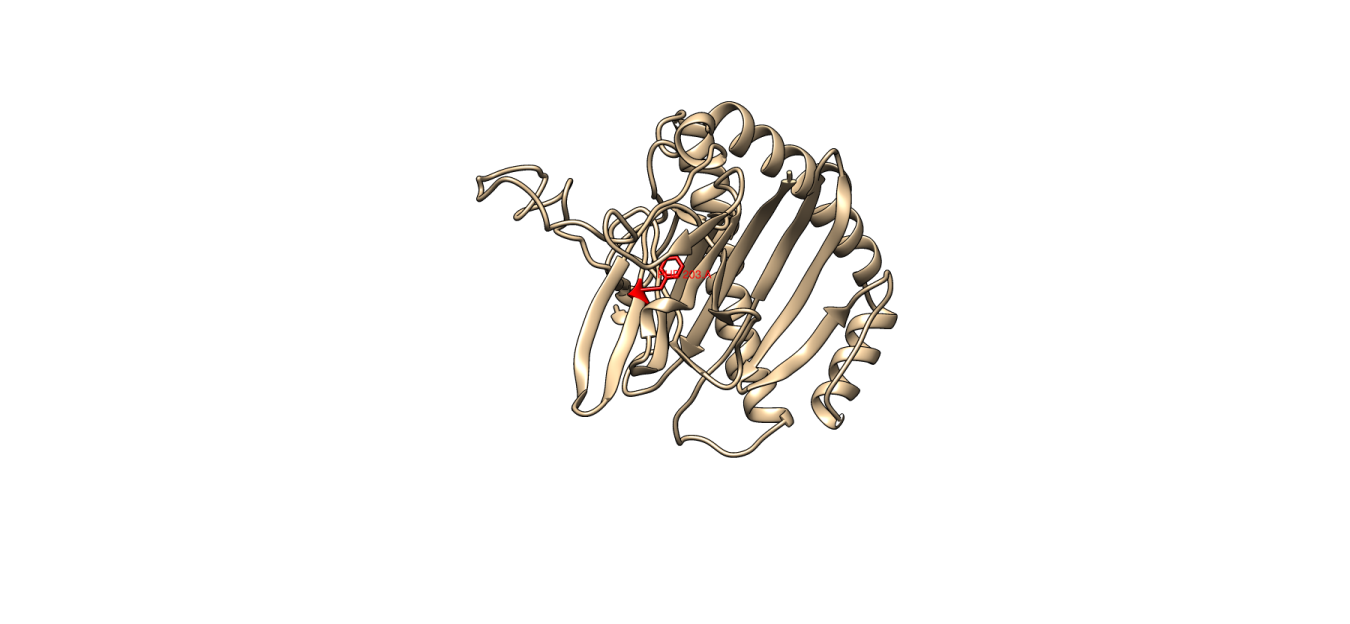 |
| **SNP ID: rs770027530** | | **SNP ID: rs1200732770** | | **SNP ID: rs142596947** | |
| **Protein position 227 changed from Cysteine (green (upper image)) to  Tyrosine (red (bottom image))** | 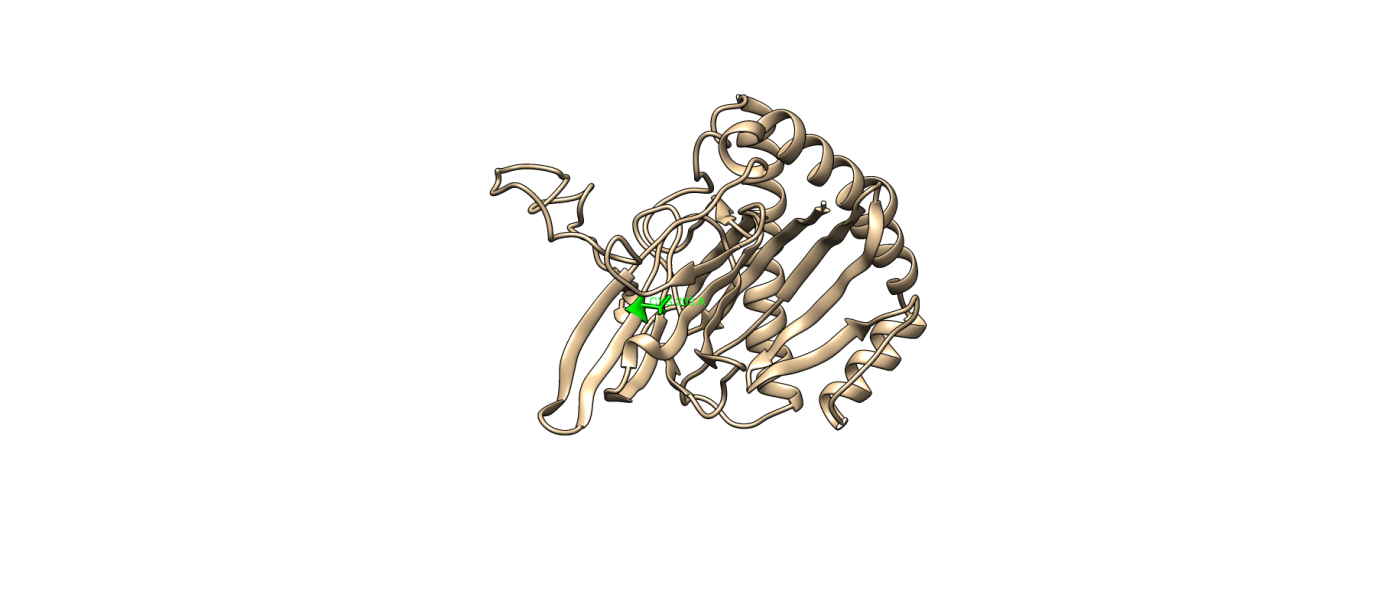 | **protein position 229 changed from Alanine (green (upper image)) to  Aspartic acid (red (bottom image))** | 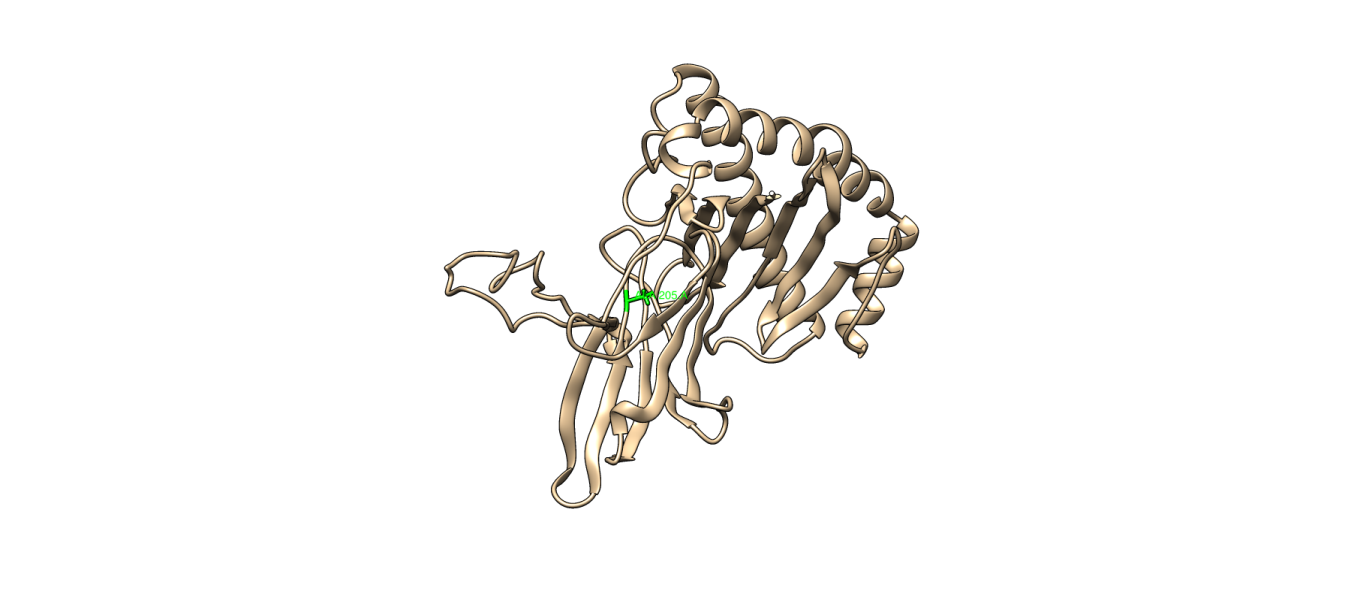 | **protein position 234 changed from Proline (green (upper image)) to  Threonine (red (bottom image))** | 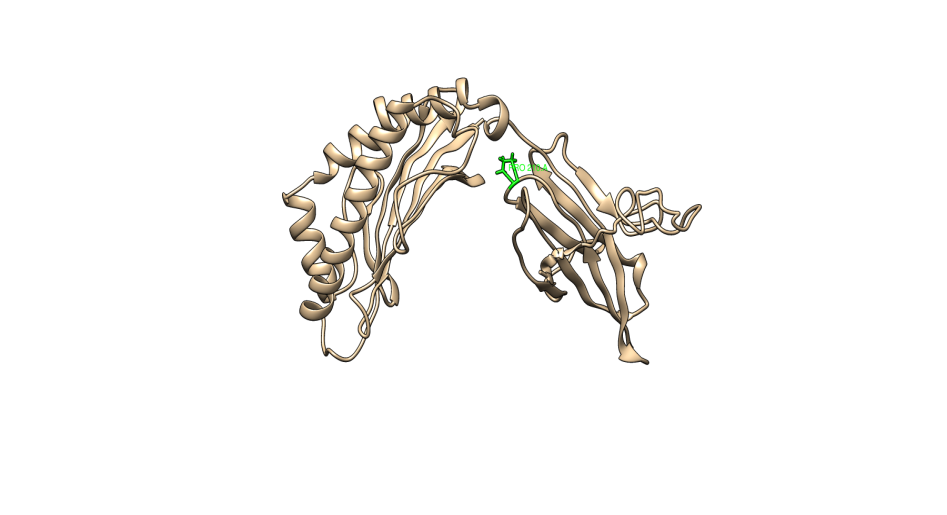 |
|  | 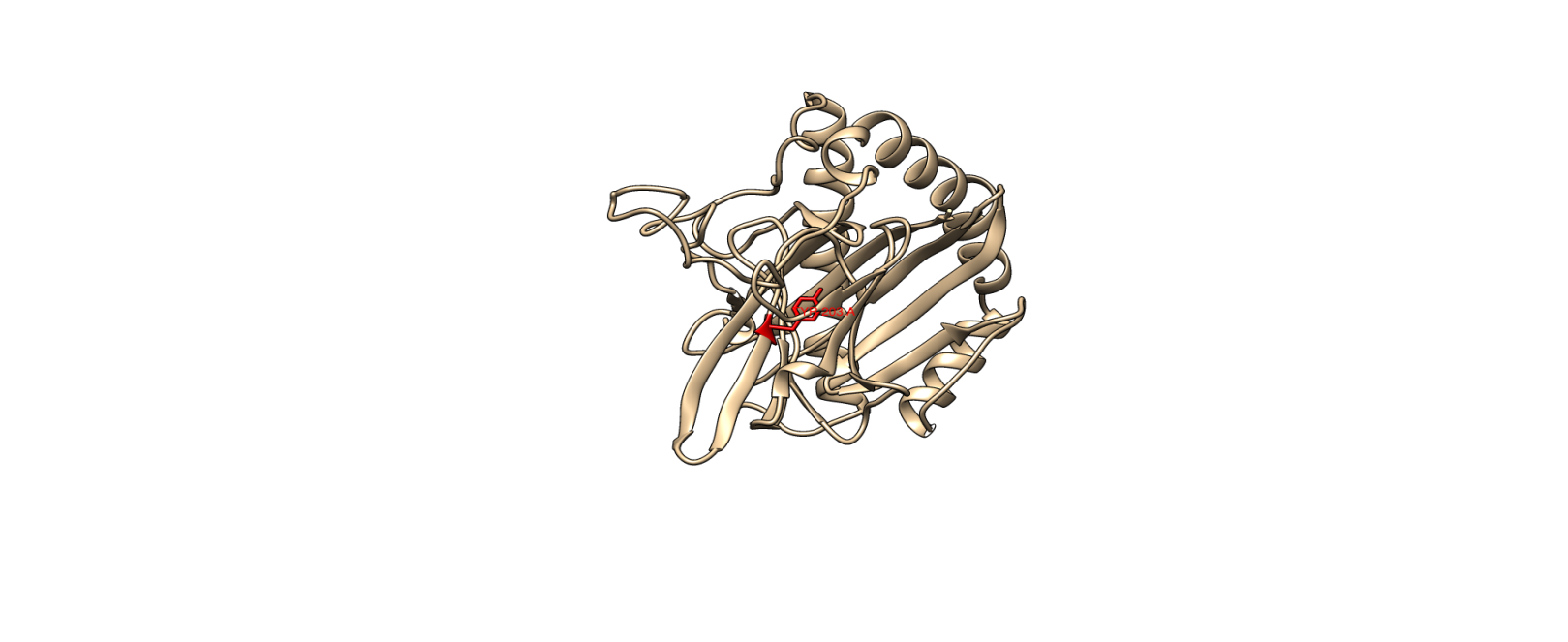 |  | 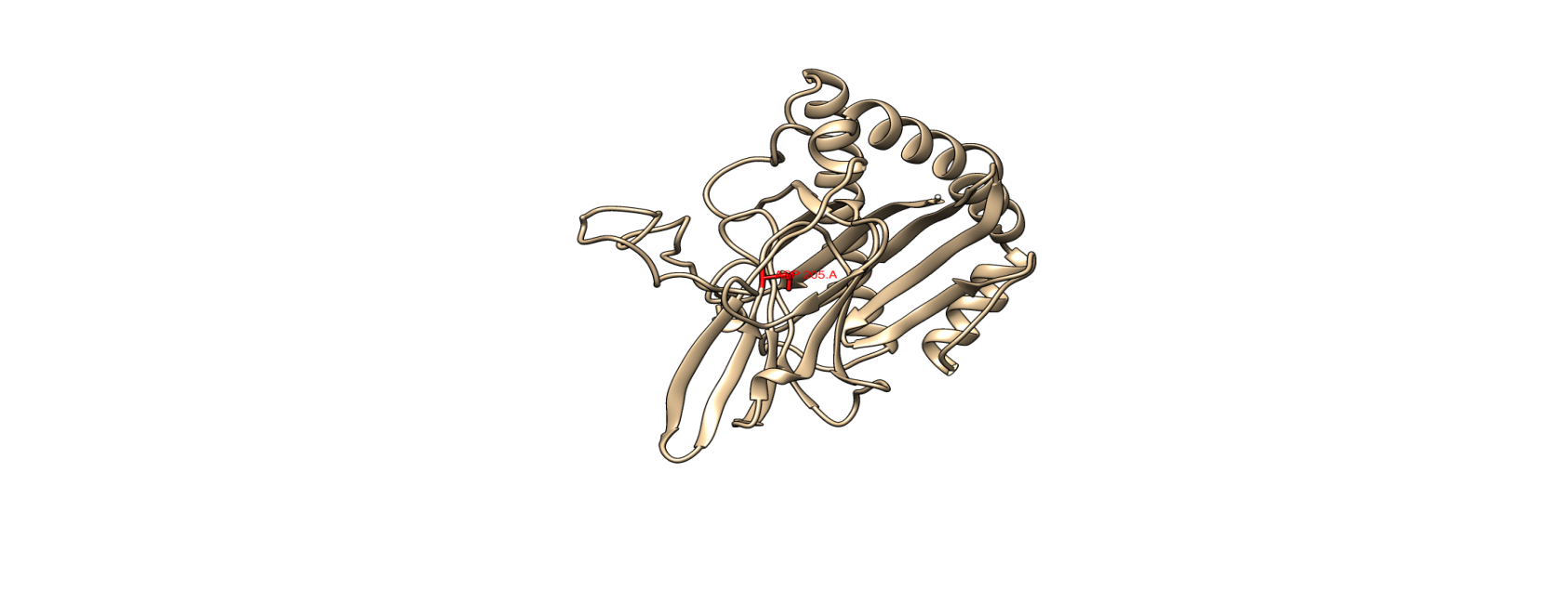 |  | 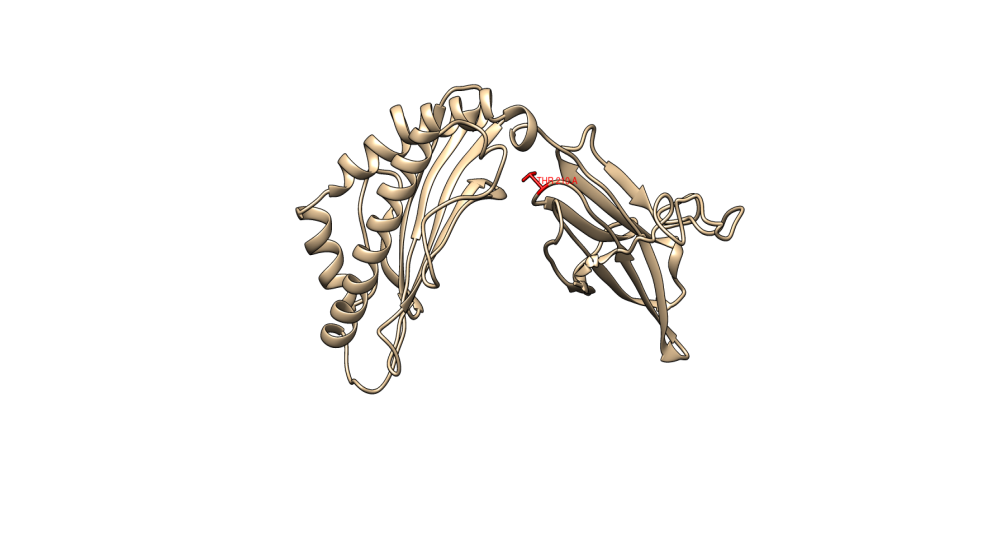 |
| **SNP ID: rs1430565057** | | **SNP ID: rs750238738** | | **SNP ID: rs754527717** | |
| **protein position 234 changed from Proline (green (upper image)) to  Leucine (red (bottom image))** | 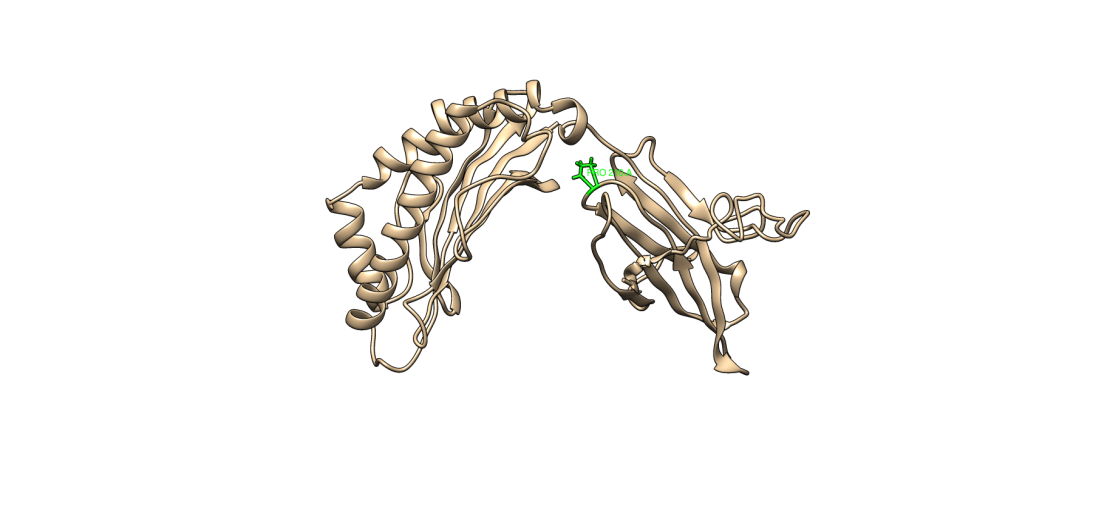 | **protein position 237 changed from Isoleucine (green (upper image)) to  Phenylalanine (red (bottom image))** | 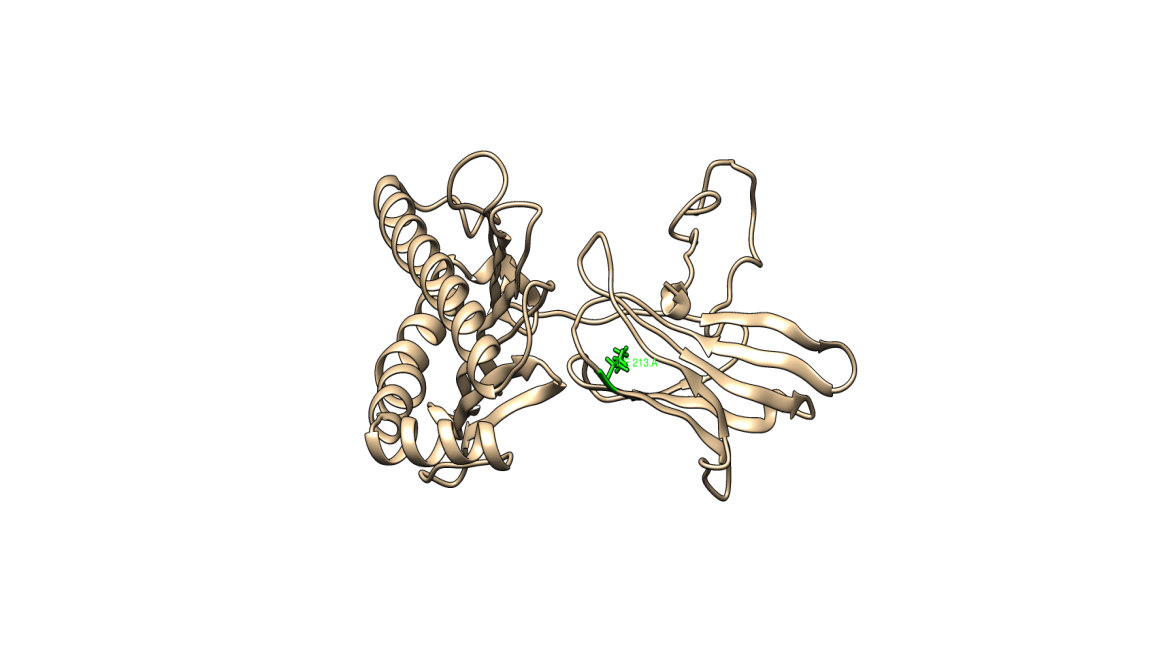 | **protein position 243 changed from Aspartic acid (green (upper image)) to  Glycine (red (bottom image))** | 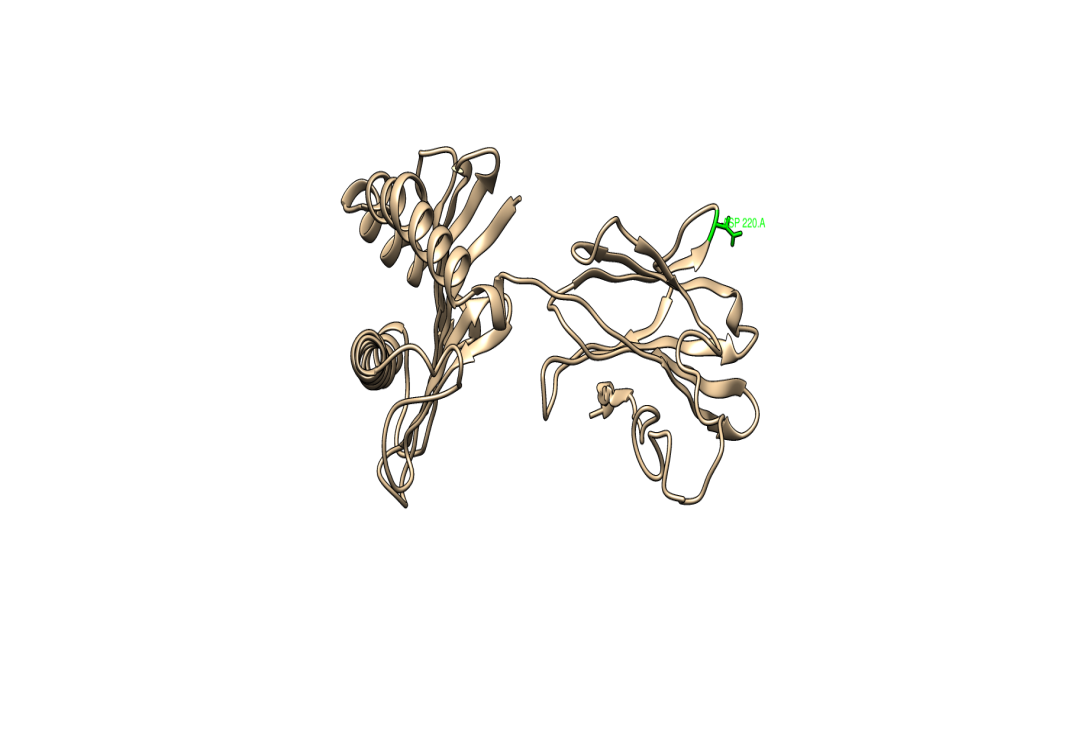 |
|  | 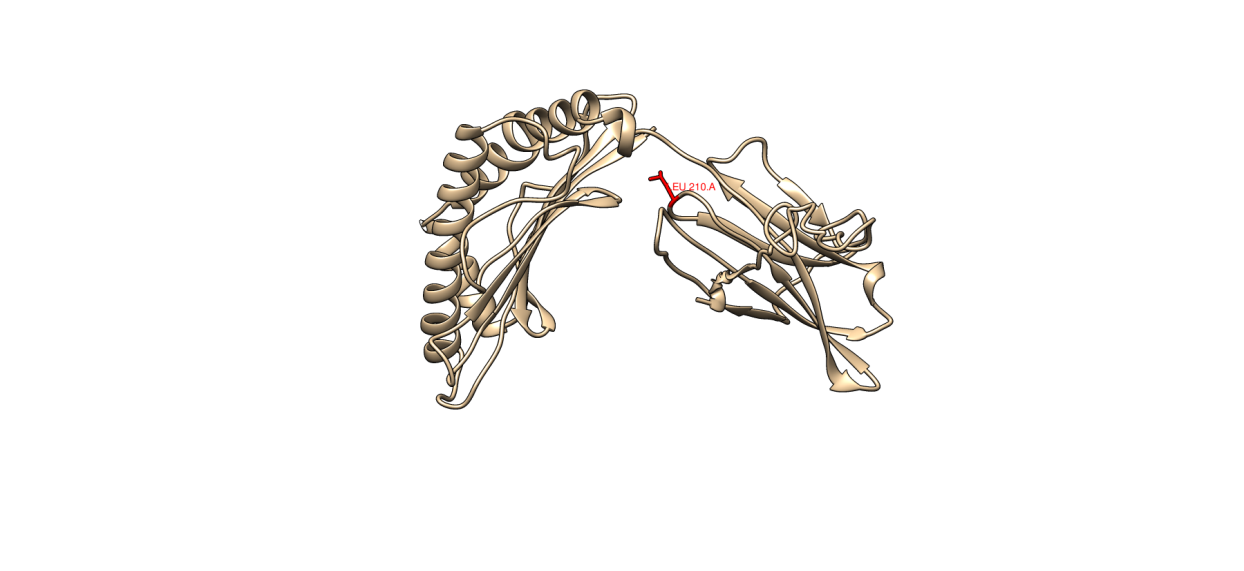 |  | 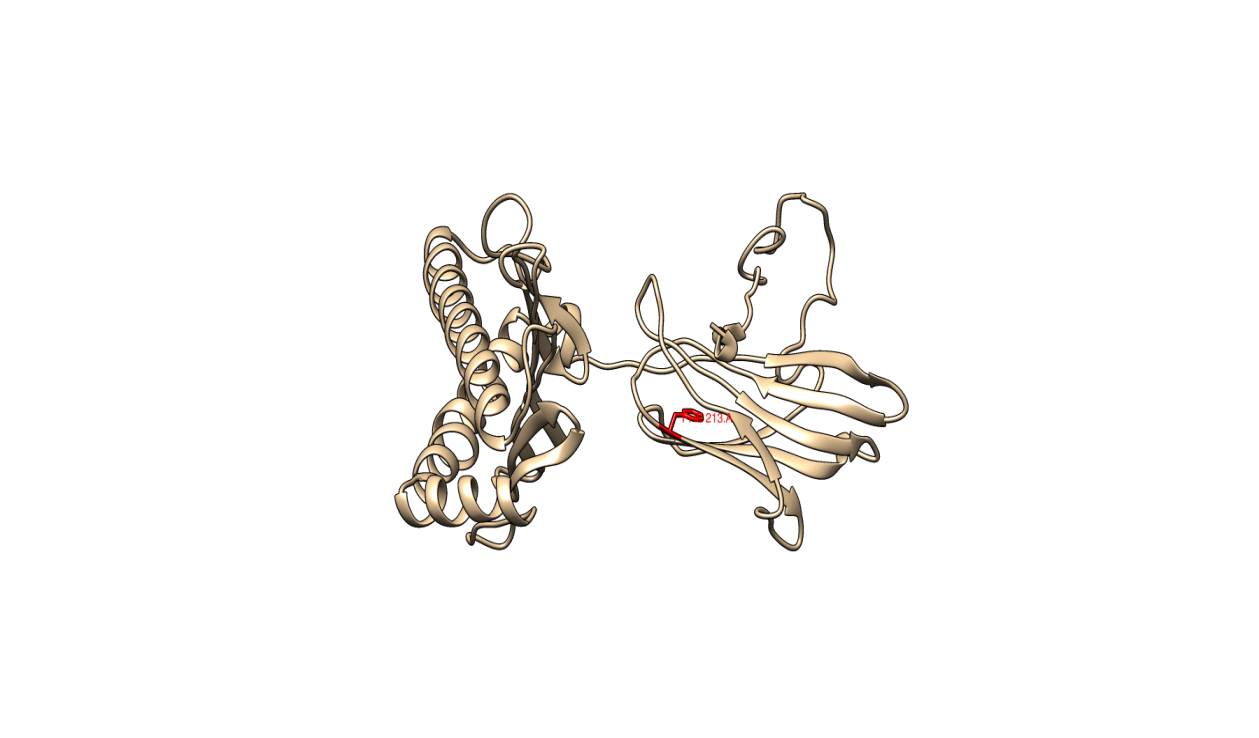 |  | 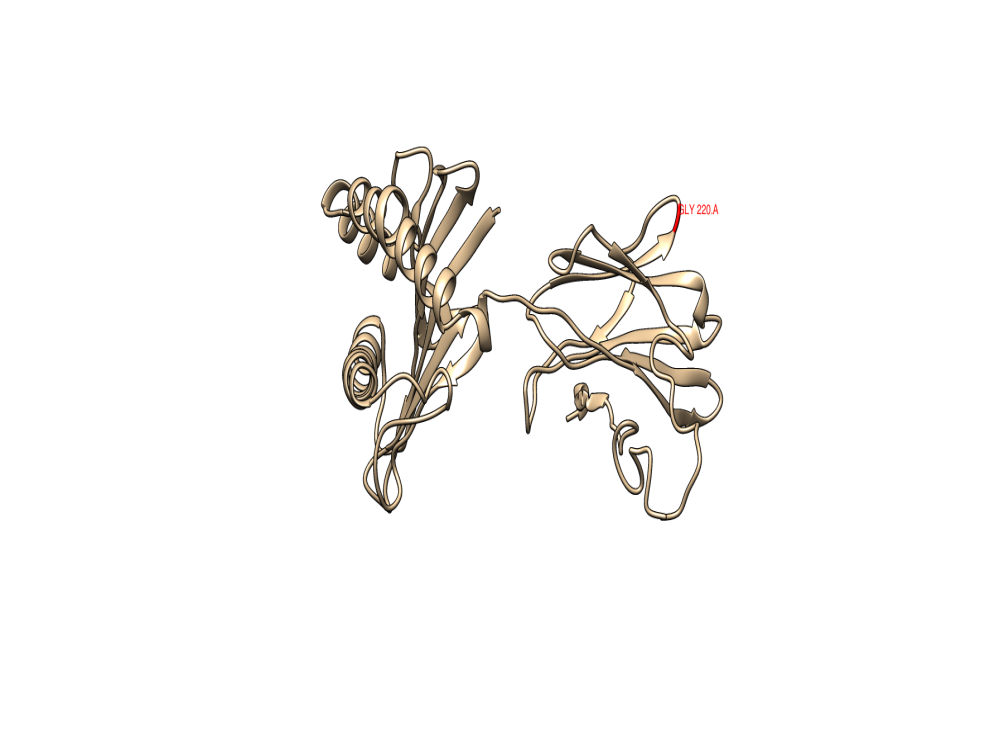 |
| **SNP ID: rs781774818** | | **SNP ID: rs760500349** | | **SNP ID: rs756652306** | |
| **protein position 259 changed from Proline (green (upper image)) to  Histidine (red (bottom image))** | 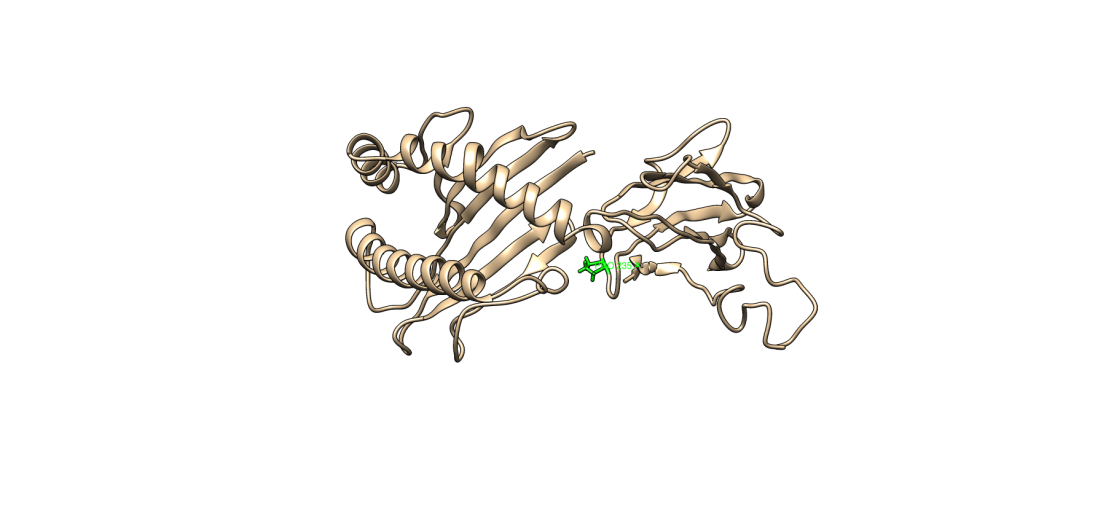 | **protein position 266 changed from Glutamine (green (upper image)) to  Leucine (red (bottom image))** | 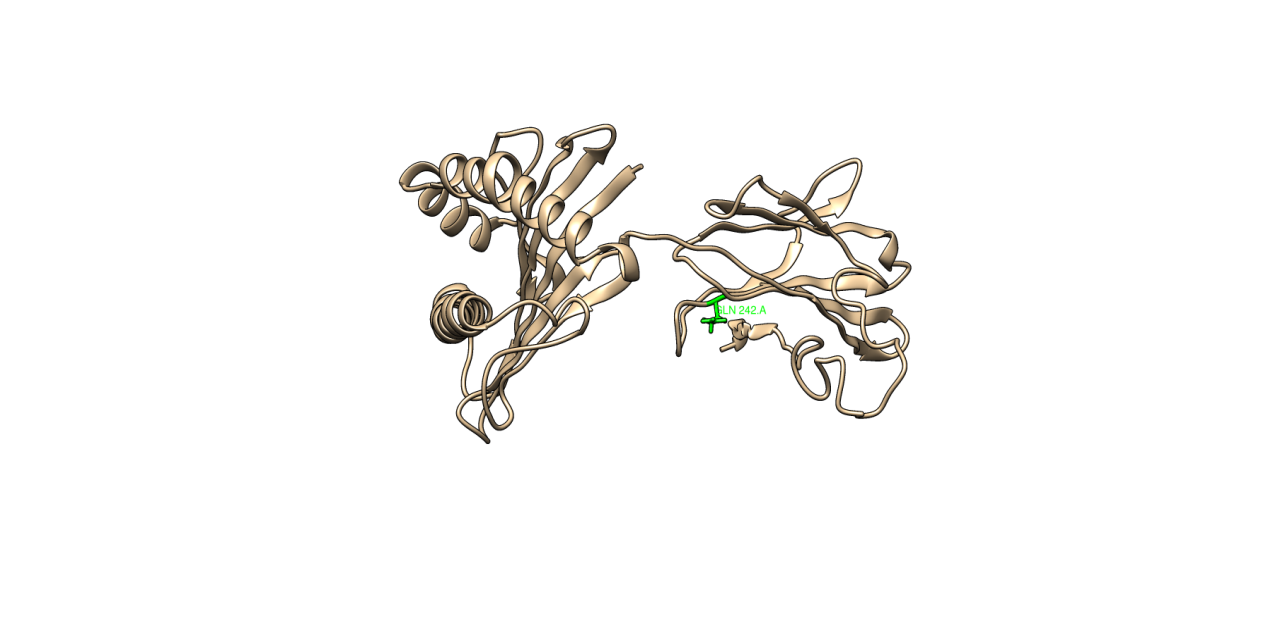 | **protein position 285 changed from Valine (green (upper image)) to  Alanine (red (bottom image))** | 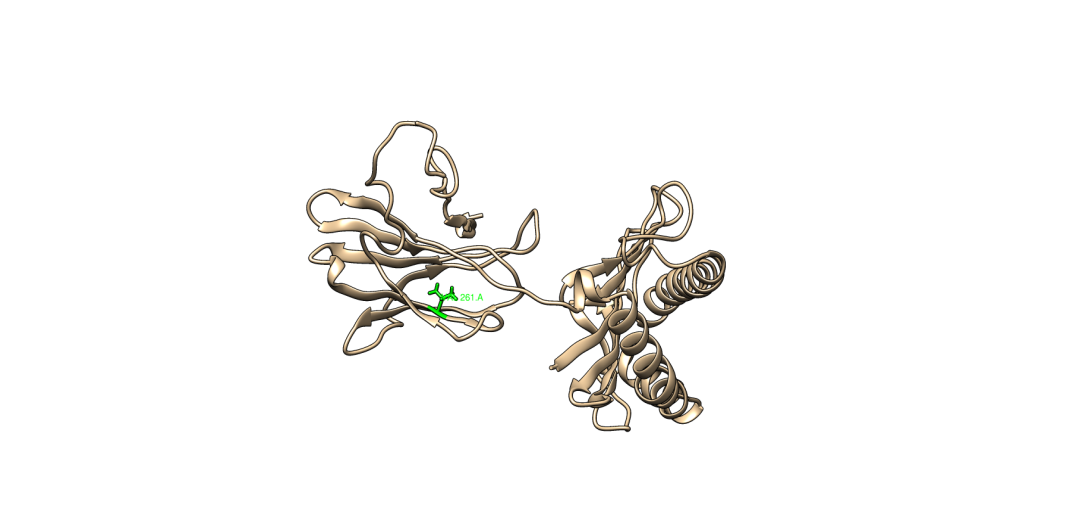 |
|  | 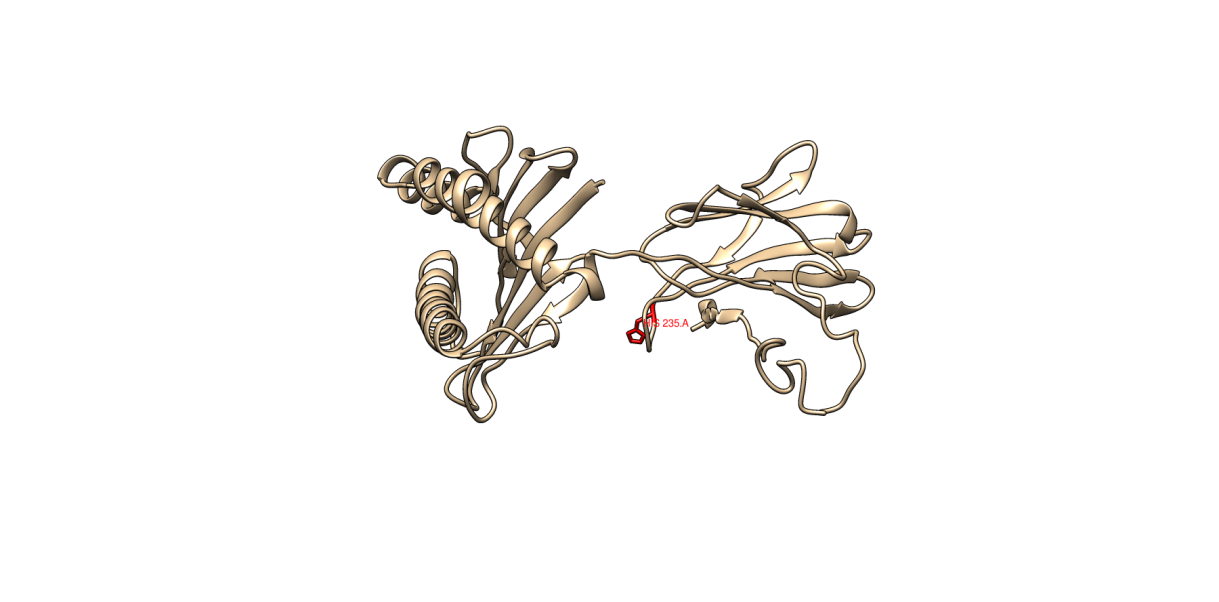 |  | 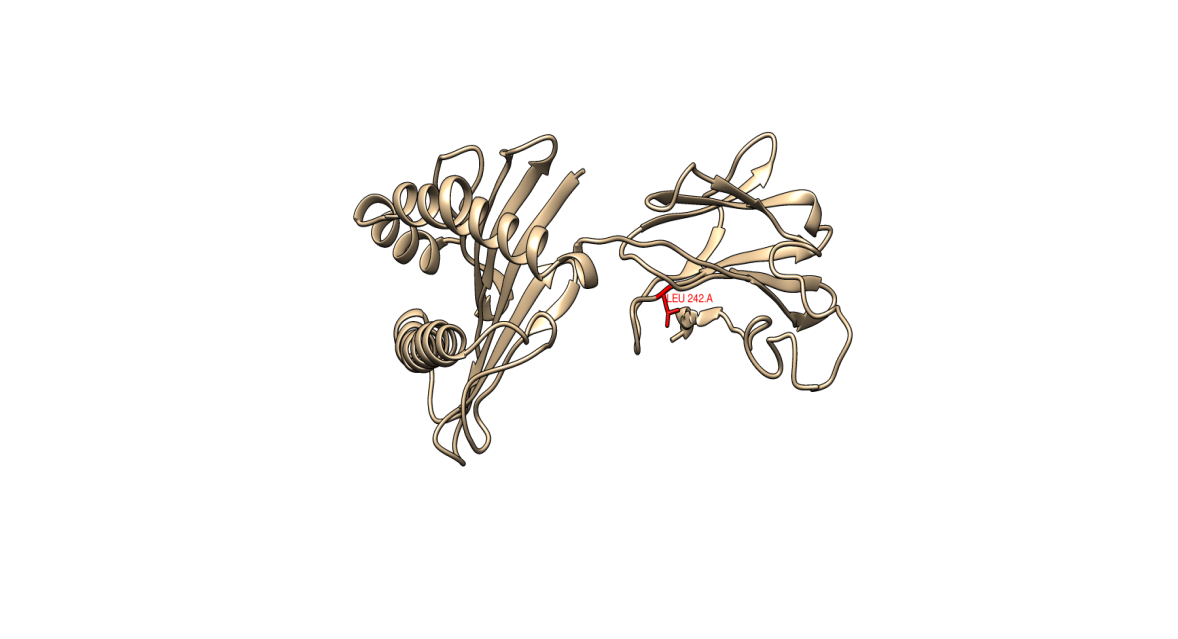 |  | 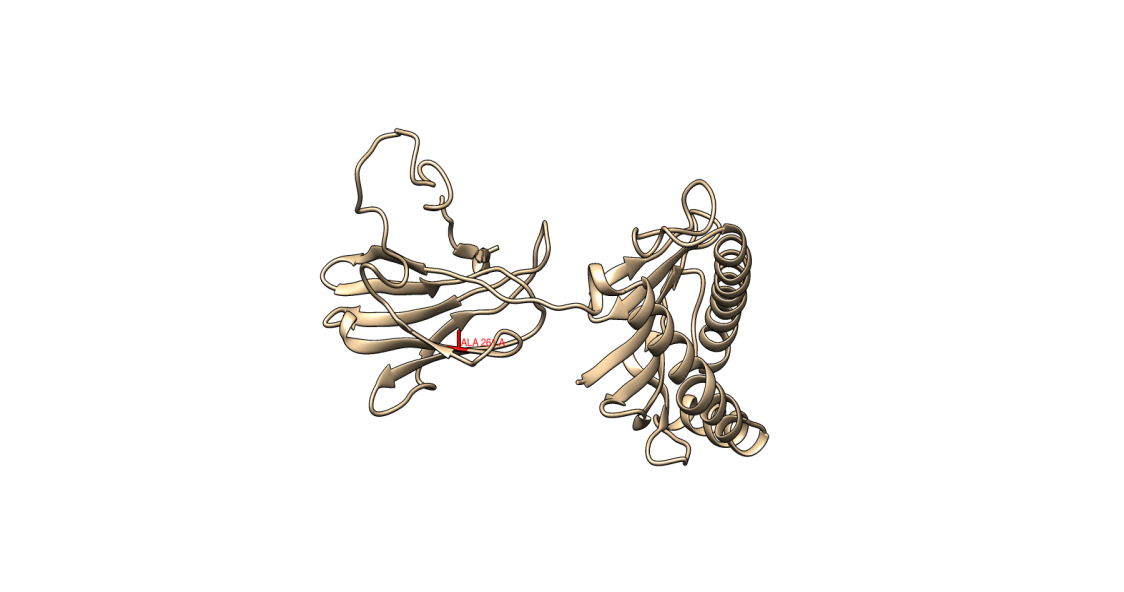 |
| **SNP ID: rs145097667** | | **SNP ID: rs111233577** | | **SNP ID: rs1265409678** | |
| **protein position 287changed from Histidine (green (upper image)) to  Tyrosine (red (bottom image))** | 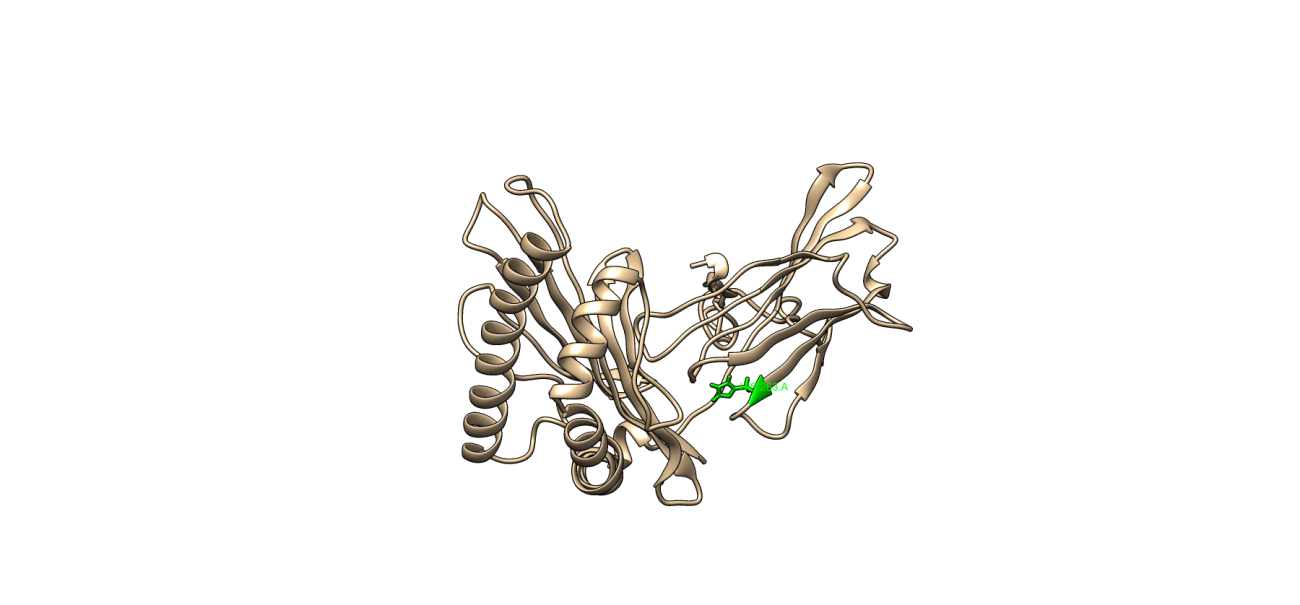 | **protein position 290 changed from Leucine (green (upper image)) to  Arginine (red (bottom image))** | 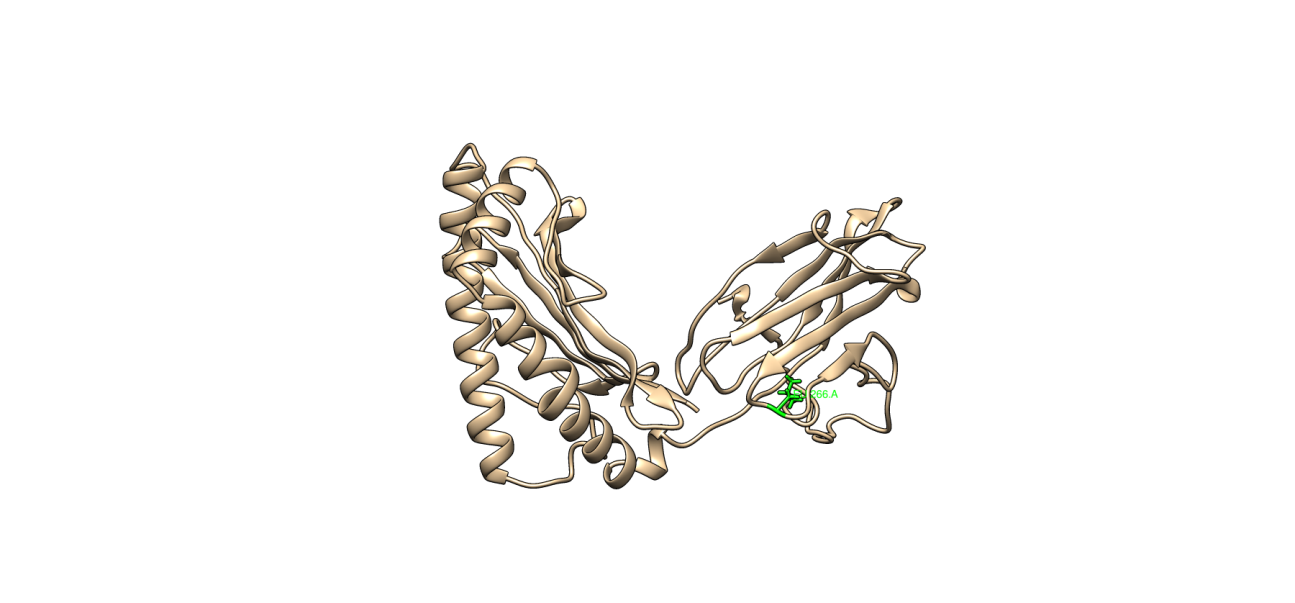 | **protein position 294 changed from Leucine (green (upper image)) to  Arginine (red (bottom image))** | 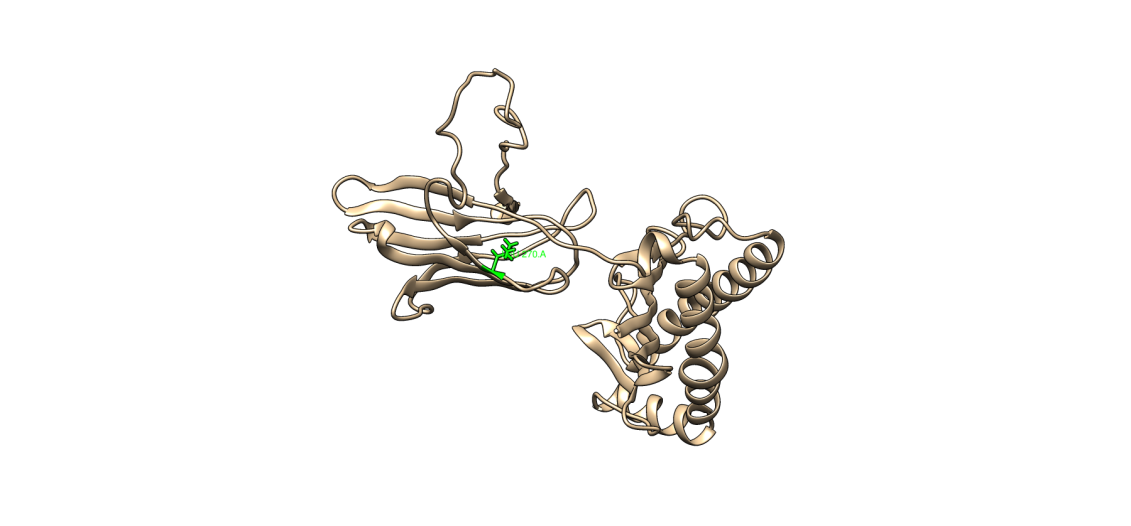 |
|  | 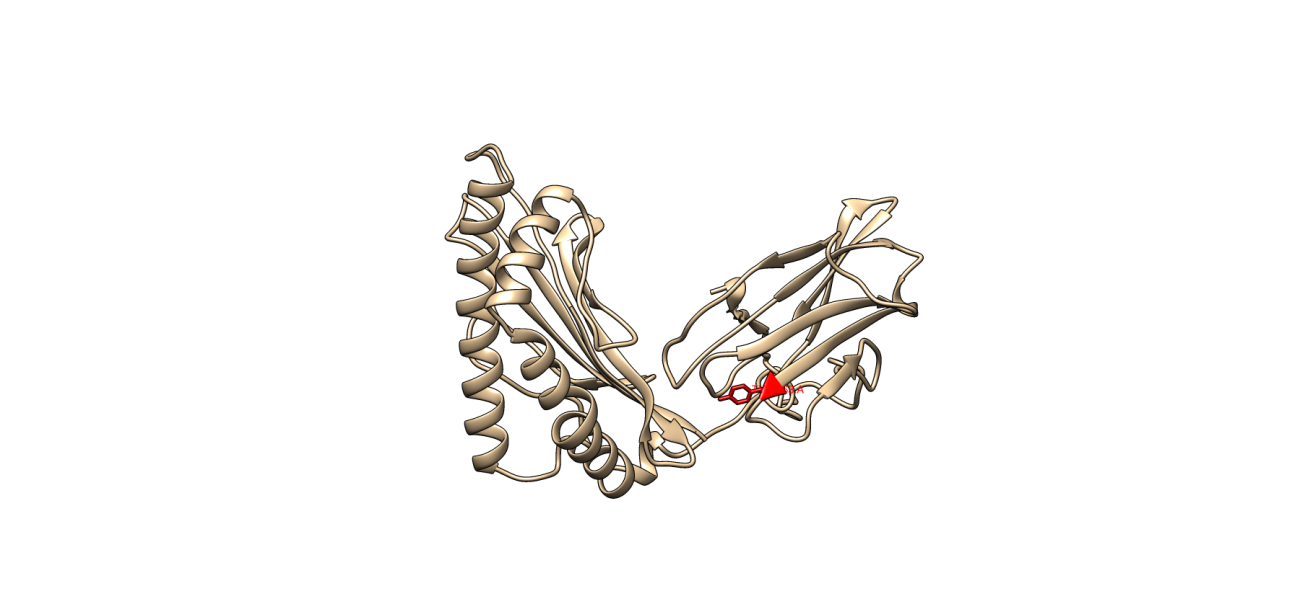 |  | 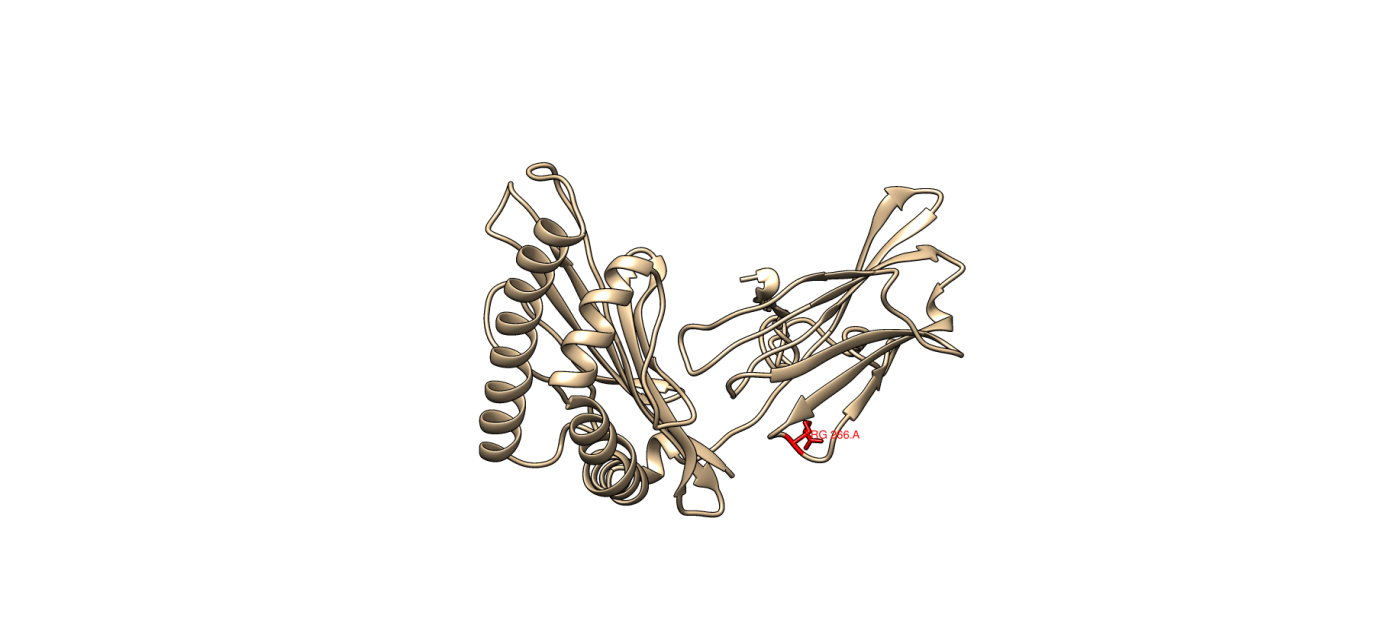 |  | 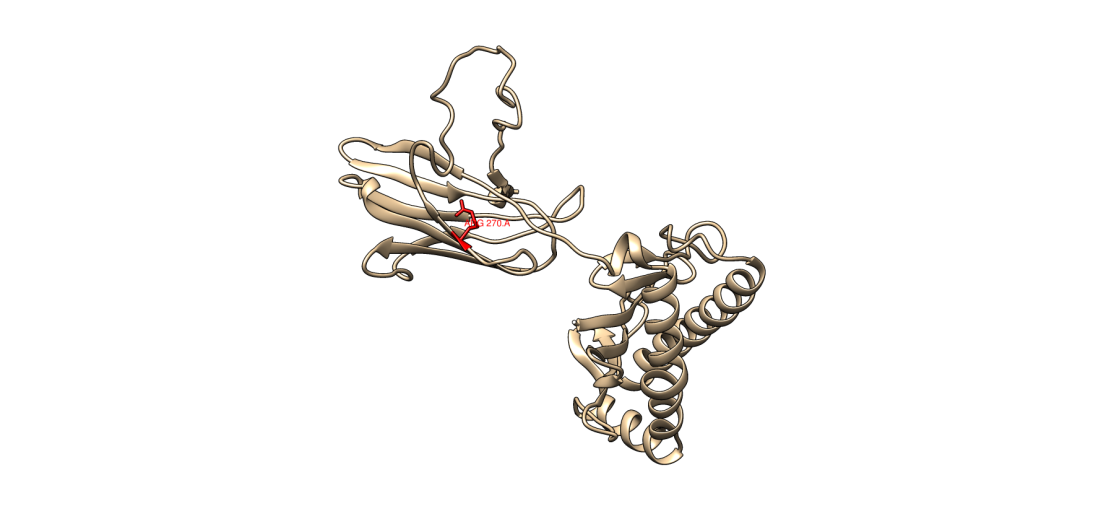 |
| **SNP ID: rs765275727** | |  | | | |
| **protein position 298 changed from Tryptophan (green (upper image)) to  Arginine (red (bottom image))** | 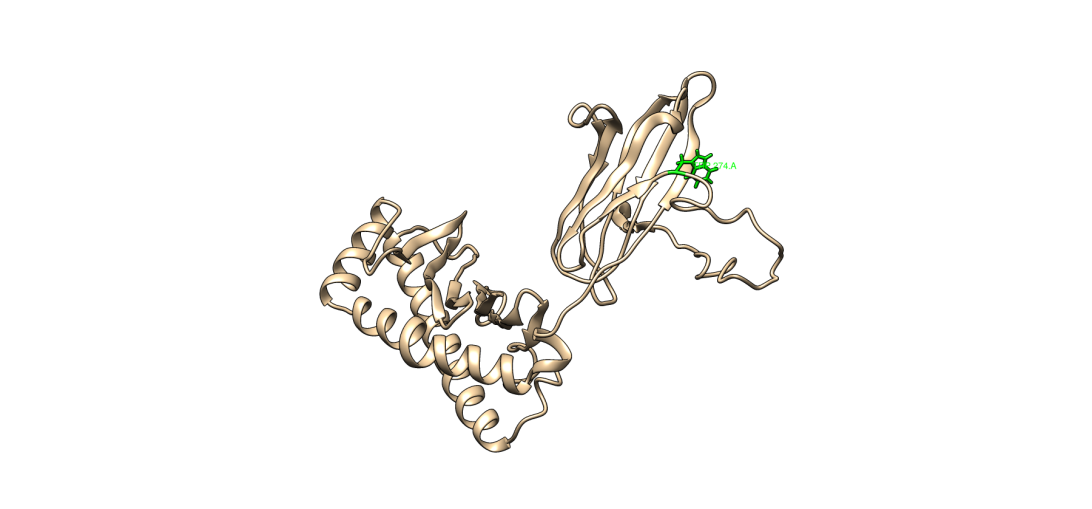 |  | | | |
|  | 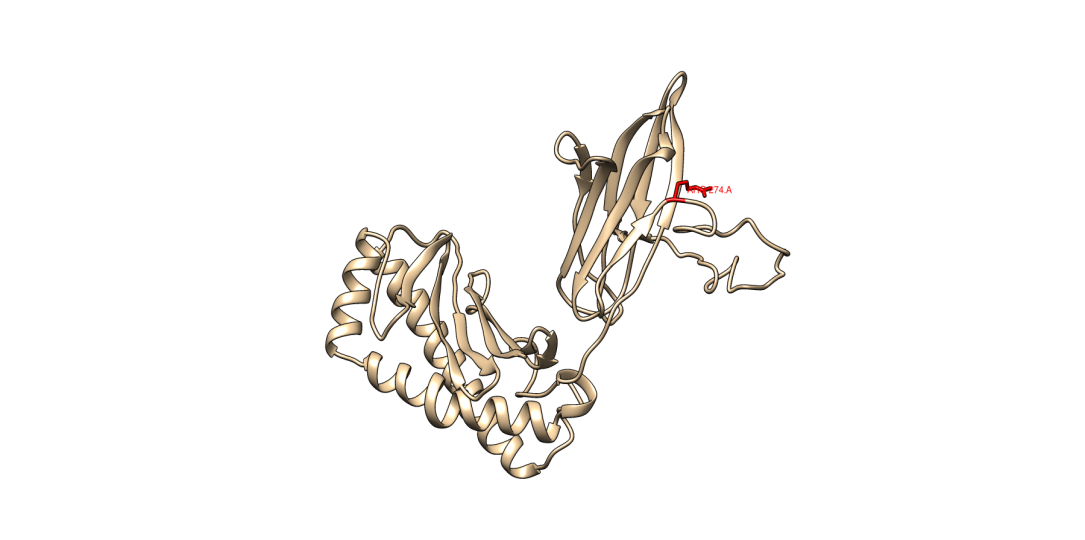 |  | | | |
